# Development of Novel Imipridones with Alkyne- and Triazole-Linked Warheads on the Tricyclic Skeleton, Showing Superior Ability to Eradicate PANC-1 and Fadu Cells Compared to ONC201

**DOI:** 10.3390/ijms252313176

**Published:** 2024-12-07

**Authors:** Tamás Czuczi, József Murányi, István Móra, Bianka Gurbi, Attila Varga, Dávid Papp, Gitta Schlosser, Miklós Csala, Antal Csámpai

**Affiliations:** 1 Department of Organic Chemistry, Eötvös Loránd University (ELTE), Pázmány P. Sétány 1/A, H-1117 Budapest, Hungary; czuczi.tamas@gmail.com (T.C.); jozsefmuranyi84@gmail.com (J.M.); 2 Hevesy György PhD School of Chemistry, Pázmány P. Sétány 1/A, H-1117 Budapest, Hungary; david.papp@ttk.elte.hu; 3 Department of Molecular Biology, Semmelweis University, Tűzoltó u. 37-47, H-1094 Budapest, Hungary; istvan.mora1313@gmail.com (I.M.); gurbi.bianka@semmelweis.hu (B.G.); varga.attila3@semmelweis.hu (A.V.); 4 MTA-ELTE Lendület (Momentum) Ion Mobility Mass Spectrometry Research Group, Institute of Chemistry, ELTE Eötvös Loránd University, Pázmány Péter Sétány 1/A, H-1117 Budapest, Hungary; gitta.schlosser@ttk.elte.hu

**Keywords:** imipridone, propargylamine, 1,2,3-triazole, CuAAc reaction, Sonogashira coupling, PANC-1 and Fadu cells, cell viability, dose–response measurements, colony formation, complete eradication of cancer cells, ClpP-independent mechanism

## Abstract

Our ongoing research focuses on the development of new imipridone derivatives. We aim to design compounds that can completely and selectively eradicate cancer cells after relatively short treatment. We have synthetized systematically designed novel hybrids and evaluated their antiproliferative activity against PANC-1 and Fadu cell lines. We have also conducted preliminary studies on the mechanism, including colony formation as well as dose–response tests in HEK293T wild-type (WT) and HEK293T CLPP^−/−^ cells. Following gradual structural fine-tuning based on high throughput screening, we identified two imipridone hybrids as the most potent derivatives. Their unique substitution pattern includes *N*-methylated propargylamine and ferrocenyl/phenyltriazole moieties on the benzyl groups attached to opposite sides of the imipridone core. We found that the compounds with IC_50_ values similar to those of **ONC201** completely eradicated cancer cells at about 4 μM, while **ONC201** treatment at even higher concentrations left 30–50% of viable cells behind. Both compounds exerted equal activity in WT and CLPP^−/−^ HEK293T cells, indicating a ClpP-independent mechanism. Further development is needed to improve the tumor selectivity of the two potent imipridone derivatives. By preserving tumor cytotoxicity, we aim to generate new drug candidates that evade resistance and can be applied in a sufficiently broad therapeutic window.

## 1. Introduction

**Figure 1:**
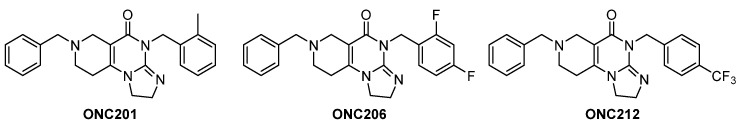
Emblematic imipridones engaged in clinical (**ONC201** and **ONC206**) or preclinical (**ONC212**) studies.

Imipridones constitute a novel class of small molecule anticancer agents with high oncotherapeutic potential. **ONC201** is the first-in-class lead compound of the imipridone family, which is currently engaged in multiple clinical trials for solid and hematological malignancies like gliomas, neuroendocrine tumors, multiple myeloma, breast cancer, endometrial cancer (https://clinicaltrials.gov/ accessed on 3 August 2023), featuring a wide therapeutic window and an exceptional safety profile [1]. Accordingly, the FDA has granted **ONC201** fast track designation for the treatment of adult recurrent H3 K27M-mutant HGG, rare pediatric disease designation for treatment of H3 K27M-mutant glioma, and orphan drug designations for the treatment of glioblastoma and malignant glioma. The anticancer activity of **ONC201** was originally identified in a target–agnostic screen searching for activators of TNF-related apoptosis-inducing ligand (TRAIL) in tumor cells [2]. Second-generation imipridones, such as **ONC206** and **ONC212** (Figure 1), were also identified as highly potent anticancer agents [3]. Compared to **ONC201**, **ONC206** demonstrated an improved inhibition of cell migration and was transferred to phase I trials in treatment of diffuse midline gliomas, while **ONC212** was tested in >1000 human cancer cell lines in vitro featuring the lowest efficient doses in this series, typically in nanomolar range, and was evaluated for safety and antitumor efficacy in vivo. **ONC212** exhibits rapid pharmacokinetics and selectively kills tumor cells. It has broad-spectrum preclinical activity across both solid tumors and hematological malignancies, including pancreatic cancer and leukemias prioritized for clinical indications [4]. These results indicate that significant improvements can be achieved by fluorinating the imipridone core at different positions.

Molecular targets of **ONC201** and other imipridones have been intensively investigated in the past few years in order to clarify their mechanisms of action. Besides inducing TRAIL, **ONC201** also upregulates the cell surface TRAIL receptor death receptor 5 (DR5) [5,6] and acts as a selective antagonist of dopamine receptor D2 (DRD2) [3]. It has also been identified as an allosteric agonist of mitochondrial protease caseinolytic protease P (ClpP) [7,8,9]. **ONC212**, in turn, selectively activates protein-coupled receptor 132 (GPCR-GPR132) [10]. Mounting evidence suggests that, among the multiple effects on various signaling pathways, it is the alteration of mitochondrial metabolism initiated by ClpP activation that constitutes the most potent and dominant pathway leading to apoptosis [11]. While ClpP is normally regulated by the chaperone ClpX, imipridones and related compounds cause unregulated hyperactivation of the protease. Subsequent structural and functional damage to mitochondria includes the degradation of respiratory complexes I and II and impairment of oxidative phosphorylation (OXPHOS). Degradation of mitochondrial respiratory proteins is accompanied by, among others, lower oxygen consumption rates, ATP depletion, increased amounts of mitochondrial reactive oxygen species (ROS), and depletion of mitochondrial DNA. These effects collectively activate the integrated stress response (ISR), which directs the cell to arrest the cell cycle and induce apoptosis. This impact is triggered by the disruption of mitochondrial function, regardless of p53 status [4,12]. Cancer cells are highly sensitive to chemical activation of ClpP, which does not induce death in normal cells despite a detectable degradation of ClpP substrate proteins [13]. This tumor-specific cytotoxicity and the wide therapeutic window of anticancer activity of imipridones may be due, in part, to an apparently lower dependence of normal cells on OXPHOS.

Imipridones exert their effects through different mechanisms of action. **ONC212** has been shown to induce cell cycle arrest in HeLa and A549 cells in the G1/G0 phase and to trigger caspase-3-mediated apoptosis independently of mitochondrial outer membrane permeabilization (MOMP) and without loss of mitochondrial membrane potential [14]. **ONC213**, a regioisomer of **ONC206**, in turn, exerts its antileukemic activity by suppressing mitochondrial respiration and α-ketoglutarate dehydrogenase (αKGDH) activity [15].

Although ClpP activators have promising antiproliferative and pro-apoptotic effects, their applicability is limited by the resistance of cancer cells. Several studies [12,13,14,15,16,17,18,19,20] have demonstrated the tumor cytotoxicity of imipridones in micro- or nanomolar concentrations, but the tumor cells were not eradicated in the assays presented. The dose–response curves revealed that approximately 10–50% of cells survived even prolonged exposure to higher concentrations of imipridone. The mechanisms underlying treatment resistance are not yet fully understood, but certain contributing elements are supported by strong evidence. The phenomenon can be at least partly attributed to the upregulation of insulin-like grow factor receptor IGF-R1 [12], endoplasmic reticulum chaperone GRP78/BIP [12], and anti-apoptotic Bcl-2 and Bcl-xL [14], as well as to a metabolic switch from impaired OXPHOS toward glycolysis and the consequential maintenance of ERK1/2 activity and survival [16].

**Figure 2:**
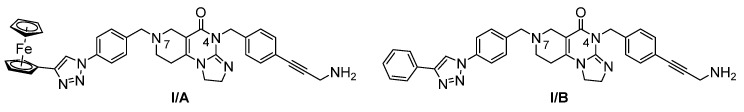
The most promising compounds identified in our previous work [23].

It is evident that electron transfer processes play a major role in the changes induced by imipridones in cancer cells. Therefore, we hypothesized that the tumor cytotoxicity of imipridone derivatives can be improved by boosting ROS formation in the mitochondria through supplementing the hybrid molecule with a ferrocene moiety that activates a Fenton-like pathway. Our approach is based partly on preclinical evidence for the cross-talk between TRAIL-induced apoptosis and redox signaling and its implications in cancer [21]. In this respect, we have already presented our study on ferrocene-impridone derivatives that exhibited more pronounced long-term cytotoxic effects on A2058 melanoma cell line than **ONC201** and **ONC212** [22]. These ferrocenylalkyl-substituted imipridones also displayed marked efficacy against COLO-205 and EBC-1 cell lines, presumably due to the activation of ROS-implicating apoptotic pathways by the ferrocenyl moieties and their contribution to the antiproliferative effect. Inspired by these results, we constructed a small library of organometallic imipridone hybrids, which contained ferrocene fragments tethered by triazole and/or alkyne linkers to different positions of the *N*4- and *N*5-benzyl groups pending from the imipridone core [23]. Complex evaluation of this set of hybrids identified propargylamines **I/A** and **I/B** as the most efficient antiproliferative agents (Figure 2).

Contrary to **ONC201**, these two compounds were able to eliminate resistance in PANC-1 pancreas ductal adenocarcinoma, Fadu pharyngeal squamous carcinoma, and A2058 and EBC-1 cell lines, and no viable cells could be observed after treatment during colony formation assays at a concentration of 10 µM. However, the IC_50_ values of these compounds were found to be 5–10 times higher than those of **ONC201**. On the other hand, we also demonstrated that in shorter treatment (24 h exposure time instead of 72 h) in PANC-1 and A2058 cells, **I/A** and **I/B** were far more efficient in apoptosis induction than **ONC201**, which caused no detectable sign of apoptosis after 24 h of exposure. Finally, in comparative cytotoxic studies on PANC-1 and A2058 cells and nontumorous primary fibroblasts, the organometallic derivative **I/A** emerged as the most potent anticancer agent. This ferrocene derivative proved to be capable of completely and selectively eradicating tumor cells at a concentration of 10 µM without exerting observable toxicity on fibroblasts.

In our current work, we aimed to expand the library of imipridone derivatives with members that exhibit selective anticancer activity, feature lower efficient doses and IC_50_ values compared to **I/A** and **I/B**, and maintain the potency to eradicate cancer cells completely. We anticipated that these goals could be achieved through a concerted cooperation of the ferrocenyl/aryltriazolyl substituent and the linear alkyne-tethered amine, with the two established pharmacophoric warheads placed in optimal relative positions on the terminal benzyl groups pending from the imipridone core.

**Figure 3:**
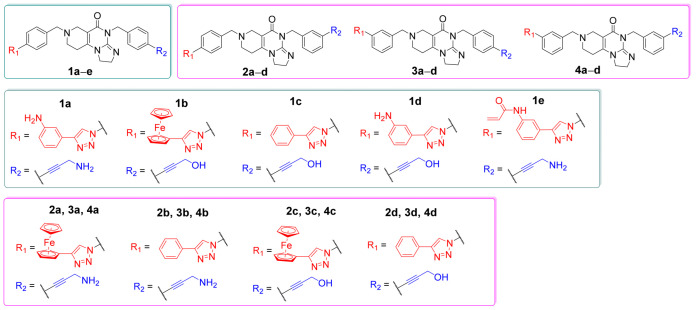
Various regioisomers and structurally related derivatives of **I/A** and **I/B** intended for synthesis and in vitro cell viability screening as members of the first set of compounds.

We started our diversity-oriented research by synthesizing an array of imipridone hybrids structurally related to **I/A** and **I/B** and carrying identical or systematically modified substituents in different positions of the terminal benzyl groups (**1a**–**e**, **2a**–**d**, **3a**–**d**, and **4a**–**d**: Figure 3). Compounds **1a**–**e** can be regarded as the derivatives of **I/A** and **I/B** with *para*-substituted benzyl groups on the terminals of the imipridone scaffold, ensuring maximal spatial separation of the arytriazole- and alkyne-containing functional groups, the two potential pharmacophoric warheads. Compounds **2a**–**4a** and **2b**–**4b**, containing at least one meta-substituted benzyl group, are regioisomers of **I/A** and **I/B**, respectively, with spatially less separated and differently oriented pharmacophoric warheads. As an additional variation, we also replaced the 3-aminopropynyl moiety with a 3-hydroxypropynyl group in hybrids **1b**–**d**, **2c**,**d**, **3c**,**d**, and **4c**,**d** to investigate the role of the basicity of this outward functional group. Moreover, for this comprehensive study, which also aimed to conduct a preliminary investigation of the mechanism of action, we synthetized hybrid **1e**, an acroylamino analog of **I/B**, which might be able to covalently bind to cysteine-containing cellular targets.

## 2. Results and Discussion

### 2.1. Multistep Synthesis of the First Group of the Targeted Imipridone Hybrids

**Scheme 1:**
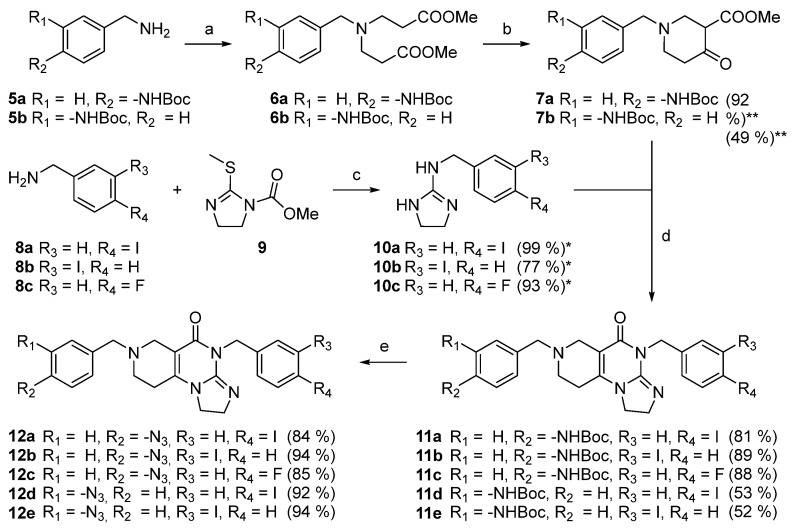
Convergent synthetic pathway leading to halogenated azidoimipridones. Reaction conditions: (a) methyl acrylate (5 eq.), MeOH, r.t., 48 h; (b) NaH (5 eq.), THF, 0 °C, then r.t., 2 h; (c) MeOH/AcOH = 4/1, reflux, 18 h; (d) 3.6 M NaOMe/MeOH (1.25 eq.), MeOH, reflux, 12 h; (e) (1) cc. HCl, r.t., 30 min. (2) NaNO_2_ (2 eq.), 0 °C, 45 min; (3) NaN_3_ (10 eq.), 0 °C, 45 min, then r.t., 1 h; (* = crude product; ** = cumulative yield, crude product).

We synthesized the designed derivates following the multistep convergent synthetic pathway described in our previous publication [23]. Accordingly, by means of double acrylate addition followed by base-catalyzed cyclization, *mono*-Boc-protected 3- and 4-aminobenzylamines **5a** and **5b** were converted into piperidones **7a** and **7b**, respectively (**5** → **6** → **7**: Scheme 1). On the other hand, 3- and 4-halobenzylamines **8a**–**c** were coupled with activated methyltioimidazoline **9**, affording cyclic guanidines **10a**–**c**, which were then reacted without purification with **7a**,**b** to construct angular tricyclic imipridones **11a**–**e** carrying a Boc-protected amino group (Scheme 1). By sequential *N*-deprotection, diazotation, and treatment with an excess of NaN_3_, **11a**–**e** were converted into regioisomeric halogenated azides **12a**–**e** in good yields, as required for the preparation of the designed hybrids (Scheme 1).

**Scheme 2:**
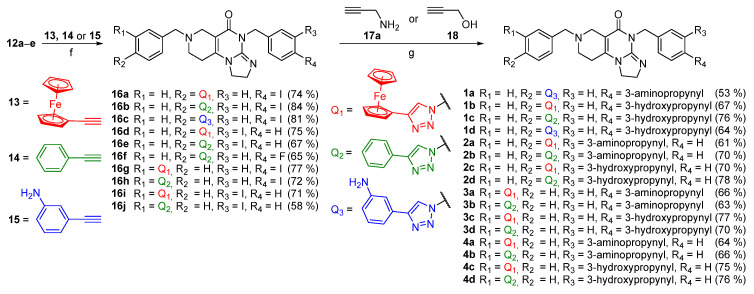
Sequential functionalization reactions on the terminal benzyl groups of the imipridone scaffold affording the first group of the targeted hybrids. Reaction conditions: (f) CuI (0.1 eq.), DMSO, r.t., 24 h; (g) CuI (0.2 eq.), PdCl_2_(PPh_3_)_2_ (0.1 eq.), DIPEA (3 eq.), DMF, r.t., 24 h.

We continued with copper-catalyzed azide-alkyne cycloadditions of **12a**–**e** using ethynylferrocene (**13**), phenylacetylene (**14**), and 3-aminophenylacetylene (**15**) as coupling partners, resulting in triazoles **16a**–**j** in mediocre to good yields (Scheme 2). Afterward, switching to the conditions of the Sonogashira reaction, iodinated 1,3-triazolyl compounds **16a**–**e** and **16g**–**j** were coupled with propargylamine **17a** and propargyl alcohol **18** to obtain **1a**–**d**, **2a**–**d**, **3a**–**d**, and **4a**–**d**, the first set of the designed compounds intended for in vitro viability screening (Scheme 2).

**Scheme 3:**
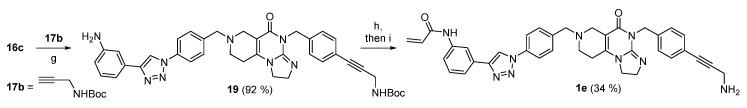
Synthesis of acrylamido derivative **1e** employing a three-step procedure supported by a selective *N*-protection strategy: Reaction conditions: (g) CuI (0.2 eq.), PdCl_2_(PPh_3_)_2_ (0.1 eq.), DIPEA (3 eq.), DMF, r.t., 24 h; (h) acryloyl chloride (1.3 eq.), DIPEA (2 eq.), DCM, r.t., 4 h.; (i) cc. HCl, r.t., 20 min.

Starting from **16c**, acrylamido derivative **1e** was accessed by employing primary Sonogashira coupling with *N*-Boc propargylamine **17b**, followed by standard acryloylation and sequence-terminating deprotection of the propargylamine moiety (Scheme 3).

### 2.2. In Vitro Cell Viability Screening of the First Set of the Novel Imipridone Hybrids

We performed cell viability screening on PANC-1 and Fadu human cancer cell lines with the first set of compounds at three concentrations (2.8, 8.3, and 25 μM: Table 1) to establish structure–activity relationships. The same cell lines were used in our previous work for antiproliferative assessment [23].

The experiments conducted at a concentration of 25 µM imipridone disclosed the most characteristic structure–activity relationships, which indicated that the presence of the protruding basic amino group, kept at a constant distance from the imipridone residue by the rigid linker, was indispensable for the desired drop in viability to 0–1% in either of the cancer cell lines. This was reflected by the viability data measured exclusively for aminopropynyl derivatives **I/B**, **2a**,**b**, **3a**,**b**, and **4a**,**b**, while the hydroxypropynyl compounds **1b**–**d**, **2c**,**d**, **3c**,**d**, and **4c**,**d** as well as the fluorinated derivative **16f** displayed substantially lower activity even at this high concentration. Moreover, the results unambiguously showed that, at a concentration of 8.3 µM, only ferrocenyltriazolyl derivative **2a** and its phenyltriazolyl counterpart **2b** could achieve practically complete eradication of both types of cancer cells. They outperformed their regioisomers **I/A** and **I/B**, respectively, and every other member of the first set of novel imipridone hybrids screened. It is worth pointing out that the substantial boost in activity reflected by the relative performance of the **I/A**-**2a** and **I/B**-**2b** isomer pairs was definitely associated with a change in the orientation of the rigid basic side chain. This pharmacophoric warhead was moved from the *para* to *meta* position on the *N*4-benzyl group, with the *para* position of the ferrocenyl/phenyltriazolyl residue retained on the *N*7-benzyl group pending from the imipridone core. Other isomers of **2a** and **2b** (**3ab** and **4ab**), along with further members of the first set of hybrids, showed little or no improvement in efficacy compared to **I/A** and **I/B**. Accordingly, we selected **2a** and **2b** as the lead compounds for further structural optimization.

Finally, it should also be highlighted that **4c** and **4d** hydroxypropynyl derivatives proved to be the best performing derivatives at 2.8 µM, even though they were not able to completely abolish cell viability at any concentration. In this regard, they were more similar to the original imipridone **ONC201** than **I/A** or **I/B** in terms of their cytotoxic profile.

### 2.3. Design of the Second Group of Potential Drugs with Activity Expected to Be Superior to That Displayed by the Members of the First Set of Hybrids

**Figure 4:**
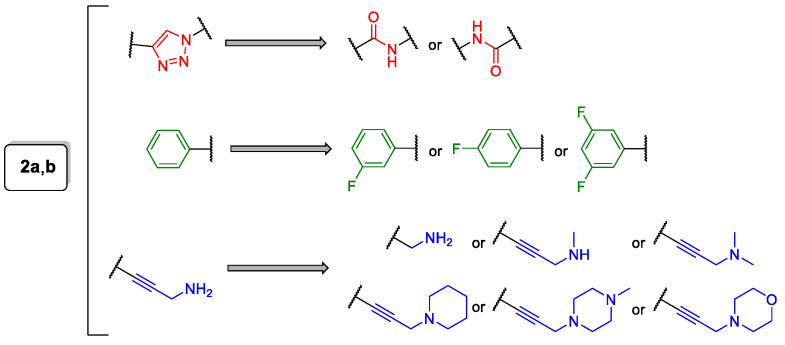
Envisaged fragment refinements in **2a**,**b**, the members of the first set of the targeted hybrids that proved to be prominently efficient in the primary viability screening.

With the overarching goal of identifying additional hybrids with further increased anticancer potency, we also devised strategies for reasonable fragment refinements in **2a**,**b** (Figure 4), the two compounds showing the greatest efficiency in viability screening. Since **1a**–**e**, the *bis*-*para*-benzyl substituted analogs of **I/A** and **I/B**, hydroxypropynoyl derivatives **2c** and **2d**, as well as regioisomers **3a**–**d** and **4a**–**d** displayed decreased activity during the primary screening, we did not consider their structural tune-up for subsequent molecular design. On the other hand, since **2a** and **2b** displayed superior activity compared to their isomers **I/A** and **I/B**, respectively, these models were selected for the following structural modifications of the terminal functional groups. We planned to replace the triazole ring with an amide bond, generally recognized as its bioisostere [24,25,26,27]. Additional envisaged modifications included the introduction of fluorine substituents in the phenyl ring of the 4-phenyl-1*H*-1,2,3-triazol-1-yl moiety, fine-tuning the basicity of the 3-aminopropynyl nitrogen, and, by exclusion of the carbon–carbon triple bond, reducing the distance between the phenyl ring and the amino group, which might be critical for antiproliferative activity. These modifications were motivated by the potential of the redesigned functionalization to influence parameters such as polarity, basicity, conformation, and membrane permeability, as well as the pharmacodynamic profile of the drug candidates.

### 2.4. Multistep Synthesis of the Second Group of the Targeted Imipridone Hybrids

**Scheme 4:**
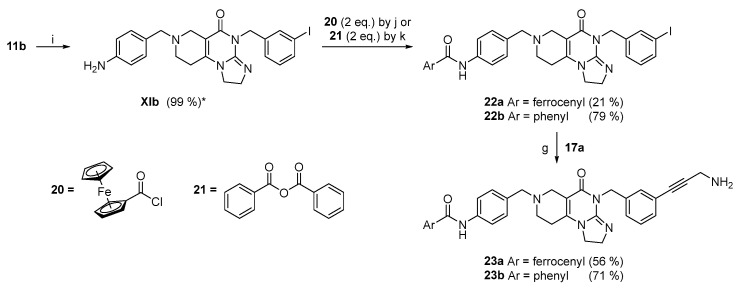
Synthetic route to the first type of amido-imipridone derivatives. Reaction conditions: (g) propargylamine (2 eq.), CuI (0.2 eq.), PdCl_2_(PPh_3_)_2_ (0.1 eq.), DIPEA (3 eq.), DMF, r.t., 24 h; (i) cc. HCl, r.t., 20 min; (j) DIPEA (3 eq.), DCM, r.t., 1.5 h; (k) DIPEA (3 eq.), DCM, reflux, 24 h; * = crude product.

**Scheme 5:**
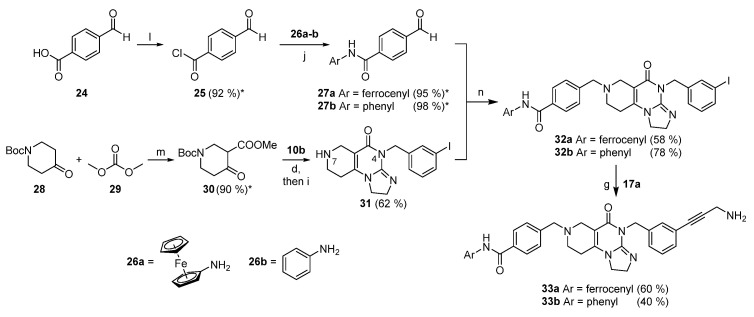
Synthetic route to the second type of amido-imipridone derivatives. Reaction conditions: (d) 3.6 M NaOMe/MeOH (1.25 eq.), MeOH, reflux, 12 h; (g) propargylamine (2 eq.), CuI (0.2 eq.), PdCl_2_(PPh_3_)_2_ (0.1 eq.), DIPEA (3 eq.), DMF, r.t., 24 h; (i) cc. HCl, r.t., 20 min; (j) DIPEA (3 eq.), DCM, 0 °C, 30 min., then r.t., 1 h; (l) oxalyl chloride (2 eq.), DMF (cat.), DCM, r.t., 20 min; (m) NaH (5 eq.), toluene, r.t., 3 h, then reflux 2 h; (n) (1) DCM, r.t., 20 min., (2) NaHB(OAc)_3_ (1.4 eq.), r.t., 2.5 h.; * = crude product.

We prepared two types of isomeric amides carrying the *N*-acylamino- or *N*-arylcarbamoyl moiety in the *para* position of the *N*7-benzyl group with 3-aminopropynylbenzyl group pending from the *N*4 position of the central imipridone core (*N*-acylamino derivatives **23a**,**b** and *N*-arylcarbamoyl derivatives **33a**,**b** presented in Scheme 4 and Scheme 5, respectively). *N*-Ferrocenyl- and benzoylamino derivatives **23a**,**b** were accessed by a three-step procedure, starting with the acid-catalyzed deprotection of Boc-protected amine **11b** (cf. Scheme 1), followed by sequential *N*-acylation of the deprotected iodoamine **XIb** with ferrocenoylchloride **20** and benzoic acid anhydride **21**, and terminating with propargylamine-mediated Sonogashira coupling of the resulting amides **22a**,**b** conducted under standard conditions (Scheme 4).

The second type of amides, **33a**,**b**, were accessed by multistep convergent synthetic pathways (Scheme 5). The first pathway led to *N*-arylamides of 4-formylbenzoic acid **27a** and **27b** obtained from 4-formylbenzoylchloride **25** by treatment with aminoferrocene **26a** and aniline **26b**, respectively. The second pathway started with carboxymethylation of *N*-Boc-oxopiperidine **28** effected by dimethyl-carbonate-mediated **29**. The generated oxocarboxylate **30** was condensed with 3-iodobenzyl-substituted 2-amino-3,4-dihydroimidazole (**10b**: cf. Scheme 1), constructing the *N*7-Boc protected imipridone scaffold of which treatment with concentrated hydrochloride acid afforded 4-(3-iodobenzyl)-substituted cyclic secondary amine **31**. Finally, the convergent synthesis was continued by triacetoxyhydroborate-mediated reductive alkylation of **31** by **27a** and **27b** to obtain amides **32a** and **32b**, respectively, which were then subjected to Sonogashira coupling with propargylamine **17**, resulting in the formation of the targeted imipridone models **33a**,**b**.

**Scheme 6:**
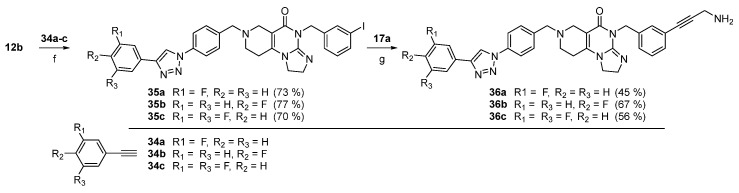
Synthetic route to **36a**–**c**, the fluorinated derivatives of **2b**. Reaction conditions: (f) CuI (0.1 eq.), DMSO, r.t., 24 h; (g) propargylamine (2 eq.), CuI (0.2 eq.), PdCl_2_(PPh_3_)_2_ (0.1 eq.), DIPEA (3 eq.), DMF, r.t., 24 h.

We also synthesized compounds **36a**–**c**, the fluorinated analogs of **2b**, by the sequence involving two orthogonal transition metal-catalyzed procedures (Scheme 6). In the first step, the CuAAc coupling of azido-imipridone **12b** (cf. Scheme 1) and fluorinated phenylacetylenes **34a**–**c** afforded iodinated fluorophenyltriazoles **35a**–**c**, which were then converted into **36a**–**c** under the conditions of the Sonogashira reaction using propargylamine **17a** as the alkyne component.

**Scheme 7:**
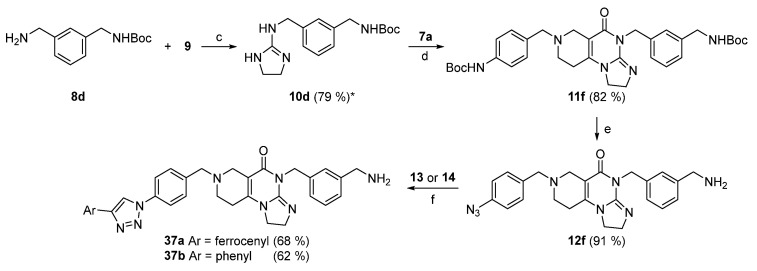
Synthetic route to the aminomethyl-imipridone derivatives. Reaction conditions: (c) MeOH/AcOH = 4/1, reflux, 18 h; (d) 3.6 M NaOMe/MeOH (1.25 eq.), MeOH, reflux, 12 h; (e) (1) cc. HCl, r.t., 30 min (2) NaNO_2_ (2 eq.), 0 °C, 45 min (3) NaN_3_ (10 eq.), 0 °C, 45 min, then r.t., 1 h; (f) CuI (0.1 eq.), DMSO, r.t., 24 h; * = crude product.

To achieve the envisaged distance shortening between the protruding basic amino group and the aryltriazolyl-substituted imipridone scaffold (see: Section 2.3), the aminopropynyl group was replaced with an aminomethyl group by the reaction sequence outlined in Scheme 7. Accordingly, *N*-Boc-3-(aminomethyl)benzylamine **8d** was incorporated in doubly protected imipridone-based diamine **11f** by guanidine formation (**8d** + **9** → **10d**) and sequential annulation of intermediate **10d** with methoxycarboxylated pipridone **7a**. The standard deprotection of **11f** was followed by the selective diazotation-azidation sequence, exploring the marked difference between the basicity of the aniline- and benzylamine moieties to obtain azide **12f**, which was finally reacted with alkynes **13** and **14** under the conditions of CuAAc to obtain the targeted imipridone hybrids **37a** and **37b**, respectively.

**Scheme 8:**
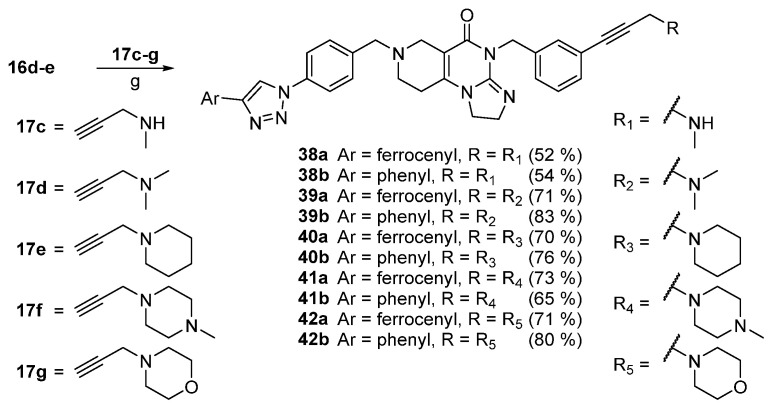
Synthesis of *N*-alkylated derivatives of **2a**–**b**. Reaction conditions: (g) CuI (0.2 eq.), PdCl_2_(PPh_3_)_2_ (0.1 eq.), DIPEA (3 eq.), DMF, r.t., 24 h.

Finally, as the results of the first screening suggested that a basic aminopropynyl group was more beneficial in reducing cell viability than a hydroxypropynyl group (comparing the activity of **2a**,**b** and **2c**,**d**: Section 2.2 and Table 1), we set out to investigate the effect of fine-tuning the basicity and size on antiproliferative activity. Thus, we introduced methylamino-, dimethylamino-, piperidinyl-, *N*-methylpiperazinyl-, and morpholinyl groups in the aryltriazolyl-substituted imipridone scaffold using Sonogashira coupling of iodinated precursors **16a**–**d** with *N*-propargylated amines **17c**–**g**, constructing a further subset of alkyne-tethered hybrids (**38a**,**b**, **39a**,**b**, **40a**,**b**, **41a**,**b** and **42a**,**b**: Scheme 8), the evaluation of which might lead to the recognition of further structure–activity relationships.

### 2.5. In Vitro Cell Viability Screening of the Second Set of the Novel Imipridone Hybrids

To see the outcome of the structural modifications, we performed cell viability screening on the second set of compounds using the same protocols applied when evaluating the first set of models on PANC-1 and Fadu human cancer cell lines at three concentrations (2.8, 8.3, and 25 μM). The results are summarized in Table 2.

The cell viability values measured for **23a**,**b**, **33a**,**b**, and **37a**,**b** helped to screen out certain fruitless structural modifications that did not enhance the cytotoxicity of the compounds. These were the replacement of triazole by one of the two tested amides (see **23a** and **33a** or **23b** and **33b**) and the shortening of the basic side chain (see **37a**,**b**).

On the other hand, mono- and difluorination at different sites of the phenyl group attached to the triazole ring led to a slight increase in efficacy at 8.3 µM on Fadu cells but did not have an appreciable influence on the activity against PANC-1 cells under the screening conditions. This was reflected by the comparison of the data obtained for parent compound **2b** and its fluorinated analogs **36a**–**c** (Table 2). It is of pronounced importance that *N*-methylated compounds **38a**,**b** were identified as the most potent models displaying the highest efficacy at the lowest concentration used in the screening, markedly outperforming primary amines **2a**,**b** and **36a**–**c**, particularly against PANC-1 cells. On the other hand, the viability data obtained with **39a**,**b**, **40a**,**b**, **41a**,**b**, and **42a**,**b** indicated that further *N*-methylation or attachment of a six-membered cyclic amine to the propargyl residue substantially decreased the efficacy of the resulting tertiary amines; however, the basicity apparently slightly modulated their activity, as reflected by the data measured for *N*-methylpiperazinyl and morpholinyl derivatives, the latter being almost completely ineffective against PANC-1 cells even at a concentration of 25 µM. This spectacular drop in efficacy can probably be ascribed to the increased bulkiness and/or the notably decreased basicity of the amine warhead.

**Scheme 9:**
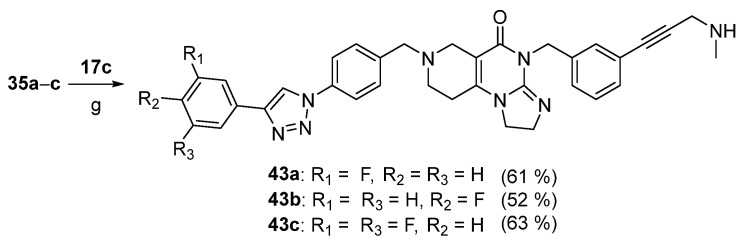
Synthesis of *N*-methylated derivatives **43a**–**c** carrying fluorinated phenyl groups on the triazolyl substituent. Reaction conditions: (g) CuI (0.2 eq.), PdCl_2_(PPh_3_)_2_ (0.1 eq.), DIPEA (3 eq.), DMF, r.t., 24 h.

Finally, based on the screening results, we hypothesized that fluorination of the triazolyl-attached phenyl group along with monomethylation of the propargylamine moiety could further enhance the efficacy in an additive, even in a synergistic manner, leading to the development of the most potent representatives of the impiridone hybrids with aminopropynyl and aryltriazolyl warheads. Accordingly, employing Sonogashira coupling of fluorinated iodoimipridones **35a**–**c** with *N*-methylpropargylaine **17c**, we prepared **43a**–**c** as the last subset of our targeted hybrids (Scheme 9).

### 2.6. Dose–Response Study on Selected Compounds

**Figure 5:**
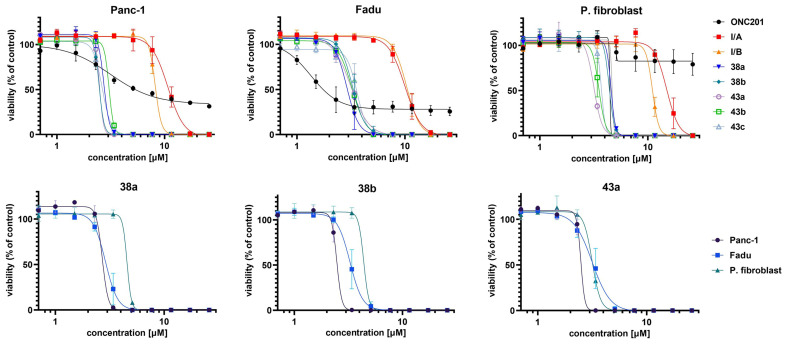
Dose–response curves of selected imipridone hybrids **I/A**, **I/B**, **38a**,**b**, **43a**–**c**, and **ONC201** in PANC-1 and Fadu cells and non-tumorous primary fibroblast cells measured by the CellTiter-Glo cell viability assay. Curves were fitted by GraphPad Prism 9.5.0 software using four-parameter nonlinear regression (variable slope; 95% CI; n = 3). The concentration scale is logarithmic.

The most efficacious compounds from the second set (**38a**,**b**) and **43a**–**c**, the fluorinated analogs of **38b**, were selected for systematic dose–response studies and determination of IC_50_ values. The measurements were conducted on PANC-1 and Fadu cells and non-tumorous primary fibroblast cells to assess the cytotoxicity profile and therapeutic window of the investigated compounds. For the sake of consistency, the dose–response curves were also detected for **ONC201**, **I/A**, and **I/B**, which served as relevant reference standards. The resulting dose–response curves are depicted in Figure 5, and the corresponding IC_50_ values are listed in Table 3 (see also Supplementary S.89, S.90).

Based on the results of the viability screening and the concept of their rational design, we expected compounds **43a**–**c** to display efficacy superior to that of **38a**,**b**. However, the graphs and IC_50_ values indicated that **38a**,**b** and **43a**–**c** all exerted highly similar activity against PANC-1 and Fadu cells, but compared to **38a**,**b**, fluorinated analogs **43a**–**c** proved to be more cytotoxic toward the non-tumorous primary fibroblast cells, featuring minimal if any therapeutic window, as indicated by the selectivity index (SI) falling in the range of 1.0–1.4 (Table 3, columns 4 and 5). Pointing to a characteristic structure–activity relationship, compounds **38a**,**b** and **43a**–**c** with the 1,3-disubstitutited *N*4-benzyl group produced 3–4 times lower IC_50_ values in the two investigated cancer cell lines than **I/A** and **I/B,** the previously published most potent imipridone derivatives carrying the 1,4-disubstitutited benzyl group on *N*4. More importantly, besides the complete eradication of both types of cancer cells, **38a**,**b** and **43a**–**c** slightly outperformed **ONC201** in PANC-1 cells in terms of efficient dose, characterized by the IC_50_ values. Nevertheless, the first-in-class imipridone featured the lowest IC_50_ along with the highest SI in Fadu cells.

Taken together, **38a** and **38b** proved to be the most promising molecules among the novel imipridone hybrids presented in this study. However, their SI did not reach the acceptable level, and the concentration range (3–4 µM) in which these halogen-free drug candidates could almost completely eradicate both PANC-1 cells and Fadu cells, while displaying little to no cytotoxic effect toward non-tumorous fibroblast cells, was very narrow.

### 2.7. Colony Formation Assay

**Figure 6:**
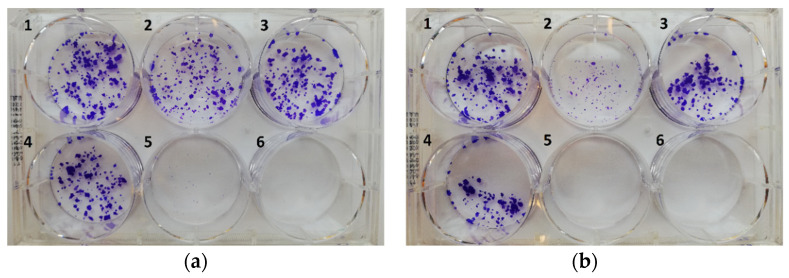
Colony formation assay of selected imipridone derivatives with PANC-1 cell line at a concentration of 4 µM: (**a**) 24 h treatment followed by 5 days post-incubation; (**b**) 72 h treatment followed by 3 days post-incubation. 1: DMSO (control), 2: **ONC201**, 3: **I/A**, 4: **I/B**, 5: **38a**, and 6: **38b**. Representative images are shown; n = 2.

Based on the dose–response analysis, the halogen-free molecules **I/A**,**B** and **38a**,**b** were selected for a comparative colony formation assay to test whether they could prevent tumor cells from forming colonies. PANC-1 cells were seeded and grown in the absence or presence of the tested imipridone derivatives for different periods of time. The selected compounds were applied at a concentration of 4 µM, and the prototypical **ONC201** was used as a positive control. Due to nearly complete elimination of cell colonies in some wells, representative images are shown in Figure 6 instead of statistical analysis.

Compounds **38a**,**b** substantially reduced the cancer cell population and left only traces of viable cells even at shorter (24 h) exposure. Following a longer (72 h) exposure with these hybrids, no viable cells were discernible in contrast to **ONC201** and **I/A**,**B**. The results further supported the outstanding efficacy of the newly designed **38a**,**b** in comparison with **ONC201** and the parent compounds **I/A**,**B**.

### 2.8. ClpP-Independent Cytotoxicity of the Selected Imipridone Hybrids

**Figure 7:**
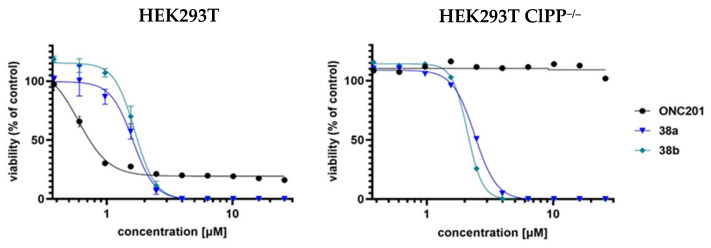
Dose–response curves of imipridone hybrids **38a**,**b** and **ONC201**. Viability measured in HEK293T and HEK293T^−/−^ cell lines based on the CellTiter-Glo cell viability assay. Curves were fitted by GraphPad Prism 9.5.0 software using nonlinear regression (variable slope; 95% CI; n = 4). The concentration scale is logarithmic. The R^2^ values were consistently above 0.95.

We also tested the role of mitochondrial ClpP activation in the cytotoxic activity of the most potent imipridone hybrids **38a**,**b** by comparing the dose–response curves from viability assays with HEK293T and HEK293T CLPP^−/−^ cells. This approach is a well-established strategy for assessing the involvement of ClpP in the action of antiproliferative agents [28,29]. As was expected, the reference imipridone **ONC201** had no effect on the viability of HEK293T CLPP^−/−^ cells due to the lack of targetable ClpP, while the effect of **38a**,**b** was maintained in the ClpP-deficient cells (Figure 7), as was also reflected by the IC_50_ values (Table 4). The results clearly indicated that the studied hybrid compounds reduced cell viability in a ClpP-independent manner under the applied experimental conditions. It has been revealed by in-depth X-ray diffraction analysis that the intercalation of the two terminal benzyl groups into the hydrophobic binding pockets of the ClpP heptamer is critical for the formation of the activated peptidase complex [9]. This intercalation was probably prevented by the two bulky and polar warheads of **38a**,**b**. The activity of these compounds, on the other hand, might be attributed to the optimal separation and relative orientation of these warheads ensured by the imipridone skeleton. In addition, the tricyclic heterocycle comprising H-bond acceptor sites may also contribute to the antiproliferative efficiency by binding to yet unidentified cellular target(s).

## 3. Materials and Methods

All chemicals were obtained from commercially available sources (Merck, Budapest, Hungary; Fluorochem, Headfield, UK; Molar Chemicals, Halásztelek, Hungary; VWR, Debrecen, Hungary) and used without further purifications. Merck Kieselgel (230–400 mesh, 60 Å) was used for flash column chromatography. Melting points (uncorrected) were determined with a Büchi M-560. The ^1^H- and ^13^C-NMR spectra were recorded in CDCl_3_ or in DMSO-*d*_6_ solution in 5 mm tubes at room temperature on a Bruker DRX-500 spectrometer (Bruker Biospin, Karlsruhe, Baden Württemberg, Germany) at 500 (^1^H) and 125 (^13^C) MHz, with the deuterium signal of the solvent as the lock and TMS as internal standard (^1^H and ^13^C). The 2D-HSQC and HMBC spectra, which supported the exact assignments of the ^1^H- and ^13^C-NMR signals, were registered using standard Bruker pulse programs. Exact mass measurements were performed using a high-resolution Waters ACQUITY RDa detector (Waters Corp., Wilmslow, UK) equipped with an electrospray ionization source using on-line UHPLC coupling. UHPLC separation was performed on a Waters ACQUITY UPLC H-Class PLUS system using a Waters Acquity UPLC BEH C18 column (2.1 × 150 mm, 1.7 µm). Samples were dissolved in MeOH/Water 5:95 *V*/*V*, and 5–5 µL volumes were injected. Linear gradient elution (0 min 5% B, 1.0 min 5% B, 7.0 min 80% B, 7.1 min 100% B, 8.0 min 100% B, 8.1 min 5% B, 12.0 min 5% B) was performed at a flow rate of 0.200 mL/min and a column temperature of 45 °C. Eluent A was 0.1 *V*/*V* % formic acid in water, and eluent B was 0.1 *V*/*V* % formic acid in methanol. High-resolution mass spectra were acquired in the *m*/*z* 50–2000 range in positive ionization mode. Leucine enkephalin peptide was used for single lock mass calibration correction.

The detailed spectral characterizations with the ^1^H- and ^13^C-NMR and HRMS data of the targeted hybrid compounds screened in the biological assays (Table 1 and Table 2) are listed in the Supplementary Material (S1). The structures of other intermediates can be regarded as chemically evidenced through the spectroscopically confirmed structures of the derived imipridone hybrids.

For each spectroscopically characterized compound, the numbering of atoms used for the assignment of the ^1^H- and ^13^C-NMR signals do not correspond to IUPAC rules, as reflected by the given systematic names (Supplementary Material).

Assignments of the NMR data, copies of the NMR spectra of the novel screened imipridone hybrids (S2–S13), and copies of the ^1^H- and ^13^C-NMR spectra (S14–S50) and the HRMS spectra (S51–S87) are included in the Supplementary Materials.

High-performance liquid chromatograms were acquired on a Jasco HPLC system (ABL&E-JASCO Hungary Ltd., Budapest, Hungary), using an Avantor ACE C18-PFP (100/4.00 mm; 3 μm; 100 Å; VWR) column. The HPLC column, acetonitrile (cat. no.: 83639.320), and TFA (cat. no.: 153112E) were obtained from VWR. Data were analyzed using ChromPass 1.8 software. Samples were dissolved in acetonitrile (injected volume: 5–10 µL). Linear gradient elution (0 min: 15% B; 7 min: 100% B; 11 min) of eluent A (0.1% TFA in water) with eluent B (acetonitrile/water 9:1 mixture complemented with 0.1% TFA) was performed at a flow rate of 0.8 mL/min (detection: 254 nm). HPLC chromatograms of the selected compounds are shown in the Supplementary Material (S.88).

### 3.1. Synthetic Procedures

The yields of the compounds for which their preparation is presented in the general procedures are listed in Scheme 1, Scheme 2, Scheme 3, Scheme 4 and Scheme 5.

#### 3.1.1. General Procedure for the Synthesis of the Methyl 1-(Tert-butoxycarbonylaminobenzyl)-4-oxopiperidine-3-carboxylates **7a**,**b** (Methods a and b)

Methyl acrylate (15 mmol, 5 eq.) was added to a solution of a 3-(Boc-amino)benzylamine (**5a**) or 4-(Boc-amino)benzylamine (**5b**) (3 mmol, 1 eq.) in methanol (5 mL), and the mixtures were stirred at room temperature for 48 h. The solutions were concentrated in vacuo to obtain crude diester products **6a**,**b**. Without further purification, they were dissolved in dry THF (8 mL), and NaH (15 mmol, 5 eq.) was added to the solutions gradually at 0 °C while stirring the mixtures. The suspensions were cooled down to room temperature. After stirring for an additional 2 h, the suspensions were poured over crashed ice (100 g), and the pH of the resulting slurry was set to 7 with acetic acid. The aqueous phases were extracted with ethyl acetate (3 × 40 mL). The combined organic phases were dried over Na_2_SO_4_ and evaporated to dryness in vacuo to obtain **7a**,**b**, which were used without further purification in the subsequent cyclization step.

#### 3.1.2. Synthesis of 2-(Methylthio)-4,5-dihydro-1H-imidazole-1-carboxylate (**9**)

2-Methylthio-4,5-dihydroimidazolium iodide (50 g, 0.205 mol, 1 eq.) and triethylamine (70 mL, 50.8 g, 0.502 mol, 2.45 eq.) were dissolved in DCM (250 mL). The solution was cooled down to 0 °C. Methylchloroformate (22 mL, 27 g, 0.28 mol, 1.4 eq.) was added dropwise to this cold solution, which was then allowed to warm up to room temperature, stirred overnight, and concentrated in vacuo. After the addition of EtOAc (400 mL), the suspension was stirred for 30 min. The precipitated ammonium salts were filtered off and washed with EtOAc (2 × 50 mL). The combined solution was evaporated to dryness. The solid residue was triturated with water (200 mL), filtered off, and dried in vacuo to obtain **9** as a white solid (27.2 g, 0.156 mol, 76%) [23].

#### 3.1.3. General Procedure for the Synthesis of Cyclic Guanidines **10a**–**d** (Method c)

Primary amines (**8a**–**d**) (10 mmol, 1 eq.) were dissolved in a mixture of methanol (24 mL) and acetic acid (6 mL). To the solutions, 2-(methylthio)-4,5-dihydro-1*H*-imidazole-1-carboxylate (**9**) (2.18 g, 12.5 mmol, 1.25 eq.) was added, and the resulting mixtures were stirred under reflux for 18 h. The solutions were concentrated in vacuo, and the oily residues were dissolved in DCM (100 mL) and were washed with 3 M NaOH solution (2 × 10 mL) and brine (1 × 10 mL). The organic phases were dried over Na_2_SO_4_ and concentrated in vacuo. The products were used without further purification in the subsequent step [23].

#### 3.1.4. Method (d)—General Procedure for the Synthesis of Imipridone Scaffold **11a**–**f** (Method d)

The appropriate *N*-substituted methylcarboxylate piperidone **7a**,**b** (2 mmol, 1 eq.) and cyclic guanidine (**10a**–**d**) (2 mmol, 1 eq.) were dissolved in methanol (10 mL). To this mixture, a 3.6 M methanolic solution of NaOMe was added (0.69 mL, 2.5 mmol, 1.25 eq.) and stirred at reflux temperature for 12 h. In case of reactions with **10a**, the mixture was cooled after 12 h, and the precipitated white crystalline product (**11a**, **11d**) was filtered off, washed twice with cold methanol (2 × 1 mL), and dried in vacuo. In other cases, the mixtures were cooled, concentrated in vacuo, dissolved in DCM (100 mL), washed with water (2 × 10 mL) and brine (10 mL), dried over Na_2_SO_4_, and concentrated in vacuo. The crude yellow oily products were purified by column chromatography on silica using DCM:MeOH:NH_3_ = 15:1:0.1) as the eluent. The collected band was crystallized from Et_2_O to obtain **11b**–**c** and **11e**,**f** as yellowish white powders.

#### 3.1.5. General Procedure for the Synthesis of Azidoimipridones **12a**–**f** (Method e)

The appropriate *N*-Boc protected compound **11a**–**f** (1 mmol, 1 eq.) was dissolved in cc. HCl (5 mL). After stirring for 30 min at 0 °C, using a syringe, aqueous NaNO_2_ solution (2 mmol, 2 eq. in 1.5 mL water) was added dropwise to the solution, which was then stirred for 45 min at 0 °C. To the resulting cold mixture solid NaN_3_ (10 mmol, 10 eq.) was added in portions over 15 min, stirred for further 30 min at 0 °C, then allowed to warm up to room temperature and stirred for an additional 1 h. The acidic mixture was neutralized with Na_2_CO_3_ and extracted with DCM (3 × 40 mL). The combined organic phases were washed with water (2 × 10 mL) and brine (1 × 10 mL), dried over Na_2_SO_4_, and concentrated in vacuo. The products were crystallized from Et_2_O to isolate **12a**–**f** as white solids.

#### 3.1.6. General Procedure for Copper(I) Catalyzed 1,4-Azide-alkyne Cycloadditions (Method f)

The appropriate azidoimipridone (**12a**–**f**) (0.5 mmol, 1 eq.), alkyne component (**13**–**15**, **34a**–**c**) (0.5 mmol, 1 eq.), and CuI (9.5 mg, 0.05 mmol, 0.1 eq.) were dissolved in DMSO (2 mL) and stirred for 12 h at room temperature in a closed vial. After 12 h, the mixture was poured in water (20 mL) and stirred for 20 min. The precipitate was filtered off and washed with water (5 × 10 mL). The filter was washed with DCM (4 × 10 mL). The combined organic filtrates were dried over Na_2_SO_4_ and concentrated in vacuo. The crude product was purified using column chromatography (silica gel, DCM:MeOH:NH_3_ = 15:1:0.1) and crystallized from Et_2_O to yield amber/orange powders (in the case of ferrocene-containing compounds—**16a**, **16d**, **16g**, **16i**, **37a**) or white powders (**16b**–**c**, **16e**–**f**, **16h**, **16j**, **35a**–**c**, **37b**).

#### 3.1.7. General Procedure for the Sonogashira Coupling Reaction (Method g)

The appropriate iodoimipridone (**16a**–**e**, **16g**–**j**, **22a**,**b**, **32a**,**b** or **35a**–**c**) (0.2 mmol, 1 eq.), propargyl derivative (**17a**–**g** or **18**) (0.2 mmol, 1 eq.), CuI (7.6 mg, 0.04 mmol, 0.2 eq.), Pd(PPh_3_)_2_Cl_2_ (14.0 mg, 0.02 mmol, 0.1 eq.), and DIPEA (129 mg, 1 mmol, 5 eq.) were dissolved in DMF (2 mL) and stirred for 24 h. After 24 h, the mixture was poured in water (30 mL) and stirred for 20 min. The precipitate was filtered off and washed with water (5 × 10 mL). The filter was washed with DCM (4 × 20 mL). The combined organic filtrate was dried over Na_2_SO_4_ and concentrated in vacuo. The crude product was purified using column chromatography (silica gel, DCM:MeOH:NH_3_ = 15:1:0.1) and crystallized from Et_2_O to yield amber/orange powders (in the case of ferrocene-containing compounds—**1b**, **2a**, **2c**, **3a**, **3c**, **4a**, **4c**, **23a**, **33a**, **38a**, **39a**, **40a**, **41a**, **42a**) or white powders (**1a**, **1c**, **2b**, **2d**, **3b**, **3d**, **4b**, **4d**, **19**, **23b**, **33b**, **36a**–**c**, **38b**, **39b**, **40b**, **41b**, **42b**, **43a**–**c**).

#### 3.1.8. Synthesis of Acrylamido Imipridone **1e** (Sequential Application of Methods h and i)

Compound **1e** was accessed by sequential acylation and deprotection procedures.

Method h.): Aniline derivative **19** (68.3 mg, 0.1 mmol, 1 eq.) and DIPEA (26 mg, 0.2 mmol, 2 eq.) were dissolved in DCM (10 mL). To this mixture, a solution of acryloyl chloride (12 mg, 0.13 mmol, 1.3 eq.) in DCM (5 mL) was added dropwise at room temperature under N_2_ flow. The resulting solution was stirred for 4 h at room temperature, then concentrated in vacuo.

Method i.): To the residue, concentrated HCl (2 mL) was added, and the solution was stirred for 20 min at room temperature, then diluted with 8 mL of water. The pH was set to 10 with K_2_CO_3_ and the mixture was extracted with DCM (3 × 15 mL). The combined organic phase was washed with water (2 × 5 mL) and brine (5 mL), dried over Na_2_SO_4_, and concentrated in vacuo. The residue was purified by column chromatography on silica using DCM:MeOH:NH_3_ (10:1:0.1) as the eluent. The collected band was evaporated, and the oily residue was crystallized from Et_2_O to obtain **1e** as a white powder.

#### 3.1.9. Deprotection of N-Boc Protected Iodoimipridone **11b** to Produce Iodinated Free Amine **XIb** (Method i)

Compound **11b** (500 mg, 0.82 mmol) and concentrated HCl acid (5 mL) were added to a flask and stirred at room temperature. After 20 min, the solution was diluted with water (45 mL) and the pH was set to 10 with K_2_CO_3_. The mixture was extracted with DCM (3 × 30 mL). The combined organic phase was washed with water (2 × 10 mL) and brine (10 mL), dried over Na_2_SO_4_, and concentrated in vacuo, yielding a white solid (**XIb**) (414 mg, 0.81 mmol, 99%), which was used in the subsequent acylation reactions without further purification.

#### 3.1.10. Synthesis of Amides **22a** and **27a**,**b** (Method j)

Acyl chlorides [ferrocenoyl chloride (**20**) or 4-formylbenzoyl chloride (**25**)] (0.8 mmol, 2 eq.) were dissolved in DCM (10 mL) and a solution of the appropriate amino compound [**XIb**, aminoferrocene (**26a**) or aniline (**26b**)] (0.4 mmol, 1 eq.) and DIPEA (155 mg, 1.2 mmol, 3 eq.) in DCM (10 mL) was added dropwise at 0 °C under N_2_ flow. The mixtures were stirred at 0 °C for 30 min, then allowed to warm up to room temperature and stirred for an additional 1 h. In the course of the synthesis of imipridone compound **22a**, the reaction mixture was washed with saturated Na_2_CO_3_ solution (2 × 5 mL), water (1 × 5 mL), and brine (1 × 5 mL). In the course of the synthesis of compounds **27a**,**b**, the reaction mixtures were subsequently washed with 1M HCl (2 × 5 mL) and saturated Na_2_CO_3_ solution. The combined organic phases were dried over Na_2_SO_4_ and concentrated in vacuo to obtain an orange oil (**22a**) (280 mg), brown-red solid (**27a**) (127 mg, 0.38 mmol, 95%), or white powder (**27b**) (88 mg, 0.39 mmol, 98%). Compounds **27a**,**b** were used in the subsequent reductive alkylation step without further purification, while **22a** was purified by column chromatography on silica gel using DCM:MeOH:NH_3_ = 30:1:0.1 as the eluent to yield an orange solid foam (61 mg, 0.084 mmol, 21%).

#### 3.1.11. Synthesis of Benzamide **22b** (Methods k)

Compound **XIb** (205 mg, 0.4 mmol, 1 eq.), benzoic anhydride (**21**) (181 mg, 0.8 mmol, 2 eq.), and DIPEA (155 mg, 1.2 mmol, 3 eq.) were dissolved in DCM (20 mL) and the solution was stirred under reflux for 24 h, then cooled down and washed with 10% NaOH solution (2 × 5 mL), water (1 × 5 mL), and brine (1 × 5 mL). The organic phase was dried over Na_2_SO_4_, concentrated in vacuo, and crystallized from Et_2_O to obtain **22b** (195 mg, 0.316 mmol, 79%) as a white powder.

#### 3.1.12. Synthesis of Ferrocenoyl Chloride **20** and 4-Formylbenzoyl Chloride **25** (Method l)

The appropriate carboxylic acid [ferrocene carboxylic acid (460 mg, 2 mmol, 1 eq.) or 4-formylbenzoic acid (**24**) (300 mg, 2 mmol, 1 eq.)] was added to DCM (10 mL). To this mixture, oxalyl chloride (1.02 g, 8 mmol, 4 eq.) dissolved in DCM (5 mL) and then 2 drops of DMF were added sequentially at room temperature. The resulting mixture was stirred for 1 h, then concentrated in vacuo, yielding an orange-brown oil (**20**) or a white solid (**25**), which was then triturated with hexane (10 mL). The insoluble impurities were filtered off and the filtrates were concentrated in vacuo to obtain ferrocenoyl chloride (**20**) (484 mg, 1.95 mmol, 97%) as an orange red oil or 4-formylbenzoyl chloride (**25**) (311 mg, 1.84 mmol, 92%) as a white powder.

#### 3.1.13. Synthesis of the Methyl N-Boc-4-oxopiperidine-3-carboxylate (**30**) (Method m)

*N*-Boc-4-oxopiperidin (**28**) (3.0 g, 15 mmol, 1 eq.) and dimethyl-carbonate (**29**) (6.8 g, 75 mmol, 5 eq.) were dissolved in dry toluene (22 mL) under an Ar atmosphere. To this solution, NaH (1.8 g, 75 mmol, 5 eq.) was added in small portions and the resulting mixture was stirred at room temperature for 3 h and refluxed for an additional 2 h under Ar. The mixture was poured over ice (100 g) and the pH was set to 7 with acetic acid. The neutral slurry was extracted with EtOAc (3 × 100 mL). The combined organic phase was dried over Na_2_SO_4_ and concentrated in vacuo to yield **30** as a yellow oil (3.48 g, 13.5 mmol, 90%), which was used as a cyclization partner in the subsequent step without further purification.

#### 3.1.14. Synthesis of 4-(3-Iodobenzyl)-2,4,6,7,8,9-hexahydroimidazo[1,2-a]pyrido[3,4-e]-pyrimidin-5(1H)-one (**31**) (Sequential Application of Methods d and i)

Compound **31** was accessed by the sequential procedures used for the construction of the imipridone scaffold.

Method d): Methyl *N*-Boc-4-oxopiperidine-3-carboxylate (**30**) (180 mg, 0.7 mmol, 1 eq.) and the appropriate cyclic guanidine (**10b**) (211 mg, 0.7 mmol, 1 eq.) were dissolved in methanol (10 mL) and 3.6 M NaOMe solution in methanol (0.24 mL, 0.825 mmol, 1.25 eq.) was added. The mixture was stirred under reflux for 12 h, then cooled, concentrated in vacuo, dissolved in DCM (40 mL), washed with water (2 × 7 mL) and brine (7 mL), dried over Na_2_SO_4_, and concentrated in vacuo. The crude yellow oily product was purified by column chromatography (silica gel, DCM:MeOH:NH_3_ = 20:1:0.1), yielding a light-yellow solid foam (234 mg, 0.46 mmol, 66%).

Method i.): The product of the previous step was dissolved in concentrated HCl (3 mL) and stirred at room temperature. After 20 min, the solution was diluted with water (30 mL) and the pH was set to 10 with K_2_CO_3_. The mixture was extracted with DCM (3 × 20 mL). The combined organic phase was washed with water (2 × 5 mL) and brine (5 mL), dried over Na_2_SO_4_, and concentrated in vacuo, yielding a light-yellow solid foam (**31**) (178 mg, 0.436 mmol, 95% for the deprotection, 62% cumulative yield), which was used without further purification.

#### 3.1.15. Synthesis of Compounds **32a**–**b** by Reductive Alkylation (Method n)

The appropriate aldehyde (**27a**–**b**, 0.25 mmol, 1 eq.) was dissolved in DCM (10 mL). To this solution, **31** dissolved in DCM (10 mL) was added. The mixtures were stirred at room temperature for 20 min. Then, NaHB(OAc)_3_ (74 mg, 0.35 mmol, 1.4 eq.) was added in small portions over the course of 15 min, and the resulting mixtures were stirred for an additional 2.5 h at room temperature. The reaction was monitored by TLC and, upon completion, a saturated solution of K_2_CO_3_ (10 mL) was added and the mixtures were stirred vigorously for 15 min. The organic phases were separated, and the aqueous phases were extracted with DCM (3 × 5 mL). The combined organic phases were washed with water (2 × 5 mL) and brine (1 × 5 mL), dried over Na_2_SO_4_, and concentrated in vacuo. The crude product was purified using column chromatography (silica gel, DCM:MeOH:NH_3_ = 15:1:0.1) and crystallized from Et_2_O to obtain **32a** (105 mg, 0.145 mmol, 58%) as an orange powder and **32b** (121 mg, 0.196 mmol, 78%) as a white powder.

#### 3.1.16. Synthesis of Propargylamine Derivatives **17e**–**g**

The appropriate amine (piperidine, *N*-methylpiperazine or morpholine) (5 mmol, 1 eq.) was dissolved in acetonitrile (20 mL) and K_2_CO_3_ (1.4 g, 10 mmol, 2 eq.), then propargyl bromide (80% in toluene) (820 mg, 5.5 mmol, 1.1 eq.) was added while stirring at room temperature. The resulting suspensions were stirred overnight at room temperature in a closed vial under argon. The mixtures were concentrated in vacuo and triturated with Et_2_O (30 mL). The insoluble salts were filtered off and the filtrates were concentrated in vacuo and purified by column chromatography on silica using DCM:MeOH:NH_3_ (15:1:0.1) as the eluent to obtain **17e** (123 mg, 1 mmol, 20%), **17f** (343 mg, 2.5 mmol, 50%), and **17g** (463 mg, 3.7 mmol, 74%) as orange-yellow oils.

### 3.2. Biological Assays

#### 3.2.1. Cell Culturing

Pharynx squamous cell carcinoma cell line Fadu (HTB-43™), pancreatic ductal adenocarcinoma cell line PANC-1 (CRL-1469™), and engineered human embryonic kidney cell lines HEK293T WT and CLPP^−/−^ were cultured in Dulbecco’s Modified Eagle’s Medium (DMEM, Gibco/Thermo Fisher Scientific, Waltham, MA, USA) supplemented with 10% fetal bovine serum (FBS, Gibco/Thermo Fisher Scientific). Primary fibroblast cultures were prepared from human skin biopsies and maintained in DMEM supplemented with 10% FBS, 1% penicillin-streptomycin, and 1% MEM Non-Essential Amino Acids Solution (Thermo Fisher Scientific). Fadu and PANC-1 cells were obtained from the American Type Culture Collection (ATCC), and the authentication of these cell lines was validated by STR DNA analysis (Eurofins Scientific, Luxembourg, Luxembourg). We received HEK293T WT and HEK293T CLPP^−/−^ cells as a gift from Aleksandra Trifunovic for testing the CLPP dependence of the cytotoxic activities. All cell lines were cultured in a humidified atmosphere at 37 °C and 5% CO_2_, passaged twice a week, and the experiments were performed on cells between passage 5 and 15. The cell lines were routinely screened for the absence of mycoplasma infection (DAPI staining).

#### 3.2.2. CellTiter-Glo Cell Viability Assay, Including the Assessment of ClpP-Coupled Action

The effects of the synthesized compounds on cell viability were measured using the CellTiter-Glo^®^ luminescent cell viability assay (Promega, Madison, WI, USA), according to the manufacturer’s instructions. Briefly, cells were seeded onto a flat-bottomed, white 96-well plate (BRANDplates, cat. no.: 781965)), with the following densities: PANC-1: 750 cells/well, Fadu: 2000 cells/well, primary fibroblasts: 2000 cells/well, HEK293T WT: 1000 cells/well, HEK293T CLPP^−/−^: 2500 cells/well. After seeding, cells were incubated for a further 48 h before treatment. Cells were treated with the compounds for 72 h. Three concentrations (25.0 µM; 8.3 µM; 2.8 µM) were applied during the initial cell viability screening, while for the determination of dose–response curves, 1.5-fold serially diluted compound concentrations were applied (range: 26 µM–0.7 µM). After treatment, the luminescence signal was recorded using a microplate reader (BioTek Synergy 2 Multi-Mode Reader, BioTek, Winooski, VT, USA).

#### 3.2.3. Colony Formation Assay

Long-term cell survival after treatment was determined using a clonogenic assay. PANC-1 cells were seeded in a transparent 6-well cell culture plate (VWR) (density: 1000 cells/well). After seeding, cells were incubated for 72 h before treatment. Cells were treated with the compounds at 4 µM for 24 h or 72 h. Then, the medium was removed, and the cells were washed with phosphate-buffered saline (PBS). The cells formerly treated for 24 or 72 h were further incubated in cell culture medium for 5 or 3 days, respectively. Thereafter, the medium was removed, and the cells were washed with PBS and fixed with 4% paraformaldehyde for 10 min. After fixation, the cells were washed with PBS, and 0.5% crystal violet (CV) solution was added. After 1 h, the CV solution was removed, and the cells were washed thoroughly with water. Images were created using Corel Photo-Paint 2019.

### 3.3. Statistics

Data analysis was performed using GraphPad Prism 9.5.0 software (GraphPad Software, San Diego, CA, USA). During the preliminary screening (three-point assays), only mean and standard deviation (SD) values were calculated and reported from the results of duplicate measurements. The dose–response curves were generated by four-parameter nonlinear regression, and the IC_50_ values were determined as the means ± SEM of the best-fit values of 3 independent measurements. The R^2^ values were consistently above 0.93, indicating a strong fit of the model to the experimental data (Supplementary S.89). Comparative statistical analyses were performed using one-way ANOVA to assess differences between the novel imipridone hybrids and the reference compound ONC201. Dunnett’s post hoc test was applied to adjust for multiple comparisons, minimizing the likelihood of false positive results. Assumptions for ANOVA, including normality (assessed via QQ plots) and homogeneity of variance (tested using the Brown–Forsythe test), were verified. The following significance levels were applied: * *p* < 0.05, ** *p* < 0.01, *** *p* < 0.001, **** *p* < 0.0001. Each cell line was analyzed separately to compare the effects of the novel imipridone hybrids against the reference compound ONC201. The analysis ensured compliance with the assumptions of ANOVA, including normality and homogeneity of variance, and the results are detailed in the Supplementary Materials S.90. The tumor selectivity of the compounds was quantitatively characterized by the selectivity index (SI). This parameter was calculated as the ratio of the average best-fit IC_50_ value for primary fibroblasts and the best-fit IC_50_ value for the corresponding tumor cells (PANC-1 or Fadu).

## 4. Conclusions

Based on the results of high-throughput screening, which served as a guideline for the iterative structural modifications, we successfully synthesized a library of systematically designed novel imipridone hybrids with optimally positioned alkyne- and triazole-linked warheads. The tumor cytotoxic potential of these compounds was evaluated against PANC-1 and Fadu cells. Our preliminary mechanistic studies identified **38a**,**b** as the most potent derivatives, which exerted their cytotoxicity in a ClpP-independent manner. Their outstanding activity appeared to result from their cooperative interactions with as yet unidentified cellular target(s) owing to the ferrocenyl/phenyltriazolyl- and *N*-methylaminopropargyl groups optimally positioned on the central imipridone core. This distinctive mechanism of action sets them apart from conventional ClpP agonist imipridone-based drugs.

The new drug candidates were comparable to **ONC201** in terms of efficient dose but outperformed this emblematic first-in-class imipridone in overcoming treatment resistance. They completely eradicated cancer cells in our experiments at concentrations around 4 μM. However, their limited tumor selectivity and narrow therapeutic window pose challenges that need to be addressed during ongoing development. Despite the promising results presented in this study, there is undeniably a need for further refinement in both molecular design and therapeutic application. Future studies will prioritize the identification of the cellular molecular target(s) of these drug candidates to elucidate their exact mechanism(s) of action. This will enable the rational design of additional derivatives with enhanced specificity and reduced toxicity toward non-cancer cells.

Widening the therapeutic window remains a critical goal in optimizing these compounds for clinical use. Advanced drug delivery systems, such as nanoparticles, liposomes, and antibody-drug conjugates (ADCs), offer viable solutions for enhancing tumor selectivity by leveraging tumor-specific properties like the enhanced permeability and retention (EPR) effect [30]. Furthermore, localized delivery strategies could reduce systemic toxicity while increasing the drug concentration directly at the tumor site [31]. Combination therapies that utilize synergistic interactions between agents may allow for effective tumor suppression at reduced doses, thereby mitigating off-target toxicity [32]. Modulating the tumor microenvironment, for example by normalizing the vasculature to improve drug perfusion, is another promising way to optimize efficacy [33]. Our results indicate the significant anticancer potential of the hybrids under development, particularly for aggressive and treatment-resistant tumors. Their ability to completely eradicate cancer cells reflects a level of efficacy that could offer very significant advances in the treatment of hard-to-treat malignancies. By coupling these compounds with targeted delivery systems or tumor-specific ligands, the therapeutic window can be effectively widened, providing reduced systemic toxicity without compromising anticancer potency [30,31,32,33].

Oncology is moving toward precision medicine, and the ongoing development of these hybrids perfectly fits this trend, as they can address unmet clinical needs by integrating rational drug design and advanced drug delivery technology. With their distinct mechanism of action and demonstrated potency, these compounds hold promise for addressing some of the most pressing challenges in modern oncology. Their development represents an exciting advance in imipridone-based therapeutics and highlights the importance of innovation in overcoming the limitations of conventional cancer treatments.

## Figures and Tables

**Table 1:** Results of the initial viability screening of the first set of novel imipridone derivatives on PANC-1 and Fadu cancer cell lines. Standard deviation (SD) values illustrate the small variation between duplicates in this preliminary screening (n = 2).

Comp.	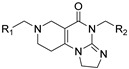		Cell Viability (% of Control)			
	R_1_	R_2_	Cell Line	25 µM	8.3 µM	2.8 µM
**ONC201**	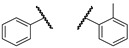		**PANC-1**	53 ± 2	58 ± 1	69 ± 1
			**Fadu**	27 ± 1	29 ± 1	31 ± 1
**I/A**	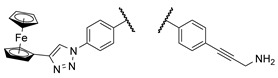		**PANC-1**	0 ± 0	39 ± 7	108 ± 4
			**Fadu**	0 ± 0	66 ± 24	105 ± 9
**I/B**	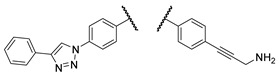		**PANC-1**	0 ± 0	61 ± 22	113 ± 4
			**Fadu**	0 ± 0	103 ± 6	112 ± 7
**16f**	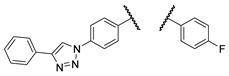		**PANC-1**	60 ± 1	68 ± 4	81 ± 3
			**Fadu**	77 ± 4	94 ± 1	100 ± 1
**1a**	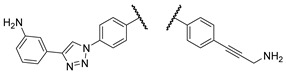		**PANC-1**	3 ± 2	93 ± 7	100 ± 5
			**Fadu**	10 ± 0	99 ± 2	106 ± 2
**1b**	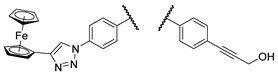		**PANC-1**	95 ± 2	93 ± 3	92 ± 0
			**Fadu**	68 ± 3	83 ± 0	88 ± 4
**1c**	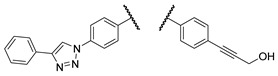		**PANC-1**	88 ± 6	87 ± 2	95 ± 3
			**Fadu**	64 ± 2	71 ± 0	79 ± 0
**1d**	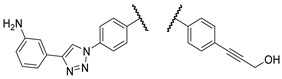		**PANC-1**	100 ± 5	101 ± 3	99 ± 3
			**Fadu**	84 ± 4	96 ± 7	99 ± 9
**1e**	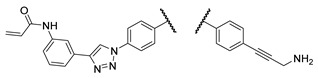		**PANC-1**	102 ± 0	91 ± 1	101 ± 7
			**Fadu**	69 ± 2	59 ± 4	96 ± 6
**2a**	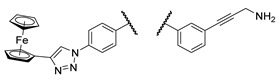		**PANC-1**	0 ± 0	0 ± 0	92 ± 2
			**Fadu**	0 ± 0	3 ± 0	107 ± 5
**2b**	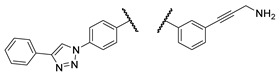		**PANC-1**	0 ± 0	0 ± 0	107 ± 1
			**Fadu**	0 ± 0	0 ± 0	102 ± 8
**2c**	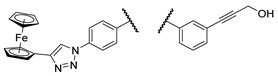		**PANC-1**	97 ± 2	95 ± 2	94 ± 2
			**Fadu**	65 ± 0	76 ± 4	79 ± 2
**2d**	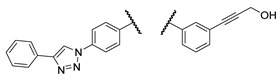		**PANC-1**	81 ± 0	87 ± 1	100 ± 8
			**Fadu**	78 ± 6	90 ± 10	99 ± 7
**3a**	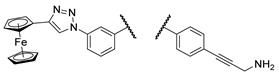		**PANC-1**	0 ± 0	88 ± 0	101 ± 7
			**Fadu**	0 ± 0	100 ± 6	104 ± 5
**3b**	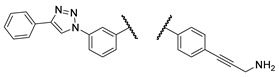		**PANC-1**	0 ± 0	84 ± 2	103 ± 8
			**Fadu**	22 ± 4	95 ± 3	103 ± 2
**3c**	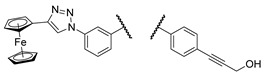		**PANC-1**	98 ± 1	98 ± 1	100 ± 3
			**Fadu**	85 ± 5	99 ± 2	103 ± 9
**3d**	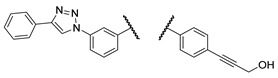		**PANC-1**	46 ± 7	55 ± 6	85 ± 1
			**Fadu**	23 ± 3	29 ± 2	87 ± 1
**4a**	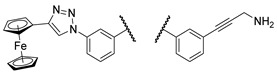		**PANC-1**	0 ± 0	73 ± 9	91 ± 4
			**Fadu**	0 ± 0	77 ± 3	102 ± 1
**4b**	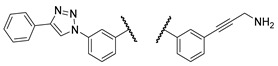		**PANC-1**	0 ± 0	70 ± 0	87 ± 5
			**Fadu**	0 ± 0	34 ± 3	76 ± 1
**4c_c_**	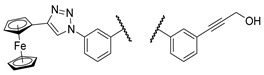		**PANC-1**	47 ± 5	51 ± 5	67 ± 2
			**Fadu**	54 ± 4	68 ± 7	75 ± 1
**4d**	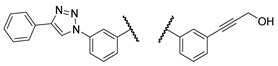		**PANC-1**	53 ± 8	60 ± 2	74 ± 3
			**Fadu**	23 ± 3	27 ± 3	37 ± 1

**Table 2:** Results of the initial viability screening of the second set of novel imipridone derivatives on PANC-1 and Fadu cancer cell lines. Standard deviation (SD) values illustrate the small variation between duplicates in this preliminary screening (n = 2).

Comp.	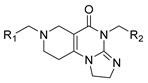		Cell Viability (% of Control)			
No.	R_1_	R_2_	Cell Line	25 µM	8.3 µM	2.8 µM
**ONC** **201** **^a^**	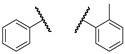		**PANC-1**	22 ± 2	35 ± 2	59 ± 9
			**Fadu**	25 ± 3	31 ± 2	38 ± 5
**I/A** **^a^**	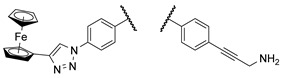		**PANC-1**	0 ± 0	39 ± 7	108 ± 4
			**Fadu**	0 ± 0	66 ± 24	105 ± 9
**I/B** **^a^**	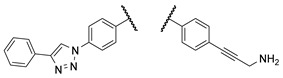		**PANC-1**	0 ± 0	61 ± 22	113 ± 4
			**Fadu**	0 ± 0	103 ± 6	112 ± 7
**2a** **^a^**	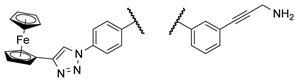		**PANC-1**	0 ± 0	92 ± 14	117 ± 4
			**Fadu**	0 ± 0	56 ± 9	114 ± 8
**2b** **^a^**	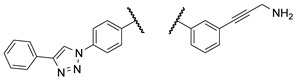		**PANC-1**	0 ± 0	0 ± 0	113 ± 3
			**Fadu**	0 ± 0	27 ± 12	117 ± 7
**23a**	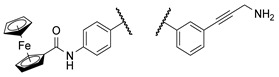		**PANC-1**	52 ± 55	104 ± 10	98 ± 7
			**Fadu**	61 ± 20	107 ± 7	109 ± 10
**23b**	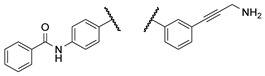		**PANC-1**	47 ± 39	106 ± 5	113 ± 15
			**Fadu**	77 ± 6	108 ± 7	117 ± 11
**33a**	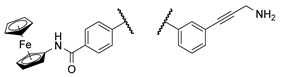		**PANC-1**	4 ± 5	107 ± 9	102 ± 9
			**Fadu**	9 ± 8	93 ± 2	99 ± 2
**33b**	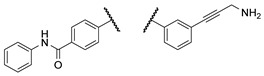		**PANC-1**	71 ± 32	109 ± 5	102 ± 9
			**Fadu**	80 ± 11	115 ± 6	120 ± 9
**36a**	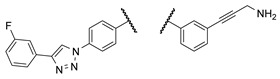		**PANC-1**	0 ± 0	0 ± 0	116 ± 8
			**Fadu**	0 ± 0	6 ± 5	105 ± 20
**36b**	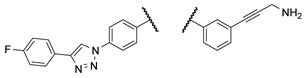		**PANC-1**	0 ± 0	0 ± 0	116 ± 7
			**Fadu**	0 ± 0	1 ± 1	114 ± 7
**36c**	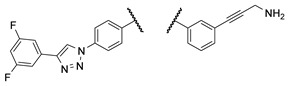		**PANC-1**	0 ± 0	0 ± 0	111 ± 8
			**Fadu**	0 ± 0	5 ± 5	111 ± 10
**37a**	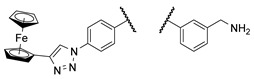		**PANC-1**	16 ± 8	123 ± 5	118 ± 4
			**Fadu**	0 ± 0	70 ± 20	106 ± 10
**37b**	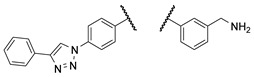		**PANC-1**	5 ± 6	113 ± 3	110 ± 5
			**Fadu**	13 ± 6	91 ± 12	108 ± 7
**38a**	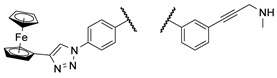		**PANC-1**	0 ± 0	0 ± 0	44 ± 41
			**Fadu**	0 ± 0	0 ± 1	85 ± 16
**38b**	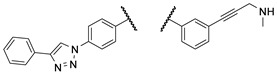		**PANC-1**	0 ± 0	0 ± 0	6 ± 6
			**Fadu**	0 ± 0	0 ± 0	91 ± 17
**39a**	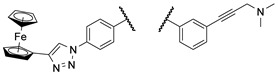		**PANC-1**	35 ± 41	62 ± 72	112 ± 7
			**Fadu**	13 ± 15	45 ± 52	99 ± 9
**39b**	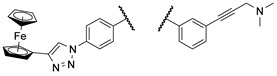		**PANC-1**	2 ± 3	25 ± 20	107 ± 3
			**Fadu**	1 ± 1	15 ± 8	96 ± 4
**40a**	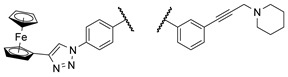		**PANC-1**	61 ± 67	72 ± 61	119 ± 4
			**Fadu**	45 ± 34	81 ± 21	105 ± 5
**40b**	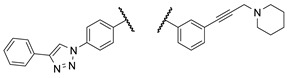		**PANC-1**	1 ± 1	12 ± 10	125 ± 5
			**Fadu**	24 ± 11	60 ± 3	92 ± 9
**41a**_c_	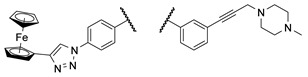		**PANC-1**	0 ± 0	58 ± 67	112 ± 7
			**Fadu**	0 ± 0	41 ± 42	97 ± 4
**41b**	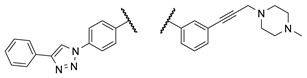		**PANC-1**	0 ± 0	0 ± 0	110 ± 6
			**Fadu**	3 ± 1	30 ± 28	106 ± 1
**42a**	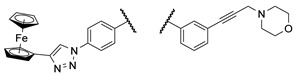		**PANC-1**	58 ± 36	75 ± 31	132 ± 4
			**Fadu**	55 ± 12	112 ± 11	121 ± 3
**42b**	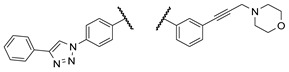		**PANC-1**	59 ± 3	82 ± 5	94 ± 5
			**Fadu**	13 ± 6	91 ± 12	108 ± 7

^a^ For a more reliable comparison and to circumvent the batch effect, **ONC201**, **I/A**, **I/B**, **2a**, and **2b**, with the latter two emerging as the most potent compounds during the screening of the first set, were repeatedly screened as references along with the samples of the second set of hybrid molecules.

**Table 3:** IC_50_ values (µM) of the selected imipridone derivatives and reference compounds in PANC-1 and Fadu cell lines and primary fibroblasts. Data obtained by the CellTiter-Glo cell viability assay after 72 h treatment were analyzed using GraphPad Prism 9.5.0 software (four-parameter nonlinear regression; variable slope; best-fit values; n = 3; n. d.: not determined).

	IC_50_ Value ± SEM			Selectivity Index (SI) ^a^	
	PANC-1	Fadu	Primary Fibroblast	Fibroblast/PANC-1	Fibroblast/Fadu
**ONC201**	3.1 ± 0.26	1.3 ± 0.15	n. d.	n. d.	n. d.
**I/A**	10.7 ± 0.31	10.0 ± 0.23	14.9 ± 0.46	1.4	1.5
**I/B**	8.2 ± 0.41	10.3 ± 0.17	11.0 ± 0.65	1.3	1.1
**38a**	2.7 ± 0.07	2.9 ± 0.06	4.6 ± 0.83	1.7	1.6
**38b**	2.5 ± 0.23	3.3 ± 0.07	4.4 ± 0.23	1.8	1.3
**43a**	2.5 ± 0.94	3.2 ± 0.10	3.2 ± 0.07	1.3	1.0
**43b**	3.1 ± 0.17	3.2 ± 0.06	3.5 ± 0.17	1.1	1.1
**43c**	2.6 ± 0.03	3.6 ± 0.09	3.7 ± 1.73	1.4	1.0

^a^ The selectivity index (SI), as a quantitative indicator of tumor selectivity, was calculated as the ratio of the mean best-fit IC_50_ for primary fibroblasts and the best-fit IC_50_ for cancer cells.

**Table 4:** IC_50_ values (µM) of **38a**,**b** and **ONC201** in HEK293T WT and CLPP^−/−^ cells measured by the CellTiter-Glo cell viability assay after 72 h treatment. IC_50_ values ± SEM were determined by GraphPad Prism 9.5.0 software (nonlinear regression; variable slope; best-fit values; n = 4).

	IC_50_ (µM)	
	HEK293T WT	HEK293T CLPP^−/−^
**ONC201**	0.6 ± 0.03	n. d. ^a^
**38a**	1.6 ± 0.04	2.4 ± 0.03
**38b**	1.7 ± 0.02	2.1 ± 0.03

^a^ Since the cell viability did not decreased down to 50% in the studied range of drug concentration, no data can be provided as IC_50_ value.

## Data Availability

The data generated and analyzed during our research are not available in any public database or repository but will be shared by the corresponding author upon reasonable request.

## Supplementary Materials

The following supporting information can be downloaded at: https://www.mdpi.com/article/10.3390/ijms252313176/s1.
