# Supplementary material for: Development of Novel Imipridones with Alkyne- and Triazole-Linked Warheads on the Tricyclic Skeleton, Showing Superior Ability to Eradicate PANC-1 and Fadu Cells Compared to ONC201
Source: Int J Mol Sci. 2024 Dec 7;25(23):13176. doi: 10.3390/ijms252313176 (PMC11642016; doi:10.3390/ijms252313176)
Supplement: Supplementary file 1 [file ijms-25-13176-s001.zip › ijms-3298738-supplementary.pdf]

Supplementary Materials

Development of Novel Imipridones with Alkyne- and Triazole-linked Warheads on the Tricyclic Skeleton, Showing Superior Ability to Eradicate PANC-1 and Fadu Cells Compared to ONC201

Tamás Czuczi [1,2], József Murányi [1], István Móra [3], Bianka Gurbi [3], Attila Varga [3], Dávid Papp [2,4], Gitta Schlosser [4], Miklós Csala [3,*] and Antal Csámpai [1,*]

1 Department of Organic Chemistry, Eötvös Loránd University (ELTE), Pázmány P. sétány 1/A, H-1117 Budapest, Hungary; czuczi.tamas@gmail.com (T.C.).
2 Hevesy György PhD School of Chemistry, Pázmány P. sétány 1/A, H-1117 Budapest, Hungary
3 Department of Molecular Biology, Semmelweis University, Tűzoltó u. 37-47, H-1094 Budapest, Hungary; istvan.mora1313@gmail.com (I.M.); gurbi.bianka@semmelweis.hu (B.G.); varga.attila3@semmelweis.hu (A.V.); csala.miklos@semmelweis.hu (M.C.)
4 MTA-ELTE Lendület Ion Mobility Mass Spectrometry Research Group, ELTE Eötvös Loránd University, Institute of Chemistry, Pázmány Péter sétány 1/A, H-1117 Budapest, Hungary; david.papp@ttk.elte.hu (D.P.); gitta.schlosser@ttk.elte.hu (G.S.)

* Correspondence: antal.csampai@ttk.elte.hu (A.C.); Tel.: +36-01-372-2500 (ext. 6591) and csala.miklos@semmelweis.hu (M.C.); Tel.: +36-01-266-2615

Content:

S.1. ^{1}H-, ^{13}C NMR and HRMS data of the targeted compounds pp. 2 – 13

S.2. Copies of the ^{1}H-NMR and ^{13}C-NMR spectra of the targeted compounds pp. 14 – 50

S.3. Copies of the HRMS spectra the targeted compounds pp. 51 – 87

S.4. **HPLC chromatograms of selected compounds pp. 88 – 89**

S.5. **CellTiter-Glo Cell Viability Assay Data pp. 90 – 92**

S.6. **Comparison of IC_{50} Values for Novel Imipridone Hybrids Across Cell Lines Using One-Way ANOVA Analysis Relative to ONC201. pp. 93 – 95**

S.1. ^{1}H-, ^{13}C NMR and HRMS data of the targeted compounds

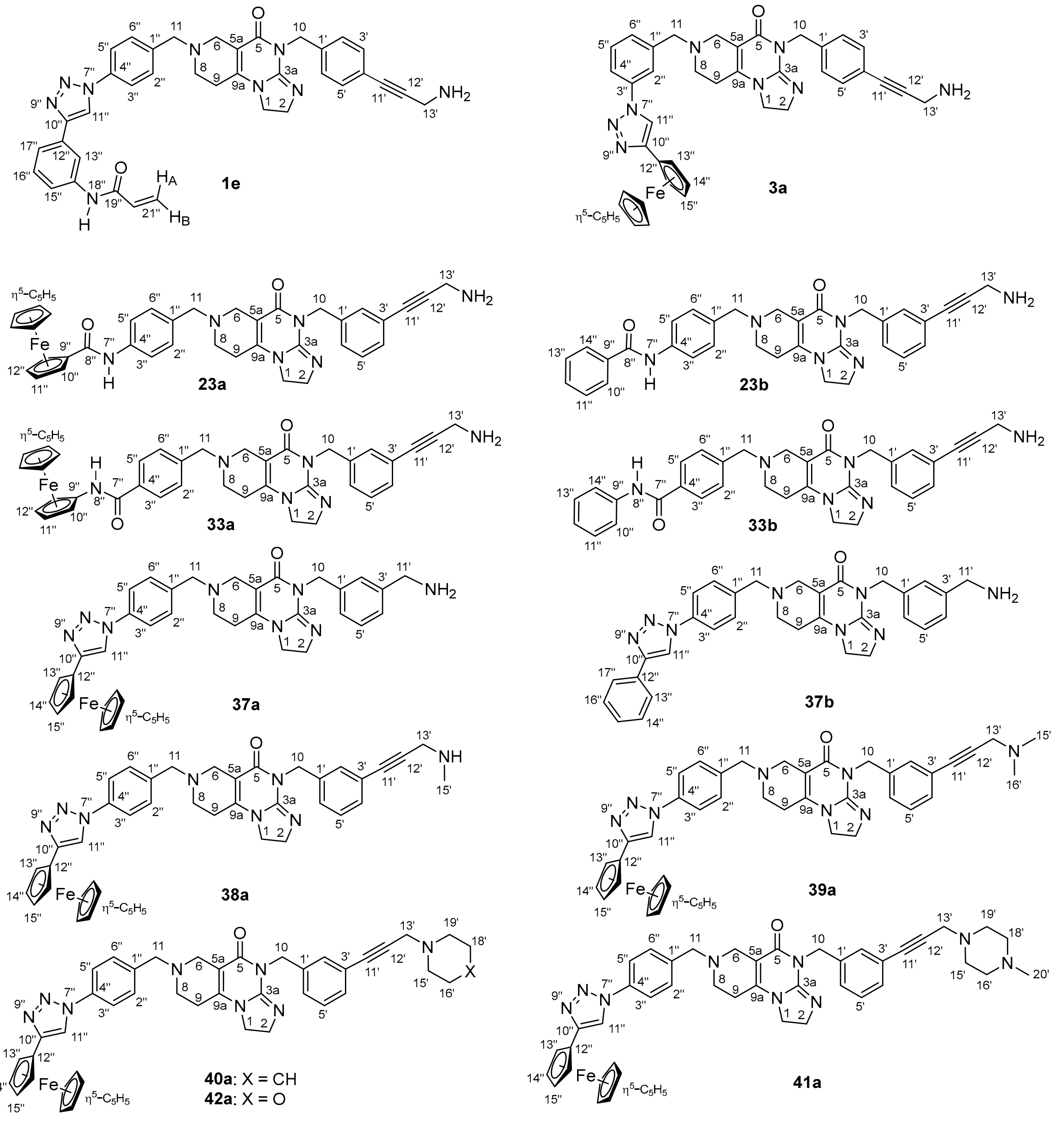

Figure S.1. Numbering of atoms presented on the structures of representative compounds used for the assignment of all ^{1}H- and ^{13}C-NMR data.

7-(4-(4-(3-Aminophenyl)-1H-1,2,3-triazol-1-yl)benzyl)-4-(4-(3-aminoprop-1-yn-1-yl)benzyl)-2,4,6,7,8,9-hexahydroimidazo[1,2-a]pyrido[3,4-e]pyrimidin-5(1H)-one (1a): ^{1}H-NMR ($CDCl_3$): 8.14 (s, 1H, H-11″); 7.74 (d, *J* = 7.9 Hz, 2H, H-3″,5″); 7.52 (d, *J* = 7.9 Hz, 2H, H-2″,6″); 7.40 (d, *J* = 7.4 Hz, 2H, H-2′,6′); 7.35 and 7.34 (overlapping br s and d, *J* =7.4 Hz for the d, 3H, H-13″ and H-3′,5′); 7-27-7.21 (overlapping m′s, 2H, H-16″,17″); 6.71 (dt, *J* = 6.9 Hz and 2.3 Hz, 1H, H-15″); 5.04 (s, 2H, H-10); 3.91 (br s, 4H, H-1 and H-2); 3.72 (s, 2H, H-11); 3.64 (s, 2H, H-13′); 3.32 (br s, 2H, H-6); 2.71 (t, *J* = 5.7 Hz, 2H, H-8); 2.50 (t, *J* = 5.7 Hz, 2H, H-9). ^{13}C-NMR ($CDCl_3$): 161.4 (C-5); 152.9 (C-3a); 148.5 =C-10″); 147.0 (two coalesced lines, C-9a and C-14″); 138.8 (C-1″); 136.9 (C-1′); 136.2 (C-4″); 131.5 (C-3′,5′); 131.2 (C-12″), 130.3 (C-2″,6″); 129.2 (C-16″); 128.5 (C-2′,6′); 122.3 (C-4′); 120.6 (C-3″,5″); 117.7 (C-11″); 116.1 (C-17″); 115.2 (C-15″); 112.5 (C-13″); 101.7 (C-5a); 90.1 (C-12′),82.4 (C-11′); 61.6

(C-11); 50.6 (C-1); 49.4 (C-6); 48.4 (C-8), 32.2 (C-13′); 26.8 (C-9). HRMS exact mass calcd. for $[C_{34}H_{33}N_9O]^+$: 584.2881 $[M+H]^+$; found: 584.28697; mass error: –1.93 ppm.

4-(4-(3-Hydroxyprop-1-yn-1-yl)benzyl)-7-(4-(4-ferrocenyl-1H-1,2,3-triazol-1-yl)benzyl)-2,4,6,7,8,9-hexahydroimidazo[1,2-a]pyrido[3,4-e]pyrimidin-5(1H)-one (1b): ^{1}H-NMR ($CDCl_3$): 7.90 (s, 1H, H-11″); 7.74 (d, *J* = 8.3 Hz, 2H, H-3″,5″); 7.51 (d, *J* = 8.3 Hz, 2H, H-2″,5″); 7.40 (d, *J* = 8.4 Hz, 2H, H-2′,6′); 7.35 (d, *J* = 8.4 Hz, 2H, H-3′,5′); 5.05 (s, 2H, H-10); 4.80 (t, *J* = 1.8 Hz, 2H, H-13″16″); 4.47 (s, 2H, H-13′); 4.36 (t, *J* = 1.8 Hz, 2H, H-14″15″); 4.13 (s, 5H, η^5-$C_5\underline{H}_5$); 3.91 (br s, 4H, H-1 and H-2); 3.74 (s, 2H, H-11); 3.31 (br s, 2H, 6-H); 2.71 (t, *J* = 5.7 Hz, 2H, H-8); 2.50 (t, *J* = 5.7 Hz, 2H, H-9). ^{13}C-NMR ($CDCl_3$): 161.4 (C-5); 153.0 (C-3a); 147.6 (C-10″); 145.7 (C-9a); 138.5 (C-1″); 137.3 (C-1′); 136.3 (C-4″); 131.6 (C-3′,5′); 130.2 (C-2″,6″); 128.5 (C-2′,6′); 121.6 (C-4′); 120.4 (C-3″,5″); 116.6 (C-11′); 101.8 (C-5a); 87.4 (C-12′); 85.5 (C-11′); 74.9 (C-12″); 69.6 (η^5-$\underline{C}_5H_5$); 68.8 (C-14″,15″); 66.8 (C-13″,16″); 61.6 (C-11); 51.5 (C-13′); 50.5 (C-1); 49.4 (C-6); 48.4 (C-8); 46.9 (C-2); 45.2 (C-10); 26.7 (C-9).). HRMS exact mass calcd. for $[C_{38}H_{35}FeN_7O_2]$: 678.2274 $[M+H]^+$; found: 678.22904; mass error: 2.42 ppm.

4-(4-(3-Hydroxyprop-1-yn-1-yl)benzyl)-7-(4-(4-phenyl-1H-1,2,3-triazol-1-yl)benzyl)-2,4,6,7,8,9-hexahydroimidazo[1,2-a]pyrido[3,4-e]pyrimidin-5(1H)-one (1c): ^{1}H-NMR ($CDCl_3$): 8.20 (s, 1H, H-11″); 7.94 (br d, *J* ‘ 8 Hz, 2H, H-13″,17″); 7.76 (d, *J* = 8.4 Hz, 2H, H-3″,5″); 7.53 (d, *J* = 8.4 Hz, 2H, H-2″,6″); 7.48 (t, *J* = 7.5 Hz, 2H, H-14″,16″); 7.39 and 7.48 (overlapping d and tt, *J* = 8.5 Hz for d and 7.5 Hz and 1.5 Hz, for tt, 3H, H-2′,6′ and H-15″); 7.33 (d, *J* = 8.5 Hz, 2H, H-3′,5′); 5.04 (s, 2H, H-10); 4.46 (br s, 2H, H-13′); 3.90 (s, 4H, H-1 and H-2); 3.74 (s, 2H-11); 3.31 (br s, 2H, H-6), 2.71 (t, *J* = 5.7 Hz, 2H, H-8); 2.50 (t, *J* = 5.7 Hz, 2H, H-9); 2.12 (br s, OH). ^{13}C-NMR ($CDCl_3$): 161.4 (C-5); 153.0 (C-3a); 148.4 (C-10″); 145.1 (C-9a); 138.8 (C-1″); 137.3 (C-1′); 136.2 (C-4″); 131.6 (C-3′,5′); 130.3 (C-2″,6″); 130.2 (C-12″); 128.9 (C-14″,16″); 128.49 (C-2′,6′); 128.45 (C-15″); 125.9 (C-13″,17″); 121.7 (C-4′); 120.6 (C-3″,5″); 117.7 (C-11′); 101.8 (C-5a); 87.5 (C-12′); 85.5 (C-11′); 61.6 (C-11′); 51.5 (C-13′); 50.5 (C-1); 49.4 (C-6); 48.4 (C-8); 46.9 (C-2); 45.2 (C-10); 26.8 (C-9). HRMS exact mass calcd. for $[C_{34}H_{31}N_7O_2]^+$: 570.2612 $[M+H]^+$; found: 570.2630; mass error: 3.16 ppm.

7-(4-(4-(3-Aminophenyl)-1H-1,2,3-triazol-1-yl)benzyl)-4-(4-(3-hydroxyprop-1-yn-1-yl)benzyl)-2,4,6,7,8,9-hexahydroimidazo[1,2-a]pyrido[3,4-e]pyrimidin-5(1H)-one (1d): ^{1}H-NMR (DMSO-*d*$_6$): 8.12 (s, 1H, H-11″); 7.92 (d, *J* = 8.3 Hz, 2H, H-3″,5″); 7.56 (d, *J* = 8.3 Hz, 2H, H-2″,6″); 7.35 (d, *J* = 8.1 Hz, 2H, H-3′,5′); 7.28 (d, *J* = 8.1 Hz, 2H, H-2′,6′); 7.22 (t, *J* = 1.7 Hz, 1H, H-13″); 7.12 (t, *J* = 8.0 Hz, 1H, H-16″); 7.04 (br d, *J* ~ 8 Hz, 1H, H-17″); 6.58 (dd, *J* = 8.0 Hz and 1.7 Hz, 1H, H-15″); 5.32 (t, *J* = 5.8 Hz, 1H, OH); 5.23 (br s, 2H, NH_2); 4.90 (s, 2H, H-10); 4.29 (d, *J* = 5.8 Hz, 2H, H-13′); 3.94 (t, *J* = 9.1 Hz, 2H, H-1); 3.71 (overlapping t and s, *J* = 9.1 for t, 4H, H-2 and H-11); 3.07 (br s, 2H, H-6), 2.68 (t, *J* = 5.7 Hz, 2H, H-8); 2.55 (t, *J* = 5.7 Hz, 2H, H-9). ^{13}C-NMR (DMSO-*d*$_6$): 161.1 (C-5); 152.5 (C-3a); 147.8 (C-9a); 149.6 (C-14″); 148.6 (C-10″); 139.4 (C-1″); 138.2 (C-1′); 136.0 (C-4″); 131.6 (C-3′,5′); 131.2 (C-12″); 130.6 (C-2″,6″); 129.9 (C-16″); 128.4 (C-2′,6′); 121.6 (C-4′); 120.4 (C-3″,5″); 119.6 (C-11″); 114.4 (C-15″); 113.7 (C-17″); 111.1 (C-13″); 99.7 (C-5a); 90.2 (C-12′); 84.0 (C-11′); 61.0 (C-11); 50.6 (C-1); 49.9 (C-13′); 49.2 (C-6); 48.8 (C-8); 46.8 (C-2); 26.3 (C-9). HRMS exact mass calcd. for $[C_{34}H_{32}N_8O_2]$: 585.2721 $[M+H]^+$; found: 585.27152; mass error: –0.99 ppm.

N-(3-(1-(4-((4-(4-(3-Aminoprop-1-yn-1-yl)benzyl)-5-oxo-1,2,4,5,8,9-hexahydroimidazo[1,2-a]pyrido[3,4-e]pyrimidin-7(6H)-yl)methyl)phenyl)-1H-1,2,3-triazol-4-yl)phenyl)acrylamide (1e): ^{1}H-NMR (DMSO-*d*$_6$): 10.32 (s, H-18″); 9.26 (s, 1H, H-11″); 8.34 (br s, 1H, H-13″); 7.94 (d, *J* = 8.3 Hz, 2H, H-3″,5″); 7.71 (br d, J = 8.0 Hz, H-15″); 7.61 (br d, *J* = 8.0 Hz, 1H, H-17″); 7.57 (d, *J* = 8.3 Hz, 2H, H-2″,6″); 7.45 (t, *J* = 8.0 Hz, 1H, H-16″); 7.32 (d, *J* = 7.8 Hz, 2H, H-3′,5′); 7.26 (d, *J* = 7.8 Hz, 2H, H-2′,6′); 6.49 (dd, *J* = 17.0 Hz and 10.2 Hz, 1H, H-20″); 6.31 (dd, *J* = 17.0 Hz and 1.5 Hz, 1H, H-21″$_A$); 5.79 (dd, *J* = 10.2 Hz and 1.5 Hz, 1H, H-21″$_B$); 4.90 (s, 2H, H-10); 3.96 (t, *J* = 9.2 Hz, 2H, H-1); 3.72 (overlapping t and s, *J* = 9.2 for t, 4H, H-2 and H-11); 3.49 (s, 2H, H-13′); 3.08 (br s, 2H, H-6); 2.69 (t, *J* = 5.6 Hz, 2H; H-8); 2.56 (t, *J* = 5.6 Hz, 2H; H-9). ^{13}C-NMR (DMSO-*d*$_6$): 163.7 (C-19″); 161.1 (C-5);

152.5 (C-3a); 147.8 (C-9a); 147.6 (C-10″); 140.1 (C-14″); 139.6 (C-1″); 137.8 (C-1′); 136.1 (C-4″); 132.3 (C-21″); 131.5 (C-3′,5′); 131.3 (C-12″); 130.5 (C-2″,6″); 128.3 (C-2′,6′); 127.5 (two coalesced lines, C-16″ and C-20″); 122.1 (C-4′); 121.2 (C-17″); 120.5 (C-3″,5″); 120.2 (C-11″); 119.7 (C-15″); 116.6 (C-13″); 99.6 (C-5a); 92.5 (C-12′); 81.6 (C-11′); 61.0 (C-11); 50.6 (C-1); 49.2 (C-6); 48.8 (C-8); 46.8 (C-2); 31.8 (C-13′); 44.8 (C-10); 26.3 (C-9). HRMS exact mass calcd. for [$C_{37}H_{35}N_9O_2$]: 638.2986 [M+H]$^+$; found: 638,29871; mass error: 0.17 ppm.

4-(3-(3-aminoprop-1-yn-1-yl)benzyl)-7-(4-(4-ferrocenyl-1H-1,2,3-triazol-1-yl)benzyl)-2,4,6,7,8,9-hexahydroimidazo[1,2-a]pyrido[3,4-e]pyrimidin-5(1H)-one (2a): ^{1}H-NMR (DMSO-*d*$_6$):8.88 (s, 1H, H-11″); 7.91 (d, *J* = 8.3 Hz, 2H, H-3″,5″); 7.56 (d, *J* = 8.3 Hz, 2H, H-2″,5″); 7.33-7.24 (overlapping m′s, 4H, H-2′, H-4′, H5′ and H-6′); 4.89 (s, 2H, H-10); 4.80 (br s, 2H, H-13″16″); 4.36 (br s, 2H, H-14″15″); 4.09 (s, 5H, η^5-C$_5$<u>H</u>$_5$); 3.95 (t, *J* = 9.5 Hz, 2H, H-1); 3.72 and 3.71 (overlapping s and t, *J* = 9.5 Hz for t, 4H, H-11 and H-2); 3.50 (s, 2H, H-13′); 3.09 (br s, 2H, 6-H); 2.68 (t, *J* = 5.7 Hz, 2H, H-8); 2.55 (t, *J* = 5.7 Hz, 2H, H-9). ^{13}C-NMR (DMSO-*d*$_6$): 161.2 (C-5); 152.5 (C-3a); 147.8 (C-9a); 147.0 (C-10″); 139.2 (C-1″); 138.2 (C-1′); 136.1 (C-4″); 130.8 (C-2′); 130.5 (C-2″,6″); 130.4 (C-4′); 129.0 (C-5′); 128.3 (C-6′); 123.2 (C-3′); 120.2 (C-3″,5″); 118.9 (C-11′); 99.6 (C-5a); 92.7 (C-12′); 81.7 (C-11′); 76.8 (C-12″); 69.8 (η^5-<u>C</u>$_5$H$_5$); 68.9 (C-14″,15″); 66.8 (C-13″,16″); 61.0 (C-11); 50.6 (C-1); 49.2 (C-6); 48.8 (C-8); 46.8 (C-2); 44.7 (C-10); 31.8 (C-13′), 26.2 (C-9). HRMS exact mass calcd. for HRMS exact mass calcd. for [$C_{38}H_{36}FeN_8O$]$^+$: 677.2434 [M+H]$^+$; found: 677.24278; mass error: –0.92 ppm.

4-(3-(3-Aminoprop-1-yn-1-yl)benzyl)-7-(4-(4-phenyl-1H-1,2,3-triazol-1-yl)benzyl)-2,4,6,7,8,9-hexahydroimidazo[1,2-a]pyrido[3,4-e]pyrimidin-5(1H)-one (2b): ^{1}H-NMR (DMSO-*d*$_6$): 9.28 (s, 1H, H-11″); 7.92 (d, *J* = 8.3 Hz, 2H, H-3″,5″); 7.96 (d, *J* = 7.4 Hz, 2H, H-13″,17″); 7.57 (d, *J* = 8.3 Hz, 2H, H-2″,5″); 7.50 (t, *J* = 7.4 Hz, 2H, H-14″,16″); 7.39 (tt, *J* = 7.4 Hz and 1.8 Hz, 1H, H-15″); 7.32-7.24 (overlapping m′s, 3H, H-4′, H5′ and H-6′); 4.88 (s, 2H, H-10); 3.95 (t, *J* = 9.5 Hz, 2H, H-1); 3.72 and 3.71 (overlapping s and t, *J* = 9.5 Hz for t, 4H, H-11 and H-2); 3.49 (s, 2H, H-13′); 3.09 (br s, 2H, 6-H); 2.68 (t, *J* = 5.7 Hz, 2H, H-8); 2.54 (t, *J* = 5.7 Hz, 2H, H-9). ^{13}C-NMR (DMSO-*d*$_6$): 161.2 (C-5); 152.5 (C-3a); 147.76 (C-9a); 147.72 (C-10″); 139.5 (C-1″); 138.2 (C-1′); 136.1 (C-4″); 130.8 (two coalesced lines, C-2′ and C-14″,16″); 130.6 (C-2″,6″); 130.4 (coalesced lines, C-4′ and C-12″); 129.0 (C-5′); 128.7 (C-15″); 128.3 (C-6′); 125.8 (C-13″,17″); 123.1 (C-3′); 120.4 (C-3″,5″); 120.1 (C-11′); 99.7 (C-5a); 92.7 (C-12′); 81.7 (C-11′); 60.9 (C-11); 50.6 (C-1); 49.2 (C-6); 48.8 (C-8); 46.8 (C-2); 44.8 (C-10); 31.8 (C-13′), 26.2 (C-9). HRMS exact mass calcd. for [$C_{34}H_{32}N_8O$]$^+$: 569.2772 [M+H]$^+$; found 569,27763; mass error: –0.76 ppm.

4-(3-(3-Hydroxyprop-1-yn-1-yl)benzyl)-7-(4-(4-ferrocenyl-1H-1,2,3-triazol-1-yl)benzyl)-2,4,6,7,8,9-hexahydroimidazo[1,2-a]pyrido[3,4-e]pyrimidin-5(1H)-one (2c):): ^{1}H-NMR (CDCl$_3$): 7.90 (s, 1H, H-11″); 7.74 (d, *J* = 8.3 Hz, 2H, H-3″,5″); 7.51 (d, *J* = 8.3 Hz, 2H, H-2″,5″); 7.50 (br s, 1H, H-2′); 7.43 (br d, *J* = 7.6 Hz, 1H, H-6′); 7.31 (br d, *J* = 7.6 Hz, 1H, H-4′); 7.24 (t, *J* = 7.6 Hz, 1H, H-5′); 5.04 (s, 2H, H-10); 4.81 (t, *J* = 1.8 Hz, 2H, H-13″16″); 4.47 (s, 2H, H-13′); 4.36 (t, *J* = 1.8 Hz, 2H, H-14″15″); 4.13 (s, 5H, η^5-C$_5$<u>H</u>$_5$); 3.92 (br s, 4H, H-1 and H-2); 3.74 (s, 2H, H-11); 3.31 (br s, 2H, 6-H); 2.71 (t, *J* = 5.7 Hz, 2H, H-8); 2.50 (t, *J* = 5.7 Hz, 2H, H-9). ^{13}C-NMR (CDCl$_3$): 161.4 (C-5); 152.9 (C-3a); 147.6 (C-10″); 145.7 (C-9a); 138.5 (C-1″); 137.1 (C-1′); 136.3 (C-4″); 131.4 (C-2′); 130.7 (C-4′); 130.3 (C-2″,6″); 128.8 (C-6′); 128.3 (C-5′); 122.7 (C-3′); 120.4 (C-3″,5″); 116.6 (C-11′); 101.7 (C-5a); 87.4 (C-12′); 85.6 (C-11′); 74.9 (C-12″); 69.6 (η^5-<u>C</u>$_5$H$_5$); 68.8 (C-14″,15″); 66.8 (C-13″,16″); 61.6 (C-11); 51.5 (C-13′); 50.5 (C-1); 49.4 (C-6); 48.5 (C-8); 46.9 (C-2); 45.0 (C-10); 26.7 (C-9). HRMS exact mass calcd. for [$C_{38}H_{35}FeN_7O_2$]$^+$: 678.2274 [M+H]$^+$; found: 678.22748; mass error: 0.12 ppm.

4-(3-(3-Hydroxyprop-1-yn-1-yl)benzyl)-7-(4-(4-phenyl-1H-1,2,3-triazol-1-yl)benzyl)-2,4,6,7,8,9-hexahydroimidazo[1,2-a]pyrido[3,4-e]pyrimidin-5(1H)-one (2d): ^{1}H-NMR (CDCl$_3$): 8.21 (s, 1H, H-11″); 7.92 (d, *J* = 7.8 Hz, 2H, H-13″,17″); 7.75 (d, *J* = 8.3 Hz, 2H, H-3″,5″); 7.52 (d, *J* = 8.3 Hz, 2H, H-2″,5″); 7.50 (br s, 1H, H-2′); 7.48 (t, *J* = 7.8 Hz, 2H, H-14″,16″); 7.42 (br d, *J* = 7.6 Hz, 1H, H-6′); 7.38 (t, *J* = 7.8 Hz, 1H, H-15″); 7.30 (br d, *J* = 7.6 Hz, 1H, H-4′); 7.23 (t, *J* = 7.6 Hz, 1H, H-5′); 5.03 (s, 2H, H-10); 4.47 (s, 2H, H-13′); 3.89 (br s, 4H, H-1

and H-2); 3.72 (s, 2H, H-11); 3.31 (br s, 2H, 6-H); 2.70 (t, *J* = 5.7 Hz, 2H, H-8); 2.49 (t, *J* = 5.7 Hz, 2H, H-9). ^{13}C-NMR ($CDCl_3$): 161.4 (C-5); 152.9 (C-3a); 148.5 (C-10″); 145.8 (C-9a); 138.8 (C-1″); 137.0 (C-1′); 136.3 (C-4″); 131.3 (C-2′); 130.6 (C-4′); 130.3 (C-2″,6″); 130.2 (C-12″); 129.0 (C-14″,16″); 128.8 (two coalesced lines, C-6′ and C-15″); 128.3 (C-5′); 122.6 (C-3′); 120.6 (C-3″,5″); 117.8 (C-11′); 101.7 (C-5a); 87.6 (C-12′); 85.4 (C-11′); 61.5 (C-11); 51.4 (C-13′); 50.5 (C-1); 49.4 (C-6); 48.4 (C-8); 46.9 (C-2); 45.0 (C-10); 26.7 (C-9). HRMS exact mass calcd. for $[C_{34}H_{31}N_7O_2]^+$: 570.2612 [M+H]$^+$; found: 570.2630; mass error: 3.16 ppm.

4-(4-(3-Aminoprop-1-yn-1-yl)benzyl)-7-(3-(4-ferrocenyl-1H-1,2,3-triazol-1-yl)benzyl)-2,4,6,7,8,9-hexahydroimidazo[1,2-a]pyrido[3,4-e]pyrimidin-5(1H)-one (3a): ^{1}H-NMR ($CDCl_3$): 7.92 (s, 1H, H-11″); 7.80 (br s, 1H, H-2″); 7.70 (br d, *J* = 7.8 Hz, 1H, H-4″); 7.49 (t, *J* = 7.8 Hz, 1H, H-5″); 7.42-7.38 (overlapping m′s, 3H, H-2′,6′ and H-6″); 7.33 (d, *J* = 8.3 Hz, 2H, H-3′,5′); 5.04 (s, 2H, H-10); 4.80 (br s, 2H, H-13″,16″); 4.35 (br s, 2H, H-14″,15″); 4.13 (s, 5H, η^5-$C_5\underline{H}_5$); 3.91 (br s, 4H, H-1 and H-2); 3.77 (s, 2H, H-11); 3.65 (s, 2H, H-13′); 3.33 (br s, 2H, 6-H); 2.74 (t, *J* = 5.6 Hz, 2H, H-8); 2.56 (t, *J* = 5.6 Hz, 2H, H-9). ^{13}C-NMR ($CDCl_3$): 161.4 (C-5); 152.9 (C-3a); 147.6 (C-10″); 145.7 (C-9a); 140.2 (C-1″); 137.3 (C-3″); 134.8 (C-1′); 131.5 (C-3′,5′); 129.8 (C-5″); 129.1 (C-6″); 128.5 (C-2′,6′); 122.4 (C-4′); 120.6 (C-2″); 119.4 (C-4″); 116.8 (C-11′); 101.7 (C-5a); 84.7 (C-12′); 82.5 (C-11′); 74.9 (C-12″); 69.6 (η^5-$\underline{C}_5H_5$); 68.8 (C-14″,15″); 66.8 (C-13″,16″); 61.8 (C-11); 50.6 (C-1); 49.5 (C-6); 48.5 (C-8); 46.9 (C-2); 45.2 (C-10); 32.2 (C-13′); 26.8 (C-9). HRMS exact mass calcd. for $[C_{38}H_{36}FeN_8O]^+$: 677.2434 [M+H]$^+$; found: 677,24557; mass error: 3.20 ppm.

4-(4-(3-Aminoprop-1-yn-1-yl)benzyl)-7-(3-(4-phenyl-1H-1,2,3-triazol-1-yl)benzyl)-2,4,6,7,8,9-hexahydroimidazo[1,2-a]pyrido[3,4-e]pyrimidin-5(1H)-one (3b): ^{1}H-NMR (DMSO-d_6): 9.32 (s, 1H, H-11″); 7.98 (br d, *J* = 7.9 Hz, 2H, H-13″,17″); 7.95 (br s, 1H, H-2″);7.86 (br d, *J* = 7.7 Hz, 1H, H-4″); 7. 60 (t, *J* = 7.7 Hz, 1H, H-5″); 7.50 (t, , *J* = 7.9 Hz, 2H, H-14″,16″); 7.47 (br d, *J* = 7.7 Hz, 1H, H-6″); 7.39 (t, *J* = 7.9 Hz, 1H, H-15″); 7.32 (d, *J* = 8.0 Hz, 2H, H-3′,5′); 7.26 (d, *J* = 8.0 Hz, 2H, H-2′,6′); 4.89 (s, 2H, H-10); 3.75 (s, 2H, H-11); 3.94 (t, *J* = 9.7 Hz, 2H, H-1); 3.71 (t, *J* = 9.7 Hz, 2H, H-2); 3.49 (s, 2H, H-13′); 3.11 (br s, 2H, H-6); 2.69 (t, *J* = 5.6 Hz, 2H, H-8); 2.56 (t, *J* = 5.6 Hz, 2H, H-9). ^{13}C-NMR (DMSO-d_6): 161.2 (C-5); 152.5 (C-3a); 147.8 (C-10″); 147.6 (C-9a); 141.2 (C-1″); 137.8 (C-1′); 137.3 (C-2″); 131.5 (C-3′,5′); 130.7 (C-3″); 130.3 (C-5″); 129.5 (C-14″,16″); 129.4 (C-6″); 128.7 (C-15″); 128.3 (2′,6′); 125.8 (C-13″,17″); 122.0 (C-4′); 120.4 (C-2″); 120.1 (C-11″); 119.2 (C-4″); 99.6 (C-5a); 92.5 (C-12′); 81.8 (C-11′); ″); 61.2 (C-11); 50.5 (C-1); 49.3 (C-6); 48.8 (C-8); 46.8 (C-2); 44.8 (C-10); 31.9 (C-13′); 26.2 (C-9). HRMS exact mass calcd. for $[C_{34}H_{32}N_8O]^+$: 569.2772. [M+H]$^+$; found: 569.27695; mass error: –0.44 ppm.

4-(4-(3-Hydroxyprop-1-yn-1-yl)benzyl)-7-(3-(4-ferrocenyl-1H-1,2,3-triazol-1-yl)benzyl)-2,4,6,7,8,9-hexahydroimidazo[1,2-a]pyrido[3,4-e]pyrimidin-5(1H)-one (3c): ^{1}H-NMR ($CDCl_3$): 7.92 (s, 1H, H-11″); 7.80 (br s, 1H, H-2″); 7.70 (br d, *J* = 7.8 Hz, 1H, H-4″); 7.49 (t, *J* = 7.8 Hz, 1H, H-5″); 7.42-7.38 (overlapping m′s, 3H, H-2′,6′ and H-6″); 7.33 (d, *J* = 8.3 Hz, 2H, H-3′,5′); 5.04 (s, 2H, H-10); 4.80 (t, *J* = 1.8 Hz, 2H, H-13″,16″); 4.46 (s, 2H, H-13′); 4.35 (t, *J* = 1.8 Hz, 2H, H-14″,15″); 4.13 (s, 5H, η^5-$C_5\underline{H}_5$); 3.89 (br s, 4H, H-1 and H-2); 3.76 (s, 2H, H-11); 3.32 (br s, 2H, 6-H); 2.72 (t, *J* = 5.6 Hz, 2H, H-8); 2.50 (t, *J* = 5.6 Hz, 2H, H-9). ^{13}C-NMR ($CDCl_3$): 161.4 (C-5); 152.9 (C-3a); 147.6 (C-10″); 145.7 (C-9a); 140.2 (C-1″); 137.3 (C-3″); 137.2 (C-1′); 131.6 (C-3′,5′); 129.8 (C-5″); 129.1 (C-6″); 128.5 (C-2′,6′); 121.9 (C-4′); 120.6 (C-2″); 119.3 (C-4″); 116.7 (C-11′); 101.7 (C-5a); 87.5 (C-12′); 85.4 (C-11′); 74.9 (C-12″); 69.6 (η^5-$\underline{C}_5H_5$); 68.8 (C-14″,15″); 66.8 (C-13″,16″); 61.8 (C-11); 51.5 (C-13′); 50.5 (C-1); 49.4 (C-6); 48.5 (C-8); 46.9 (C-2); 45.2 (C-10); 26.7 (C-9). HRMS exact mass calcd. for $[C_{38}H_{35}FeN_7O_2]^+$: 678.2274 [M+H]$^+$; found: 678.22769; mass error: 0.43 ppm.

4-(4-(3-Hydroxyprop-1-yn-1-yl)benzyl)-7-(3-(4-phenyl-1H-1,2,3-triazol-1-yl)benzyl)-2,4,6,7,8,9-hexahydroimidazo[1,2-a]pyrido[3,4-e]pyrimidin-5(1H)-one (3d): ^{1}H-NMR (DMSO-d_6): 9.31 (s, 1H, H-11″); 7.97 (dd, *J* = 7.4 Hz and 1.4 Hz, 2H, H-13″,17″); 7.95 (br s, 1H, H-2″); 7.86 (br d, *J* = 7.8 Hz, 1H, H-4″); 7.60 (t, *J* = 7.8 Hz, 1H, H-5″); 7.50 (t, *J* = 7.4 Hz, 2H, H-14″,16″); 7.47 (br d, *J* = 8.3 Hz, 1H, H-6″);7.39 (tt, *J* = 7.4 Hz and 1.4 Hz,

1H, H-15″); 7.35 (d, *J* = 8.3 Hz, 2H, H-3′,5′); 7.27 (d, *J* = 8.3 Hz, 2H, H-2′,6′); 5.31 (t, *J* = 5.5 Hz, 1H, OH); 4.90 (s, 2H, H-10); 4.29 (d, *J* = 5.5 Hz, 2H, H-13′); 3.95 (t, *J* = 9.7 Hz, 2H, H-1); 3.76 (s, 2H, H-11); 3.71 (t, *J* = 9.7 Hz, 2H, H-2); 3.12 (br s, 2H, 6-H); 2.70 (t, *J* = 5.7 Hz, 2H, H-8); 2.56 (t, *J* = 5.7 Hz, 2H, H-9). ^{13}C-NMR (DMSO-d_6): 161.2 (C-5); 152.5 (C-3a); 147.77 (C-10″); 147.79 (C-9a); 141.2 (C-1″); 138.2 (C-1′); 137,2 (C-3″); 131.6 (C-3′,5′); 130.7 (C-12″); 130.3 (C-5″); 129.5 (C-14″,16″); 129.4 (C-6″); 128.7 (C-15″); 128.3 (C-2′,6′); 125.8 (C-13″,17″); 121.6 (C-4′); 120.4 (C-2″); 120.1 (C-11′); 119.2 (-4″); 99.6 (C-5a); 90.3 (C-12′); 84.0 (C-11′); 61.2 (C-11); 50.5 (C-1); 49.9 (C-13′), 49.3 (C-6); 48.8 (C-8); 46.8 (C-2); 44.8 (C-10); 26.2 (C-9). HRMS exact mass calcd. for [$C_{34}H_{31}N_7O_2$]$^+$: 570.2612. [M+H]$^+$; found: 570,25985 mass error: –2.37 ppm.

4-(3-(3-Aminoprop-1-yn-1-yl)benzyl)-7-(3-(4-ferrocenyl-1H-1,2,3-triazol-1-yl)benzyl)-2,4,6,7,8,9-hexahydroimidazo[1,2-a]pyrido[3,4-e]pyrimidin-5(1H)-one (4a): ^{1}H-NMR (CDCl$_3$): 7.92 (s, 1H, H-11″); 7.80 (br s, 1H, H-2″); 7.70 (br d, *J* = 7.6 Hz, 1H, H-4″); 7.50 and 7.49 (overlapping br s and t, *J* = 7.6 Hz for the t, 2H, H-2′ and H-5″); 7.42-7.38 (overlapping m′s, 2H, H-6′ and H-6″); 7.28 (br d, *J* = 7.6 Hz, 1H, H-4′); 7.22 (t, *J* = 7.6 Hz, 1H, H-5′); 5.03 (s, 2H, H-10); 4.80 (br s, 2H, H-13″,16″); 4.35 (br s, 2H, H-14″,15″); 4.13 (s, 5H, η^5-$C_5\underline{H}_5$); 3.91 (br s, 4H, H-1 and H-2); 3.77 (s, 2H, H-11); 3.64 (s, 2H, H-13′); 3.33 (br s, 2H, 6-H); 2.73 (t, *J* = 5.6 Hz, 2H, H-8); 2.52 (t, *J* = 5.6 Hz, 2H, H-9). ^{13}C-NMR (CDCl$_3$): 161.4 (C-5); 152.9 (C-3a); 147.6 (C-10″); 145.7 (C-9a); 140.2 (C-1″); 137.3 (C-3″); 137.0 (C-1′); 130.6 (C-5″); 130.3 (C-2′); 129.2 (C-4′); 129.1 (C-6″); 128.5 (C-6′); 128.3 (C-5′); 123.2 (C-3′); 120.6 (C-2″); 119.5 (C-4″); 116.7 (C-11′); 101.6 (C-5a); 84.7 (C-12′); 82.7 (C-11′); 74.9 (C-12″); 69.6 (η^5-$\underline{C}_5H_5$); 68.8 (C-14″,15″); 66.8 (C-13″,16″); 61.8 (C-11); 50.6 (C-1); 49.5 (C-6); 48.5 (C-8); 46.9 (C-2); 45.1 (C-10); 32.2 (C-13′); 26.8 (C-9). HRMS exact mass calcd. for [$C_{38}H_{36}FeN_8O$]$^+$: 677.2434 [M+H]$^+$; found: 677.24278; mass error: –0.92 ppm.

4-(3-(3-Aminoprop-1-yn-1-yl)benzyl)-7-(3-(4-phenyl-1H-1,2,3-triazol-1-yl)benzyl)-2,4,6,7,8,9-hexahydroimidazo[1,2-a]pyrido[3,4-e]pyrimidin-5(1H)-one (4b): ^{1}H-NMR (DMSO-d_6): 9.32 (s, 1H, H-11″); 7.95 (br d, *J* = 7.5 Hz, 2H, H-13″,17″); 7.90 (br s, 1H, H-2″); 7.87 (br d, *J* = 7.6 Hz, 1H, H-4″); 7.60 (t, *J* = 7.6 Hz, 1H, H-5″); 7.50 (t, *J* = 7.5 Hz, 2H, H-14″,16″); 7.47 (br d, *J* = 7.6 Hz, 1H, H-6″); 7.39 (t, *J* = 7.5 Hz, 1H, H-15″); 7.31-7.24 (overlapping m′s, 4H, H-2′, H4′, H5′ and H-6′); 4.89 (s, 2H, H-10); 3.95 (t, *J* = 9.7 Hz, 2H, H-1); 3.76 (s, 2H, H-11); 3.72 (t, *J* = 9.7 Hz, 2H, H-2); 3.49 (s, 2H, H-13′); 3.13 (br s, 2H, 6-H); 2.70 (t, *J* = 5.7 Hz, 2H, H-8); 2.56 (t, *J* = 5.7 Hz, 2H, H-9). ^{13}C-NMR (DMSO-d_6): 161.2 (C-5); 152.5 (C-3a); 147.81 (C-10″); 147.78 (C-9a); 141.2 (C-1″); 138.2 (C-1′); 137.2 (C-3″); 130.8 (C-12″); 130.4 (C-5′); 130.3 (C-2′); 129.5 (C-14″,16″); 129.4 (C-6″); 129.0 (C-4′); 128.7 (two coalesced lines, C-5′ and C-15″); 128.2 (C-6′); 125.8 (C-13″,17″); 123.2 (C-3′); 120.4 (C-2″); 120.1 (C-11′); 119.2 (C-4″); 99.6 (C-5a); 92.6 (C-12′); 81.7 (C-11′); 61.2 (C-11); 50.6 (C-1); 49.3 (C-6); 48.7 (C-8); 46.8 (C-2); 44.7 (C-10); 31.7 (C-13′); 26.2 (C-9). HRMS exact mass calcd. for [$C_{34}H_{32}N_8O$]$^+$: 569.2772 [M+H]$^+$; found: 569.27853; mass error: 2.34 ppm.

4-(3-(3-Hydroxyprop-1-yn-1-yl)benzyl)-7-(3-(4-ferrocenyl-1H-1,2,3-triazol-1-yl)benzyl)-2,4,6,7,8,9-hexahydroimidazo[1,2-a]pyrido[3,4-e]pyrimidin-5(1H)-one (4c): ^{1}H-NMR (DMSO-d_6): 8.92 (s, 1H, H-11″); 7.92 (br s, 1H, H-2″); 7.85 (br d, *J* = 7.6 Hz, 1H, H-4″); 7.58 (t, *J* = 7.6 Hz, 1H, H-5″); 7.45 (br d, *J* = 7.6 Hz, 1H, H-6″); 7.34-7.26 (overlapping m′s, 4H, H-2′, H4′, H5′ and H-6′); 5.34 (t, *J* = 5.5 Hz, 1H, OH); 4.89 (s, 2H, H-10); 4.81 (br s, 2H, H-13″,16″); 4.37 (br s, 2H, H-14″,15″); 4.29 (d, *J* = 5.5 Hz, 2H, H-13′); 4.09 (s, 5H, η^5-$C_5\underline{H}_5$); 3.96 (t, *J* = 9.8 Hz, 2H, H-1); 3.76 (s, 2H, H-11); 3.72 (t, *J* = 9.8 Hz, 2H, H-2); 3.13 (br s, 2H, 6-H); 2.70 (t, *J* = 5.6 Hz, 2H, H-8); 2.56 (t, *J* = 5.6 Hz, 2H, H-9). ^{13}C-NMR (DMSO-d_6): 161.2 (C-5); 152.5 (C-3a); 145.8 (C-9a); 147.2 (C-10″); 141.2 (C-1″); 138.3 (C-1′); 137.3 (C-3″); 130.8 (C-2′); 130.4 (C-5″); 130.3 (C-4′); 129.15 (C-6″); 129.09 (C-5′); 128.6 (C-6′); 122.7 (C-3′); 120.2 (C-2″); 118.92 (C-4″); 118.87 (C-11′); 99.6 (C-5a); 90.4 (C-12′); 84.0 (C-11′); 75.8 (C-12″); 69.8 (η^5-$\underline{C}_5H_5$); 69.0 (C-14″,15″); 66.9 (C-13″,16″); 61.2 (C-11); 50.6 (C-1); 49.9 (C-13′); 49.3 (C-6); 48.8 (C-8); 46.8 (C-2); 44.6 (C-10); 26.2 (C-9). HRMS exact mass calcd. for [$C_{38}H_{35}FeN_7O_2$]$^+$: 678.2274 [M+H]$^+$; found: 678.22748; mass error: 0.12 ppm.

4-(3-(3-Hydroxyprop-1-yn-1-yl)benzyl)-7-(3-(4-phenyl-1H-1,2,3-triazol-1-yl)benzyl)-2,4,6,7,8,9-hexahydroimidazo[1,2-a]pyrido[3,4-e]pyrimidin-5(1H)-one (4d): ^{1}H-NMR (DMSO-d_6): 9.32 (s, 1H, H-11″); 7.97 (br d, *J* = 7.5 Hz, 2H, H-13″,17″); 7.95 (br s, 1H, H-2″); 7.87 (br d, *J* = 7.6 Hz, 1H, H-4″); 7.60 (t, *J* = 7.6 Hz, 1H, H-5″); 7.50 (t, *J* = 7.5 Hz, 2H, H-14″,16″); 7.47 (br d, *J* = 7.6 Hz, 1H, H-6″); 7.39 (t, *J* = 7.5 Hz, 1H, H-15″); 7.33-7.27 (overlapping m′s, 4H, H-2′, H4′, H5′ and H-6′); 5.33 (t, *J* = 5.5 Hz, 1H, OH); 4.89 (s, 2H, H-10); 4.29 (d, *J* = 5.5 Hz, 2H, H-13′); 3.95 (t, *J* = 9.7 Hz, 2H, H-1); 3.76 (s, 2H, H-11); 3.72 (t, *J* = 9.7 Hz, 2H, H-2); 3.13 (br s, 2H, 6-H); 2.69 (t, *J* = 5.7 Hz, 2H, H-8); 2.55 (t, *J* = 5.7 Hz, 2H, H-9). ^{13}C-NMR (DMSO-d_6): 161.2 (C-5); 152.5 (C-3a); 147.82 (C-10″); 147.79 (C-9a); 141.2 (C-1″); 138.3 (C-1′); 137.2 (C-3″); 130.8 (C-2′); 130.7 (C-12″); 130.4 (C-5″); 130.3 (C-4′); 129.5 (C-14″,16″); 129.4 (C-6″); 129.1 (C-5′); 128.7 (C-15″); 128.6 (C-6′); 125.8 (C-13″,17″); 122.7 (C-3′); 120.4 (C-2″); 120.1 (C-11′); 119.2 (C-4″); 99.6 (C-5a); 90.4 (C-12′); 84.0 (C-11′); 61.2 (C-11); 50.6 (C-1); 49.8 (C-13′); 49.3 (C-6); 48.7 (C-8); 46.8 (C-2); 44.7 (C-10); 26.2 (C-9). HRMS exact mass calcd. for $[C_{34}H_{31}N_7O_2]^+$: 570.2612 $[M+H]^+$; found: 570.26085; mass error: –0.61 ppm.

N-(4-((4-(3-(3-Aminoprop-1-yn-1-yl)benzyl)-5-oxo-1,2,4,5,8,9-hexahydroimidazo[1,2-a]pyrido[3,4-e]pyrimidin-7(6H)-yl)methyl)phenyl)ferrocenecarboxamide (23a): ^{1}H-NMR (DMSO-d_6): 9.41 (s, 1H, H-5″); 7.68 (d, *J* = 8.3 Hz, 2H, H-3″,5″); 7.23-7.27 (overlapping m′s, 6H, H-2′, H-4′, H-5′, H-6′ and H-2″,6″); 5.00 (br s, 2H, H-8″,11″); 4.88 (s, 2H, H-10); 4.45 (br s, 2H, H-9″,10″); 4.22 (s, 5H, η^5-$C_5\underline{H}_5$); 3.95 (t, *J* = 9.1 Hz, 2H, H-1); 3.72 (t, *J* = 9.1 Hz, 2H, H-2); 3.59 (s, 2H, H-11); 3.52 (br, s, 2H, H-13″); 3.05 (br s, 2H, H-6); 2.64 (t, *J* = 5.6 Hz, 2H, H-8); 2.54 (t, *J* = 5.6 Hz, 2H, H-9). ^{13}C-NMR (DMSO-d_6): 168.5 (C-6″); 161.2 (C-5); 152.5 (C-3a); 147.8 (C-9a); 138. 7 (C-4″); 138.2 (C-1′); 137.3 (C-2″); 133.3 (two coalesced lines, C-4′ and C-1″); 130.8 (C-2′); 130.3 (C-5′); 129.5 (C-2″,6″); 128.3 (C-6′); 123.2 (C-3′); 120.7 (C-3″,5″); 99.8 (C-5a); 92.3 (C-12′), 81.8 (C-11′); 77.0 (C-7″); 70.9 (C-8″,9″); 69.9 (η^5-$\underline{C}_5H_5$); 69.1 (C-8″,10″); 61.4 (C-11); 50.6 (C-1); 49.1 (C-6), 48.7 (C-8); 46.8 (C-2); 44.7 (C-10); 31.7 (C-13″); 26.3 (C-9). HRMS exact mass calcd. for $[C_{37}H_{36}FeN_6O_2]^+$: 653.2322. $[M+H]^+$; found: 653,23324; mass error: 1.59 ppm.

N-(4-((4-(3-(3-Aminoprop-1-yn-1-yl)benzyl)-5-oxo-1,2,4,5,8,9-hexahydroimidazo[1,2-a]pyrido[3,4-e]pyrimidin-7(6H)-yl)methyl)phenyl)benzamide (23b): ^{1}H-NMR (DMSO-d_6): 10.25 (s, 1H, H-7″); 7.96 (d, *J* = 7.6 Hz, 2H, H-10″,14″); 7.76 (d, *J* = 8.2 Hz, 2H, H-3″,5″); 7.59 (t, *J* = 7.6 Hz, 1H, H-12″); 7.53 (t, *J* = 7.6 Hz, 2H, H-11″,13″); 7.33-7.24 (overlapping m′s, 6H, H-2′, H-4′, H-5′, H-6′and H-2″,6″); 4.88 (s, 2H, H-10); 3.94 (t, *J* = 9.7 Hz, 2H, H-1); 3.71 (t, *J* = 9.7 Hz, 2H, H-2); 3.59 (s, 2H, H-11); 3.51 (s, 2H, H-13′); 3.04 (br s, 2H, H-6); 2.63 (t, *J* = 5.7 Hz, 2H, H-8); 2.53 (t, *J* = 5.7 Hz, 2H, H-9); ^{13}C-NMR (DMSO-d_6): 165.9 (C-8″); 161.2 (C-5); 152.5 (C-3a); 147.8 (C-9a); 138.6 (C-4″); 138.3 (C-1′); 135.5 (C-9″); 133.9 (C-1″); 132-0 (C-12″); 130.8 (C-2′); 130.3 (C-4′); 129.6 (C-2″,6″); 129.0 (C-5′); 128.8 (C-11″,13″); 128.3 (C-6′); 128.1 (C-10″,14″); 123.2 (C-3′); 120.7 (C-3″,5″); 99.8 (C-5a); 92.3 (C-12′); 81.9 (C-11″); 61.3 (C-11); 50.6 (C-1); 49.1 (C-6), 48.7 (C-8); 46.8 (C-9); 44.7 (C-10); 31.7 (C-13′); 26.3 (C-9). HRMS exact mass calcd. for $[C_{33}H_{32}N_6O_2]^+$: 545,2660. $[M+H]^+$; found: 545,26641; mass error: 0.75 ppm.

4-((4-(3-(3-Aminoprop-1-yn-1-yl)benzyl)-5-oxo-1,2,4,5,8,9-hexahydroimidazo[1,2-a]pyrido[3,4-e]pyrimidin-7(6H)-yl)methyl)-N-ferrocenylnylbenzamide (33a): ^{1}H-NMR (DMSO-d_6): 9.70 (s, 1H, H-8″); 7.87 (d, *J* = 7.9 Hz, 2H, H-3″,5″); 7.45 (d, *J* = 7.9 Hz, 2H, H-2″,6″); 7.34-7.24 (overlapping m′s, 4H, H-2′, H-4′ H-5′ and H-6′); 4.88 (s, 2H, H-10); 4.80 (br s, 2H, H-10″,13″); 4.13 (s, 5H, η^5-$C_5\underline{H}_5$); 4.01 (br s, 2H, H-11″,12″); 3.96 (t, *J* = 9.6 Hz, 2H, H-1); 3.72 (partly overlapping t and s, *J* = 9.6 Hz for t, 4H, H-2 and H-11); 3,51 (s, 2H, H-13′); 3.07 (br s, 2H, H-6); 2.67 (t, *J* = 5.6 Hz, 2H, H-8); 2.55 (t, *J* = 5.6 Hz, 2H, H-9). ^{13}C-NMR (DMSO-d_6): 165.3 (C-7″); 161.2 (C-5); 152.5 (C-3a); 147.8 (C-9a); 142.2 (C-1′); 138.2 (C-1′); 134.2 (C-4″); 130.8 (C-2′); 130.4 (C-5′); 129.04 (C-2″,6″); 129.02 (C-4′); 128.3 (C-6′); 127.9 (C-3″,5″); 123.2 (C-3′); 99.7 (C-5a); 96.3 (C-9″); 92.5 (C-12′); 81.7 (C-11′); 69.3 (η^5-$\underline{C}_5H_5$); 64.4 (C-11′,12″); 61.6 (C-10″,13″); 61.3 (C-11); 50.6 (C-1); 49.2 (C-6); 48.9 (C-8); 44.7 (C-10); 31.8 (C-13′); 26.2 (C-9). HRMS exact mass calcd. for $[C_{37}H_{36}FeN_6O_2]^+$: 653.2322. $[M+H]^+$; found: 653.22814; mass error: –6.22 ppm.

4-((4-(3-(3-Aminoprop-1-yn-1-yl)benzyl)-5-oxo-1,2,4,5,8,9-hexahydroimidazo[1,2-a]pyrido[3,4-e]pyrimidin-7(6H)-yl)methyl)-N-phenylbenzamide (33b): ^{1}H-NMR (DMSO-d_6): 10.22 (s, 1H, H-8″); 7.92 (d, *J* = 7.9 Hz, 2H, H-3″,5″); 7.78 (d, *J* = 7.7 Hz, 2H, H-10″,14″); 7.35 (t, *J* = 7.7 Hz, 2H, H-11″,13″); 7.48 (d, *J* = 7.9 Hz, 2H, H-2″,6″); 7.33-7.23 (overlapping m′s, 4H, H-2′, H-4′ H-5′ and H-6′); 7.10 (t, *J* = 7.7 Hz, 1H, H-12″); 4.88 (s, 2H, H-10); 3.96 (t, *J* = 9.6 Hz, 2H, H-1); 3.74 (partly overlapping t and s, *J* = 9.6 Hz for t, 4H, H-2 and H-11); 3,50 (s, 2H, H-13′); 3.06 (br s, 2H, H-6); 2.68 (t, *J* = 5.6 Hz, 2H, H-8); 2.55 (t, *J* = 5.6 Hz, 2H, H-9). ^{13}C-NMR (DMSO-d_6): 166.0 (C-7″); 161.2 (C-5); 152.5 (C-3a); 147.8 (C-9a); 142.5 (C-1′); 139.7 (C-9″); 138.3 (C-1′); 134.5 (C-4″); 130.7 (C-2′); 130.4 (C-4′); 129.09 (C-11″,13″); 129.02 (C-5′); 129.05 (C-2″,6″); 128.20 (C-6′); 128.18 (C-3″,5″); 124.1 (C-12″); 123.2 (C-3′); 120.07 (C-10″,14″); 99.6 (C-5a); 92.6 (C-12′); 81.7 (C-11′); 61.3 (C-11); 50.6 (C-1); 49.1 (C-6); 48.9 (C-8); 44.7 (C-10); 31.8 (C-13′); 26.2 (C-9). HRMS exact mass calcd. for $[C_{33}H_{32}N_6O_2]^+$: 545.2660 [M+H]$^+$; found: 545.26669; mass error: 1.27 ppm.

4-(3-(3-Aminoprop-1-yn-1-yl)benzyl)-7-(4-(4-(3-fluorophenyl)-1H-1,2,3-triazol-1-yl)benzyl)-2,4,6,7,8,9-hexahydroimidazo[1,2-a]pyrido[3,4-e]pyrimidin-5(1H)-one (36a): ^{1}H-NMR (DMSO-d_6): 9.39 (s, 1H, H-11″); 7.91 (d, *J* = 8.3 Hz, 2H, H-3″,5″); 7.81 (br d, *J* = 8.8 Hz, 1H, H-17″); 7.75 (br d, *J* = 10.1 Hz, 1H, H-13″); 7.59 (d, *J* = 8.3 Hz, 2H, H-2″,6″); 7.55 (t, *J* = 8.8 Hz, 1H, H-16″); 7.31–7.26 (overlapping m′s, 4H, H-2′, H-4′, H-5′, H-6′); 7.22 (td, *J* = 8.8 Hz and 2.4 Hz, 1H, H-15″); 4.88 (s, 2H, H-10); 3.96 (t, *J* = 9.5 Hz, 2H, H-1); 3.74 and 3.72 (overlapping t and s, *J* = 9.5 Hz for the t, 4H, H-11 and H-2); 3.51 (s, 2H, H-13′); 3.09 (br s, 2H, H-6); 2.69 (t, *J* = 5.7 Hz, 2H, H-8); 2.55 (t, *J* = 5.7 Hz, 2H, H-9). ^{13}C-NMR (DMSO-d_6): 163.1 (d, *J* = 243.1 Hz, C-14″); 161.2 (C-5); 152.5 (C-3a); 147.8 (C-9a); 146.7 (C-10″); 139.6 (C-1″); 138.3 (C-1′); 136.2 (C-4″), 133.1 (d, *J* = 8.1 Hz, C-12″); 133.3 (C-4′); 131.6 (d, *J* = 9.0 Hz, C-16″); 130.9 (C-2′); 130.6 (C-2″,6″); 130.2 (C-5′); 128.3 (C-6′); 123.4 (C-3′); 121.8 (d, *J* = 2.2 Hz, C-17″); 120.9 (C-11″); 120.5 (C-3″,5″); 115.4 (d, *J* = 20.1 Hz, C-15″); 112.4 (d, *J* = 22.1 Hz, C-13″); 99.6 (C-5a); 92.7 (C-12′); 81.8 (C-11″); 60.9 (C-11); 50.7 (C-1); 49.2 (C-6), 48.8 (C-8); 46.8 (C-9); 44.8 (C-10); 31.8 (C-13″); 26.2 (C-9). HRMS exact mass calcd. for $[C_{34}H_{31}FN_8O]^+$: 587.2678. [M+H]$^+$; found: 587.26811; mass error: 0.53 ppm.

4-(3-(3-Aminoprop-1-yn-1-yl)benzyl)-7-(4-(4-(4-fluorophenyl)-1H-1,2,3-triazol-1-yl)benzyl)-2,4,6,7,8,9-hexahydroimidazo[1,2-a]pyrido[3,4-e]pyrimidin-5(1H)-one (36b): ^{1}H-NMR (DMSO-d_6): 9.26 (s, 1H, H-11″); 7.91 (d, *J* = 8.4 Hz, 2H, H-3″,5″); 7.98 (dd, J = 8.5 Hz and 5.5 Hz), 2H, H-13″,17″); 7.57 (d, *J* = 8.4 Hz, 2H, H-2″,6″); 7.32-7.24 (overlapping m′s, 4H, H-2′, H-4′, H-5′, H-6′); 4.88 (s, 2H, H-10); 3.96 (t, *J* = 9.6 Hz, 2H, H-1); 3.73 (overlapping s and t, *J* = 9.6 Hz for the t, 4H, H-11 and H-2); 3.50 (s, 2H, H-13′); 3.10 (br s, 2H, H-6); 2.69 (t, *J* = 5.7 Hz, 2H, H-8); 2.56 (t, *J* = 5.7 Hz, 2H, H-9). ^{13}C-NMR (DMSO-d_6): 162.5 (d, *J* = 245.5 Hz, C-15″); 161.2 (C-5); 152.6 (C-3a); 147.9 (C-9a); 145.7 (C-10″); 139.3 (C-1″); 138.2 (C-1′); 136.2 (C-4″), 134.3 (t, *J* = 10.8 Hz, C-12″); 133.3 (C-4′); 130.9 (C-2′); 130.6 (C-2″,6″); 130.3 (C-5′); 128.3 (C-6′); 127.8 (d, *J* = 8.3 Hz, C-13″,17″); 127.3 (d, *J* = 2.9 Hz, C-12″); 123.3 (C-3′); 120.5 (C-3″,5″); 120.0 (C-11″); 116.4 (d, *J* = 21.7 Hz, C-14″,16″); 99.6 (C-5a); 92.6 (C-12′); 81.8 (C-11″); 61.0 (C-11); 50.6 (C-1); 49.2 (C-6), 48.8 (C-8); 46.8 (C-9); 44.7 (C-10); 31.8 (C-13″); 26.3 (C-9). HRMS exact mass calcd. for $[C_{34}H_{31}FN_8O]^+$: 587.2678. [M+H]$^+$; found: 587.26770; mass error: –0.17 ppm.

4-(3-(3-Aminoprop-1-yn-1-yl)benzyl)-7-(4-(4-(3,5-difluorophenyl)-1H-1,2,3-triazol-1-yl)benzyl)-2,4,6,7,8,9-hexahydroimidazo[1,2-a]pyrido[3,4-e]pyrimidin-5(1H)-one (36c): ^{1}H-NMR (DMSO-d_6): 9.41 (s, 1H, H-11″); 7.89 (d, *J* = 8.3 Hz, 2H, H-3″,5″); 7.65 (m, 2H, H-13″,17″); 7.59 (d, *J* = 8.3 Hz, 2H, H-2″,6″); 7.32-7.24 (overlapping m′s, 4H, H-2′, H-4′, H-5′, H-6′); 6.69 (tt, *J*=9.0 Hz and 2.3 Hz, 1H, H-15″); 4.88 (s, 2H, H-10); 3.96 (t, *J* = 9.6 Hz, 2H, H-1); 3.73 (overlapping s and t, *J* = 9.6 Hz for the t, 4H, H-11 and H-2); 3.50 (s, 2H, H-13′); 3.10 (br s, 2H, H-6); 2.69 (t, *J* = 5.7 Hz, 2H, H-8); 2.56 (t, *J* = 5.7 Hz, 2H, H-9). ^{13}C-NMR (DMSO-d_6): 163.4 (dd, *J* = 246.6 Hz and 15.1 Hz, C-14″,16″); 161.2 (C-5); 152.6 (C-3a); 147.9 (C-9a); 145.7 (C-10″); 139.3 (C-1″); 138.2 (C-1′); 136.2 (C-4″), 134.3 (t, *J* = 10.8 Hz, C-12″); 133.3 (C-4′); 130.9 (C-2′); 130.6 (C-2″,6″); 130.3 (C-5′); 128.3 (C-6′); 123.3 (C-3′); 121.6 (C-11″); 120.5 (C-3″,5″); 108.8 (dd, *J* = 20.4 Hz and 5.7 Hz, C-13″,17″); 103.9 (t, *J* = 25.7 Hz, C-15″); 99.7

(C-5a); 92.7 (C-12′); 81.8 (C-11″); 60.9 (C-11); 50.6 (C-1); 49.2 (C-6), 48.8 (C-8); 46.8 (C-9); 44.8 (C-10); 31.8 (C-13″); 26.3 (C-9). HRMS exact mass calcd. for $[C_{34}H_{30}F_2N_8O]^+$: 605.2583. $[M+H]^+$; found: -; mass error: -.

4-(3-(Aminomethyl)benzyl)-7-(4-(4-ferrocenyl-1H-1,2,3-triazol-1-yl)benzyl)-2,4,6,7,8,9-hexahydroimidazo[1,2-a]pyrido[3,4-e]pyrimidin-5(1H)-one (**37a**): ^{1}H-NMR (DMSO-d_6): 8.89 (s, 1H, H-11″); 7.91 (d, *J* = 8.2 Hz, 2H, H-3″,5″); 7.56 (d, *J* = 8.2 Hz, 2H, H-2″,5″); 7.25 (br s, 1H, H-2′); 7.23-7.18 (br m, 2H, H-4′ and H-5′); 7.12-7.09 (br m, 1H, H-6′); 4.89 (s, 2H, H-10); 4.80 br s, 2H, H-13″,16″); 4.36 br s, 2H, H-14″,15″); 4.10 (s, 5H, η^5-C$_5$H̲$_5$); 3.95 (t, *J* = 9.7 Hz, 2H, H-1); 3.72 and 3.70 (overlapping s and *t*, *J* = 9.7 Hz, 4H, H-11 and H-2); 3.67 (s, 2H, H-11′); 3.09 (br s, 2H, H-6); 2.69 (t, *J*=5.7 Hz, 2H, H-8); 2.56 (t, 2H, *J*=5.7 Hz, H-9). ^{13}C-NMR (DMSO-d_6): 161.2 (C-5); 152.6 (C-3a); 147.5 (C-9a); 147.0 (C-10″); 144.5 (C-3′); 139.2 (C-1″); 137,5 (C-1′); 136.1 (C-4″); 130.5 (C-2″,6″); 128.4 (C-5′); 126.9 (C-2′); 126.2 (C-3′); 125.9 (C-6′); 120.2 (C-3″,5″); 118.9 (C-11″); 99.7 (C-5a); 75.8 (C-12″); 69.8 (η^5-C̲$_5$H$_5$); 69.0 (C-14″,15″); 66.9 (C-13″,16″); 61.0 (C-11); 50.7 (C-1); 49.2 (C-6); 48.8 (C-8); 46.8 (C-2); 46.1 (C-11′); 45.1 (C-10); 26.2 (C-9). HRMS exact mass calcd. for $[C_{36}H_{36}FeN_8O]^+$: 653.2434. $[M+H]^+$; found: 653.24266; mass error: –1.13 ppm.

4-(3-(Aminomethyl)benzyl)-7-(4-(4-phenyl-1H-1,2,3-triazol-1-yl)benzyl)-2,4,6,7,8,9-hexahydroimidazo[1,2-a]pyrido[3,4-e]pyrimidin-5(1H)-one (**37b**): ^{1}H-NMR (DMSO-d_6): 9.28 (s, 1H, H-11″); 7.95 (d, *J* = 7.6 Hz, 2H, H-13″,17″); 7.92 (d, *J*=8.4 Hz, 2H, H-3′,5′); 7.57 (d, *J* = 8.2 Hz, 2H, H-2″,5″); 7.51 (d, *J* = 7.6 Hz, 2H, H-14″,16″); 7.39 (t, *J* = 7.6 Hz, 1H, H-15″); 7.25 (br s, 1H, H-2′); 7.23-7.18 (br m, 2H, H-4′ and H-5′); 7.12-7.09 (br m, 1H, H-6′); 4.89 (s, 2H, H-10); 3.94 (t, *J* = 9.5 Hz, 2H, H-1); 3.71 (overlapping s and *t*, *J* = 9.5 Hz, 4H, H-11 and H-2); 3.67 (s, 2H, H-11′); 3.09 (br s, 2H, H-6); 2.68, (t, *J*=5.7 Hz, 2H, H-8); 2.55 (t, 2H, *J*=5.7 Hz, H-9). ^{13}C-NMR (DMSO-d_6): 161.3 (C-5); 152.6 (C-3a); 147.7 (C-10″); 147.5 (C-9a); 144.4 (C-3′); 139.2 (C-1″); 137.6 (C-1′); 136.1 (C-4″); 130.8 (C-12″); 130.6 (C-14″,16″); 130.5 (C-2″,6″); 128.7 (C-15″); 128.4 (C-5′); 126.9 (C-2′); 126.3 (C-3′); 126.0 (C-6′); 125.8 (C-13″,17″); 120.4 (C-3″,5″); 120.1 (C-11″); 99.8 (C-5a); 61.1 (C-11); 50.6 (C-1); 49.2 (C-6); 48.8 (C-8); 46.9 (C-2); 46.0 (C-11′); 45.1 (C-10); 26.3 (C-9). HRMS exact mass calcd. for $[C_{32}H_{32}N_8O]^+$: 545.2772. $[M+H]^+$; found: .; mass error: -.

4-(3-(3-(Methylamino)prop-1-yn-1-yl)benzyl)-7-(4-(4-ferrocenyl-1H-1,2,3-triazol-1-yl)benzyl)-2,4,6,7,8,9-hexahydroimidazo[1,2-a]pyrido[3,4-e]pyrimidin-5(1H)-one (38a): ^{1}H-NMR (DMSO-d_6):8.89 (s, 1H, H-11″); 7.91 (d, *J* = 8.3 Hz, 2H, H-3″,5″); 7.56 (d, *J* = 8.3 Hz, 2H, H-2″,5″); 7.33-7.26 (overlapping m′s, 4H, H-2′, H-4′, H5′ and H-6′); 4.89 (s, 2H, H-10); 4.80 (br s, 2H, H-13″16″); 4.37 (br s, 2H, H-14″15″); 4.09 (s, 5H, η^5-C$_5$H̲$_5$); 3.97 (t, *J* = 9.7 Hz, 2H, H-1); 3.73 and 3.72 (overlapping s and t, *J* = 9.7 Hz for t, 4H, H-2 and H-11); 3.50 (s, 2H, H-13′); 3.09 (br s, 2H, 6-H); 2.69 (t, *J* = 5.7 Hz, 2H, H-8); 2.56 (t, *J* = 5.7 Hz, 2H, H-9); 2.33 (s, 3H, H-15′). ^{13}C-NMR (DMSO-d_6): 161.3 (C-5); 152.5 (C-3a); 147.8 (C-9a); 147.1 (C-10″); 139.2 (C-1″); 138.3 (C-1′); 136.1 (C-4″); 130.9 (C-2′); 130.54 (C-2″,6″); 130.49 (C-4′); 129.0 (C-5′); 128.3 (C-6′); 123.0 (C-3′); 120.2 (C-3″,5″); 118.9 (C-11′); 99.6 (C-5a); 89.3 (C-12′); 83.2 (C-11′); 76.8 (C-12″); 69.8 (η^5-C̲$_5$H$_5$); 69.0 (C-14″,15″); 66.9 (C-13″,16″); 61.0 (C-11); 50.6 (C-1); 49.1 (C-6); 48.8 (C-8); 46.8 (C-2); 44.7 (C-10); 40.59 (close to the *septet* signal of the solvent, C-13′); 35.3 (C-15′); 26.2 (C-9). HRMS exact mass calcd. for $[C_{39}H_{38}FeN_8O]^+$: 691.2591. $[M+H]^+$; found: 691.25909; mass error: –0.01 ppm.

4-(3-(3-(Methylamino)prop-1-yn-1-yl)benzyl)-7-(4-(4-phenyl-1H-1,2,3-triazol-1-yl)benzyl)-2,4,6,7,8,9-hexahydroimidazo[1,2-a]pyrido[3,4-e]pyrimidin-5(1H)-one (38b): ^{1}H-NMR (DMSO-d_6): 9.28 (s, 1H, H-11″); 7.95 (d, *J* = 7.6 Hz, 2H, H-13″,17″); 7.92 (d, *J* = 8.3 Hz, 2H, H-3″,5″); 7.58 (d, *J* = 8.3 Hz, 2H, H-2″,5″); 7.51 (t, *J* = 7.6 Hz, 2H, H-14″,16″); 7.39 (t, *J* = 7.6 Hz, 1H, H-15″); 7.33-7.26 (overlapping m′s, 4H, H-2′, H-4′, H5′ and H-6′); 4.88 (s, 2H, H-10); 3.96 (t, *J* = 9.7 Hz, 2H, H-1); 3.73 and 3.72 (overlapping s and t, *J* = 9.7 Hz for t, 4H, H-2 and H-11); 3.50 (s, 2H, H-13′); 3.09 (br s, 2H, 6-H); 2.69 (t, *J* = 5.7 Hz, 2H, H-8); 2.55 (t, *J* = 5.7 Hz, 2H, H-9); 2.33 (s, 3H, H-15′). ^{13}C-NMR (DMSO-d_6): 161.3 (C-5); 152.5 (C-3a); 147.79 (C-10″); 147.72 (C-9a); 139.6 (C-1″); 138.3 (C-

1′); 136.1 (two coalesced signals, C-4″ and C-12″); 130.9 (C-2′); 130.6 (C-2″,6″); 130.5 (C-4′); 129.5 (C-14″,16″); 129.0 (C-5′); 128.7 (C-15″); 128.3 (C-6′); 125.8 (C-13″,17″); 123.0 (C-3′); 120.4 (C-3″,5″); 120.1 (C-11′); 99.6 (C-5a); 89.4 (C-12′); 83.2 (C-11′); 60.9 (C-11); 50.6 (C-1); 49.2 (C-6); 48.8 (C-8); 46.8 (C-2); 44.7 (C-10); 40.59 (close to the *septet* signal of the solvent, C-13′); 35.4 (C-15′); 26.2 (C-9). HRMS exact mass calcd. for $[C_{35}H_{34}N_8O]^+$: 583.2928 $[M+H]^+$; found: 583.29369; mass error: 1.53 ppm.

4-(3-(3-(dimethylamino)prop-1-yn-1-yl)benzyl)-7-(4-(4-ferrocenyl-1H-1,2,3-triazol-1-yl)benzyl)-2,4,6,7,8,9-hexahydroimidazo[1,2-a]pyrido[3,4-e]pyrimidin-5(1H)-one (39a): ^{1}H-NMR (DMSO-*d*$_6$):8.87 (s, 1H, H-11″); 7.90 (d, *J* = 8.3 Hz, 2H, H-3″,5″); 7.56 (d, *J* = 8.3 Hz, 2H, H-2″,5″); 7.35 (br s, 1H, H-2′); 7.33-7.25 (overlapping m′s, 3H, H-4′, H5′ and H-6′); 4.89 (s, 2H, H-10); 4.80 (br s, 2H, H-13″16″); 4.37 (br s, 2H, H-14″15″); 4.09 (s, 5H, η^5-C$_5$H̲$_5$); 3.97 (br t, *J* = 10 Hz, 2H, H-1); 3.73 and 3.72 (overlapping s and br t, *J* ~ 10 Hz for t, 4H, H-2 and H-11); 3.42 (s, 2H, H-13′); 3.09 (br s, 2H, 6-H); 2.69 (br ~t, *J* ~ 6 Hz, 2H, H-8); 2.56 (br ~t, *J* ~ 6 Hz, 2H, H-9); 2.22 (s, 6H, H-15′,16′). ^{13}C-NMR (DMSO-*d*$_6$): 161.2 (C-5); 152.5 (C-3a); 147.8 (C-9a); 147.1 (C-10″); 139.2 (C-1″); 138.3 (C-1′); 136.1 (C-4″); 131.1 (C-2′); 130.6 (C-4′); 130.5 (C-2″,6″); 129.0 (C-5′); 128.4 (C-6′); 122.8 (C-3′); 120.2 (C-3″,5″); 118.9 (C-11′); 99.7 (C-5a); 86.1 (C-12′); 85.2 (C-11′); 75.8 (C-12″); 69.8 (η^5-C̲$_5$H$_5$); 69.0 (C-14″,15″); 66.9 (C-13″,16″); 61.0 (C-11); 50.6 (C-1); 49.1 (C-6); 48.8 (C-8);); 48.2 (C-13′); 46.8 (C-2); 44.7 (C-10); 44.3 (C-15′,16′); 26.2 (C-9). HRMS exact mass calcd. for $[C_{40}H_{40}FeN_8O]^+$: 705.2747. $[M+H]^+$; found: 705,27461; mass error: –0.13 ppm.

4-(3-(3-(dimethylamino)prop-1-yn-1-yl)benzyl)-7-(4-(4-phenyl-1H-1,2,3-triazol-1-yl)benzyl)-2,4,6,7,8,9-hexahydroimidazo[1,2-a]pyrido[3,4-e]pyrimidin-5(1H)-one (39b): ^{1}H-NMR (CDCl$_3$): 8.19 (s, 1H, H-11″); 7.93 (br d, *J* ‘ 8 Hz, 2H, H-13″,17″); 7.75 (d, *J* = 8.4 Hz, 2H, H-3″,5″); 7.53 (d, *J* = 8.4 Hz, 2H, H-2″,6″); 7.51 (br s, 1H, H-2′); 7.48 (t, *J* = 7.9 Hz, 2H, H-14″,16″); 7.42-7.37 (overlapping m′s, 2H, H-5′ and H-6′); 7.33 (br d *J* = 7.6 Hz, 1H, H-4′); 7.23 (t, *J* = 7.6 Hz, 1H, H-5′); 5.04 (s, 2H, H-10); 3.91 (br s, 4H, H-1 and H-2); 3.75 (s, 2H, H-11); 3.45 (s, 2H, H-13′); 3.33 (s, H-6); 2.72 (t, *J* = 5.7 Hz, 2H, H-8); 2.51 (t, *J* = 5.7 Hz, 2H, H-9); 2.37 (s, 6H, H-15′,16′).^{13}C-NMR (CDCl$_3$): 161.4 (C-5); 152.9 (C-3a); 148.4 (C-10″); 145.6 (C-9a); 139.0 (C-1″); 137.1 (C-1′); 137.0 (C-4″); 136.2 (C-12″); 131.5 (C-2′); 130.8 (C-4′); 130.3 (C-2″,6″); 128.9 (C-14″,16″); 128.43 and128.40 (C-5′and C-6′, interchangeable assignments); 128.2 (C-5″); 125.9 (C-13″,17″); 123.2 (C-3′); 120.7 (C-3″,5″); 117.7 (C-11″); 101.8 (C-5a); 85.3 (C-11′); 84.6 (C-12′); 61.5 (C-11); 50.6 (C-1); 49.5 (C-6); 48.6 (C-13′); 48.4 (C-8); 46.9 (C-2); 45.1 (C-10); 44.4 (C-15′,16′); 35.4 (C-15′); 26.8 (C-9). HRMS exact mass calcd. for $[C_{36}H_{36}N_8O]^+$: 597.3085 $[M+H]^+$; found: 597.30836; mass error: –0.13 ppm.

7-(4-(4-Ferrocenyl-1H-1,2,3-triazol-1-yl)benzyl)-4-(3-(3-(piperidin-1-yl)prop-1-yn-1-yl)benzyl)-2,4,6,7,8,9-hexahydroimidazo[1,2-a]pyrido[3,4-e]pyrimidin-5(1H)-one (40a): ^{1}H-NMR (CDCl$_3$): 7.89 (s, 1H, H-11″); 7.75 (d, *J* = 8.4 Hz, 2H, H-3″,5″); 7.52 (d, *J* = 8.4 Hz, 2H, H-2″,6″); 7.51 (br s, 1H, H-2′); 7.39 (d, *J* = 7.7 Hz, 1H, H-6′); 7.32 (d, *J* = 7.7 Hz, 1H, H-4′); 7.23 (t, *J* = 7.7 Hz, 1H, H-5′); 5.03 (s, 2H, H-10); 4.80 (t, *J* = 1.8 Hz, 2H, H-13″,16″); 4.35 (t, *J* = 1.8 Hz, 2H, H-14″,15″); 4.13 (s, 5H, η^5-C$_5$H̲$_5$); 3.91 (br s, 4H, H-1 and H-2); 3.74 (s, 2H, H-11); 3.46 (s, 2H, H-13′); 3.32 (s, 2H, H-6); 2.71 (t, *J* = 5.6 Hz, 2H, H-8); 2.57 (br~s, 4H, H-15′,19′); 2.51 (t, *J* = 5.6 Hz, 2H, H-9); 1.65 (qi, *J* = 5.8 Hz, 4H, H-16′,18′); 1.46 (br~s, 2H, H-17′). ^{13}C-NMR (CDCl$_3$): 161.3 (C-5); 153.0 (C-3a); 147.6 (C-10″); 145.6 (C-9a); 138.7 (C-1″); 137.0 (C-1′); 136.3 (C-4″); 131.4 (C-2′); 130.8 (C-5′); 130.2 (C-2″,6″), 128.4 (C-6′); 128.2 (C-4′); 123.0 (C-3′); 120.4 (C-3″,5″); 116.0 (C-11′); 101.7 (C-5a); 85.1 (C-12′); 85.0 (C-11′); 75.0 (C-12″); 69.6 (η^5-C̲$_5$H$_5$); 68.9 (C-14″,15″); 66.8 (C-13″,16″); 61.6 (C-11); 53.6 (C-15′,19′); 50.6 (C-1); 49.5 (C-6), 48.5 (C-13′); 48.4 (C-8); 46.9 (C-2); 45.0 (C-10); 26.8 (C-9); 26.0 (C-16′,18′); 23.9 (C-17′). HRMS exact mass calcd. for $[C_{43}H_{44}FeN_8O]^+$: 745.3060 $[M+H]^+$; found: 745.3060; mass error: 1.25 ppm.

7-(4-(4-Phenyl-1H-1,2,3-triazol-1-yl)benzyl)-4-(3-(3-(piperidin-1-yl)prop-1-yn-1-yl)benzyl)-2,4,6,7,8,9-hexahydroimidazo[1,2-a]pyrido[3,4-e]pyrimidin-5(1H)-one (40b): ^{1}H-NMR (CDCl$_3$): 7.92 (s, 1H, H-11″); 7.93 (dd, *J* = 7.5 Hz and 2.0 Hz, 2H, H-13″,17″); 7.75 (d, *J* = 8.4 Hz, 2H, H-3″,5″); 7.53 (d, *J* = 8.4 Hz, 2H, H-2″,6″);

7.51 (br s, 1H, H-2′); 7.47 (t, J = 7.5 Hz, 2H, H-14″,16″); 7.40-7.38 (overlapping m′s, 2H, H-6′ and H-15″); 7.32 (d, J = 7.7 Hz, 1H, H-4′); 7.22 (t, J = 7.7 Hz, 1H, H-5′); 5.03 (s, 2H, H-10); 3.92 (br s, 4H, H-1 and H-2); 3.74 (s, 2H, H-11); 3.45 (s, 2H, H-13′); 3.33 (s, 2H, H-6); 2.72 (t, J = 5.6 Hz, 2H, H-8); 2.57 (br~s, 4H, H-15′,19′); 2.51 (t, J = 5.6 Hz, 2H, H-9); 1.65 (qi, J = 5.8 Hz, 4H, H-16′,18′); 1.46 (br~s, 2H, H-17′). ^{13}C-NMR ($CDCl_3$): 161.3 (C-5); 152.9 (C-3a); 148.4 (C-10″); 145.6 (C-9a); 138.9 (C-1″); 137.0 (C-1′); 136.2 (two coalesced lines, C-4″ and C-12″); 131.4 (C-2′); 130.8 (C-5′); 130.3 (C-2″,6″), 128.9 (C-14″,16″); 128.4 (C-6′); 128.3 (C-15″); 128.2 (C-4′); 125.9 (C-13″,17″); 123.3 (C-3′); 120.6 (C-3″,5″); 117.6 (C-11′); 101.7 (C-5a); 85.04 and 85.05 (interchangeable signals,C-12′ and C-11′); 61.5 (C-11); 53.5 (C-15′,19′); 50.6 (C-1); 49.5 (C-6), 48.5 (two coalesced lines, C-8 and C-13′); 46.9 (C-2); 45.0 (C-10); 26.8 (C-9); 255.9 (C-16′,18′); 23.9 (C-17′). HRMS exact mass calcd. for $[C_{39}H_{40}N_8O]^+$: 637.3398 [M+H]$^+$; found: 637.34082; mass error: 1.60 ppm.

4-(3-(3-(4-Methylpiperazin-1-yl)prop-1-yn-1-yl)benzyl)-7-(4-(4-ferrocenyl-1H-1,2,3-triazol-1-yl)benzyl)-2,4,6,7,8,9-hexahydroimidazo[1,2-a]pyrido[3,4-e]pyrimidin-5(1H)-one (41a): ^{1}H-NMR ($CDCl_3$): 7.89 (s, 1H, H-11″); 7.77 (d, J = 8.4 Hz, 2H, H-3″,5″); 7.52 (d, J = 8.4 Hz, 2H, H-2″,6″); 7.49 (t, J = 1.5 Hz, 1H, H-2′); 7.39 (d, J = 7.7 Hz, 1H, H-6′); 7.32 (dt, J = 7.7 Hz and 1.5 Hz, 1H, H-4′); 7.22 (t, J = 7.7 Hz, 1H, H-5′); 5.03 (s, 2H, H-10); 4.80 (t, J = 1.8 Hz, 2H, H-13″,16″); 4.35 (t, J = 1.8 Hz, 2H, H-14″,15″); 4.13 (s, 5H, η^5-$C_5\underline{H}_5$); 3.92 (br s, 4H, H-1 and H-2); 3.75 (s, 2H, H-11); 3.50 (s, 2H, H-13′); 3.32 (s, 2H, H-6); 2.71 (t, J = 5.6 Hz, 2H, H-8); 2.7 and 2.5 (two very br ~s′s, ~2x2H, H-15′,19′ and H-16′,18′); 2.31 (s, 3H, H-20′). ^{13}C-NMR ($CDCl_3$): 161.2 (C-5); 152.9 (C-3a); 147.5 (C-10″); 145.6 (C-9a); 138.6 (C-1″); 137.0 (C-1′); 136.3 (C-4″); 131.4 (C-2′); 130.8 (C-5′); 130.2 (C-2″,6″); 128.4 (C-6′); 128.1 (C-4′); 123.1 (C-3′); 120.4 (C-3″,5″); 116.6 (C-11′); 101.7 (C-5a); 85.4 (C-12′); 84.4 (C-11′); 74.9 (C-12″); 69.6 (η^5-$\underline{C}_5H_5$); 68.8 (C-14″,15″); 66.8 (C-13″,16″); 61.6 (C-11); 55.1 (C-16′,18′); 52.2 (C-15′,19′); 50.6 (C-1); 49.4 (C-6); 48.4 (C-8); 47.7 (C-13′); 46.9 (C-2); 46.0 (C-20′); 45.1 (C-10); 26.8 (C-9). HRMS exact mass calcd. for $[C_{43}H_{45}FeN_9O]^+$: 760.3169 [M+H]$^+$; found: 760.3179; mass error: 1.32 ppm.

4-(3-(3-(4-Methylpiperazin-1-yl)prop-1-yn-1-yl)benzyl)-7-(4-(4-phenyl-1H-1,2,3-triazol-1-yl)benzyl)-2,4,6,7,8,9-hexahydroimidazo[1,2-a]pyrido[3,4-e]pyrimidin-5(1H)-one (41b): ^{1}H-NMR ($CDCl_3$): 8.20 (s, 1H, H-11″); 7.92 (d, J = 7.6 Hz, 2H, H-13″,17″); 7.75 (d, J = 8.4 Hz, 2H, H-3″,5″); 7.53 (d, J = 8.4 Hz, 2H, H-2″,6″); 7.49 (br s, 1H, H-2′); 7.47 (t, J = 7.6 Hz, 2H, H-14″,16″); 7.41-7.36 (overlapping m′s, H-6′ and H15″); 7.32 (d, J = 7.7 Hz, 1H, H-4′); 7.22 (t, J = 7.7 Hz, 1H, H-5′); 5.03 (s, 2H, H-10); 3.92 (br s, 4H, H-1 and H-2); 3.74 (s, 2H, H-11); 3.51 (s, 2H, H-13′); 3.33 (s, 2H, H-6); 2.7 (overlapping m′s, 6H, H-8 and H-15′,19′); 2.5 (overlapping m′s, 6H, H-9 and H-16′,18′); 2.32 (s, 3H, H-20′). ^{13}C-NMR ($CDCl_3$): 161.4 (C-5); 152.9 (C-3a); 148.4 (C-10″); 145.6 (C-9a); 138.9 (C-1″); 137.0 (C-1′); 136.2 (C-4″); 131.4 (C-2′); 130.8 (C-4′); 130.3 (two coalesced lines, C-2″,6″ and C-12″); 128.9 (C-14″,16″); 128.5 (C-6′); 128.4 (C-5′); 128.2 (C-15″); 125.9 (C-13″,17″); 123.1 (C-3′); 120.6 (C-3″,5″); 117.6 (C-11′); 101.6 (C-5a); 85.3 (C-12′); 84.4 (C-11′); 61.5 (C-11); 55.0 (C-16′,18′); 52.2 (C-15′,19′); 50.6 (C-1); 49.5 (C-6); 48.4 (C-8); 47.8 (C-13′); 46.9 (C-2); 46.0 (C-20′); 45.1 (C-10); 26.8 (C-9). HRMS exact mass calcd. for $[C_{39}H_{41}N_9O]^+$: 652.3507. [M+H]$^+$; found: 652.35219; mass error: 2.28 ppm.

4-(3-(3-Morpholinoprop-1-yn-1-yl)benzyl)-7-(4-(4-ferrocenyl-1H-1,2,3-triazol-1-yl)benzyl)-2,4,6,7,8,9-hexahydroimidazo[1,2-a]pyrido[3,4-e]pyrimidin-5(1H)-one (42a): ^{1}H-NMR ($CDCl_3$): 7.89 (s, 1H, H-11″); 7.75 (d, J = 8.3 Hz, 2H, H-3″,5″); 7.53 and 7.51 (partly overlapping d and br s, J = 8.3 Hz for t, 3H, H-2″,6″ and H-2′); 7.41 (d, J = 7.6 Hz, 1H, H-6′); 7.32 (br d, J = 7.6 Hz, 1H, H-4′); 7.24 (t, J = 7.6 Hz, 1H, H-5′); 5.03 (s, 2H, H-10); 4.80 (br s, 2H, H-13″,16″); 4.35 (br s 2H, H-14″,15″); 4.13 (s, 5H, η^5-$C_5\underline{H}_5$); 3.92 (br s, 4H, H-1 and H-2); 3.78 (t, J = 4.6 Hz, 4H, H-16′,18′); 3.74 (s, 2H, H-11); 3.50 (s, 2H, H-13′); 3.33 (s, 2H, H-6); 2.71 (t, J = 5.6 Hz, 2H, H-8); 2.64 (t, J = 4.6 Hz, 4H, H-15′,19′); 2.51 (t, J = 5.6 Hz, 2H, H-9). ^{13}C-NMR ($CDCl_3$): 161.4 (C-5); 152.9 (C-3a); 147.6 (C-10″); 145.6 (C-9a); 138.6 (C-1″); 137.1 (C-1′); 136.3 (C-4″); 131.4 (C-2′); 130.8 (C-5′); 130.3 (C-2″,6″); 128.6 (C-6′); 128.2 (C-4′); 123.1 (C-3′); 120.4 (C-3″,5″); 116.6 (C-11′); 101.8 (C-5a); 85.6 (C-12′); 84.0 (C-11′); 75.0 (C-12″); 69.5 (η^5-$\underline{C}_5H_5$); 68.8 (C-14″,15″); 66.8 (C-13″,16″); 66.9 (C-16′,18′); 61.6 (C-11); 52.6 (C-15′,19′); 50.6 (C-1); 49.4 (C-6); 48.2

(C-13′); 48.1 (C-8); 46.9 (C-2); 45.1 (C-10); 26.8 (C-9). HRMS exact mass calcd. for $[C_{42}H_{42}FeN_8O_2]^+$: 747.2853. $[M+H]^+$; found: 747.28687; mass error: 2.10 ppm.

4-(3-(3-Morpholinoprop-1-yn-1-yl)benzyl)-7-(4-(4-phenyl-1H-1,2,3-triazol-1-yl)benzyl)-2,4,6,7,8,9-hexahydroimidazo[1,2-a]pyrido[3,4-e]pyrimidin-5(1H)-one (42b): ^{1}H-NMR ($CDCl_3$): 8.20 (s, 1H, H-11″); 7.92 (d, *J* = 7.6 Hz, 2H, H-13″,17″); 7.75 (d, *J* = 8.4 Hz, 2H, H-3″,5″); 7.52 (overlapping br s and d *J* = 8.4 Hz for the d, 3H, H-2′ and H-2″,6″); 7.48 (t, *J* = 7.6 Hz, 2H, H-14″,16″); 7.42 (br d, *J* = 7.7 Hz, 1H, H-6′); 7.38 (t, *J* = 7.6 Hz, 1H, H-15″); 7.32 (br d, *J* = 7.7 Hz, 1H, H-4′); 7.23 (t, *J* = 7.7 Hz, 1H, H-5′); 5.03 (s, 2H, H-10); 3.91 (br s, 4H, H-1 and H-2); 3.77 (t, *J* = 5.2 Hz, 4H, H-16′,18′); 3.74 (s, 2H, H-11); 3.48 (s, 2H, H-13′); 3.32 (br s, 2H, H-6); 2.71 (t, *J* = 5.7 Hz, 2H, H-8); 2.64 (br ~t, *J* ~ 5 Hz, 4H, H-15′,19′); 2.56 (t, *J* = 5.7 Hz, 2H, H-9). ^{13}C-NMR ($CDCl_3$): 161.4 (C-5); 152.9 (C-3a); 148.4 (C-10″); 145.6 (C-9a); 138.9 (C-1″); 137.1 (C-1′); 136.2 (C-4″); 131.5 (C-2′); 130.8 (C-4′); 130.3 (two coalesced lines, C-2″,6″ and C-12″); 128.9 (C-14″,16″); 128.6 (C-6′); 128.4 (C-5′); 128.2 (C-15″); 125.9 (C-13″,17″); 122.9 (C-3′); 120.6 (C-3″,5″); 117.6 (C-11′); 101.6 (C-5a); 85.6 (C-12′); 84.1 (C-11′); 66.9 (C-16′,18′); 61.5 (C-11); 52.6 (C-15′,19′); 50.6 (C-1); 49.5 (C-6); 48.4 (C-8); 48.1 (C-13′); 46.9 (C-2); 45.0 (C-10); 26.8 (C-9). HRMS exact mass calcd. for $[C_{38}H_{38}N_8O_2]^+$: 639.3190. $[M+H]^+$; found: 639.31912; mass error: 0.19 ppm.

7-(4-(4-(3-Fluorophenyl)-1H-1,2,3-triazol-1-yl)benzyl)-4-(3-(3-(methylamino)prop-1-yn-1-yl)benzyl)-2,4,6,7,8,9-hexahydroimidazo[1,2-a]pyrido[3,4-e]pyrimidin-5(1H)-one (43a): ^{1}H-NMR (DMSO-*d*$_6$): 9.35 (s, 1H, H-11″); 7.91 (d, *J* = 8.3 Hz, 2H, H-3″,5″); 7.81 (br d, *J* = 8.8 Hz Hz, 2H, H-17″); 7.75 (br d, *J* = 10.1 Hz, 1H, H-13″); 7.59 (d, *J* = 8.3 Hz, 2H, H-2″,5″); 7.55 (t, *J* = 8.8 Hz, 1H, H-16″); 7.32 (br s, 1H, H-2′); 7.30-7.26 (overlapping m′s, 3H, H-4′, H5′ and H-6′); 7.22 (td, *J* = 8.8 Hz and 2.4 Hz, 1H, H-15″); 4.88 (s, 2H, H-10); 3.95 (t, *J* = 9.0 Hz, 2H, H-1); 3.72 and 3.71 (overlapping s and t, *J* = 9.0 Hz for t, 4H, H-11 and H-2); 3.49 (s, 2H, H-13′); 3.09 (br s, 2H, 6-H); 2.68 (t, *J* = 5.6 Hz, 2H, H-8); 2.55 (t, *J* = 5.6 Hz, 2H, H-8); 2.33 (s, 3H, H-15′). ^{13}C-NMR (DMSO-*d*$_6$): 163.1 (d, *J* = 243.0 Hz, C-14″); 161.2 (C-5); 152.5 (C-3a); 147.8 (C-9a); 146.7 (C-10″); 139.7 (C-1″); 138.3 (C-1′); 136.0 (C-4″); 133.1 (d, *J* = 8.1 Hz, C-12″); 131.6 (d, *J* = 9.0 Hz, C-16″); 130.9 (C-6′); 130.6 (C-2″,6″); 130.5 (C-4′); 129.0 (C-5′); 128.3 (C-2′); 123.1 (C-3′); 120.5 (C-3″,5″); 120.9 (C-11′); 115.4 (d, *J* = 20.1 Hz, C-15″); 112.4 (d, *J* = 23.1 Hz, C.13″); 99.6 (C-5a); 89.5 (C-12′); 83.1 (C-11′); 60.9 (C-11); 50.8 (C-1); 49.2 (C-6); 48.8 (C-8); 46.8 (C-2); 44.7 (C-10); 40.5 (overlapped by the solvent signal, C-13′), 35.4 (C-15′); 26.2 (C-9). HRMS exact mass calcd. for $[C_{35}H_{33}FN_8O]^+$: 601.2834. $[M+H]^+$; found: 601.28288; mass error: –0.86 ppm.

7-(4-(4-(4-Fluorophenyl)-1H-1,2,3-triazol-1-yl)benzyl)-4-(3-(3-(methylamino)prop-1-yn-1-yl)benzyl)-2,4,6,7,8,9-hexahydroimidazo[1,2-a]pyrido[3,4-e]pyrimidin-5(1H)-one (43b): ^{1}H-NMR (DMSO-*d*$_6$): 9.26 (s, 1H, H-11″); 7.98 (dd, *J* = 8.5 Hz and 5.5 Hz, 2H, H-13″,17″); 7.91 (d, *J* = 8.4 Hz, 2H, H-3″,5″); 7.57 (d, *J* = 8.5 Hz, 2H, H-2″,5″); 7.35 (t, *J* = 8.5 Hz, 2H, H-14″,16″); 7.31 (br s, 1H, H-2′); 7.30-7.26 (overlapping m′s, 3H, H-4′, H5′ and H-6′); 4.88 (s, 2H, H-10); 3.95 (t, *J* = 9.2 Hz, 2H, H-1); 3.71 and 3.70 (overlapping s and t, *J* = 9.2 Hz for t, 4H, H-11 and H-2); 3.49 (s, 2H, H-13′); 3.09 (br s, 2H, 6-H); 2.68 (t, *J* = 5.6 Hz, 2H, H-8); 2.54 (t, *J* = 5.6 Hz, 2H, H-8); 2.32 (s, 3H, H-15′). ^{13}C-NMR (DMSO-*d*$_6$): 162.5 (d, *J* = 245.5 Hz, C-15″); 161.3 (C-5); 152.5 (C-3a); 147.8 (C-9a); 146.9 (C-10″); 139.6 (C-1″); 138.3 (C-1′); 136.1 (C-4″); 131.0 (C-6′); 130.6 (C-2″,6″); 130.5 (C-4′); 129.0 (C-5′); 128.3 (C-2′); 127.8 (d, *J* = 8.3 Hz, C-13″,17″); 127.3 (d, *J* = 2.9 Hz, C-12″); 123.2 (C-3′); 120.5 (C-3″,5″); 120.2 (C-11′); 116.4 (d, *J* = 21.7 Hz, C-14″,16″); 99.6 (C-5a); 89.6 (C-12′); 83.4 (C-11′); 61.0 (C-11); 50.6 (C-1); 49.2 (C-6); 48.8 (C-8); 46.8 (C-2); 44.7 (C-10); 40.5 (overlapped by the solvent signal, C-13′), 35.4 (C-15′); 26.3 (C-9). HRMS exact mass calcd. for $[C_{35}H_{33}FN_8O]^+$: 601.2834. $[M+H]^+$; found: 601.28303; mass error: –0.62 ppm.

7-(4-(4-(3,5-Difluorophenyl)-1H-1,2,3-triazol-1-yl)benzyl)-4-(3-(3-(methylamino)prop-1-yn-1-yl)benzyl)-2,4,6,7,8,9-hexahydroimidazo[1,2-a]pyrido[3,4-e]pyrimidin-5(1H)-one (43c): ^{1}H-NMR (DMSO-*d*$_6$): 9.39 (s, 1H, H-11″); 7.90 (d, *J* = 8.3 Hz, 2H, H-3″,5″); 7.65 (m, 2H, H-13″,17″); 7.59 (d, *J* = 8.3 Hz, 2H, H-2″,6″); 7.32 (br s, 1H, H-2′); 7.30-7.26 (overlapping m′s, 3H, H-4′, H-5′, H-6′); 6.69 (tt, *J*=9.0 Hz and 2.3 Hz, 1H, H-15″); 4.88 (s, 2H, H-

10); 3.96 (t, *J* = 9.6 Hz, 2H, H-1); 3.73 (overlapping s and t, *J* = 9.6 Hz for the t, 4H, H-11 and H-2); 3.49 (s, 2H, H-13′); 3.09 (br s, 2H, H-6); 2.69 (t, *J* = 5.7 Hz, 2H, H-8); 2.56 (t, *J* = 5.7 Hz, 2H, H-9); 2.32 (s, 3H, H-15′). ^{13}C-NMR (DMSO-d_6): 163.4 (dd, *J* = 246.6 Hz and 15.4 Hz, C-14″,16″); 161.3 (C-5); 152.5 (C-3a); 147.8 (C-9a); 145.7 (C-10″); 139.1 (C-1″); 138.3 (C-1′); 136.1 (C-4″), 134.3 (t, *J* = 10.8 Hz, C-12″); 131.2 (C-2′); 131.0 (C-6′); 130.6 (C-2″,6″); 130.3 (C-4′); 130.3 (C-5′); 123.3 (C-2′); 120.8 (C-11″); 120.5 (C-3″,5″); 108.8 (dd, *J* = 20.4 Hz and 5.7 Hz, C-13″,17″); 103.9 (t, *J* = 25.9 Hz, C-15″); 99.6 (C-5a); 89.6 (C-12′); 83.4 (C-11″); 61.0 (C-11); 50.6 (C-1); 49.2 (C-6), 48.8 (C-8); 46.8 (C-9); 44.7 (C-10); 40.5 (C-13″); 35.4 (C-15′); 26.3 (C-9). HRMS exact mass calcd. for $[C_{35}H_{32}F_2N8O]^+$: 619.2740. [M+H] $^+$; found: 619.27594; mass error: 3.13 ppm.

S3. Copy of ^{1}H- and ^{13}C-NMR spectra of the targeted compounds

^{1}H-NMR of **1a**

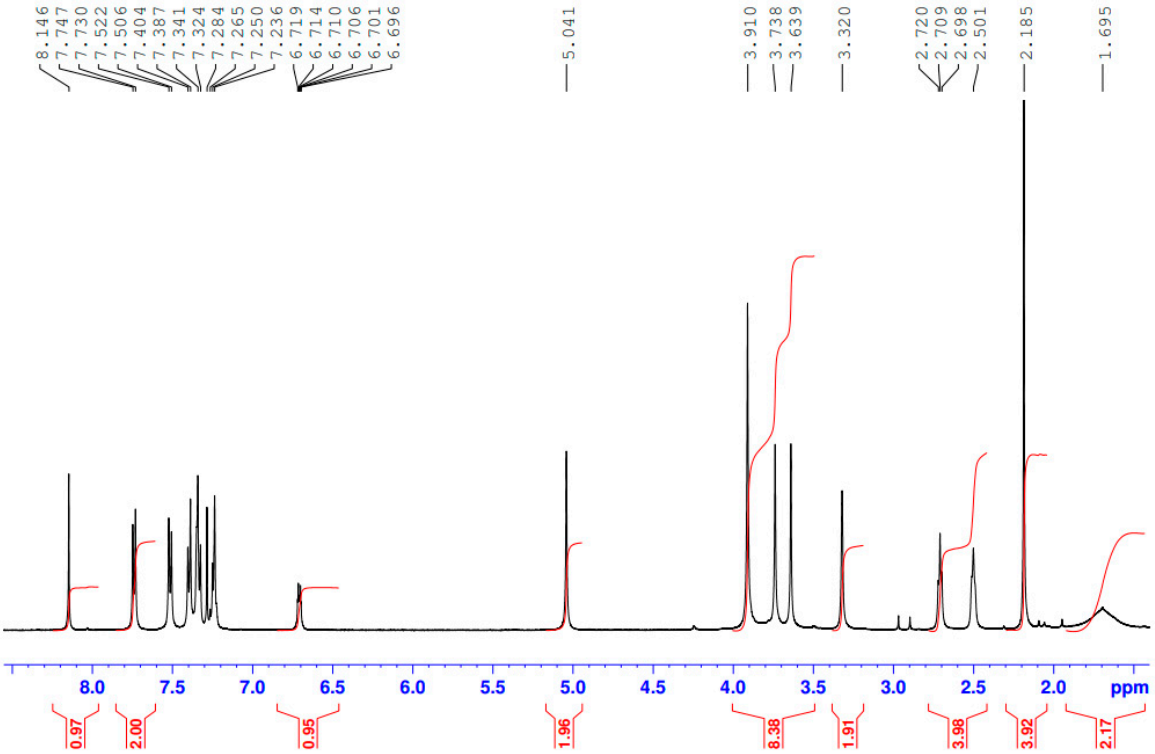

^{13}C-NMR of **1a**

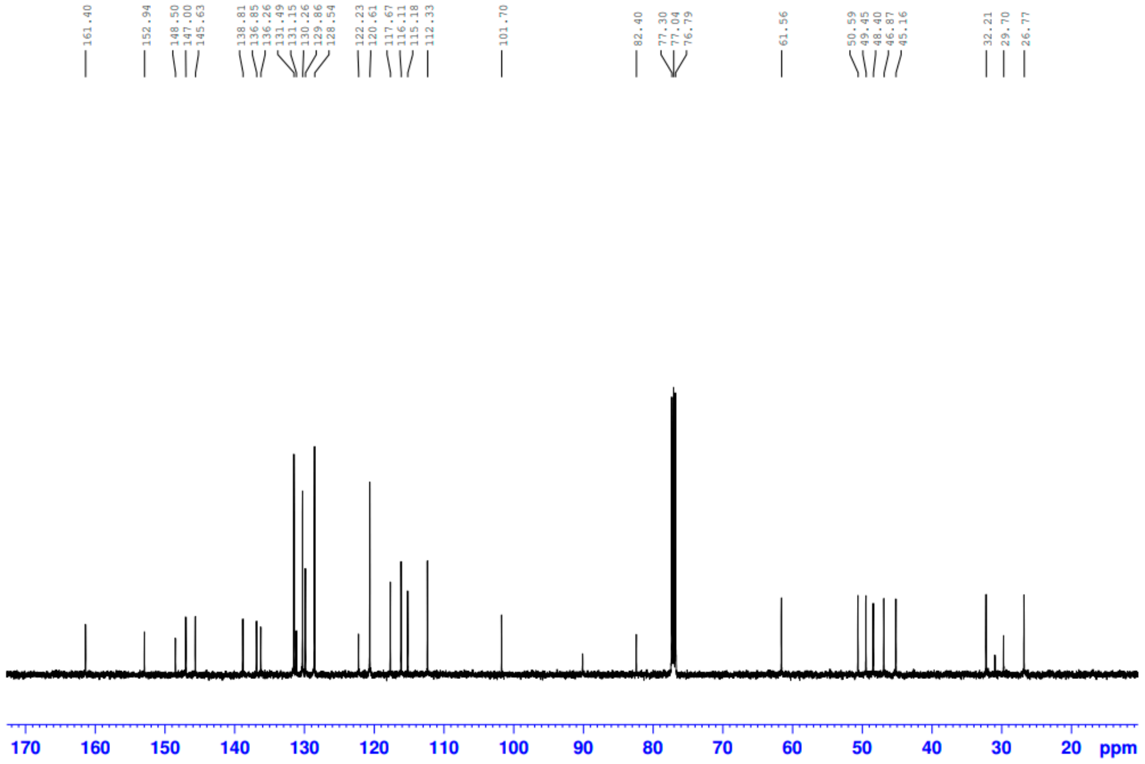

^{1}H-NMR of **1b**

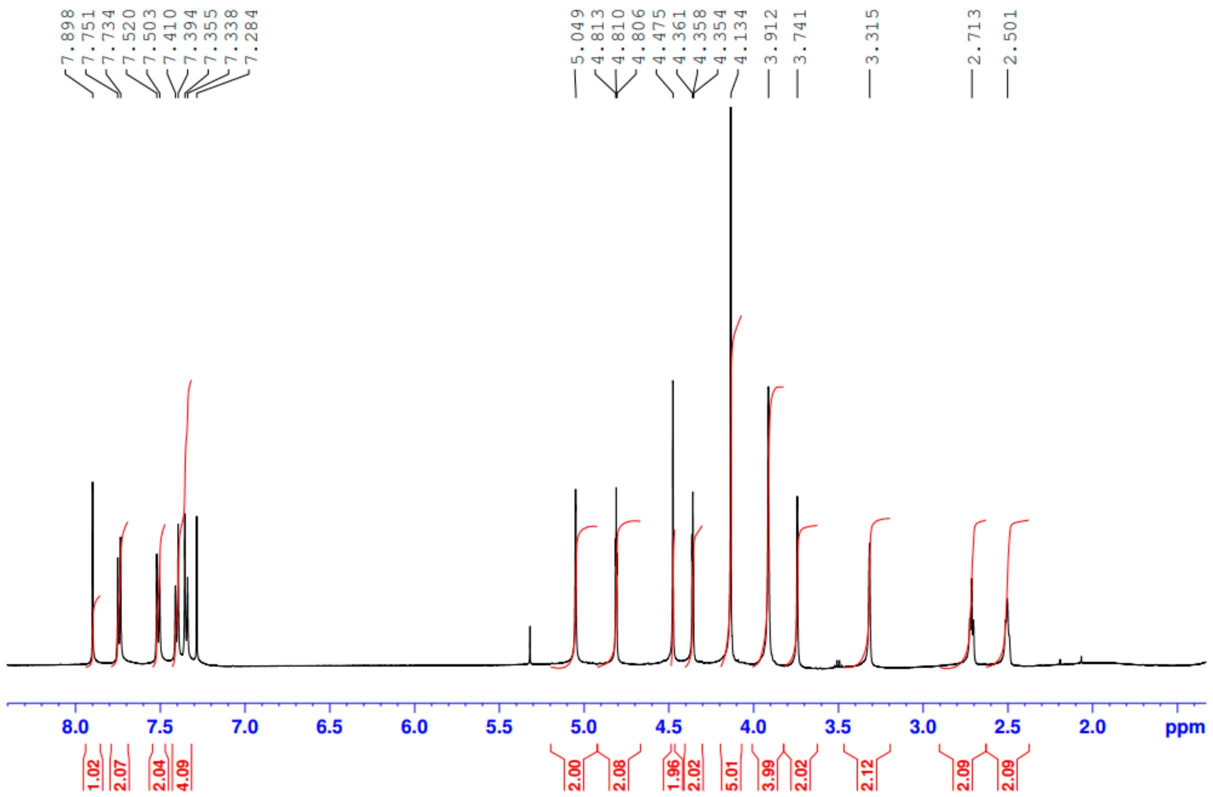

^{13}C-NMR of **1b**

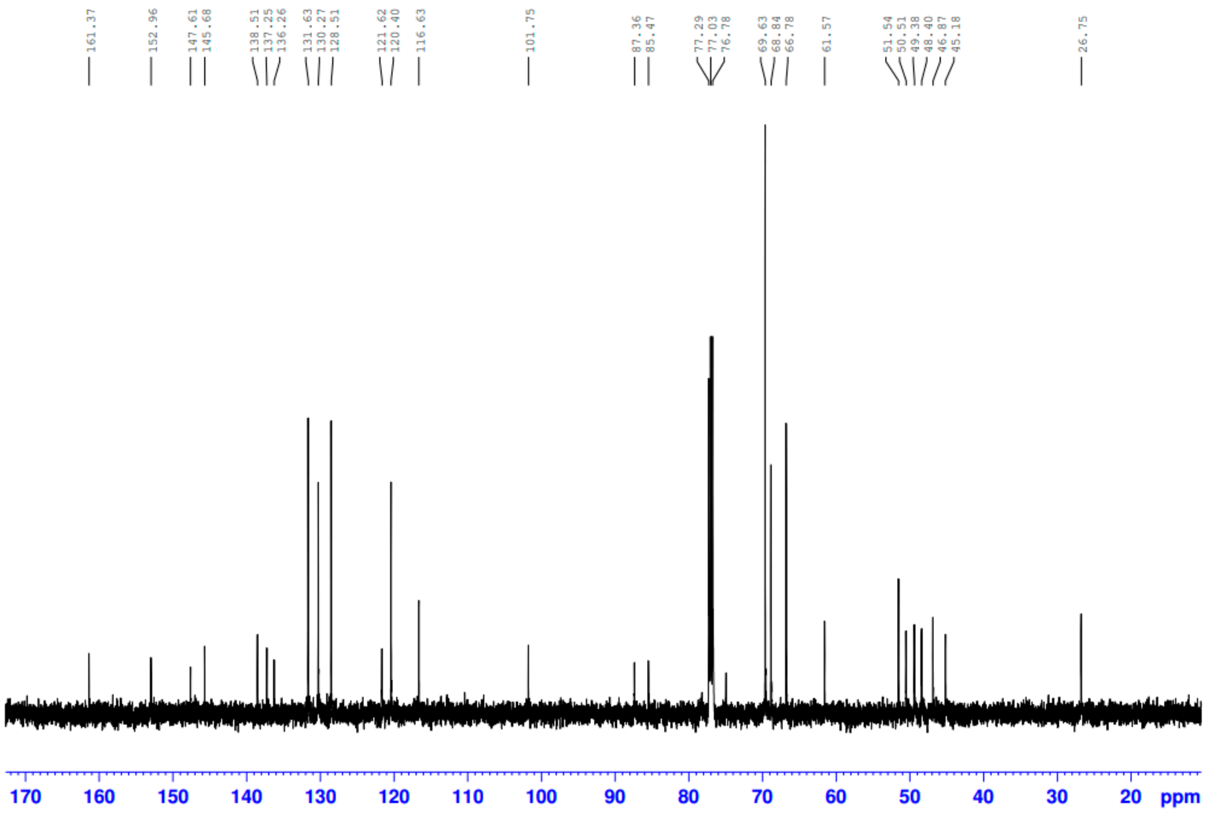

^{1}H-NMR of **1c**

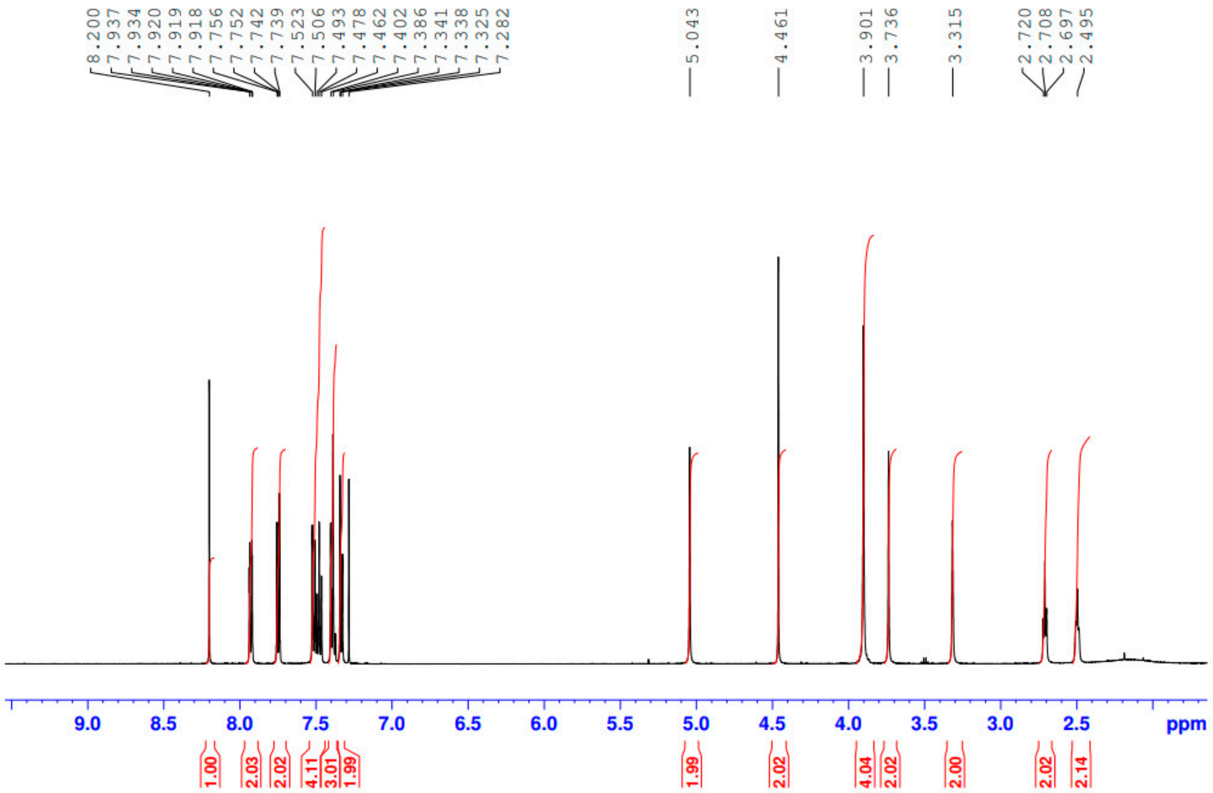

^{13}C-NMR of **1c**

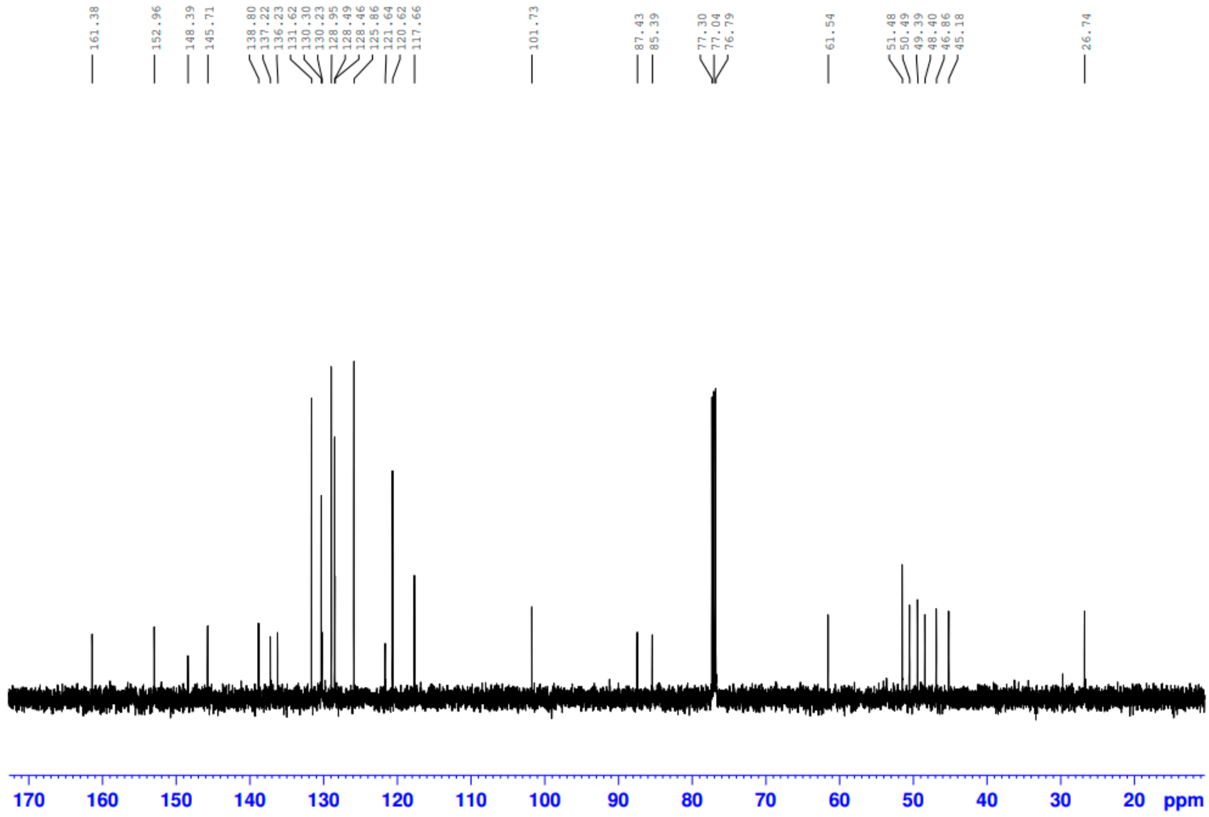

^{1}H-NMR of **1d**

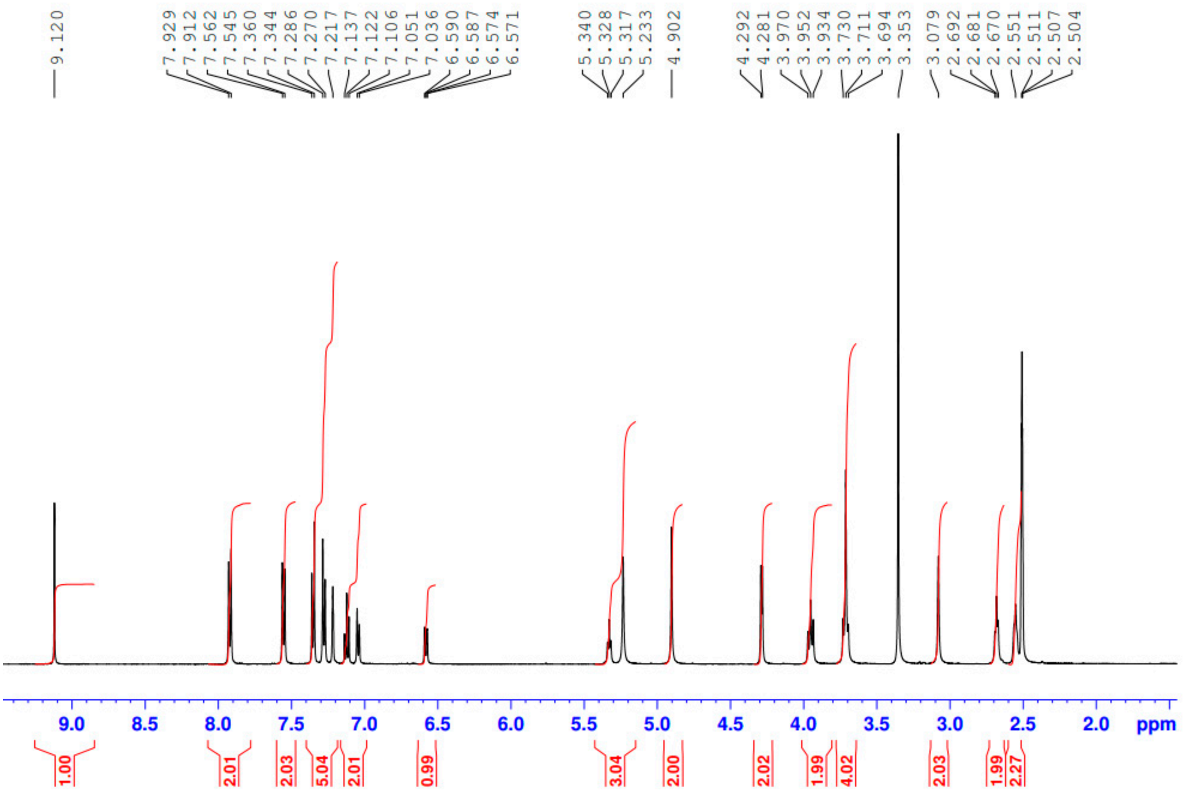

^{13}C-NMR of **1d**

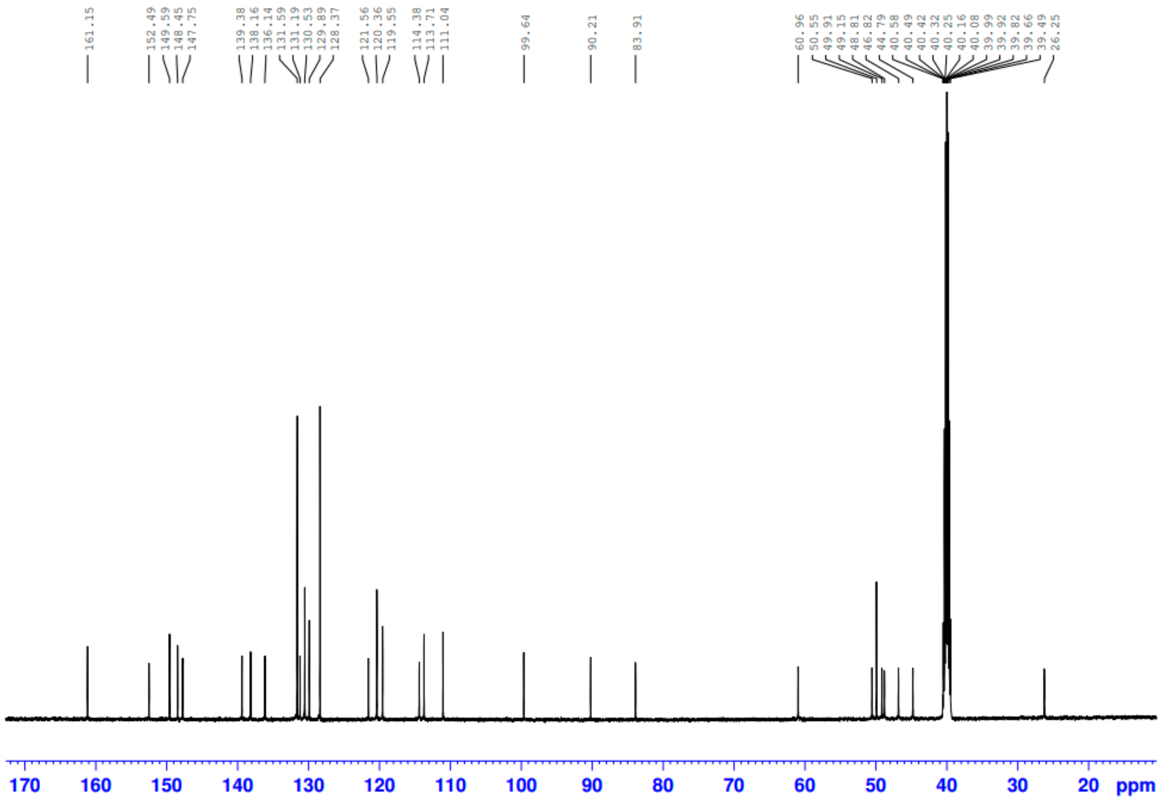

^{1}H-NMR of **1e**

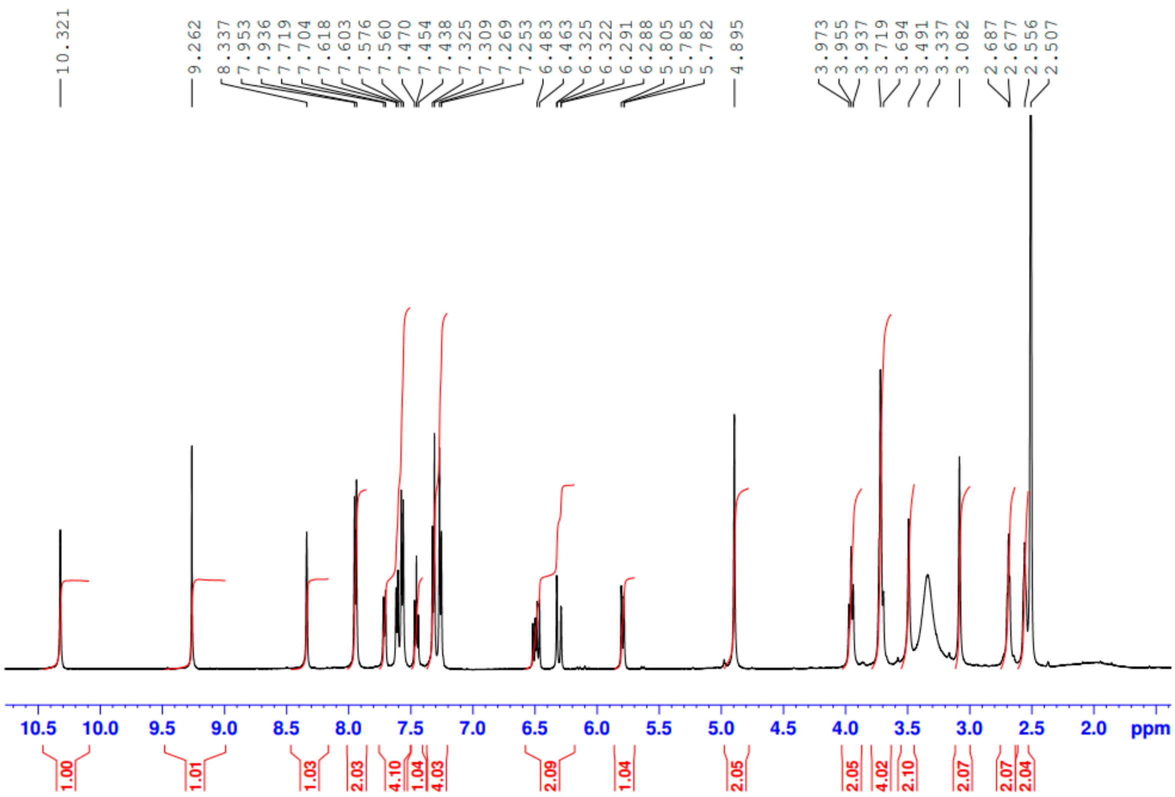

^{13}C-NMR of **1d**

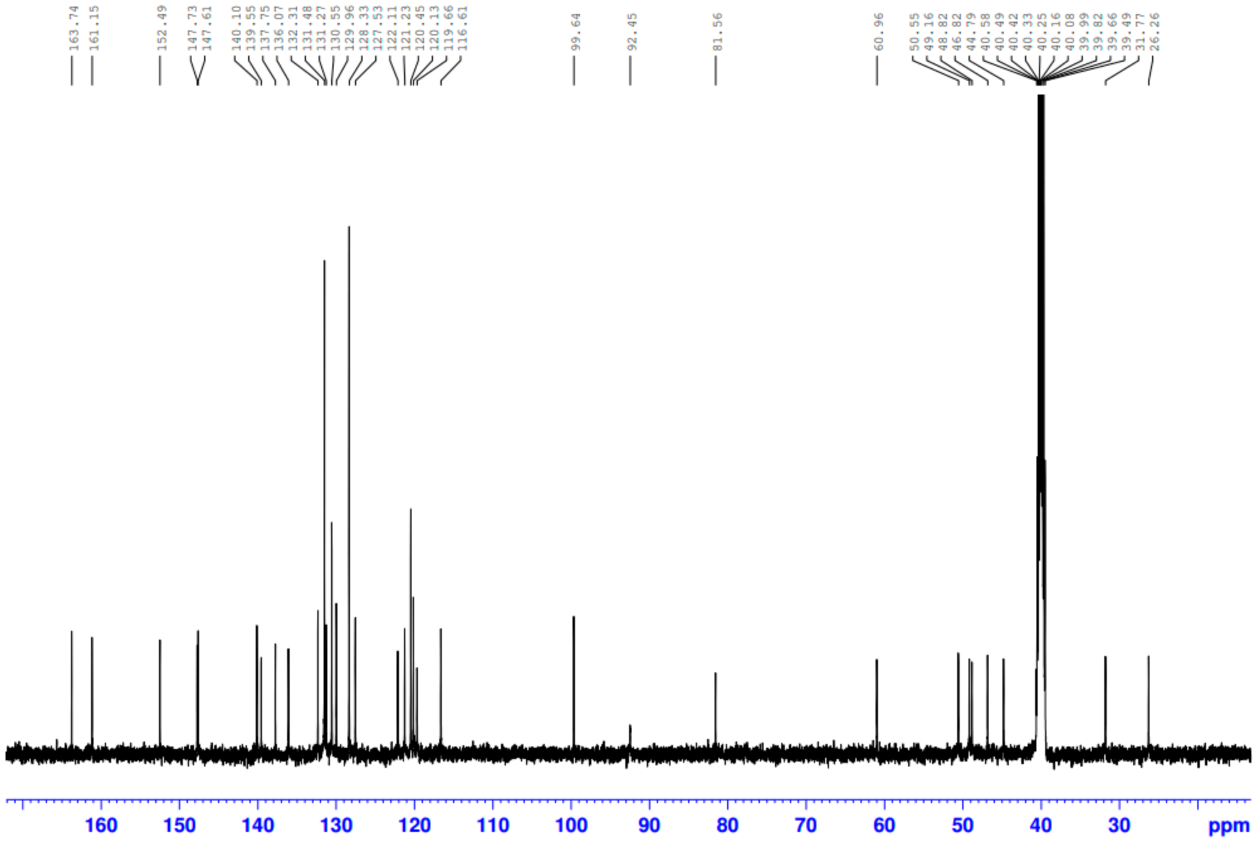

^{1}H-NMR of **2a**

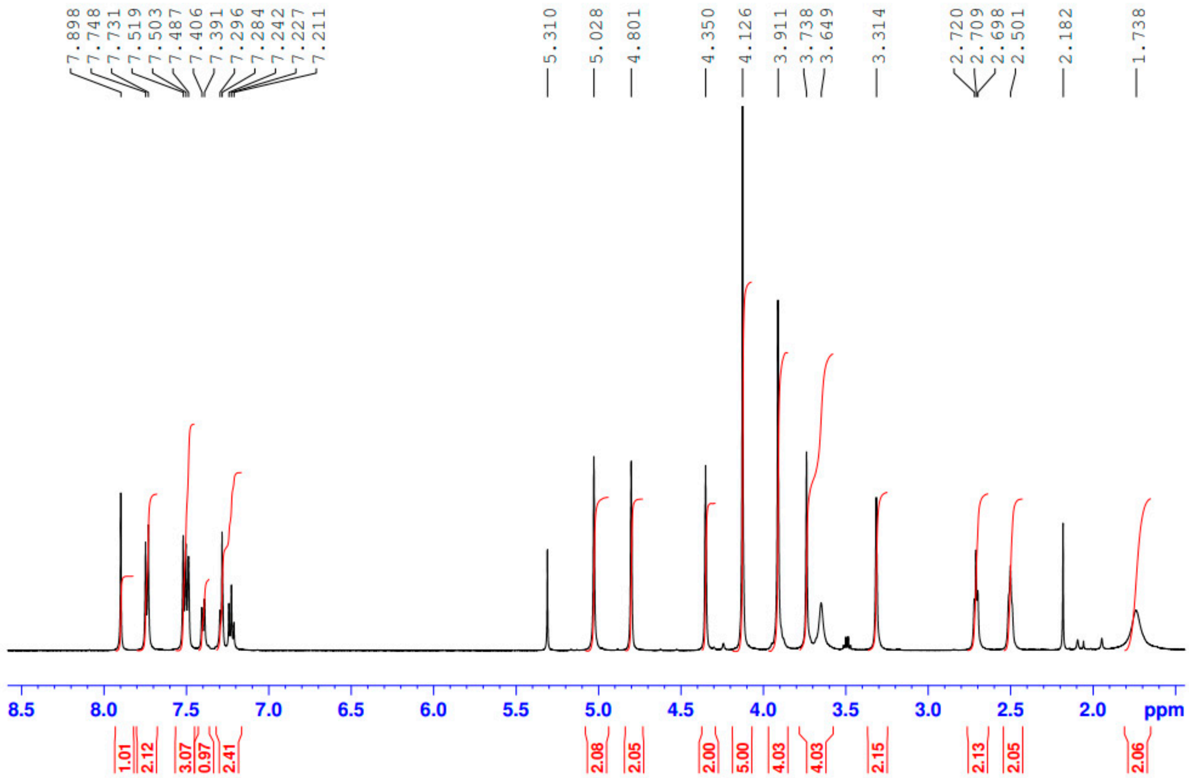

^{13}C-NMR of **2a**

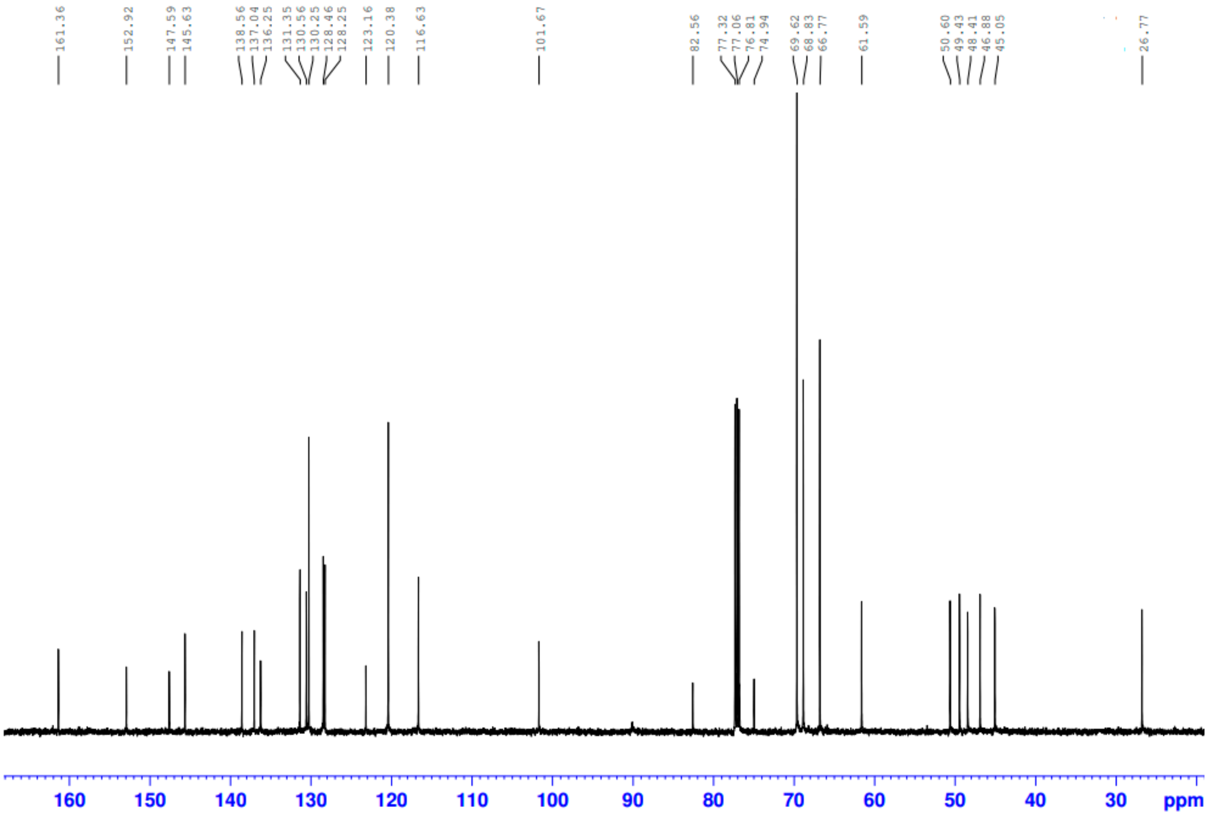

^{1}H-NMR of **2b**

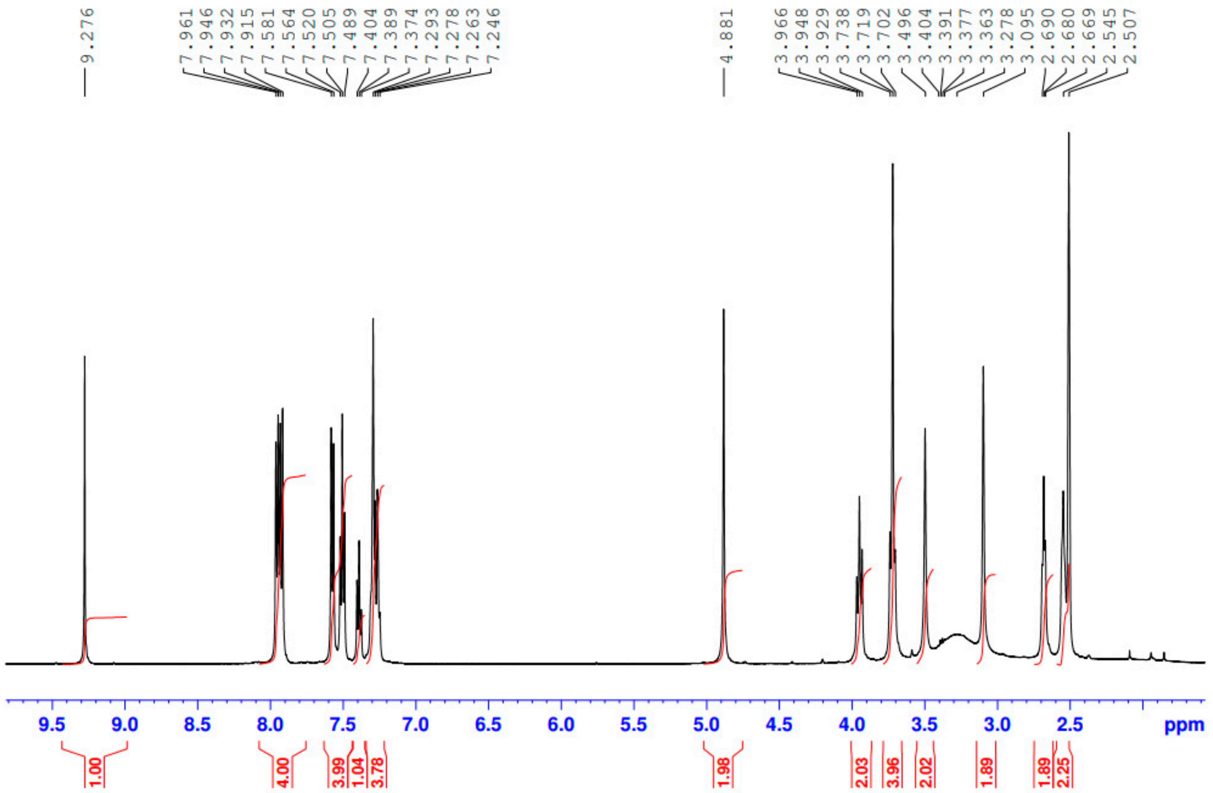

^{13}C-NMR of **2b**

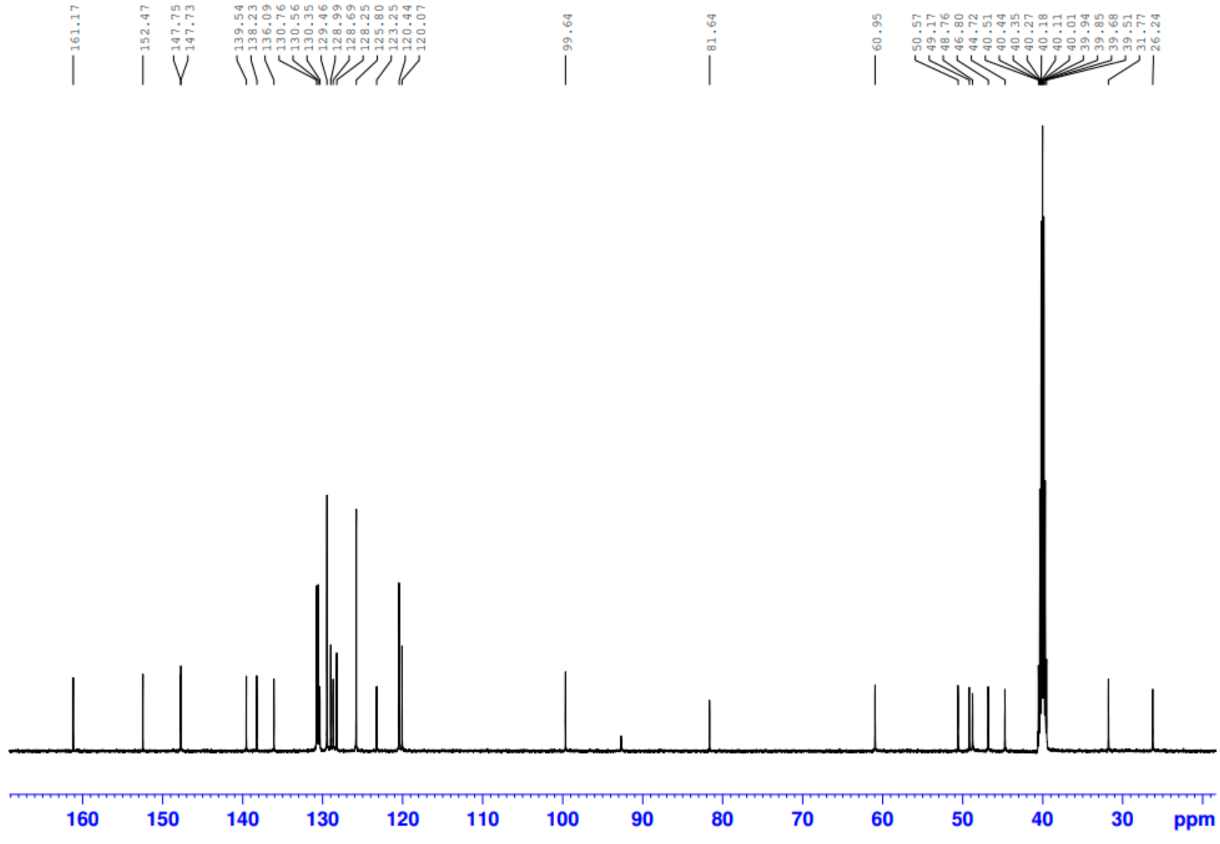

^{1}H-NMR of **2c**

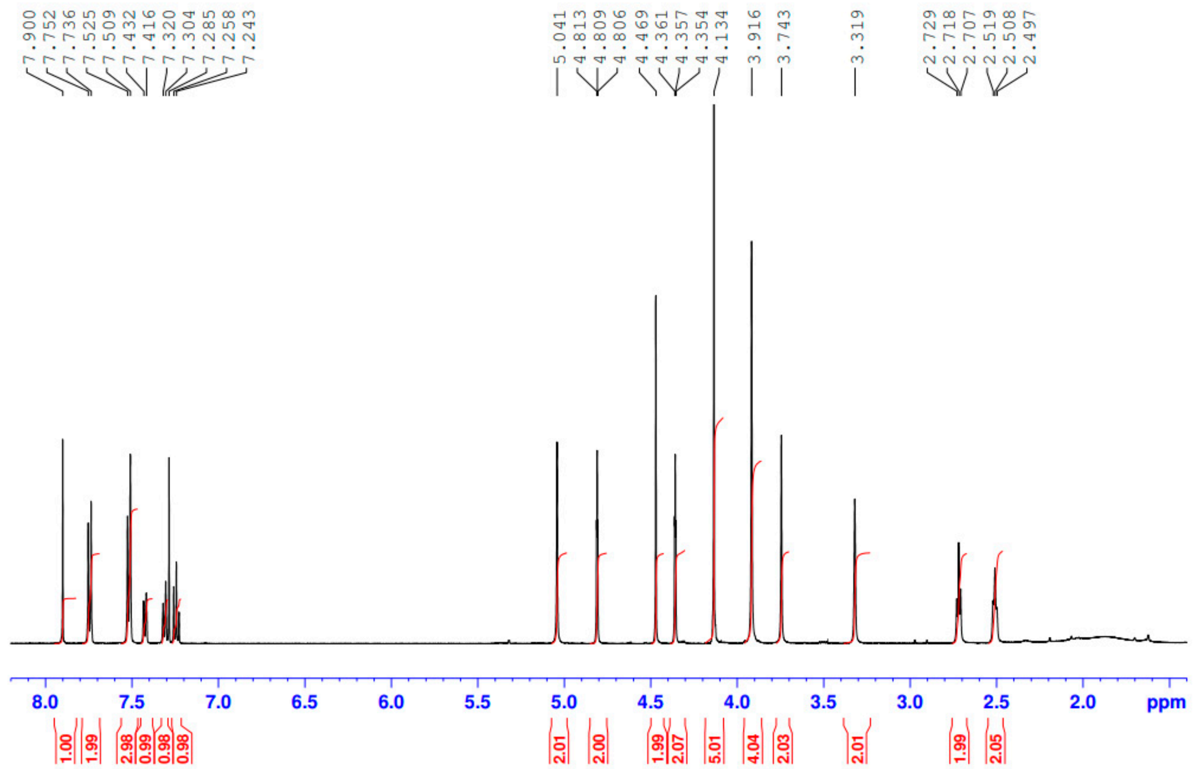

^{13}C-NMR of **2c**

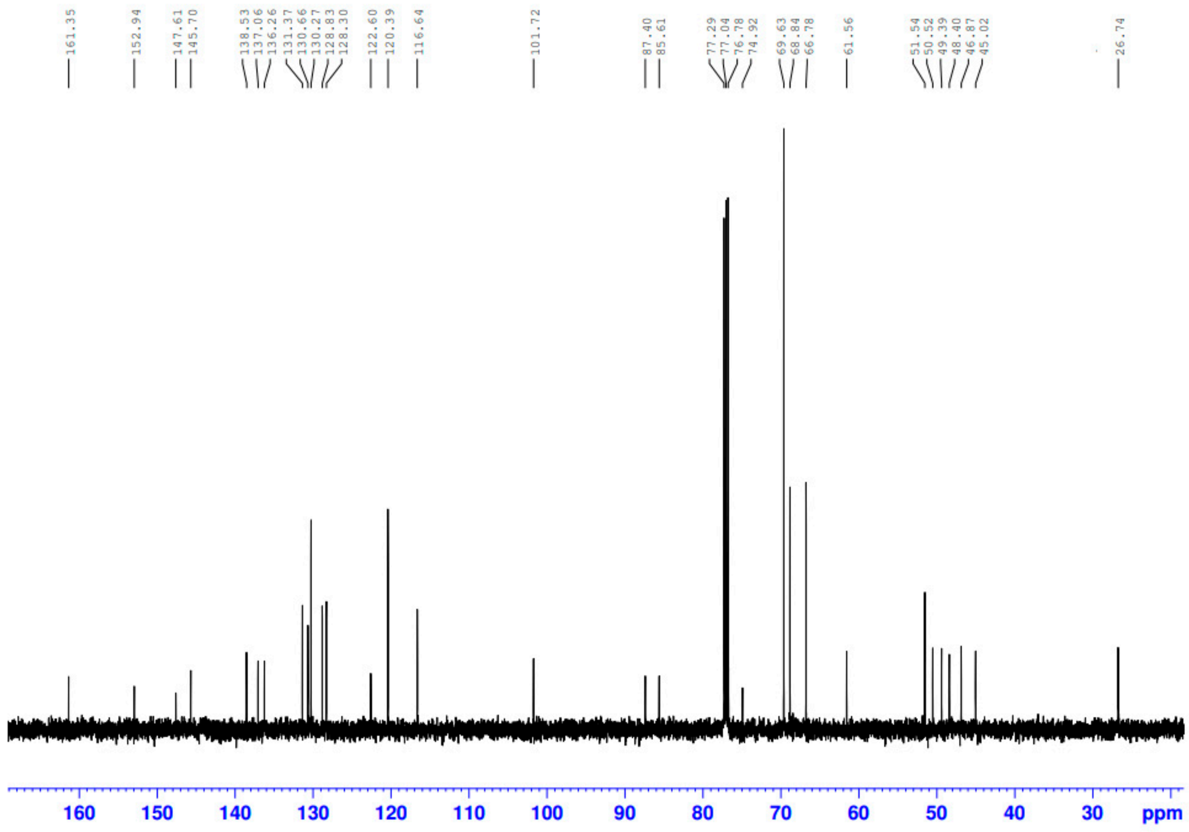

^{1}H-NMR of **2d**

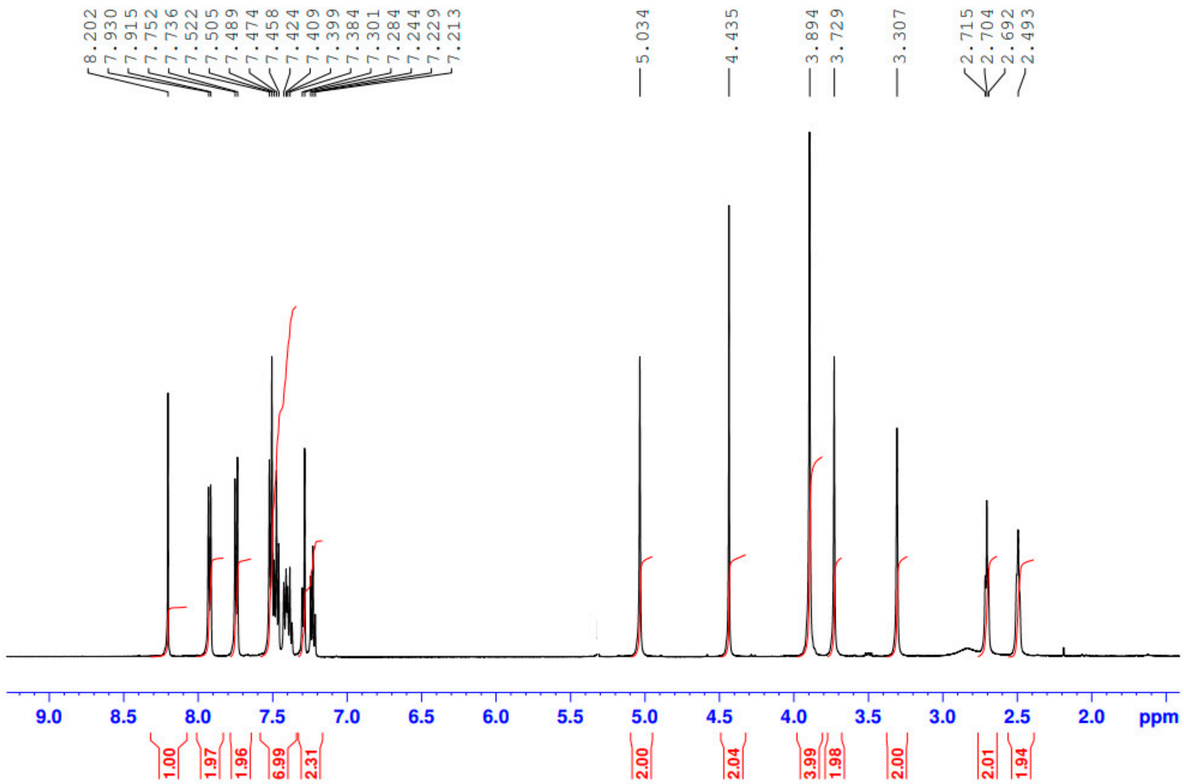

^{13}C-NMR of **2d**

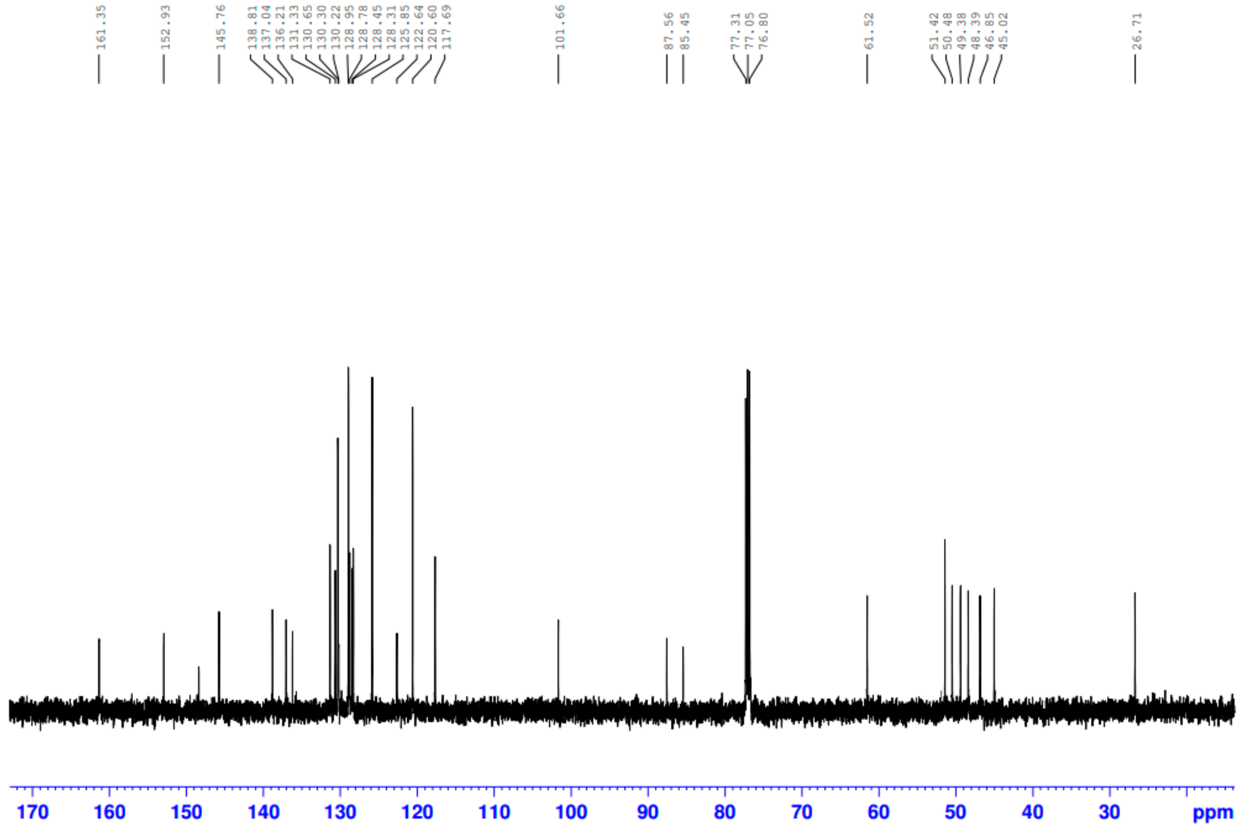

^{1}H-NMR of **3a**

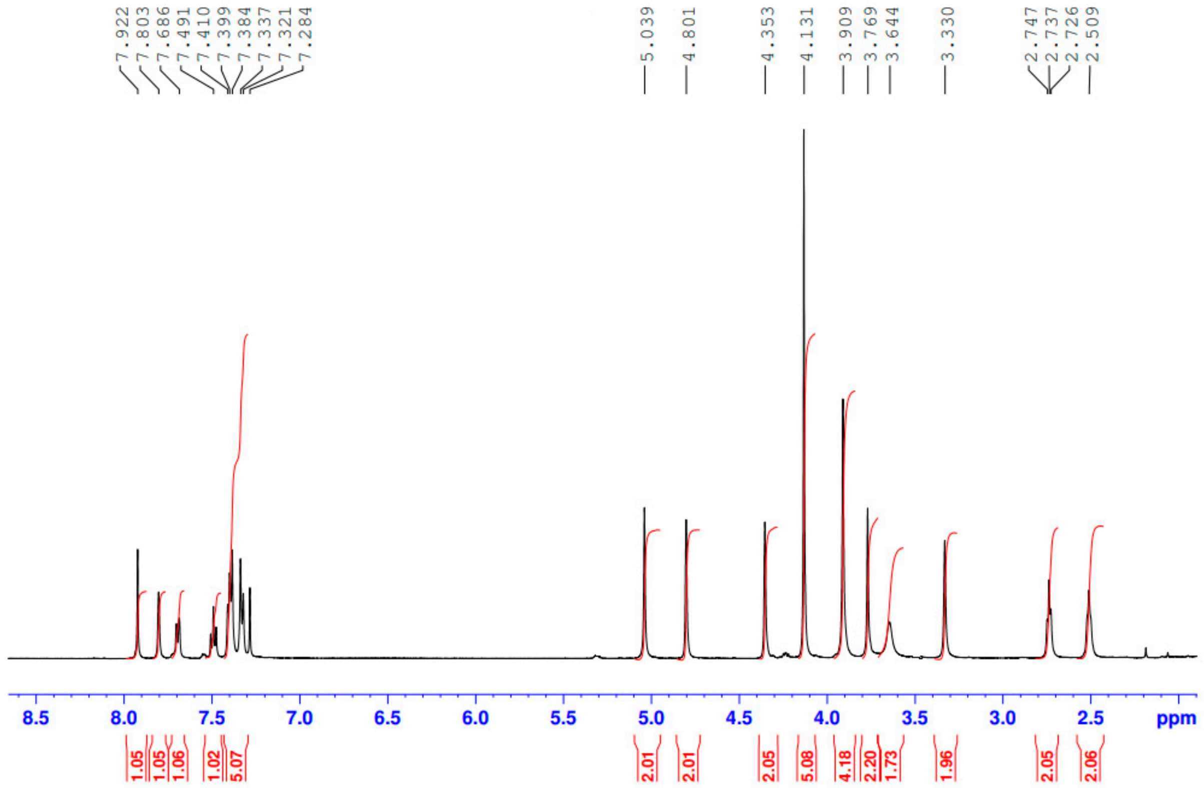

^{13}C-NMR of **3a**

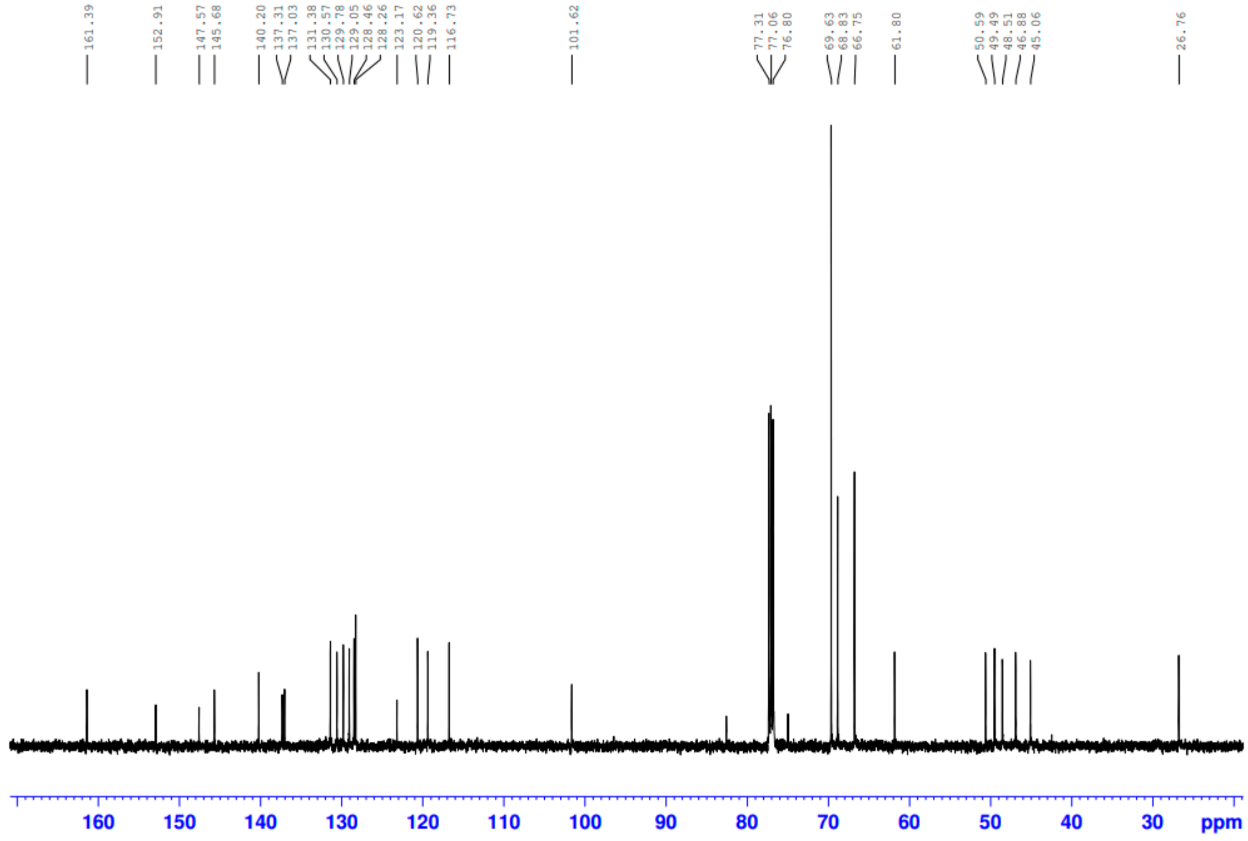

^{1}H-NMR of **3b**

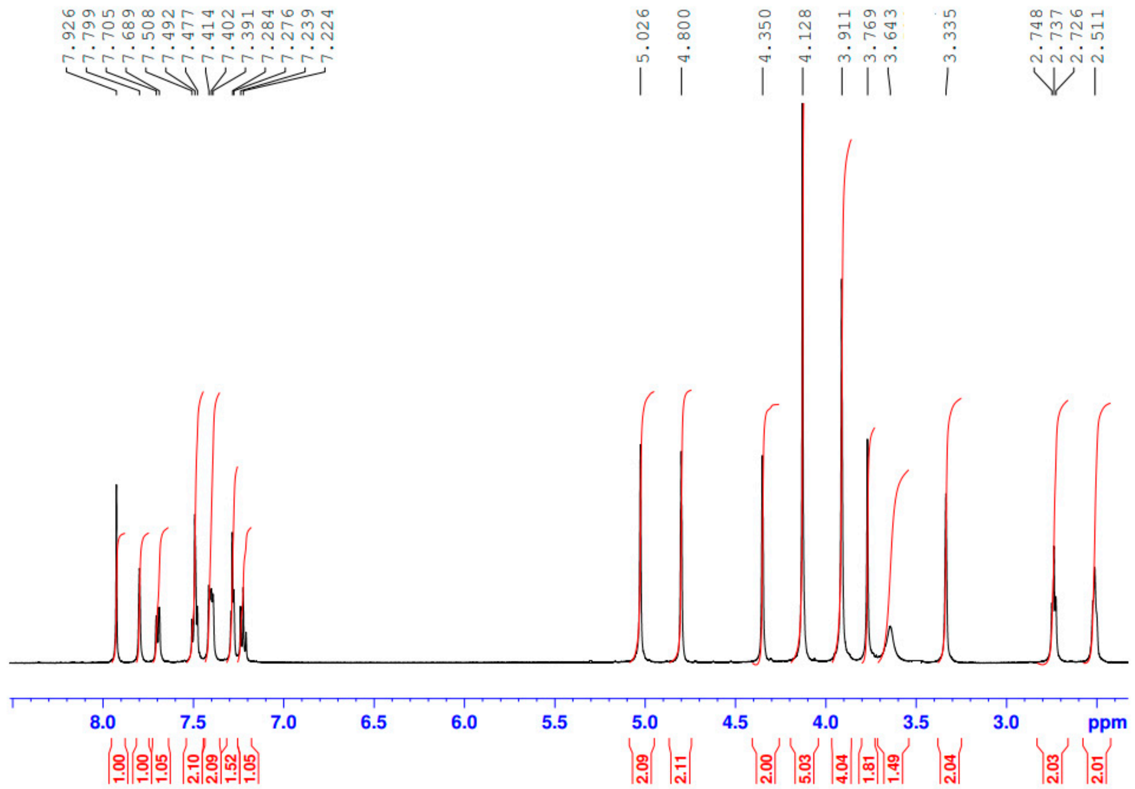

^{13}C-NMR of **3b**

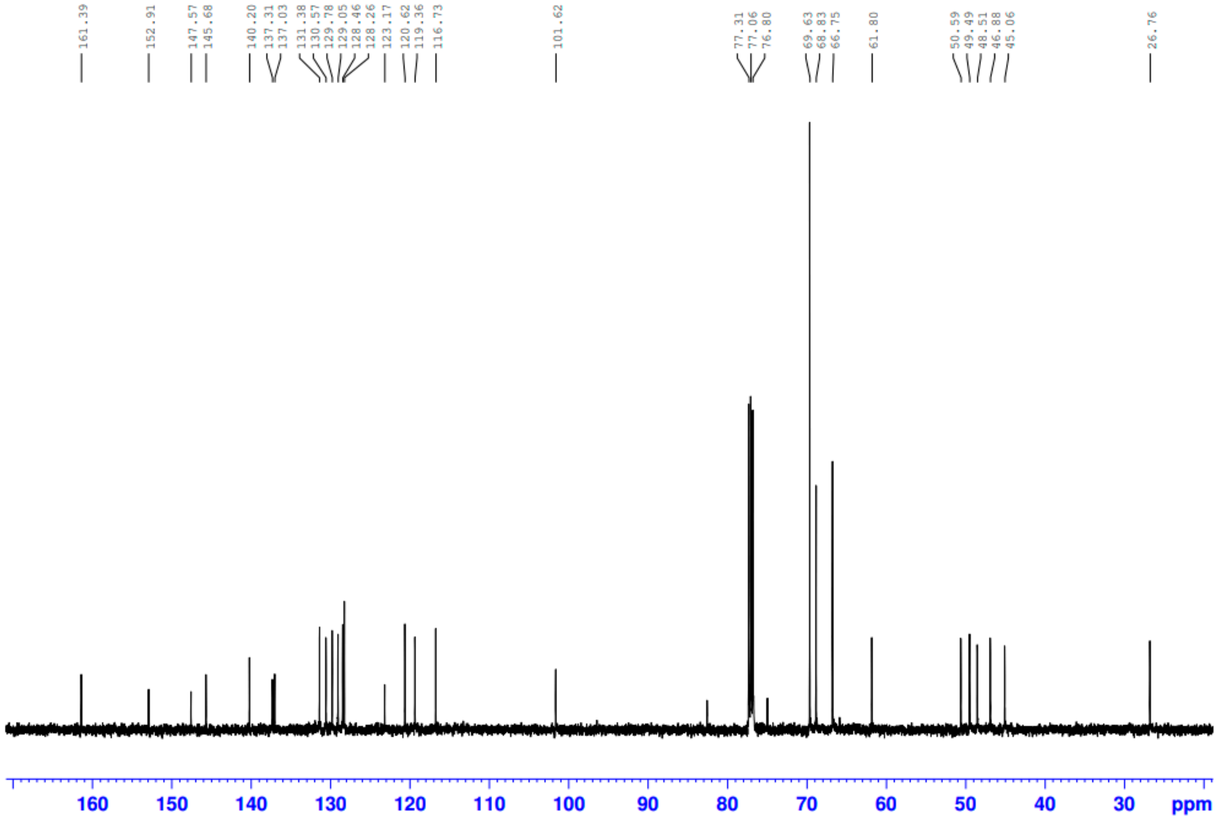

^{1}H-NMR of **3c**

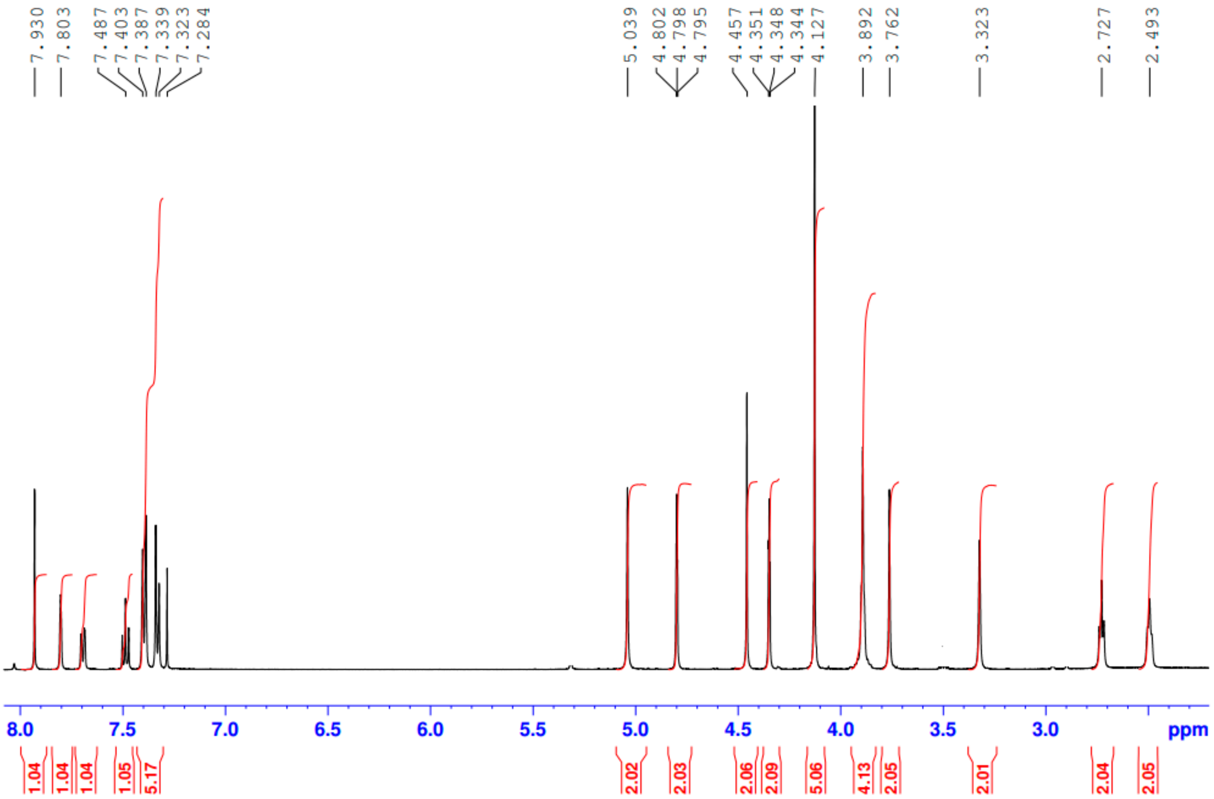

^{13}C-NMR of **3c**

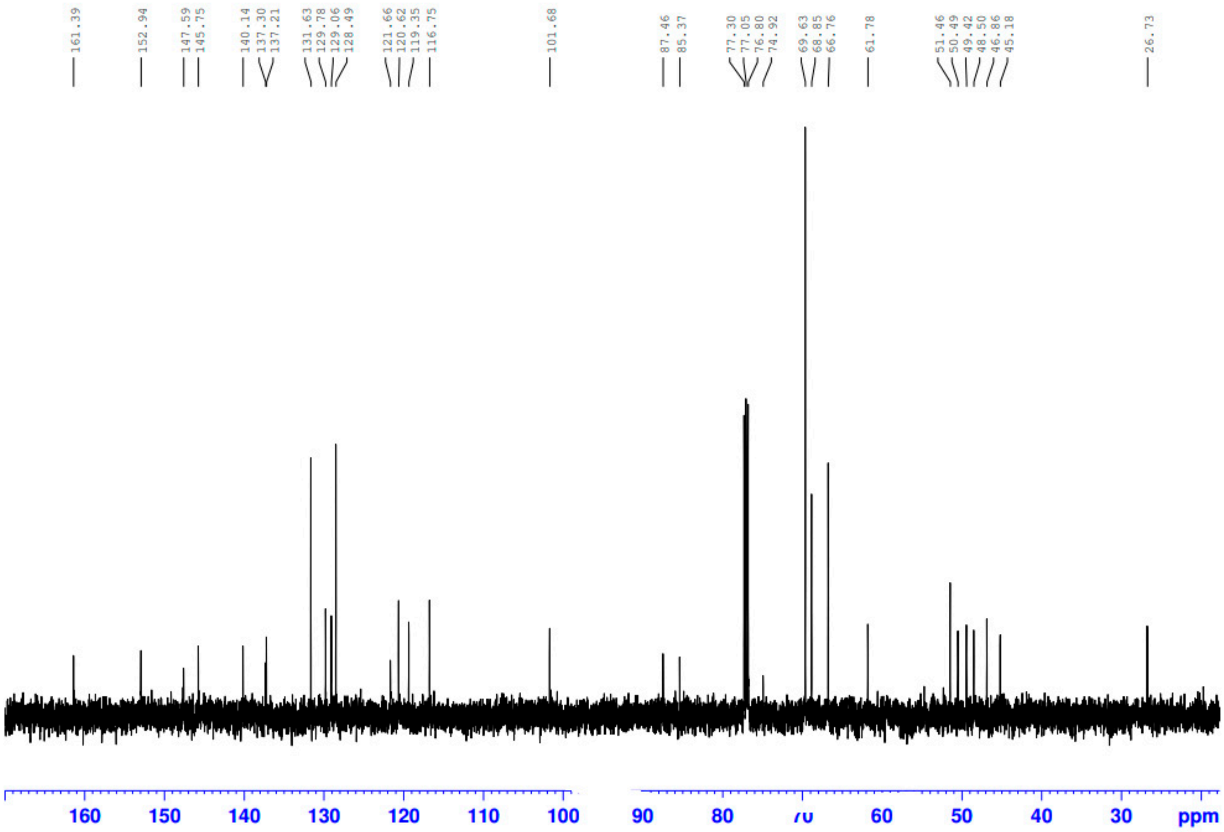

^{1}H-NMR of **3d**

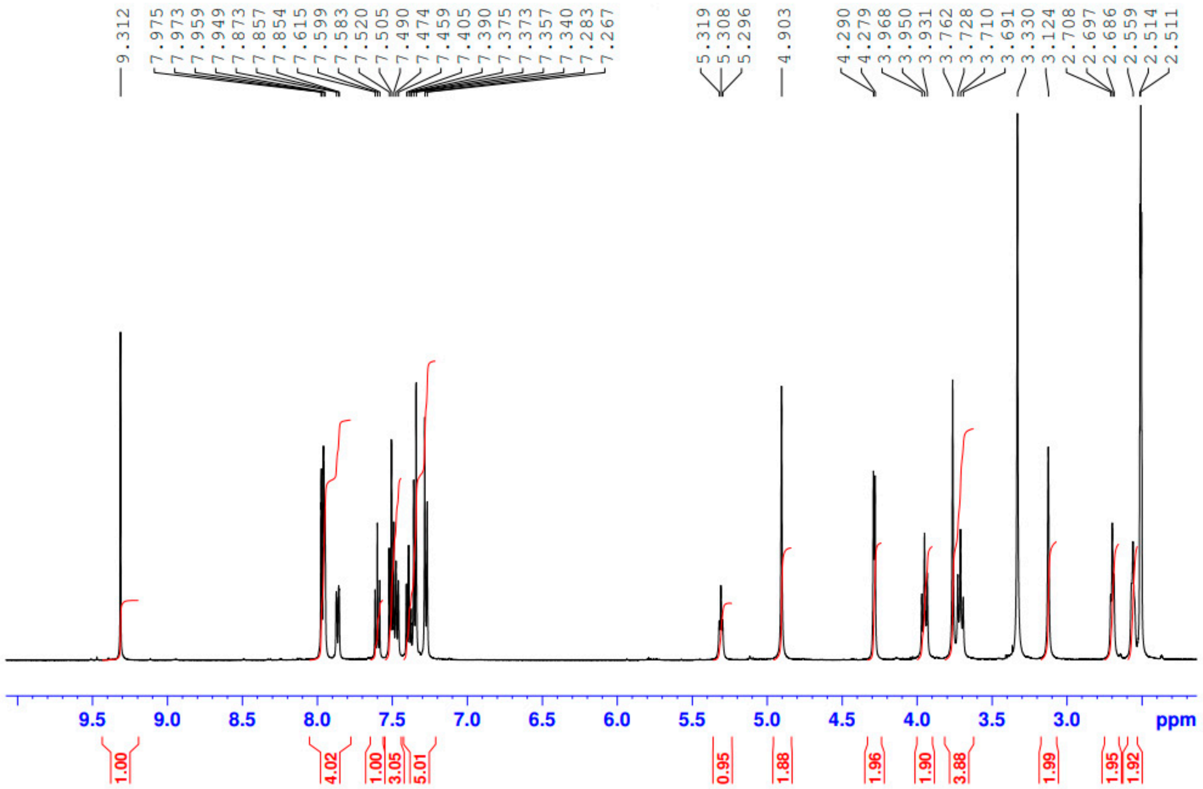

^{13}C-NMR of **3d**

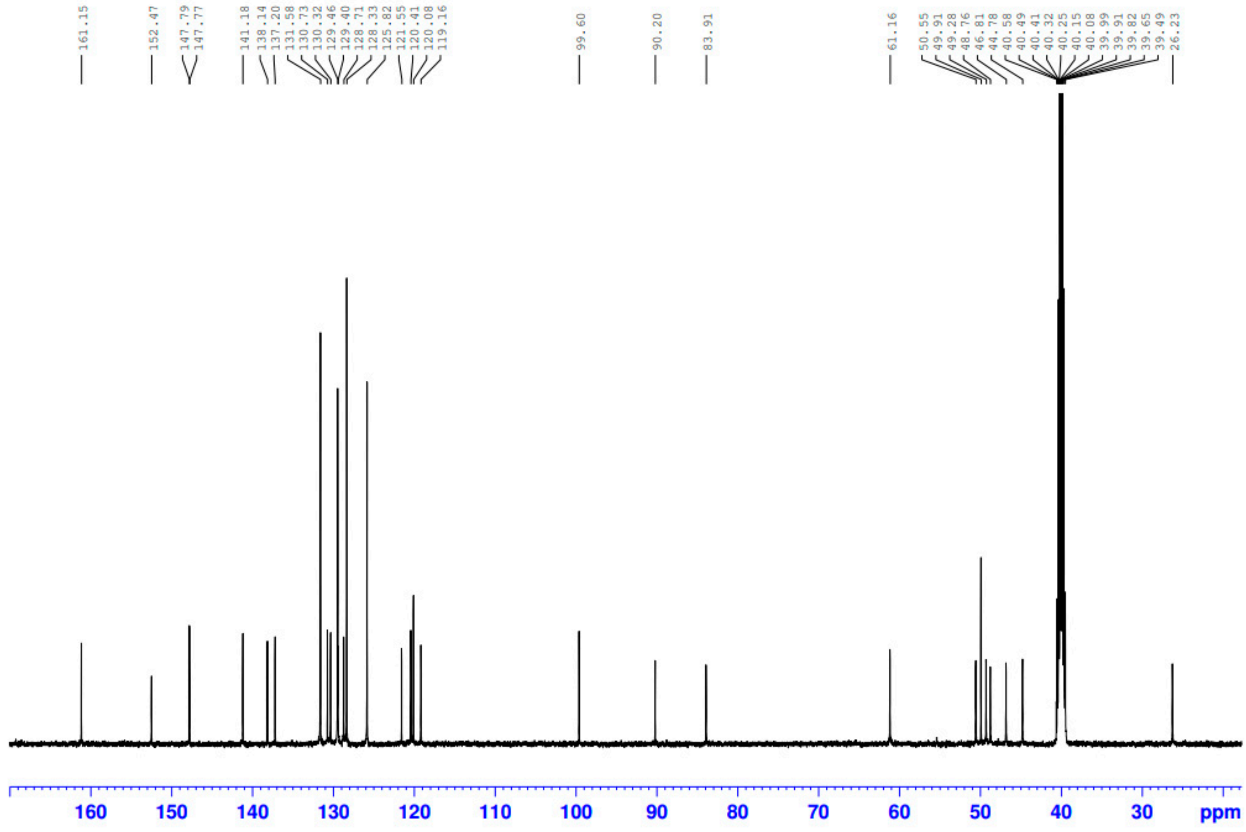

^{1}H-NMR of **4a**

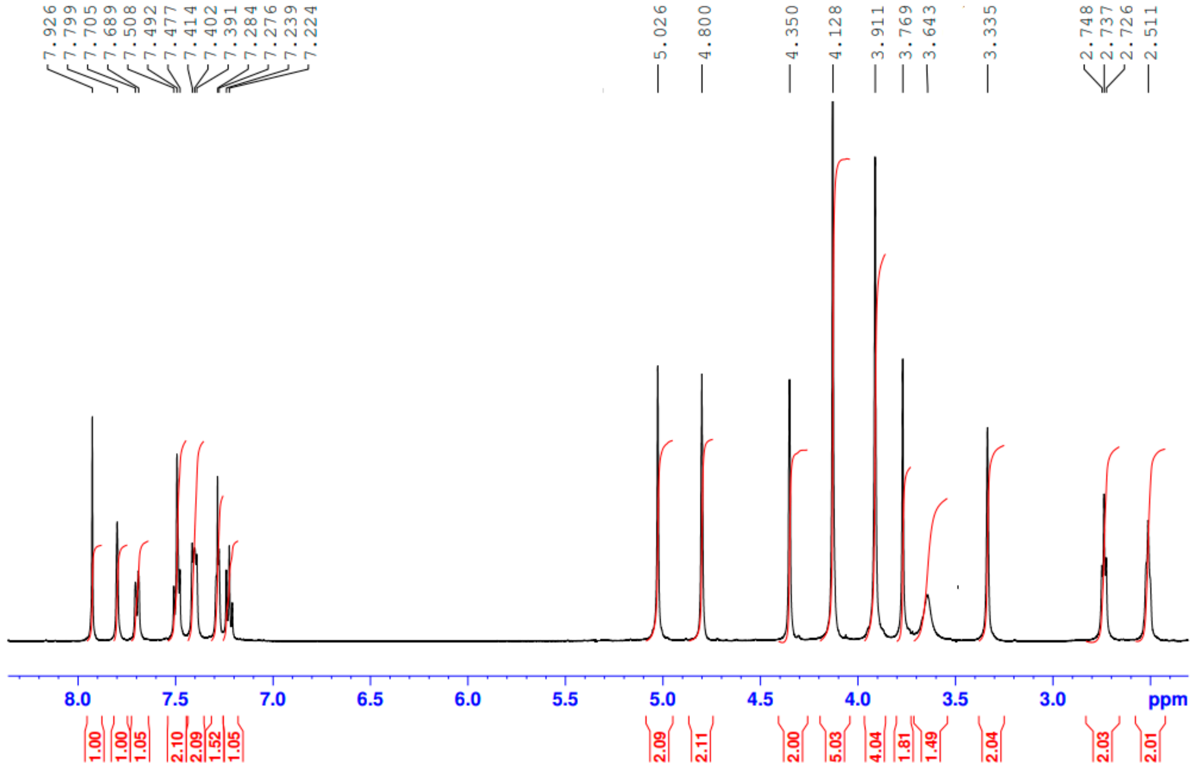

^{13}C-NMR of **4a**

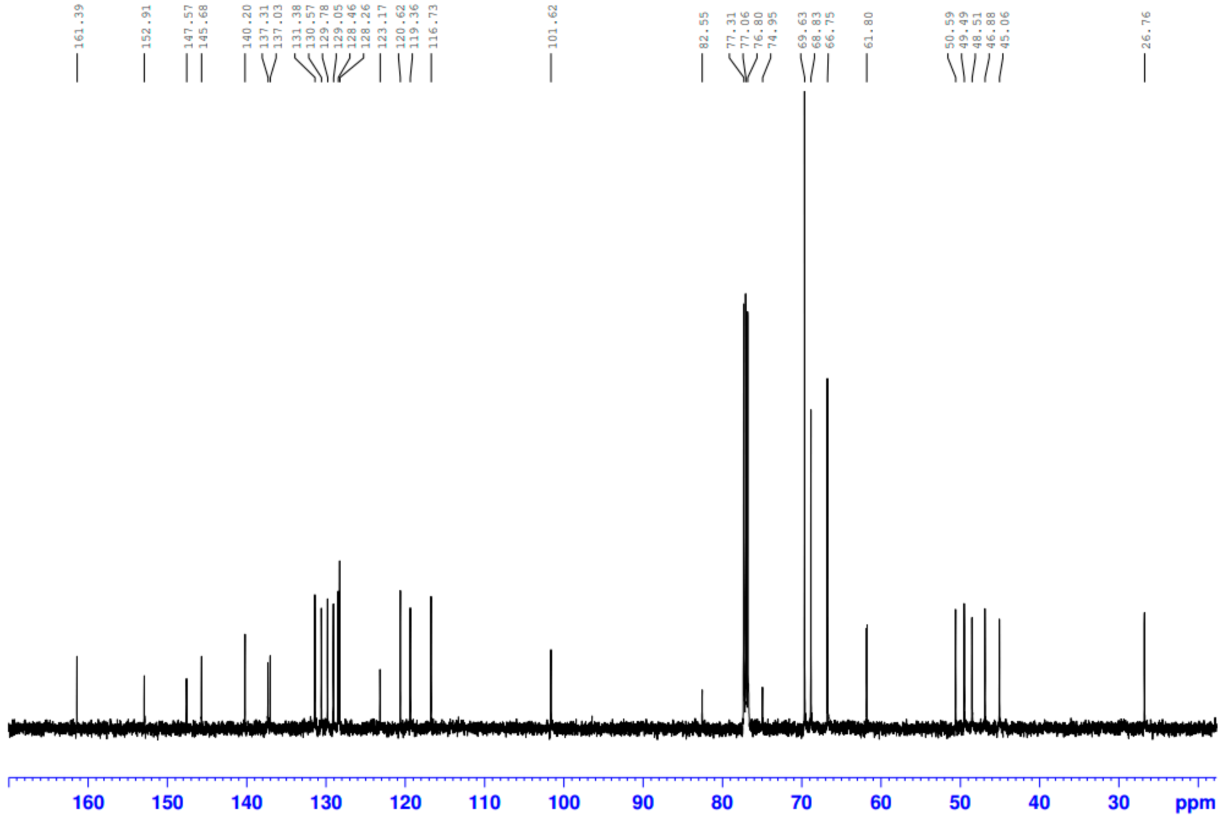

^{1}H-NMR of **4b**

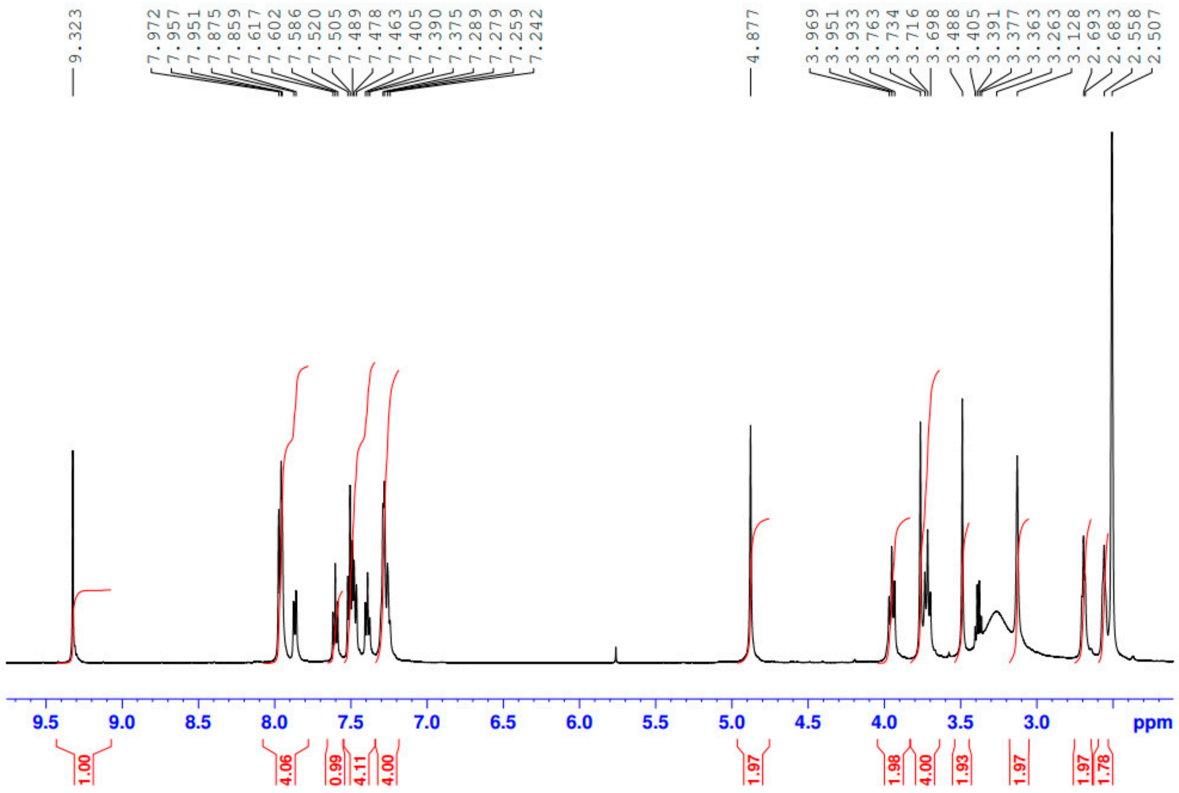

^{13}C-NMR of **4b**

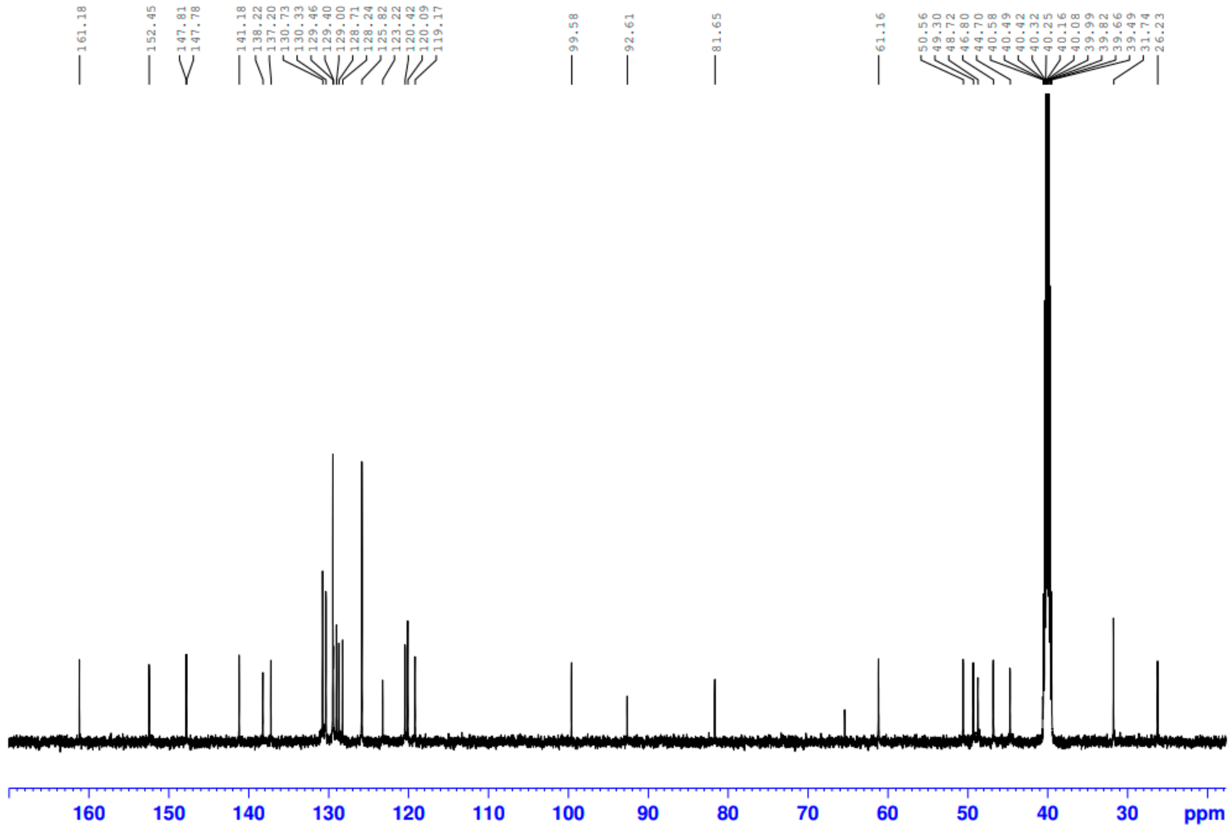

¹H-NMR of **4c**

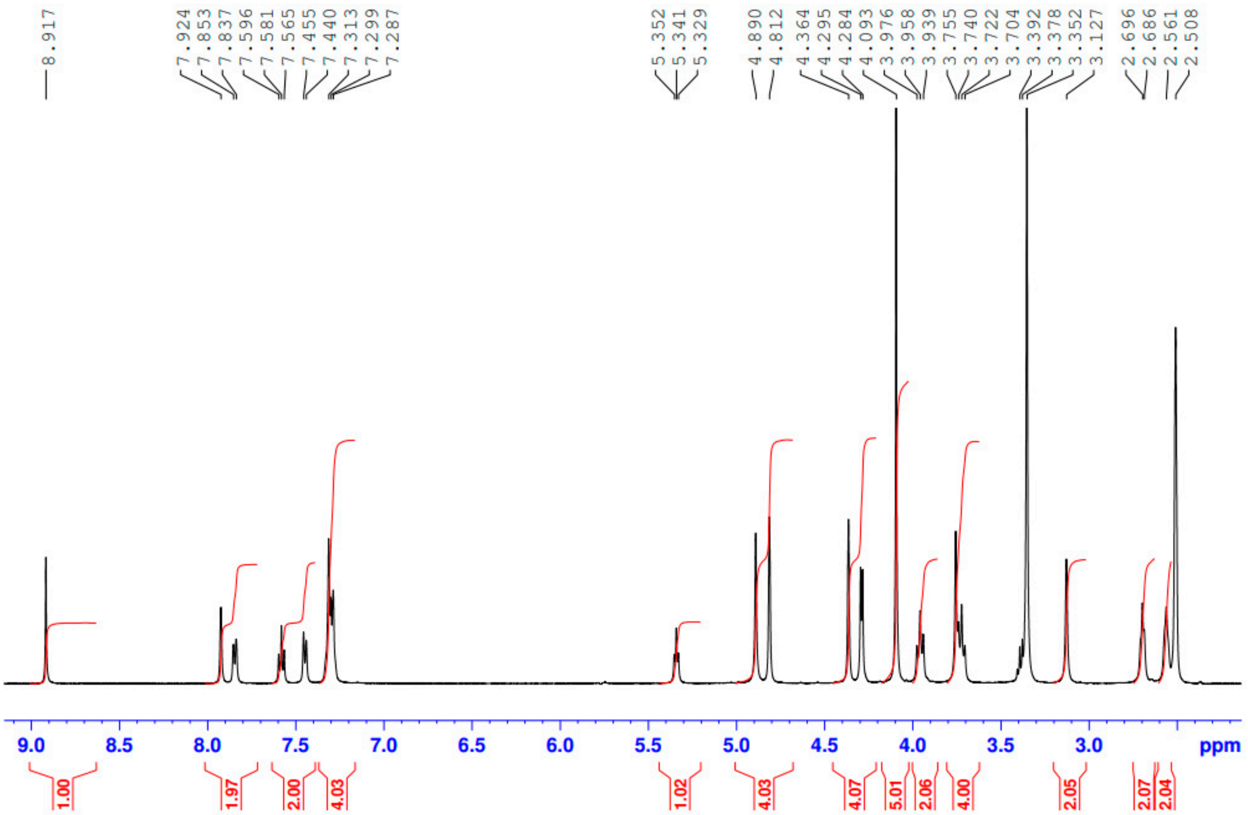

¹³C-NMR of **4c**

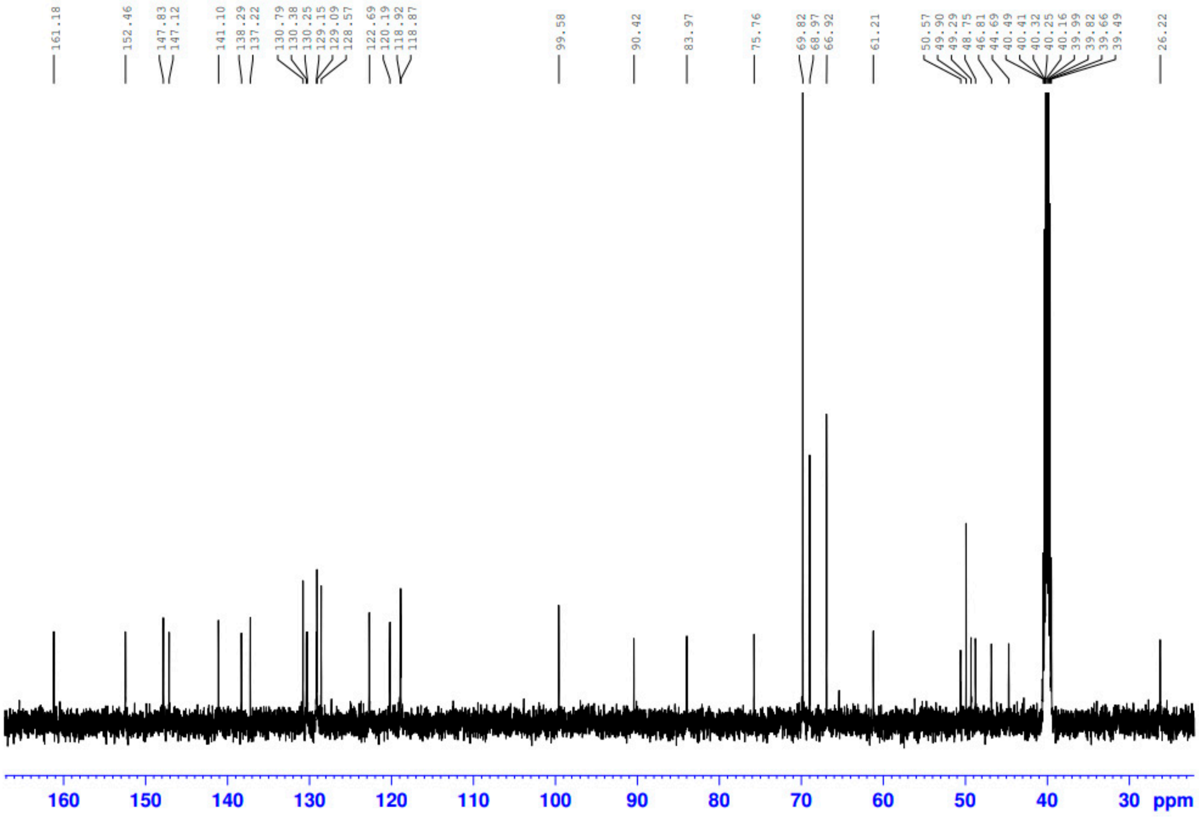

^{1}H-NMR of **4d**

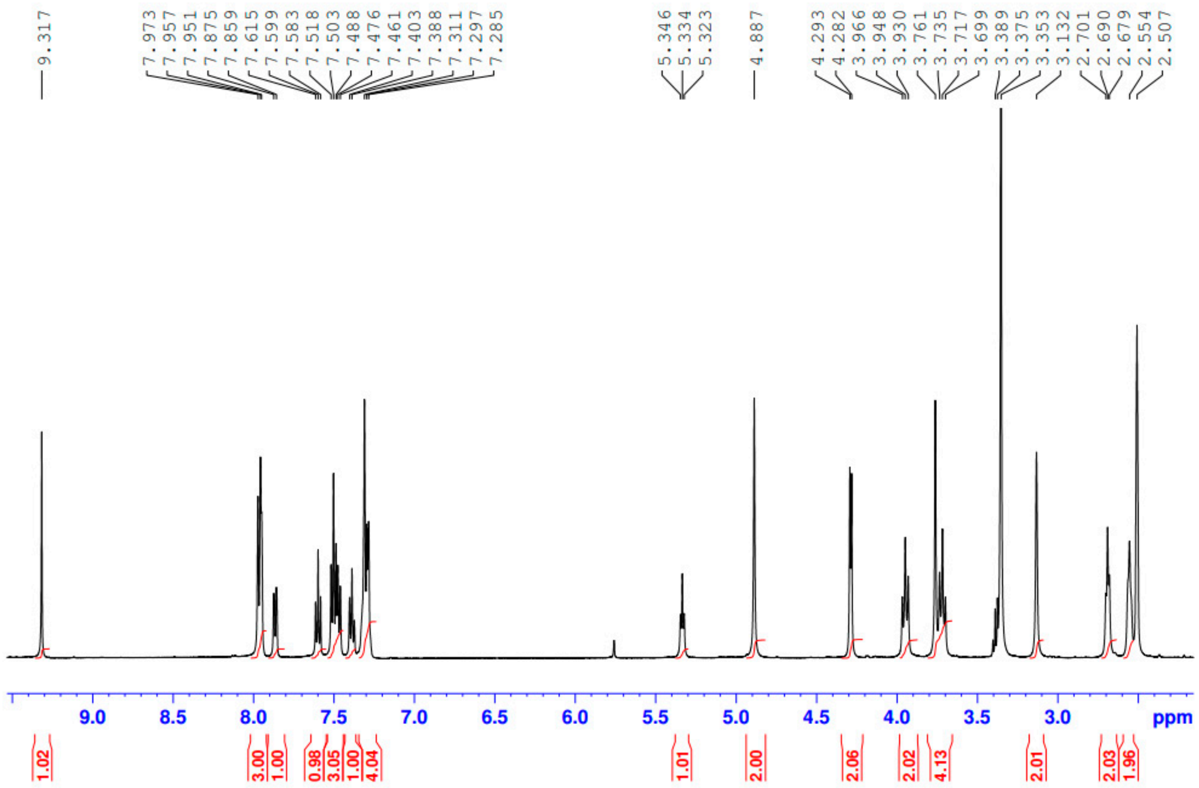

^{13}C-NMR of **4d**

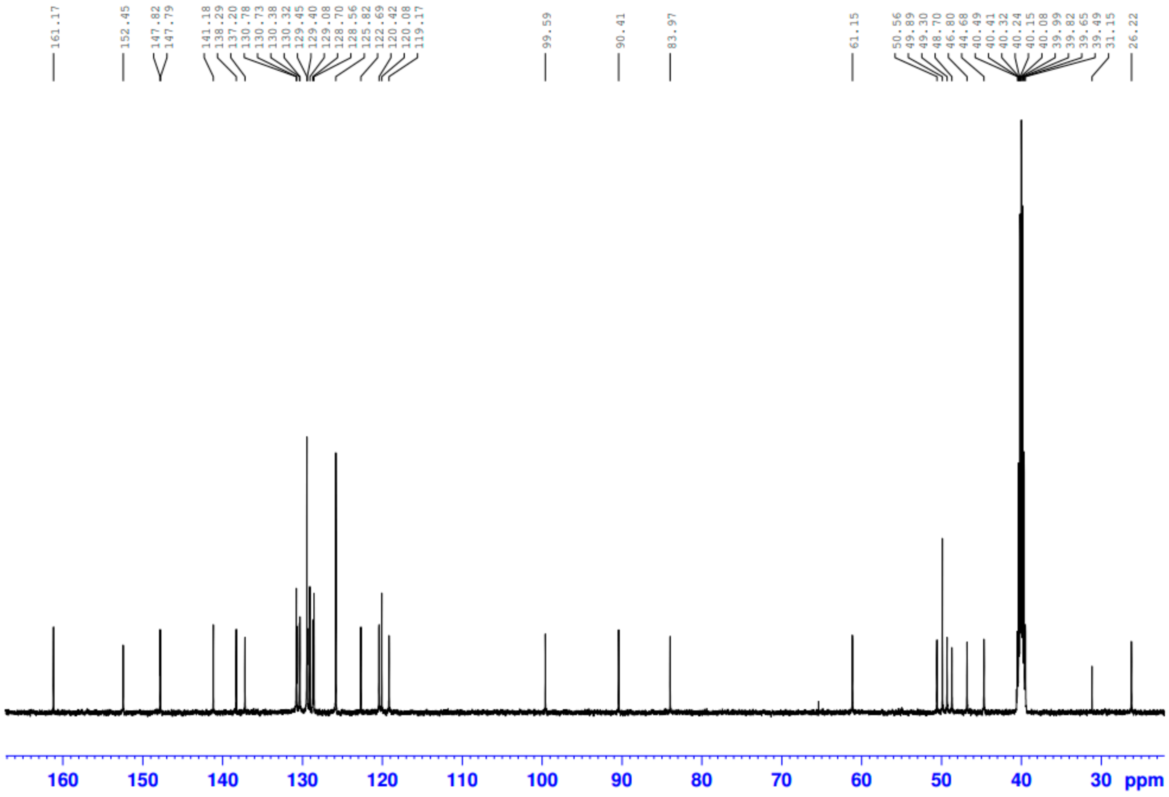

^{1}H-NMR of **23a**

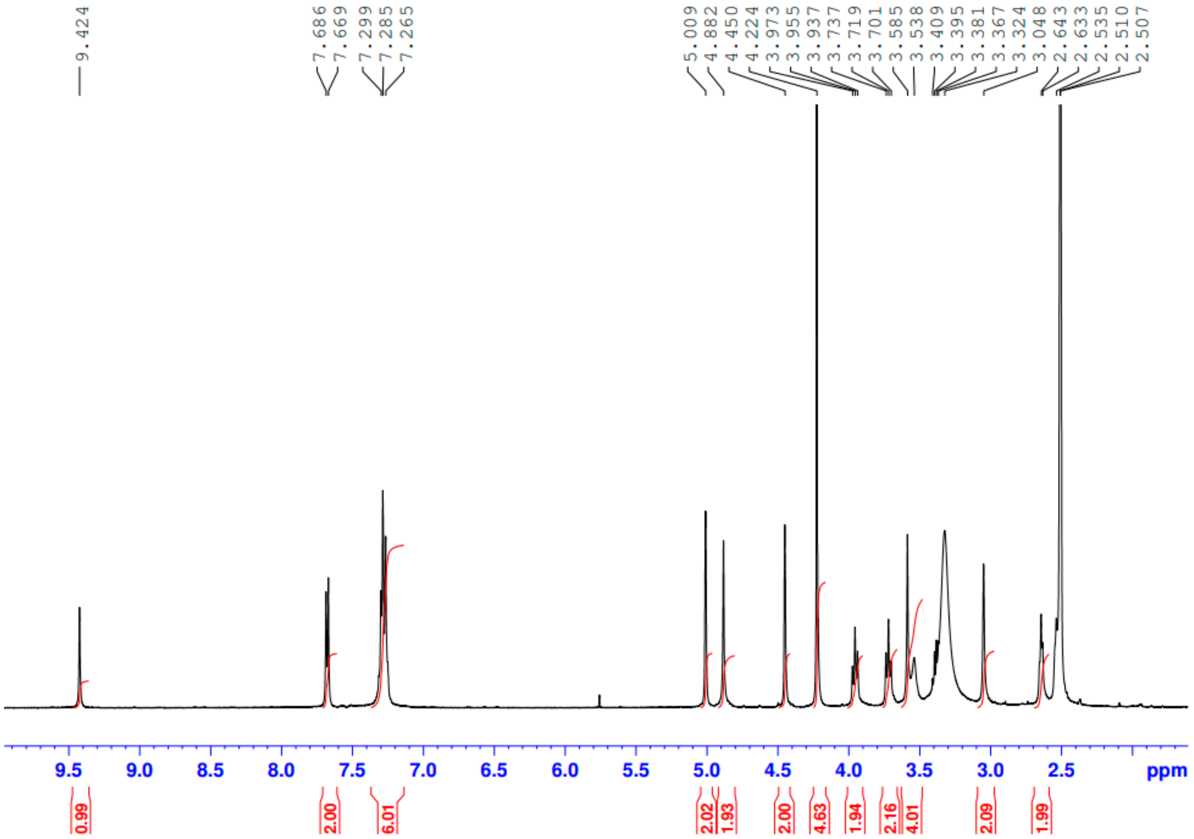

^{13}C-NMR of **23a**

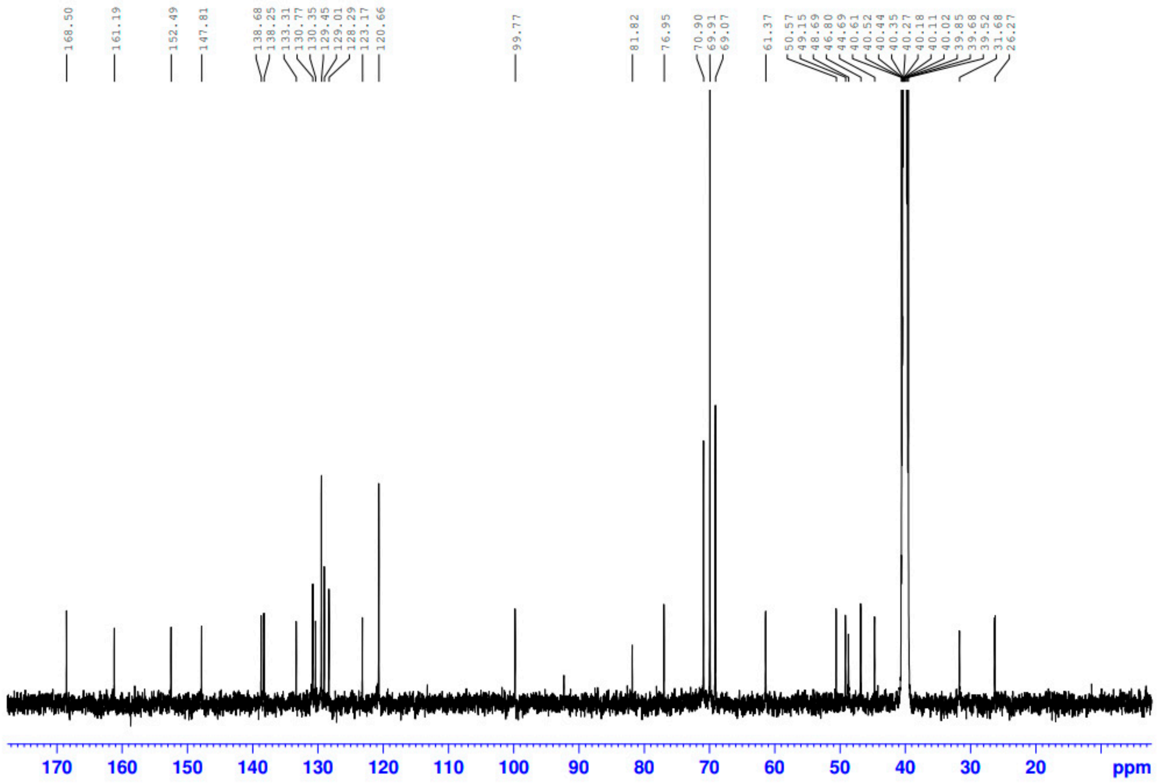

^{1}H-NMR of **23b**

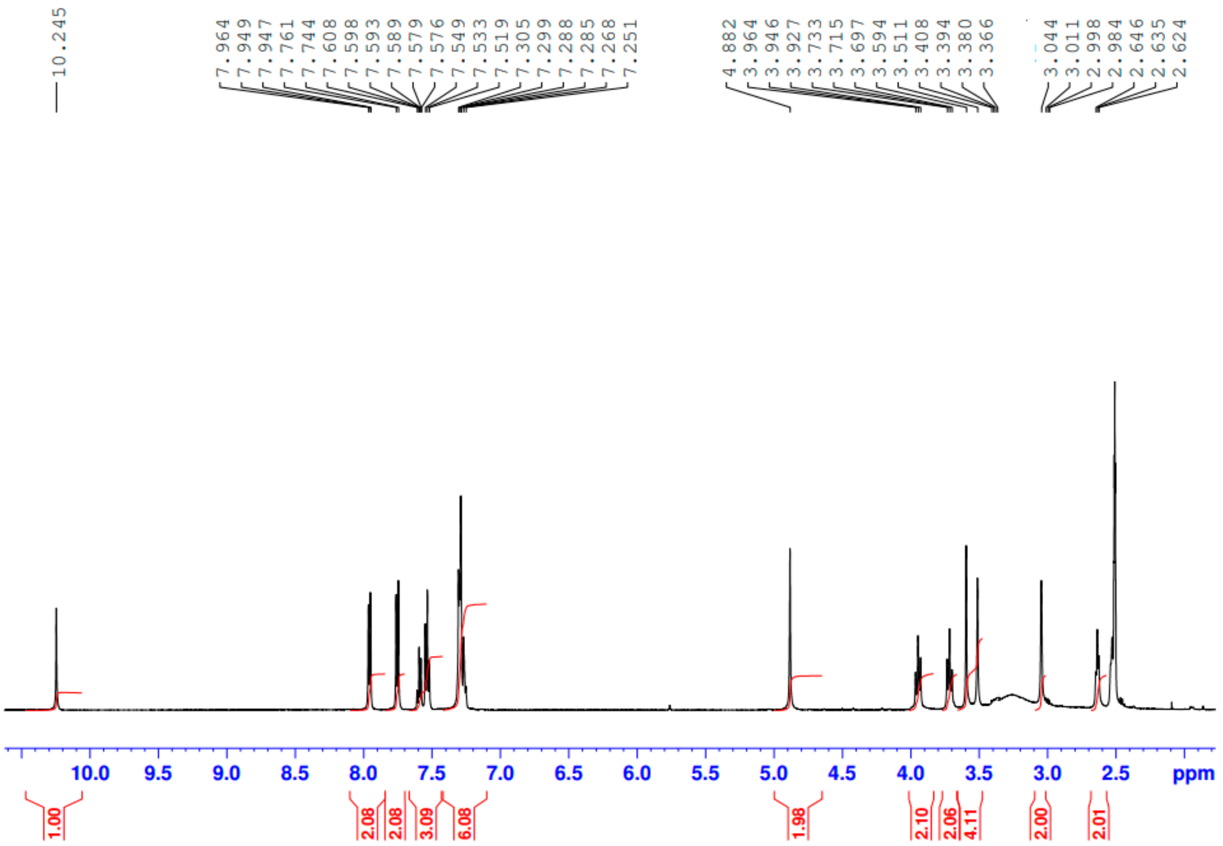

^{13}C-NMR of **23b**

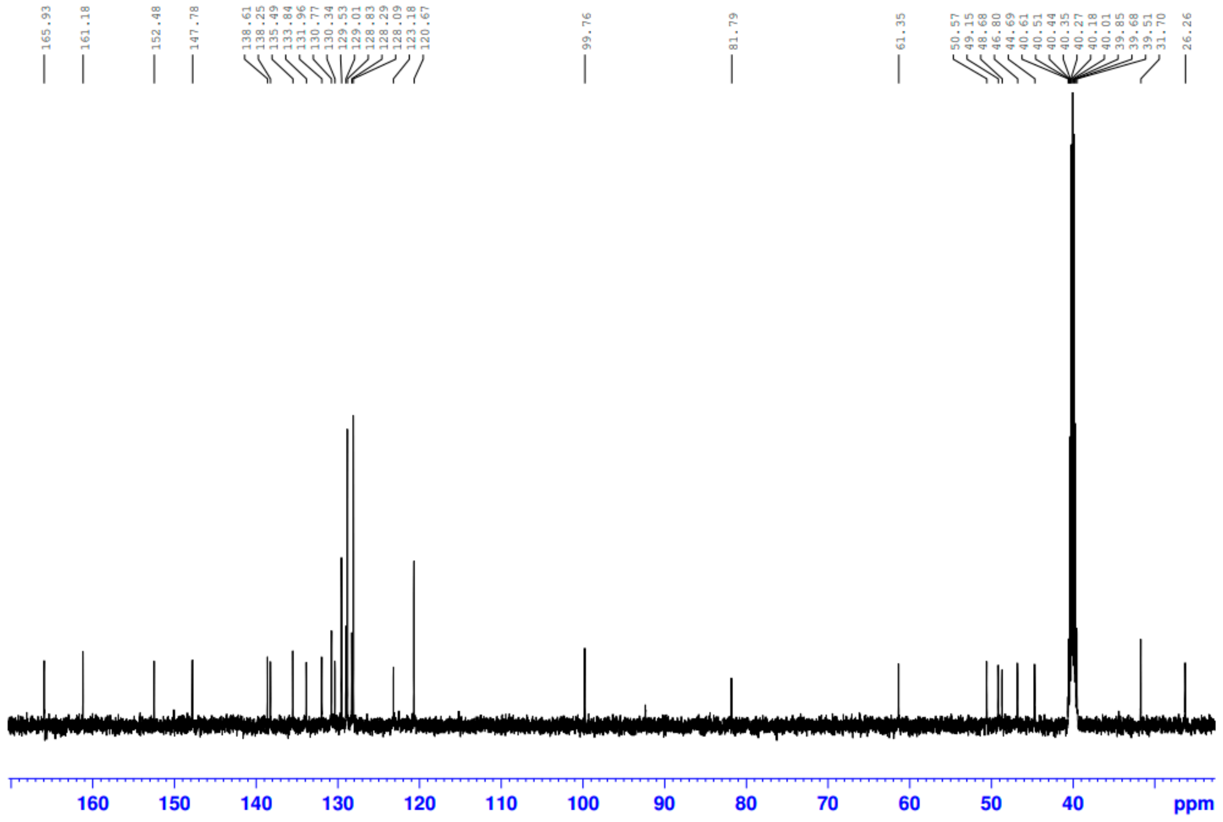

¹H-NMR of **33a**

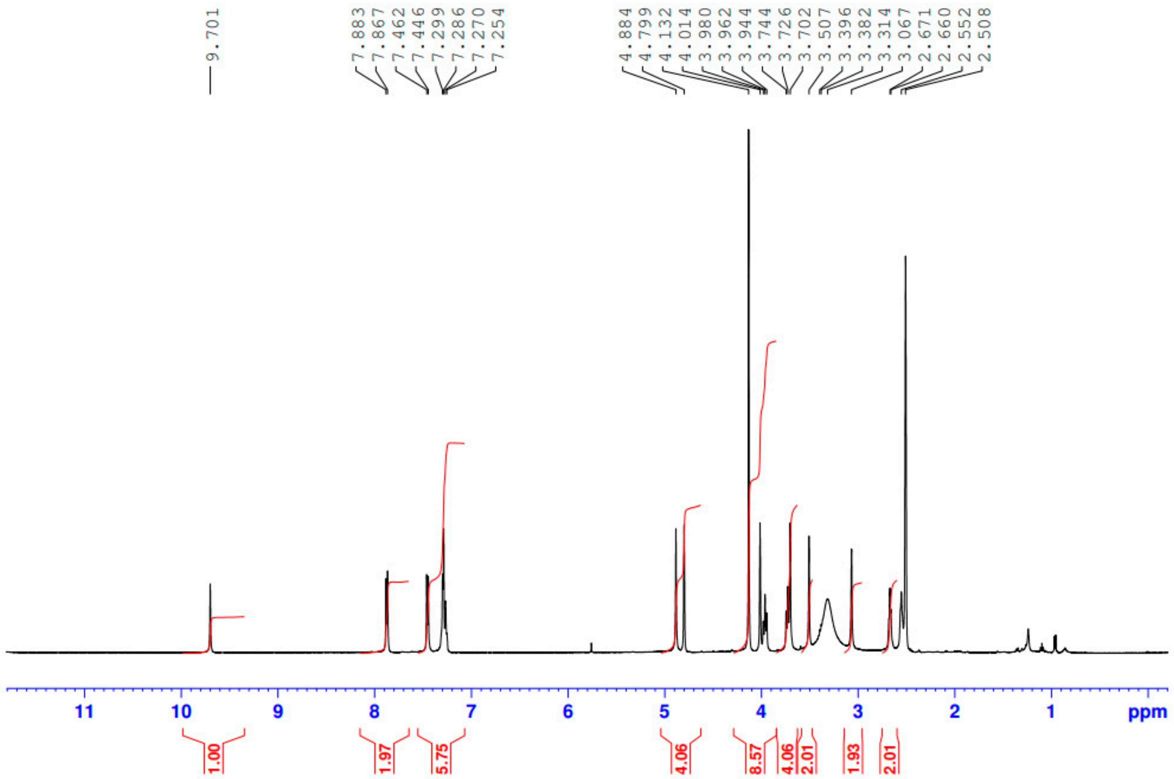

¹³C-NMR of **33a**

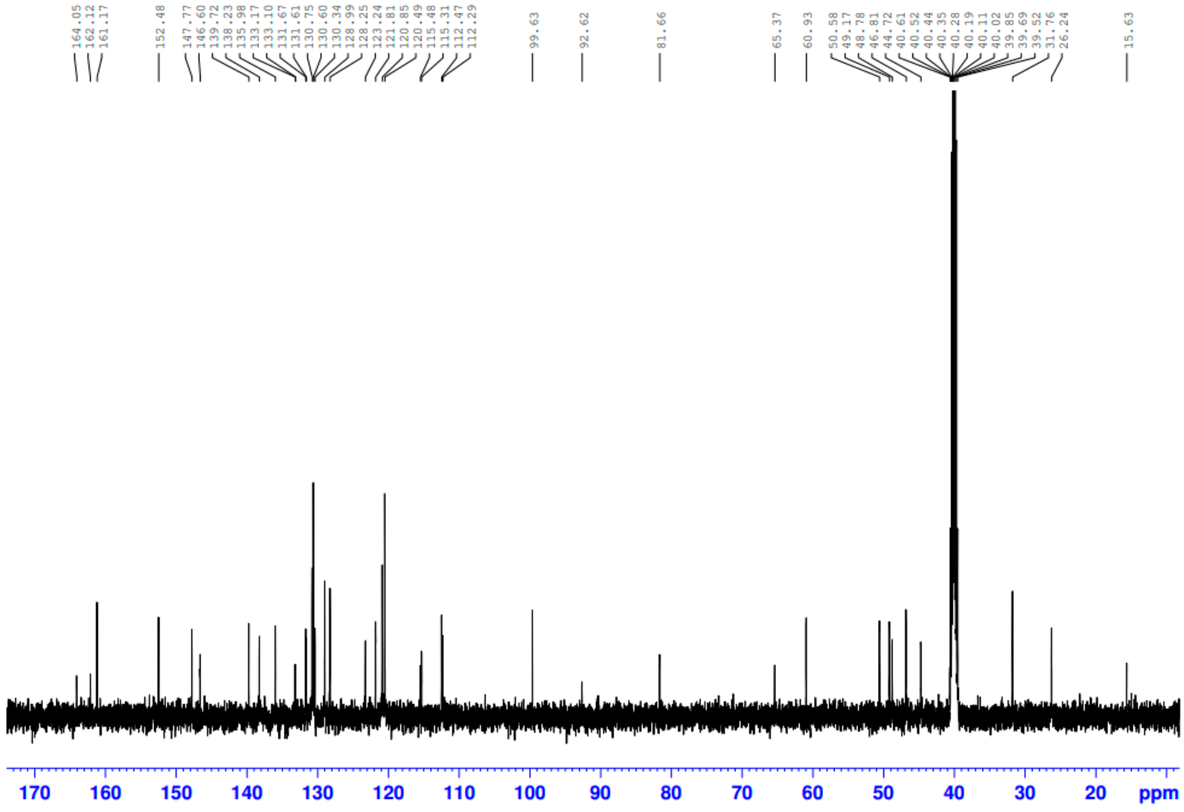

^{1}H-NMR of **33b**

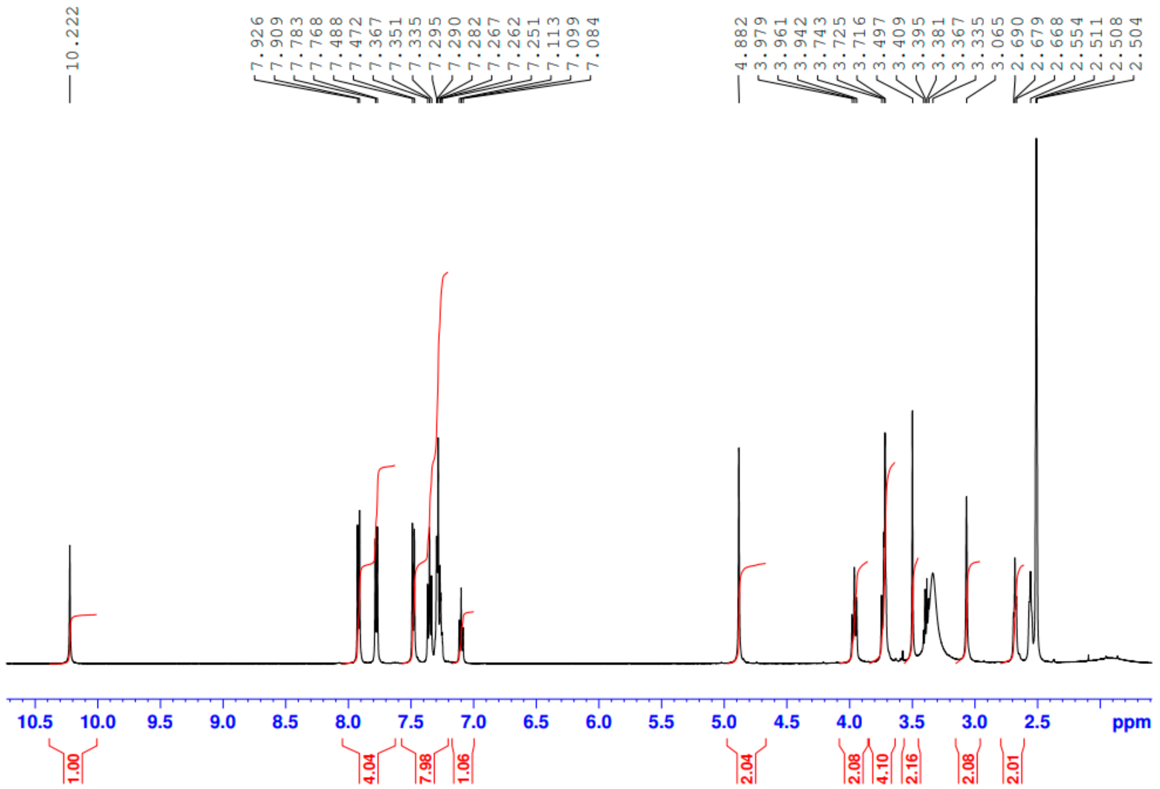

^{13}C-NMR of **33b**

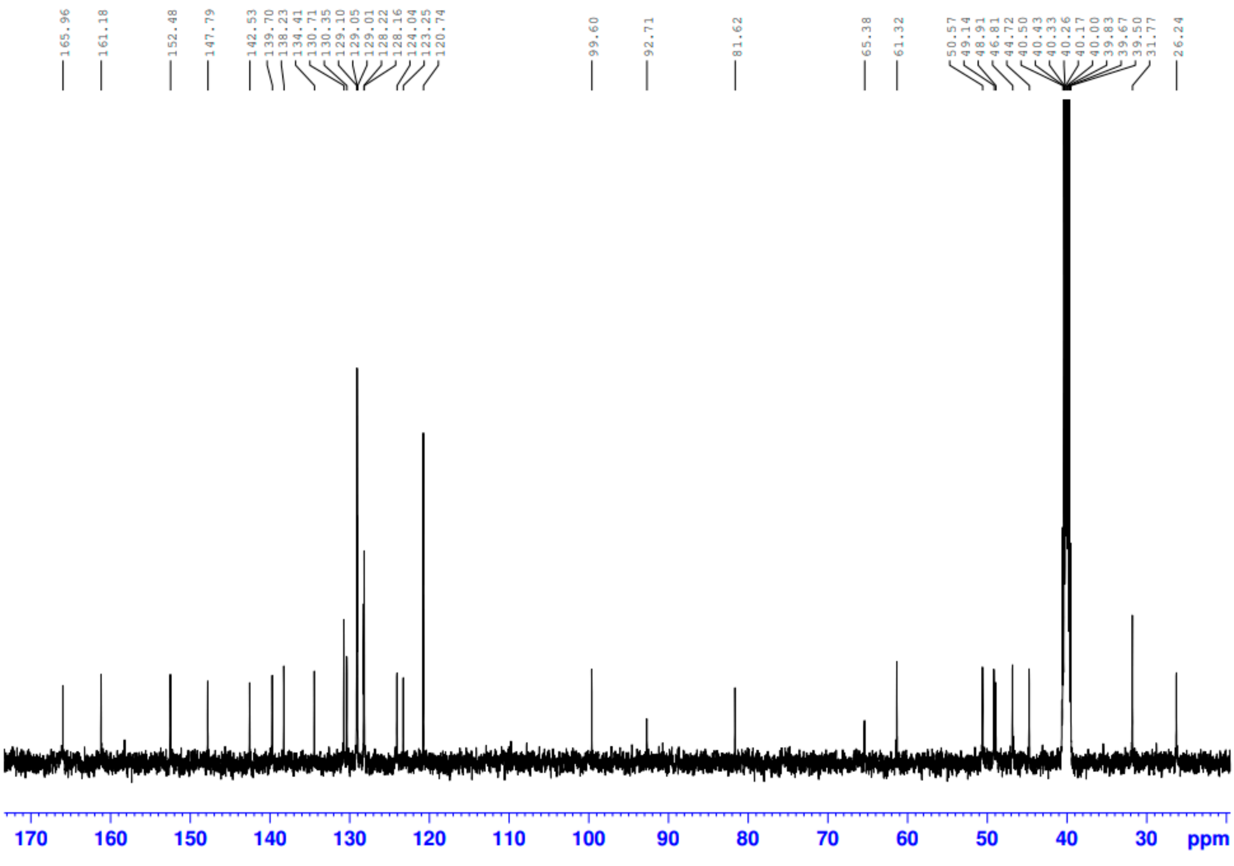

^{1}H-NMR of **36a**

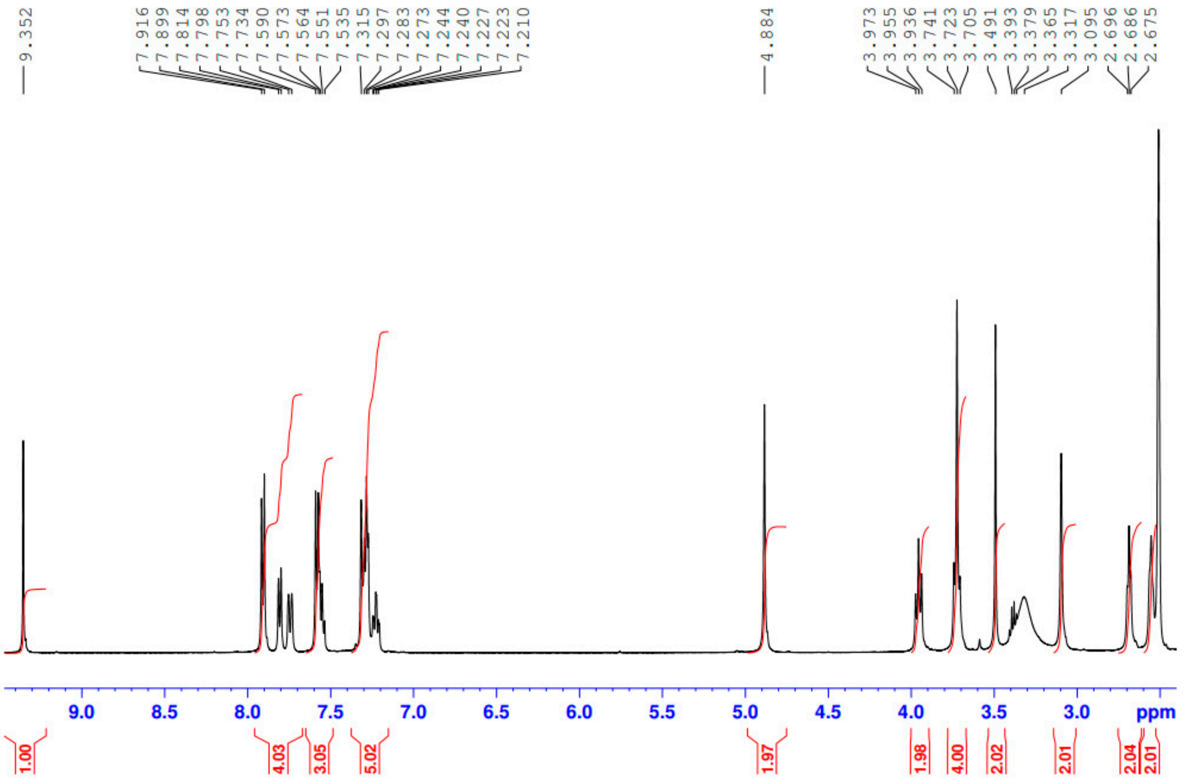

^{13}C-NMR of **36a**

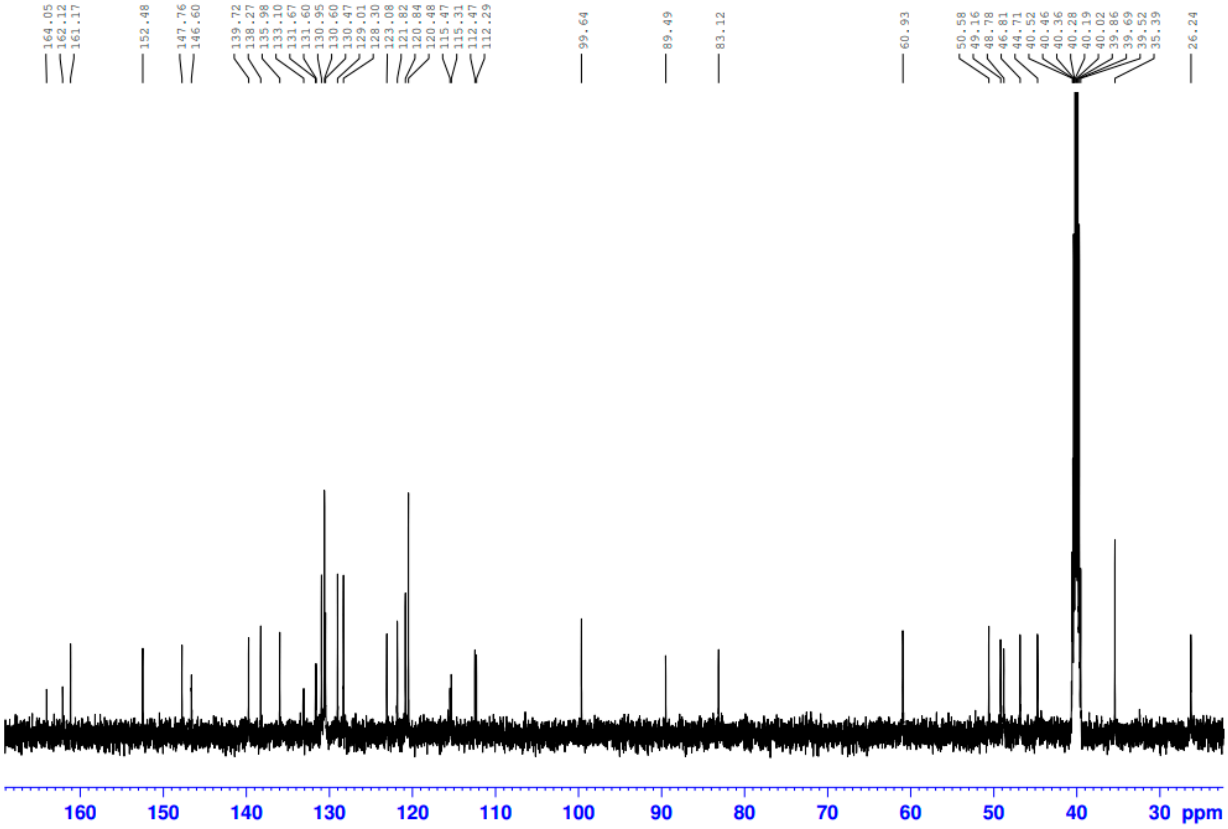

^{1}H-NMR of **36b**

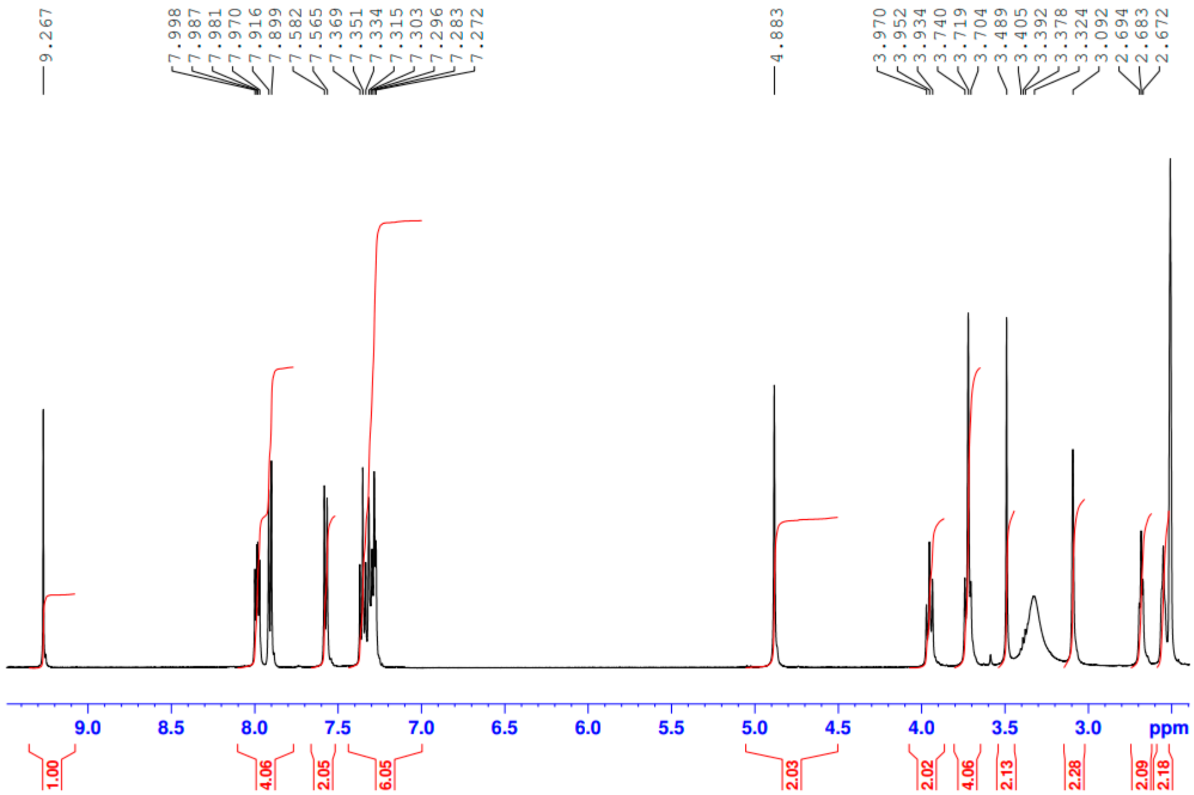

^{13}C-NMR of **36b**

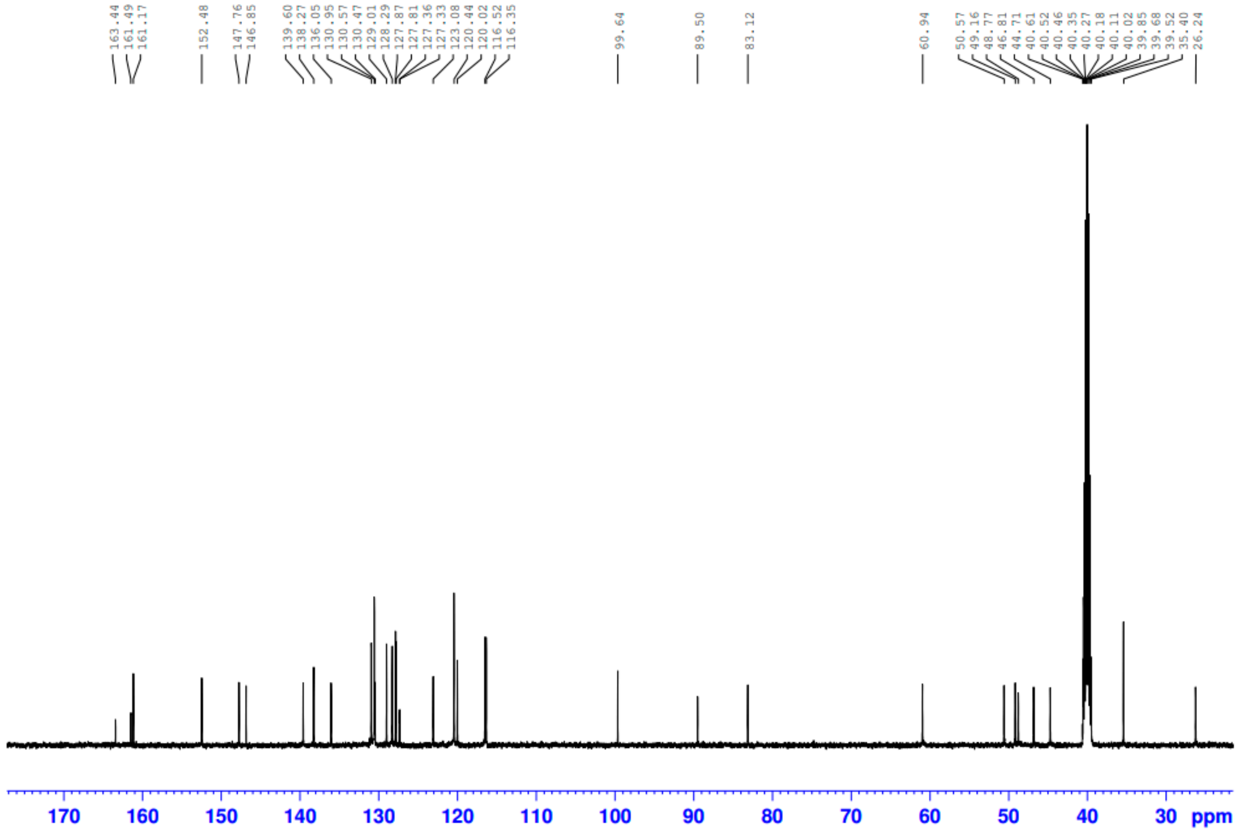

^{1}H-NMR of **36c**

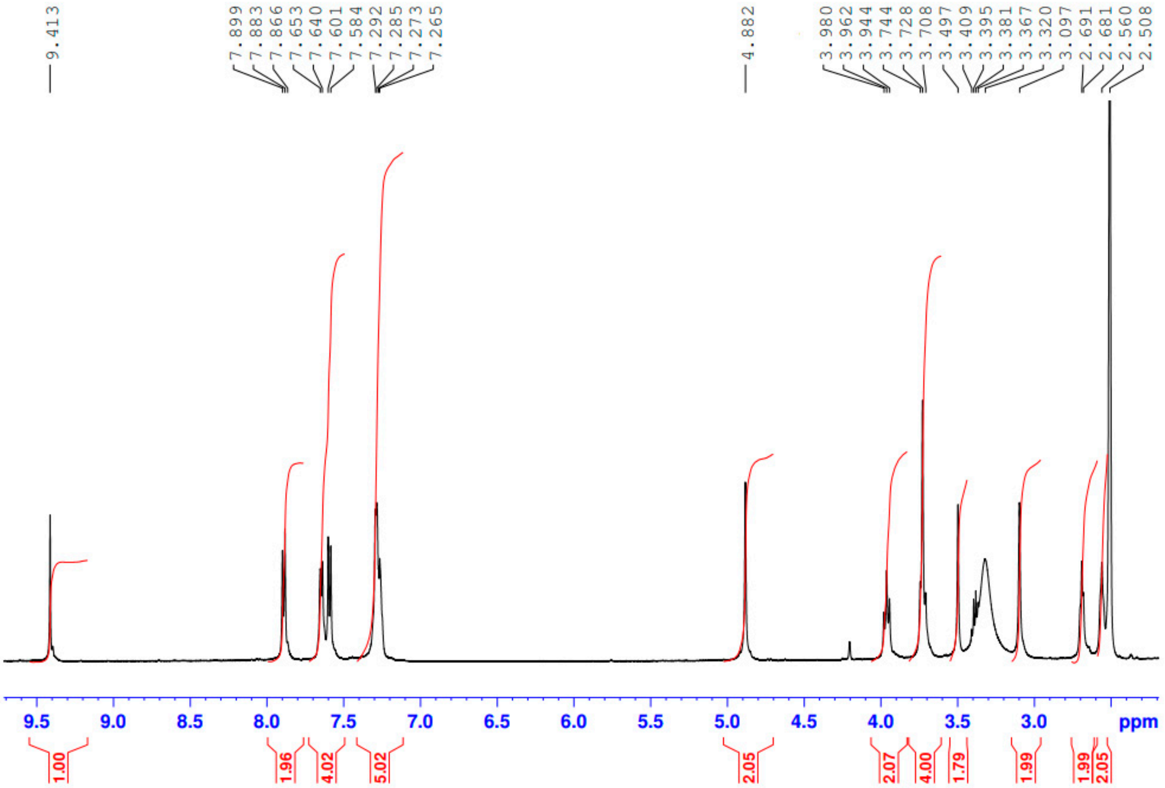

^{13}C-NMR of **36c**

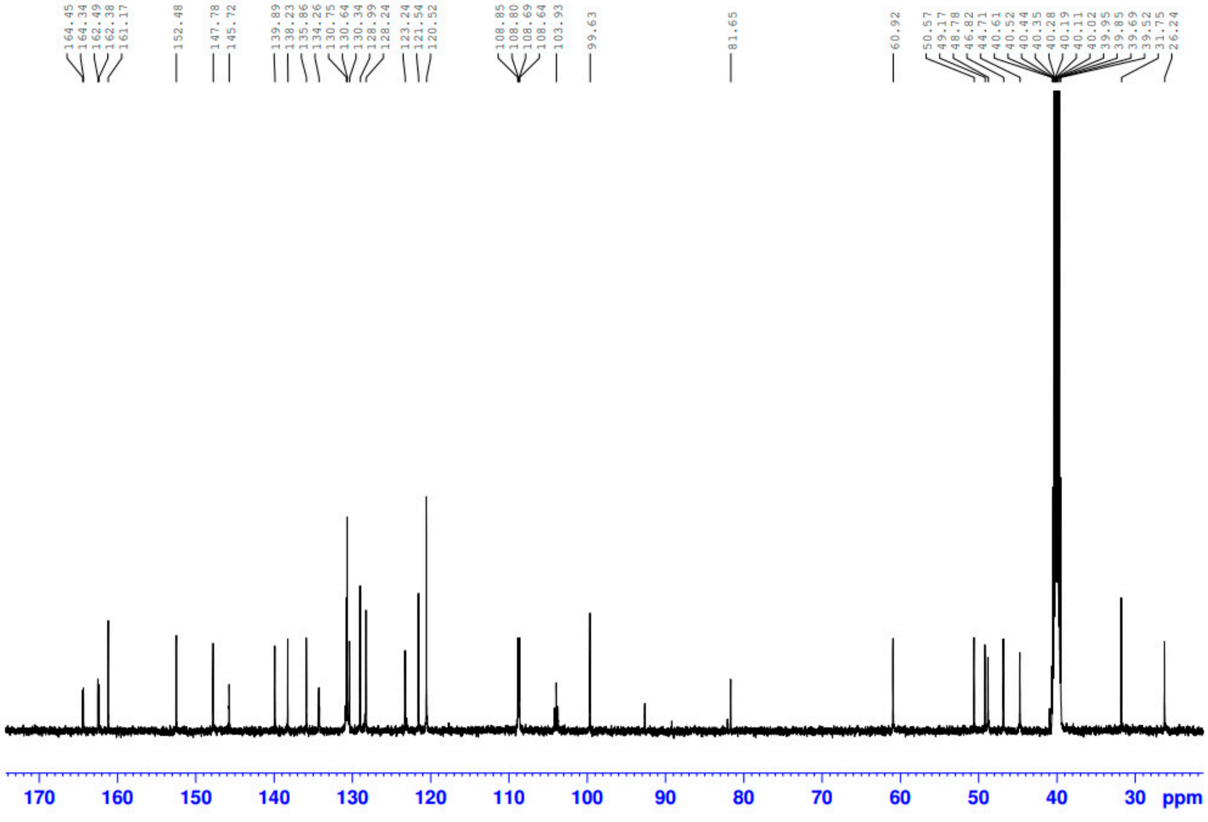

^{1}H-NMR of **37a**

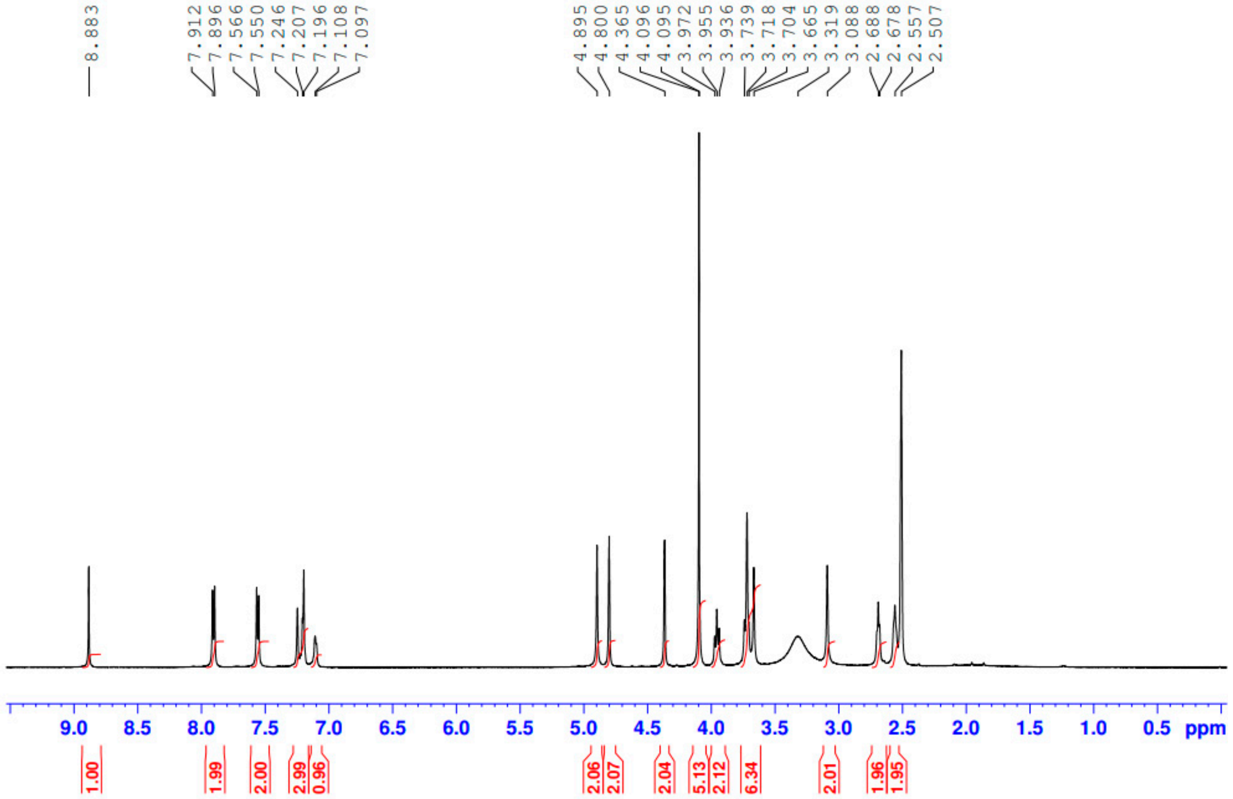

^{13}C-NMR of **37a**

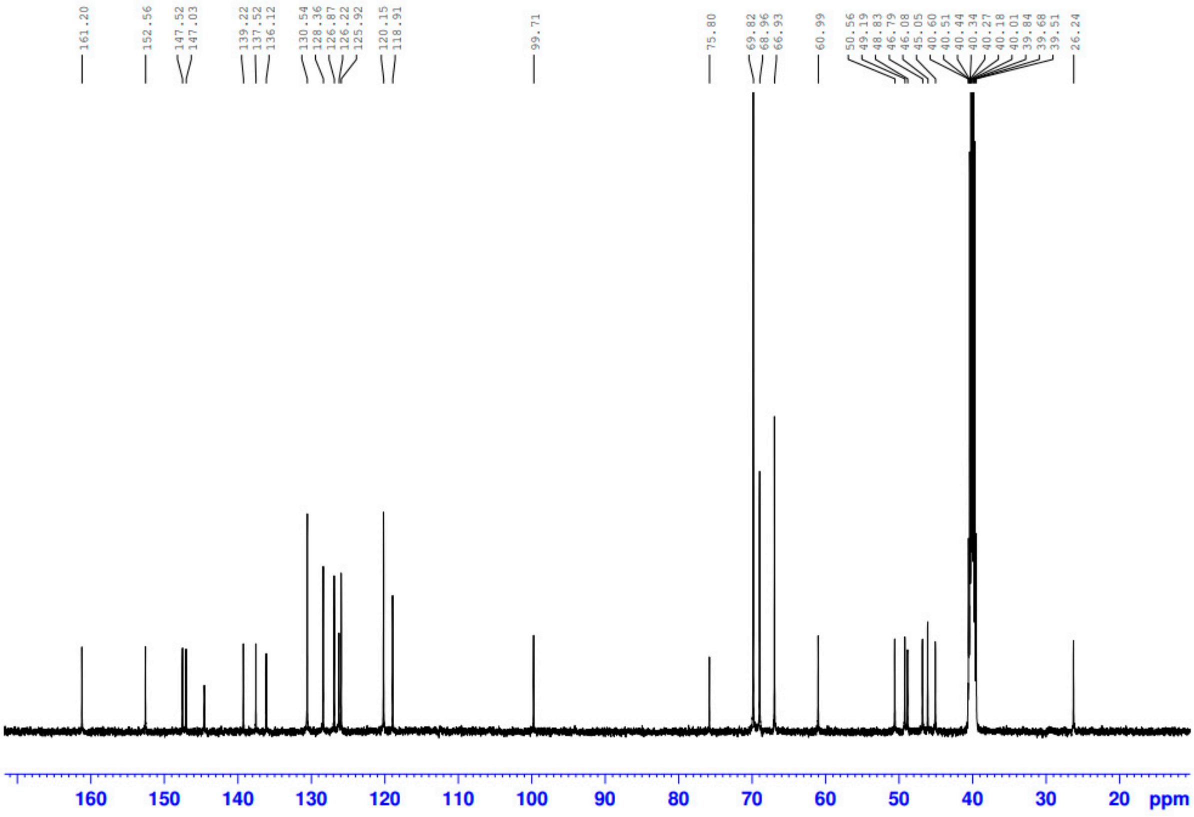

^{1}H-NMR of **37b**

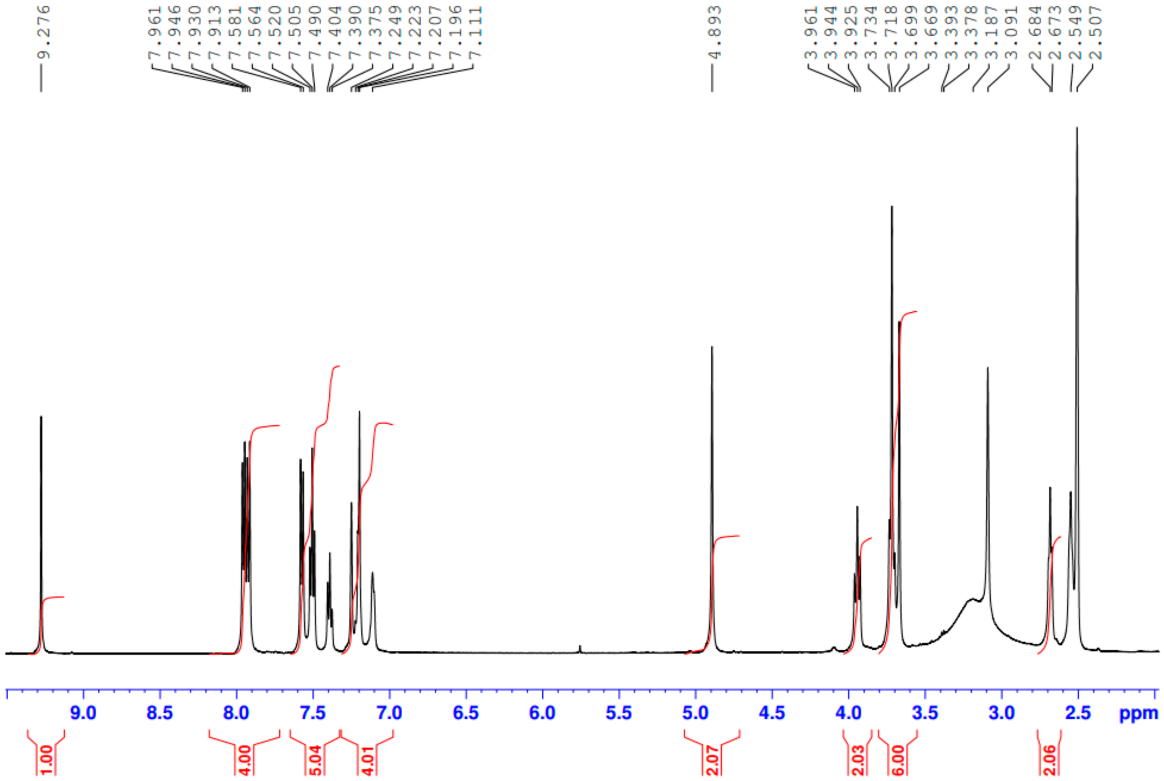

^{13}C-NMR of **37b**

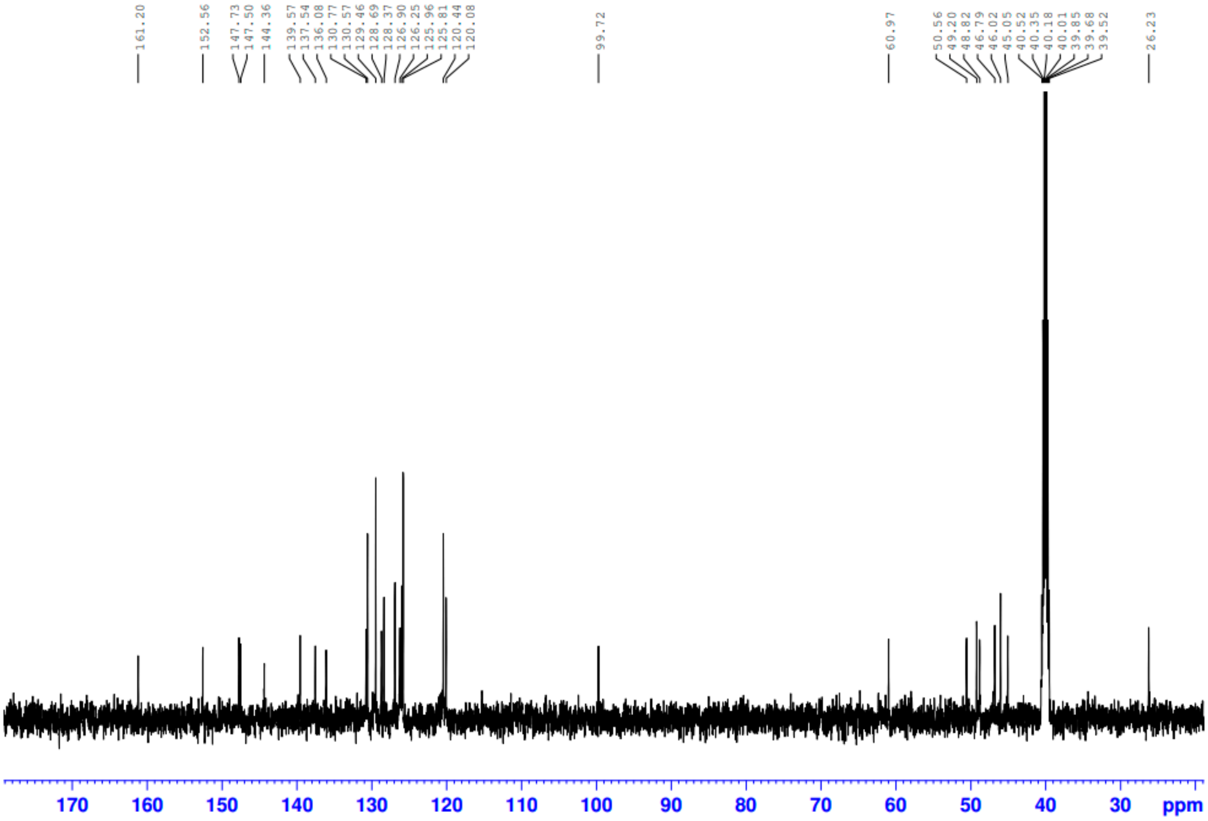

^{1}H-NMR of **38a**

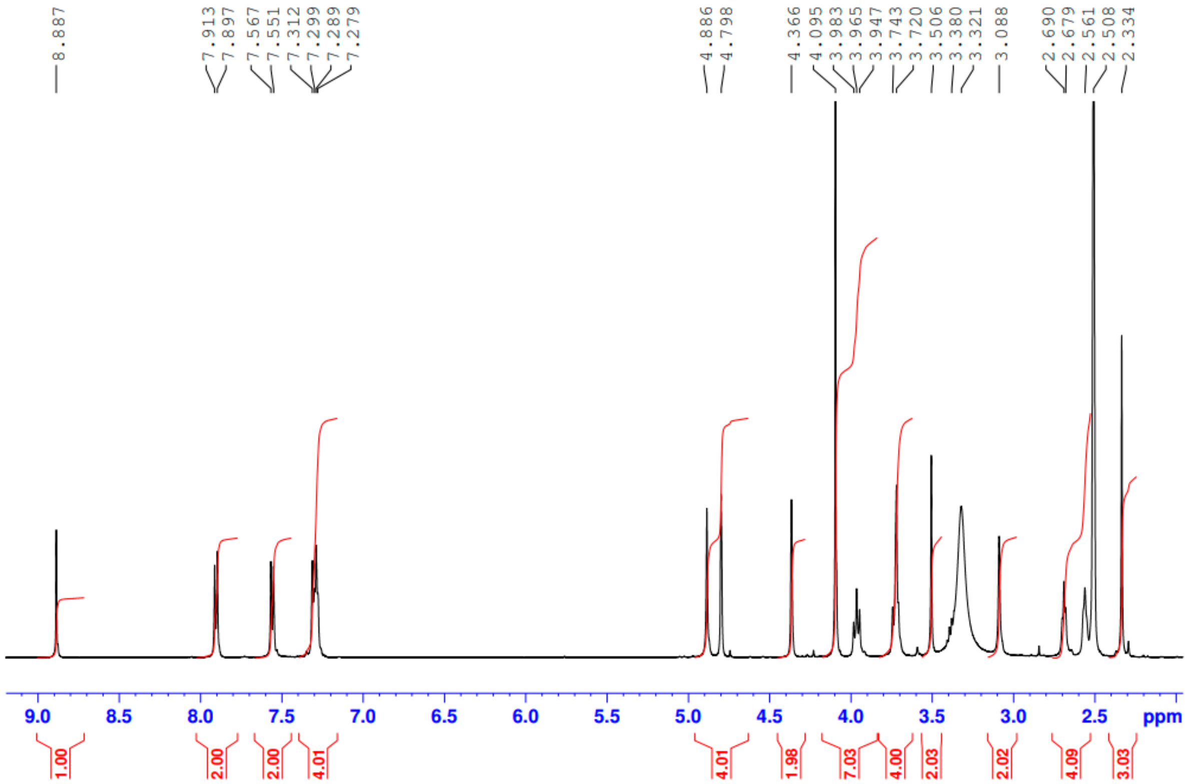

^{13}C-NMR of **38a**

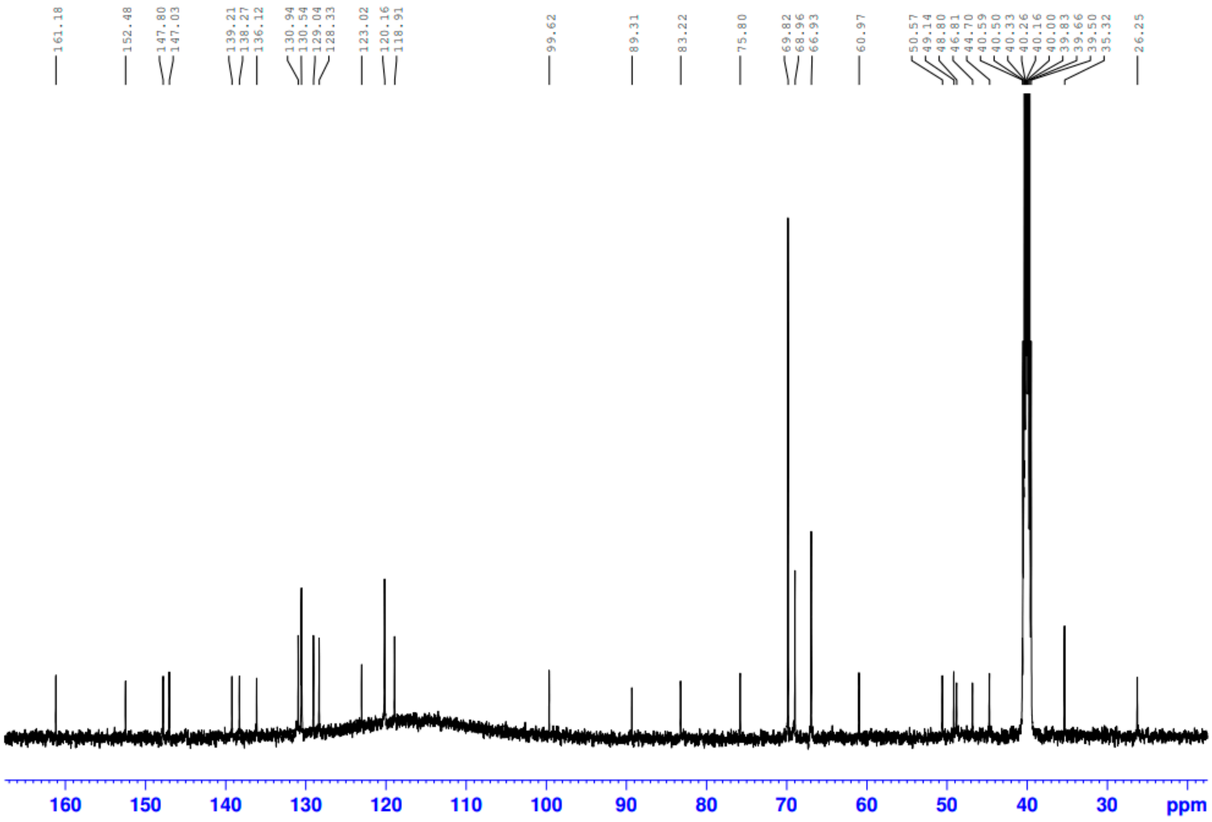

^{1}H-NMR of **38b**

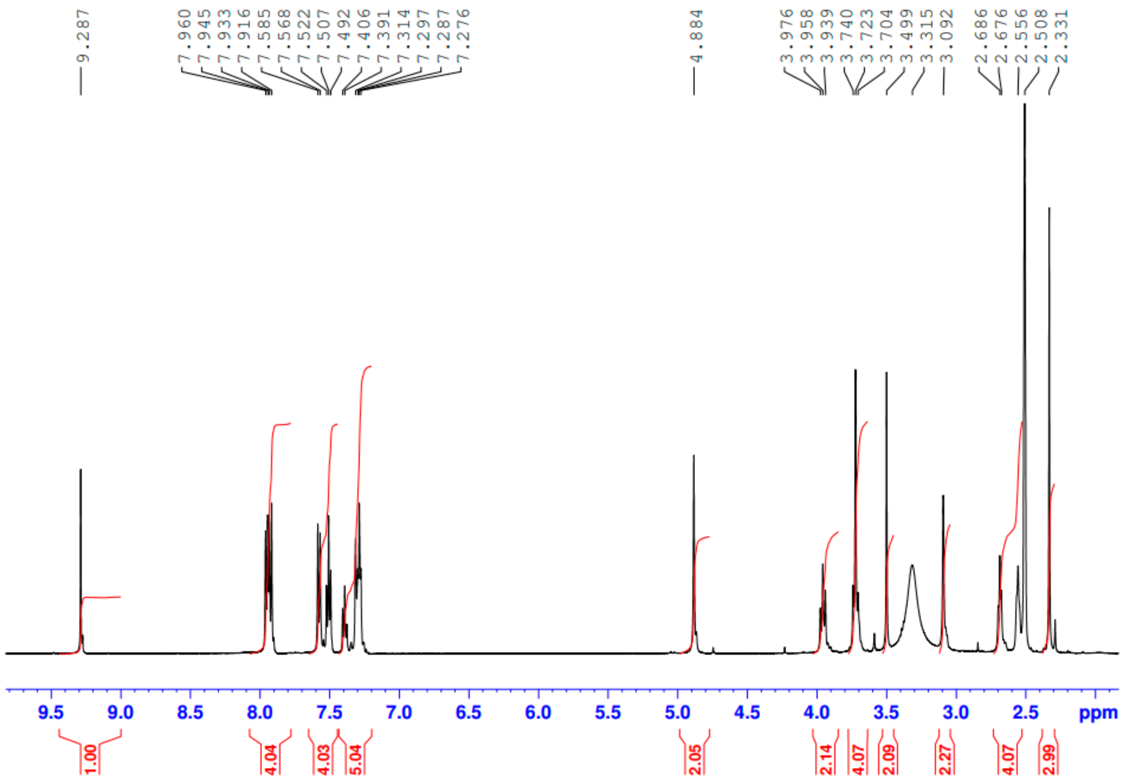

^{13}C-NMR of **38b**

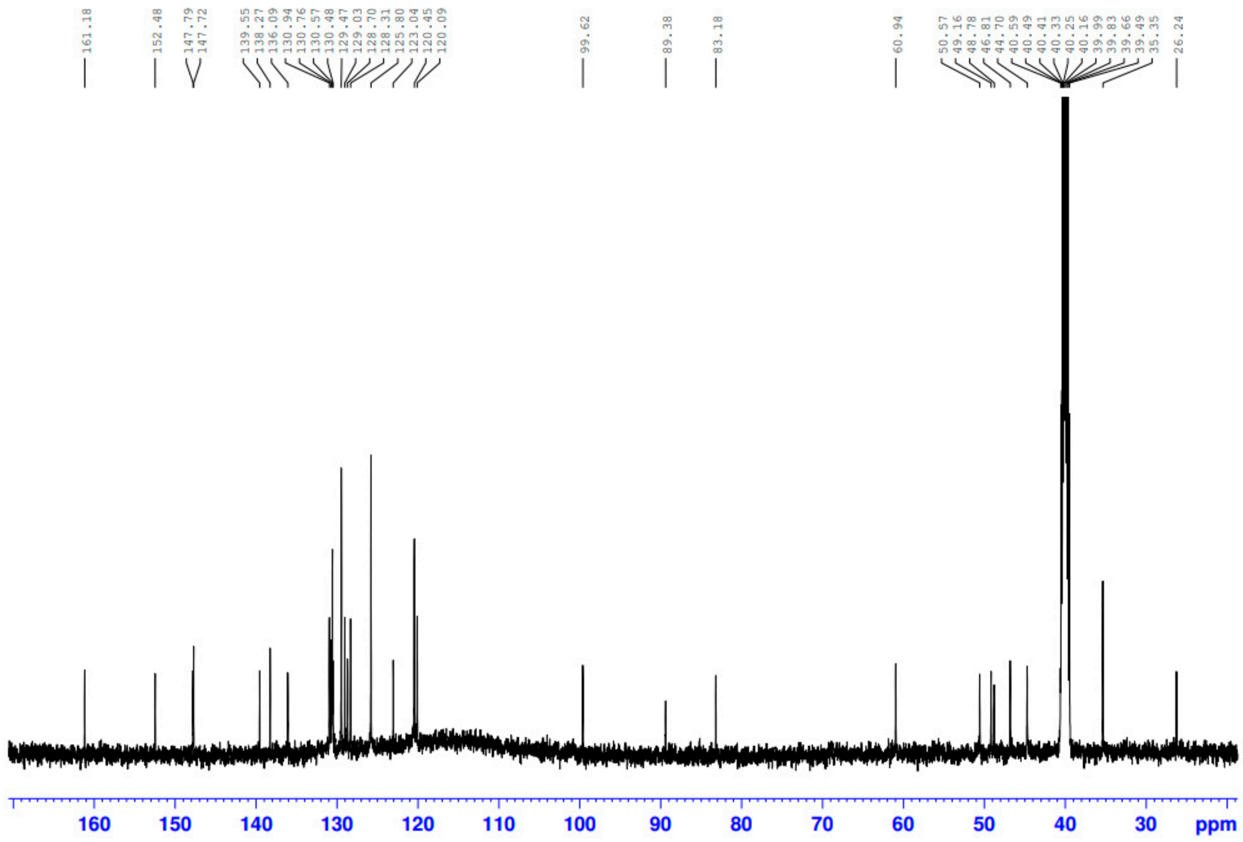

^{1}H-NMR of **39a**

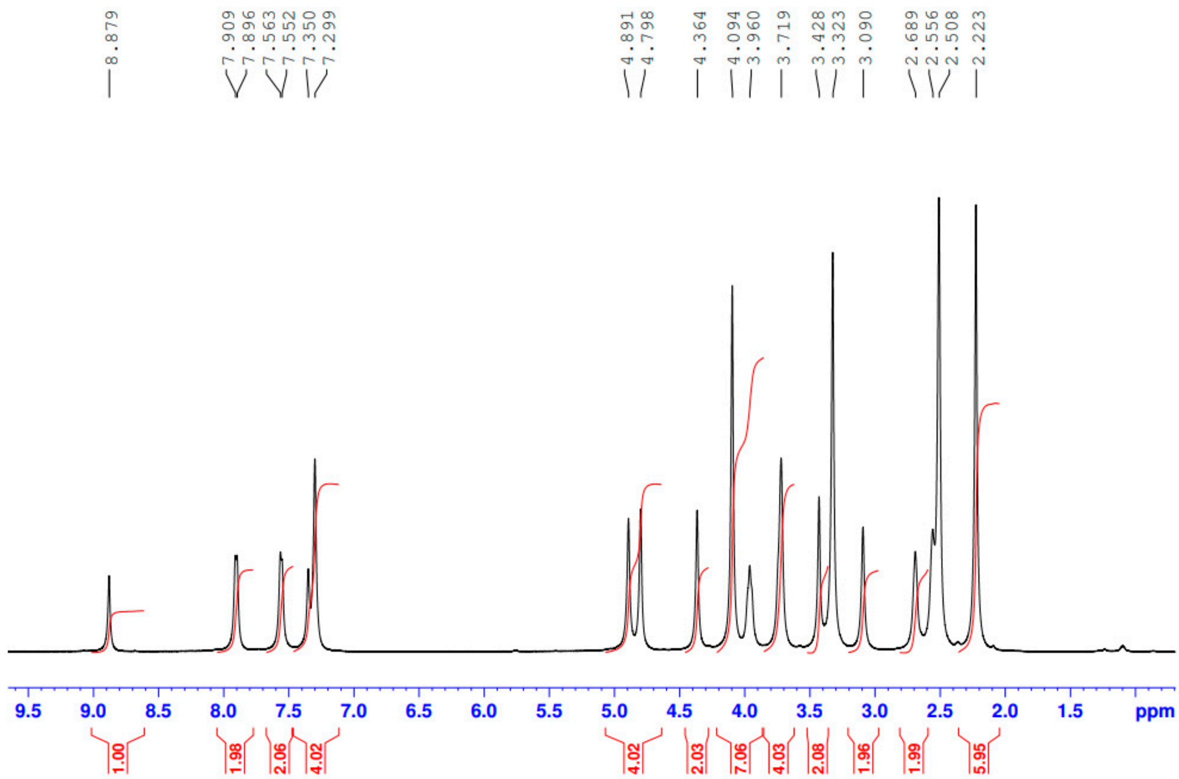

^{13}C-NMR of **39a**

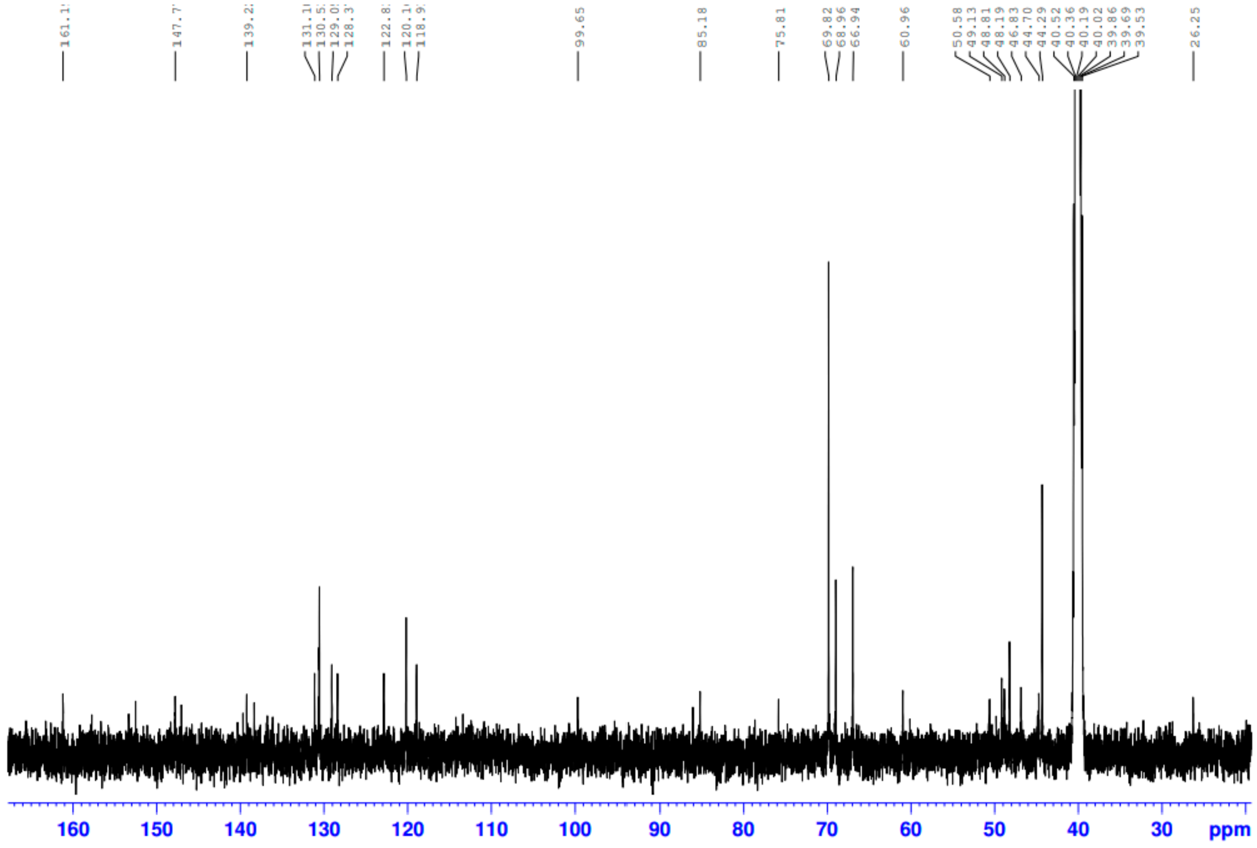

^{1}H-NMR of **39b**

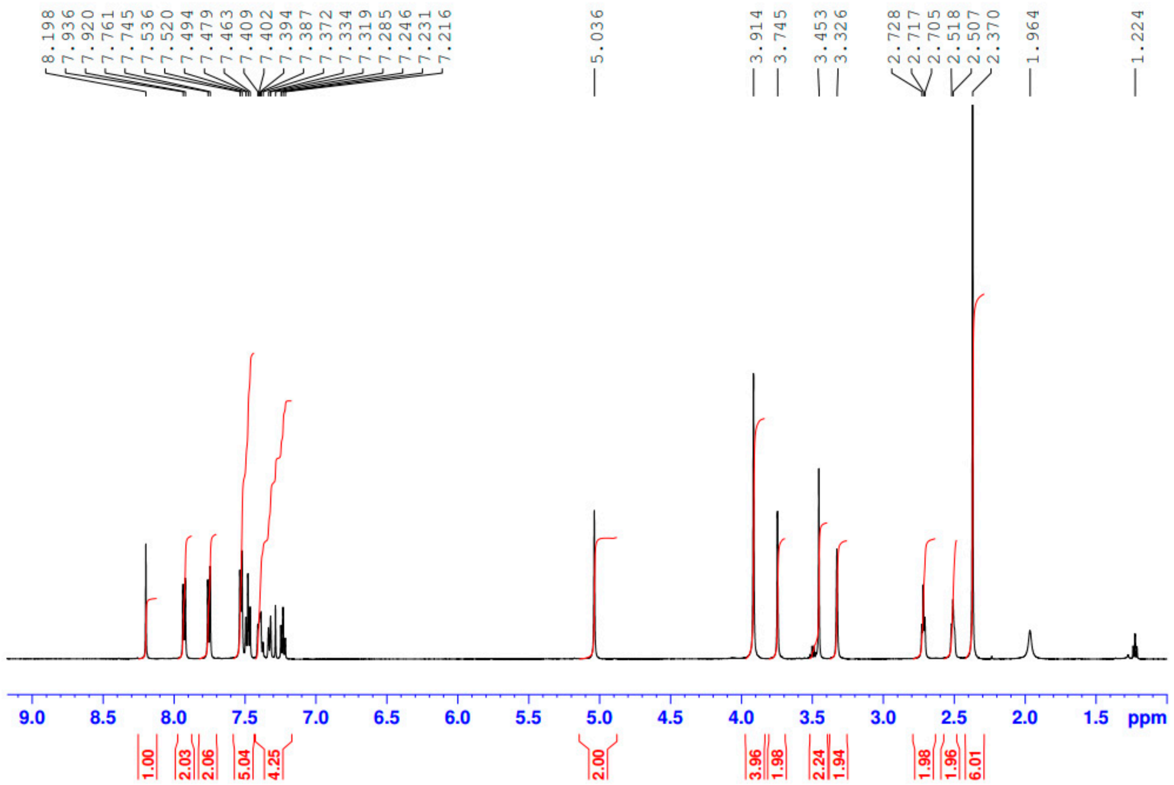

^{13}C-NMR of **39b**

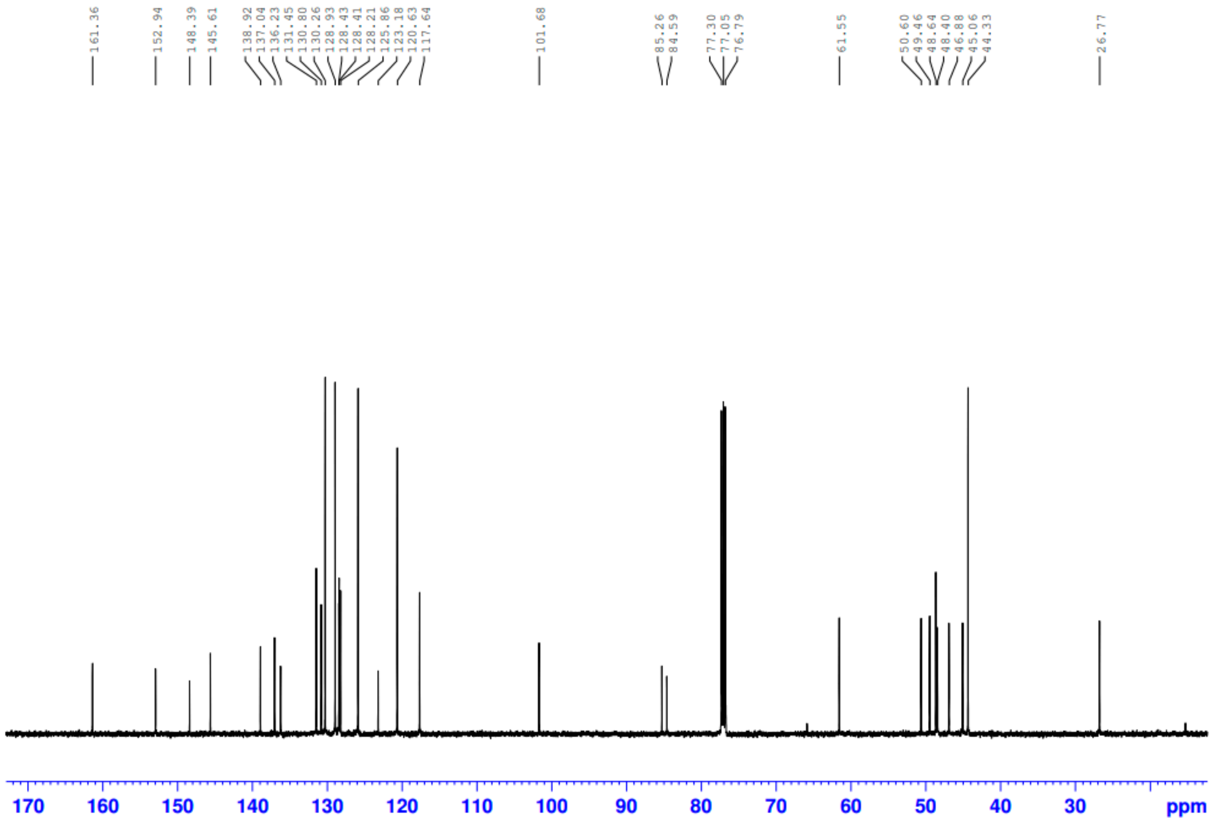

^{1}H-NMR of **40a**

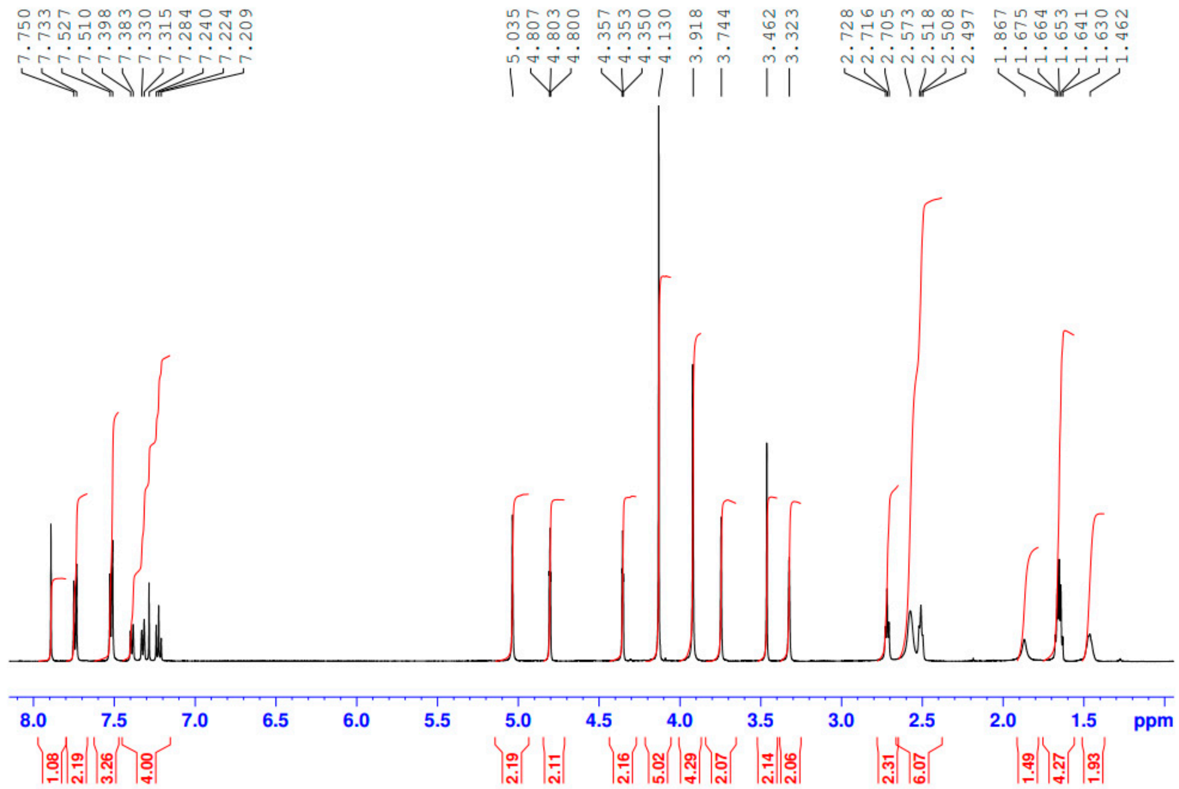

^{13}C-NMR of **40a**

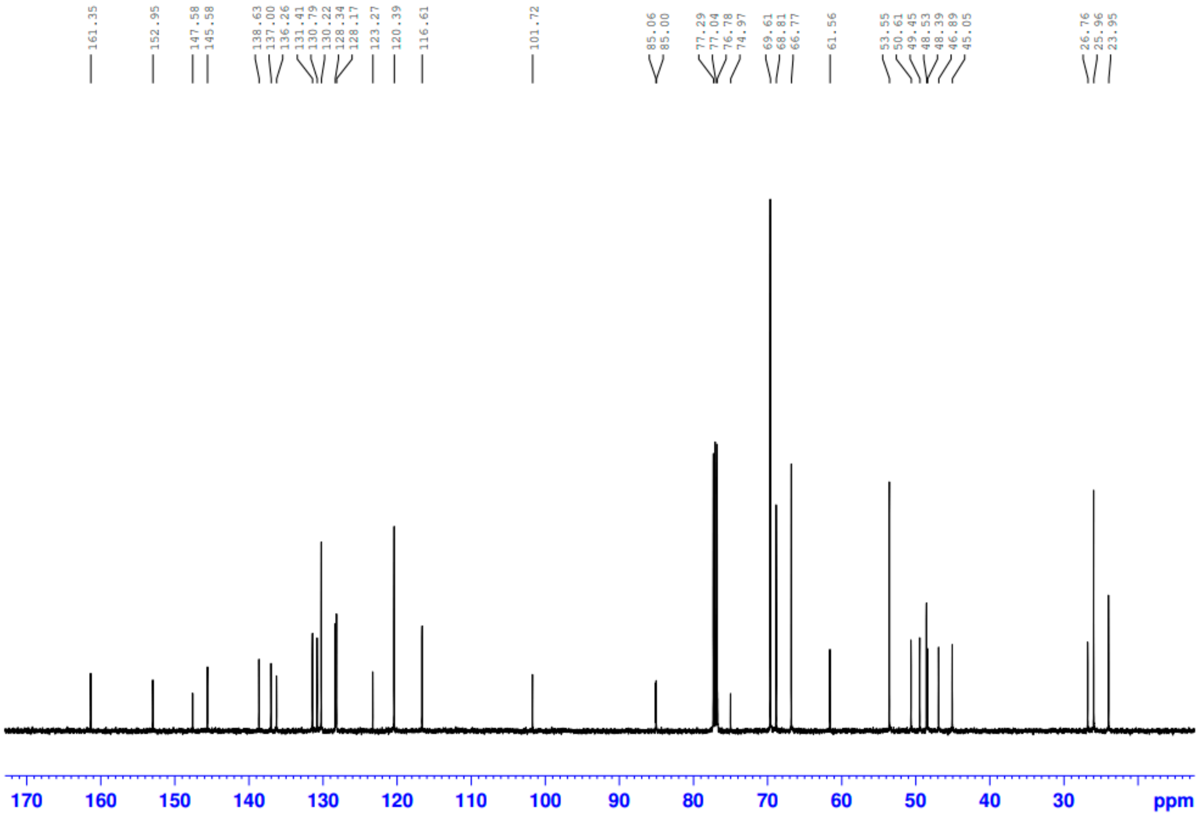

^{1}H-NMR of **41a**

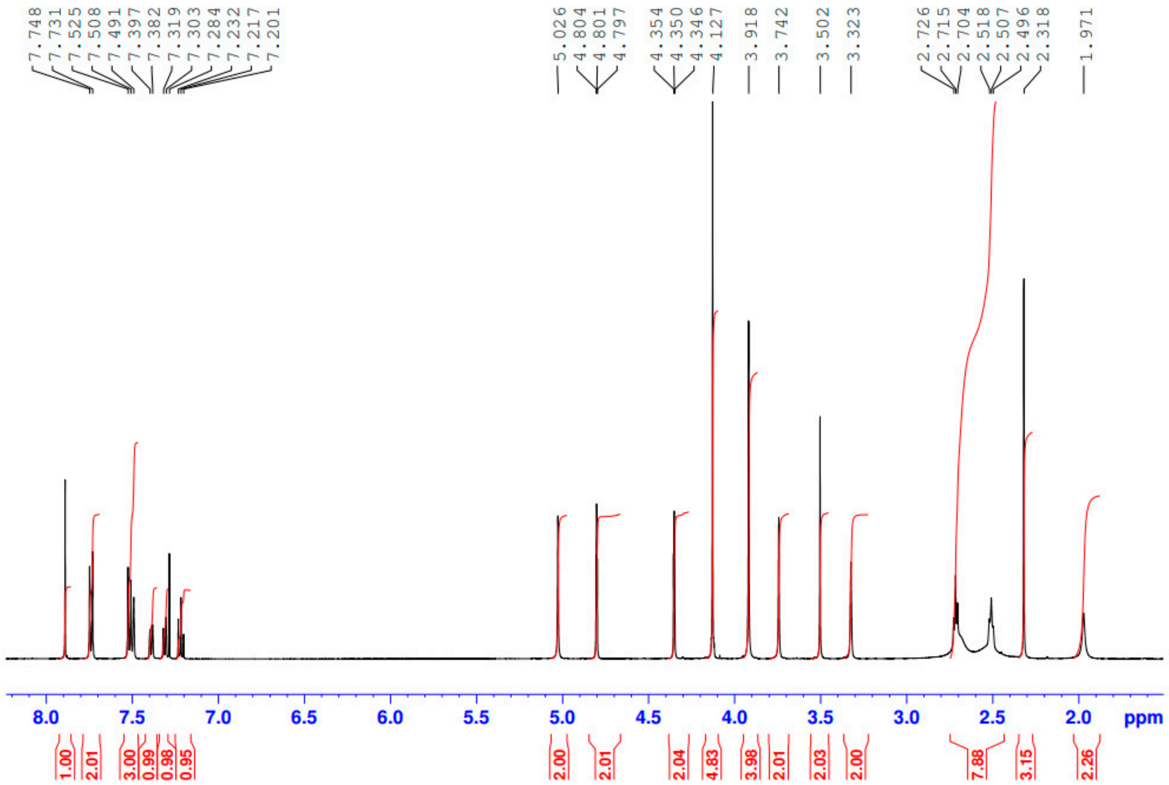

^{13}C-NMR of **41a**

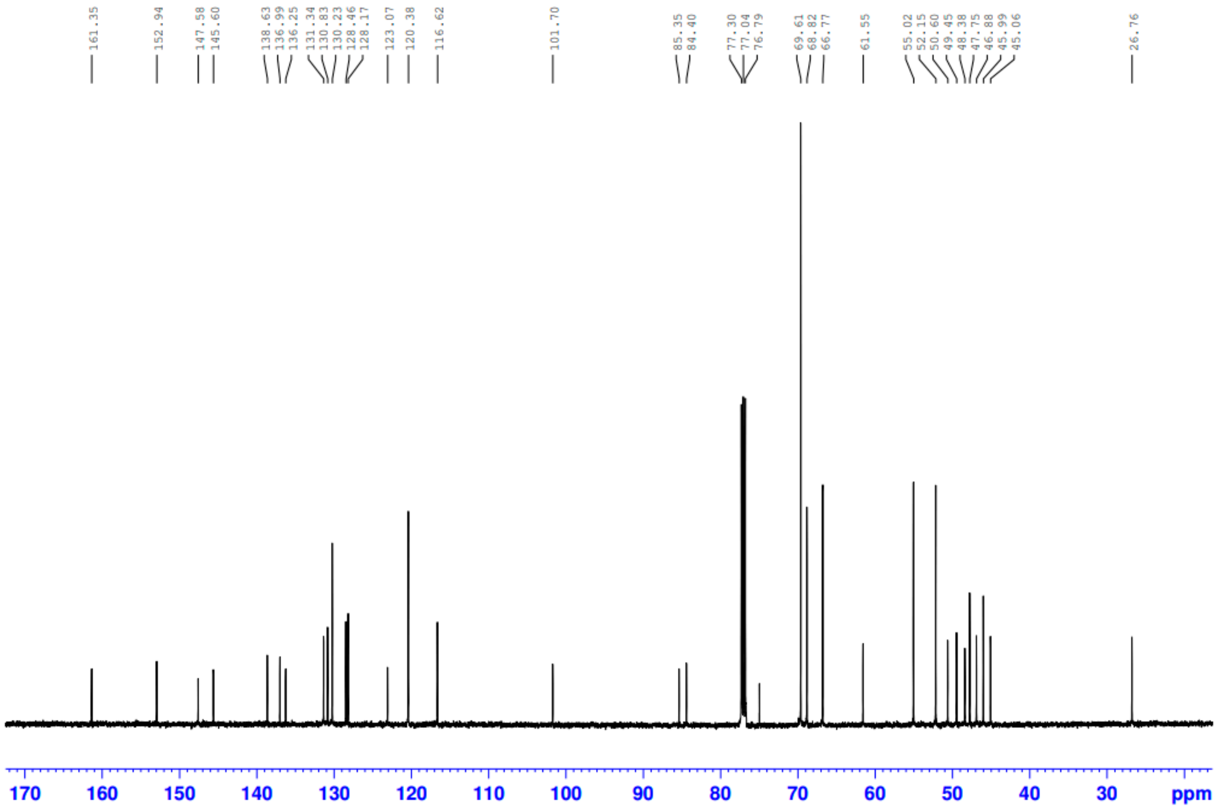

^{1}H-NMR of **42a**

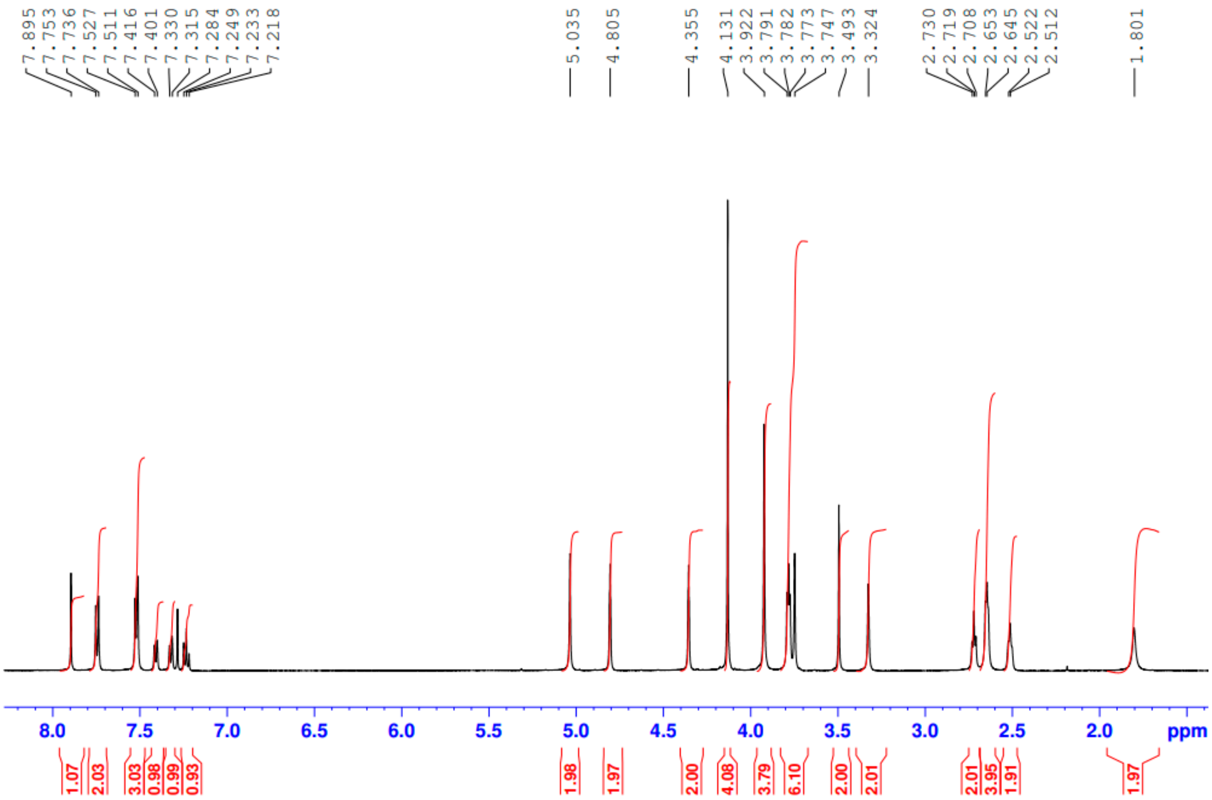

^{13}C-NMR of **42a**

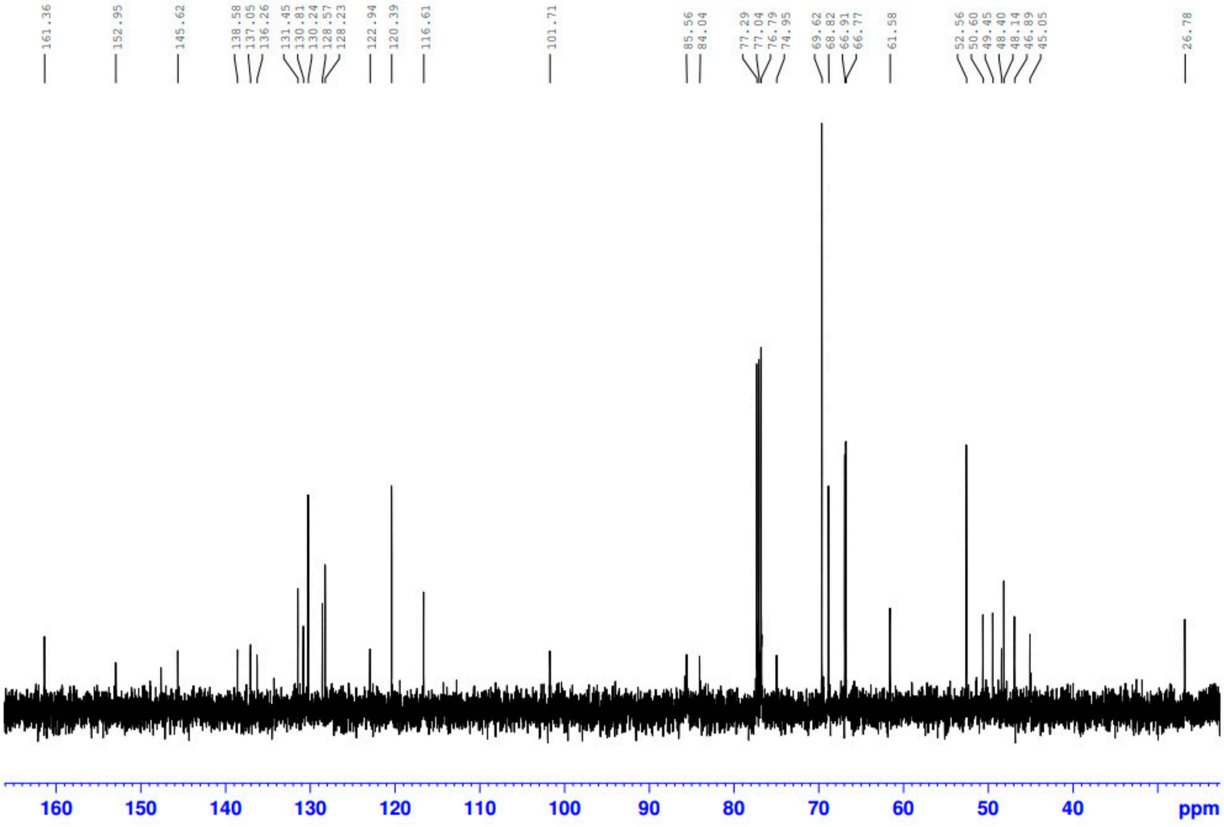

¹H-NMR of **42b**

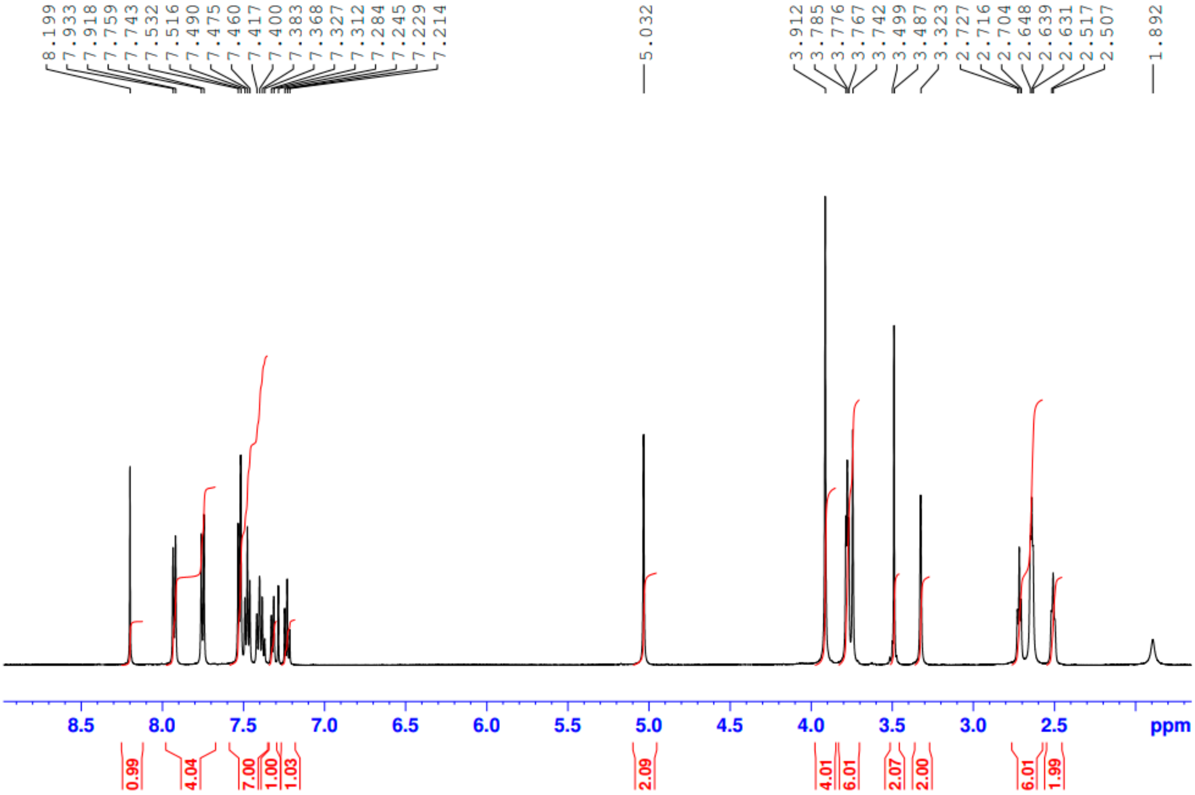

¹³C-NMR of **42b**

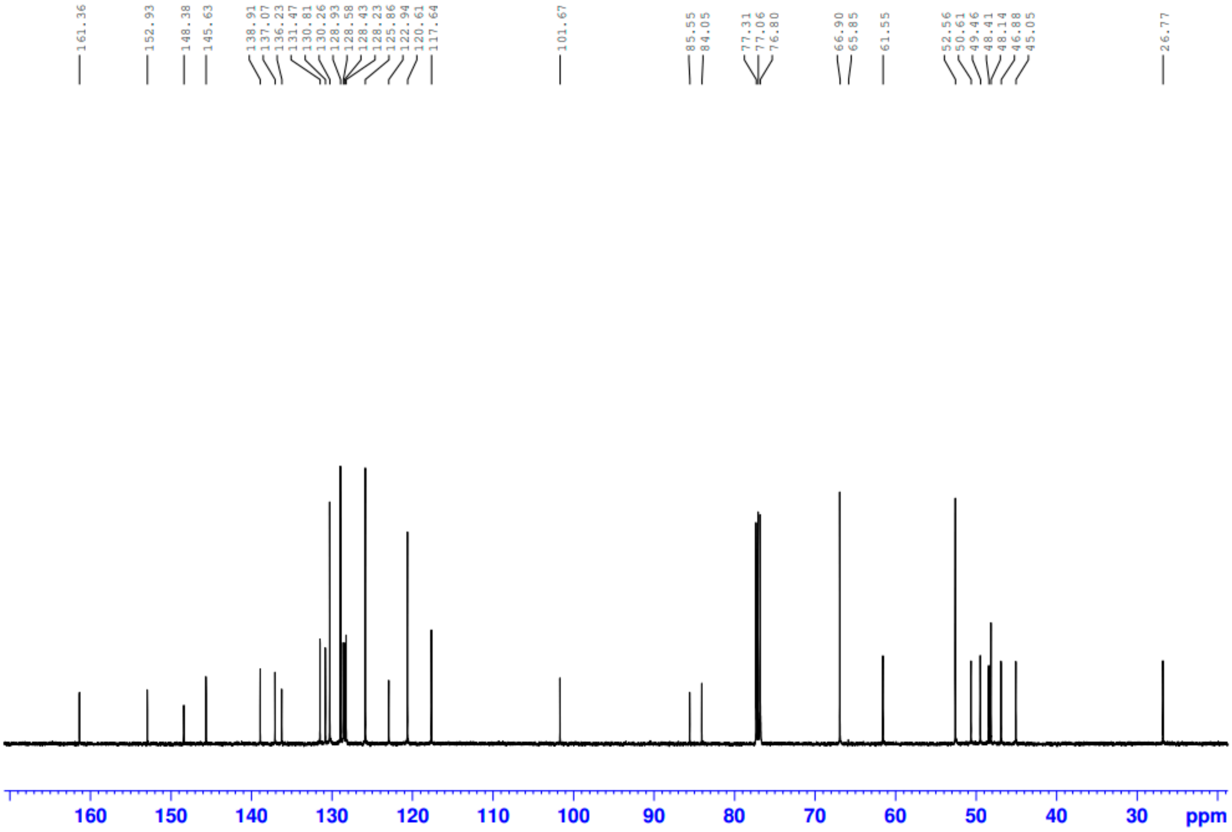

^{1}H-NMR of **43a**

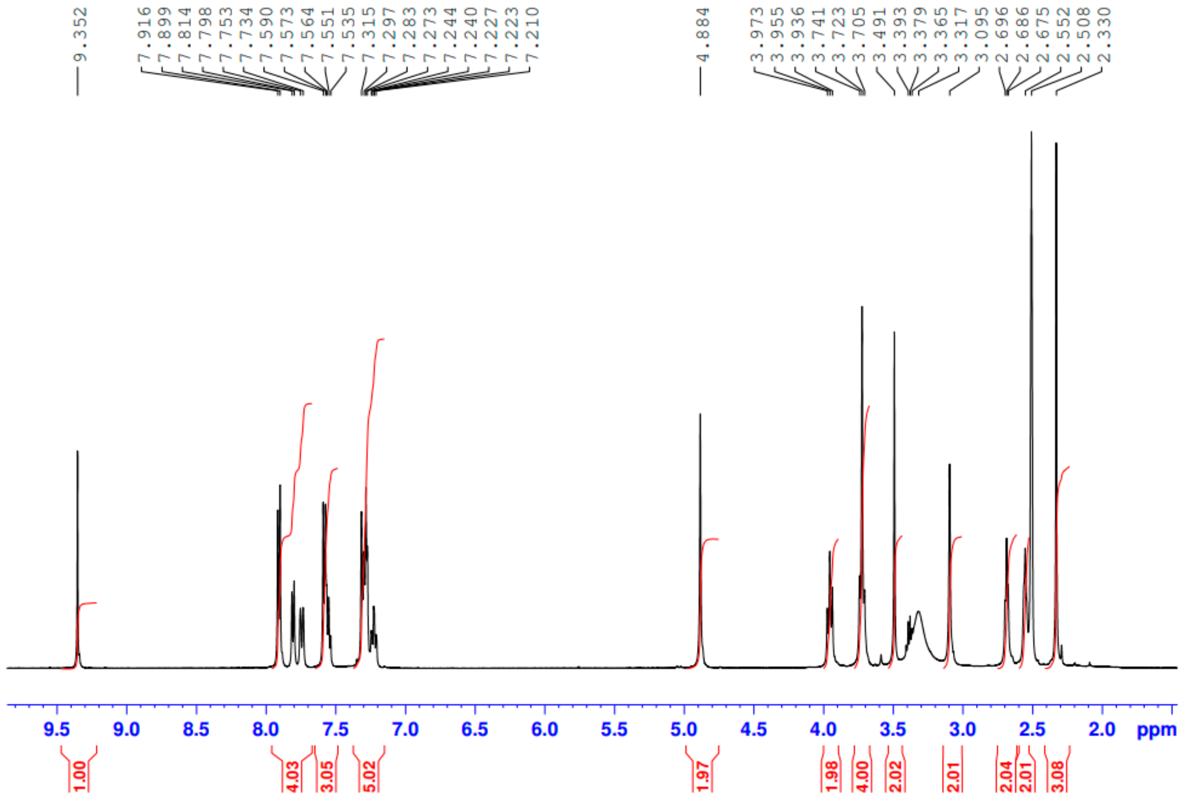

^{13}C-NMR of **43a**

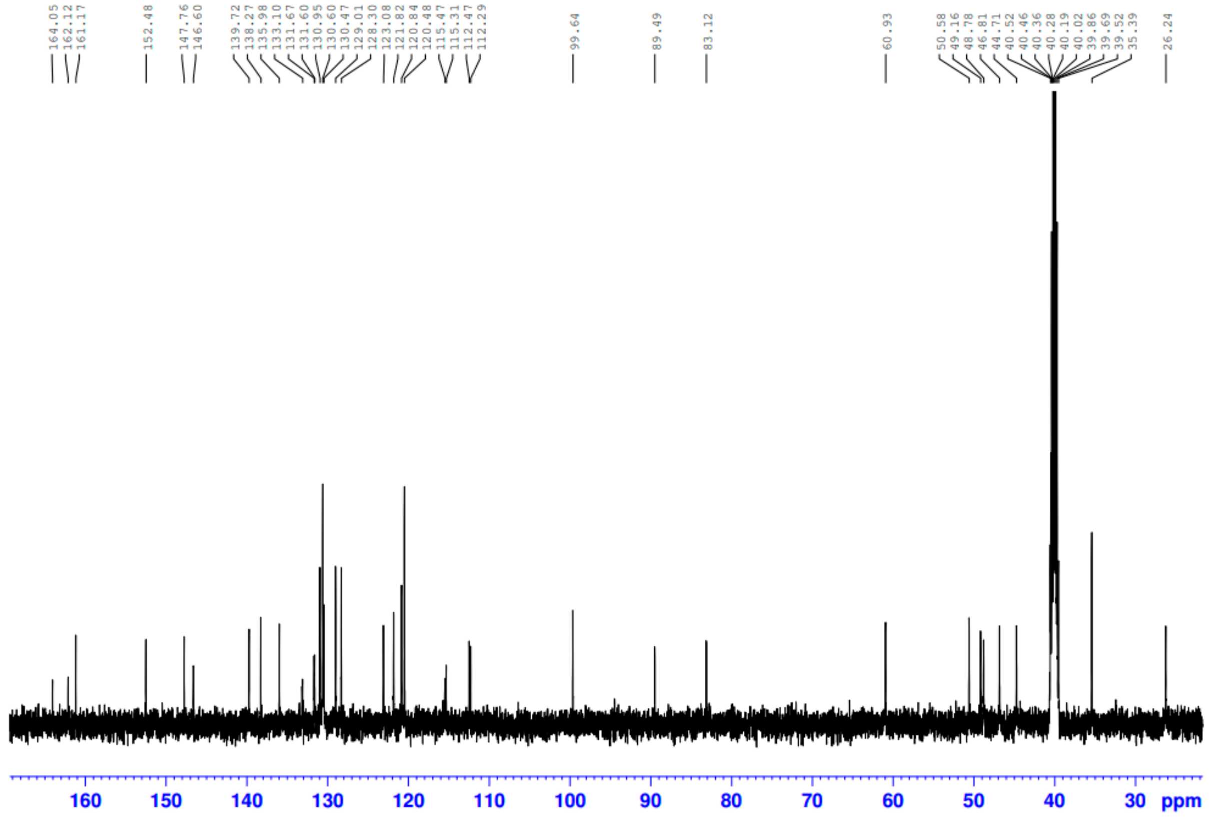

^{1}H-NMR of **43b**

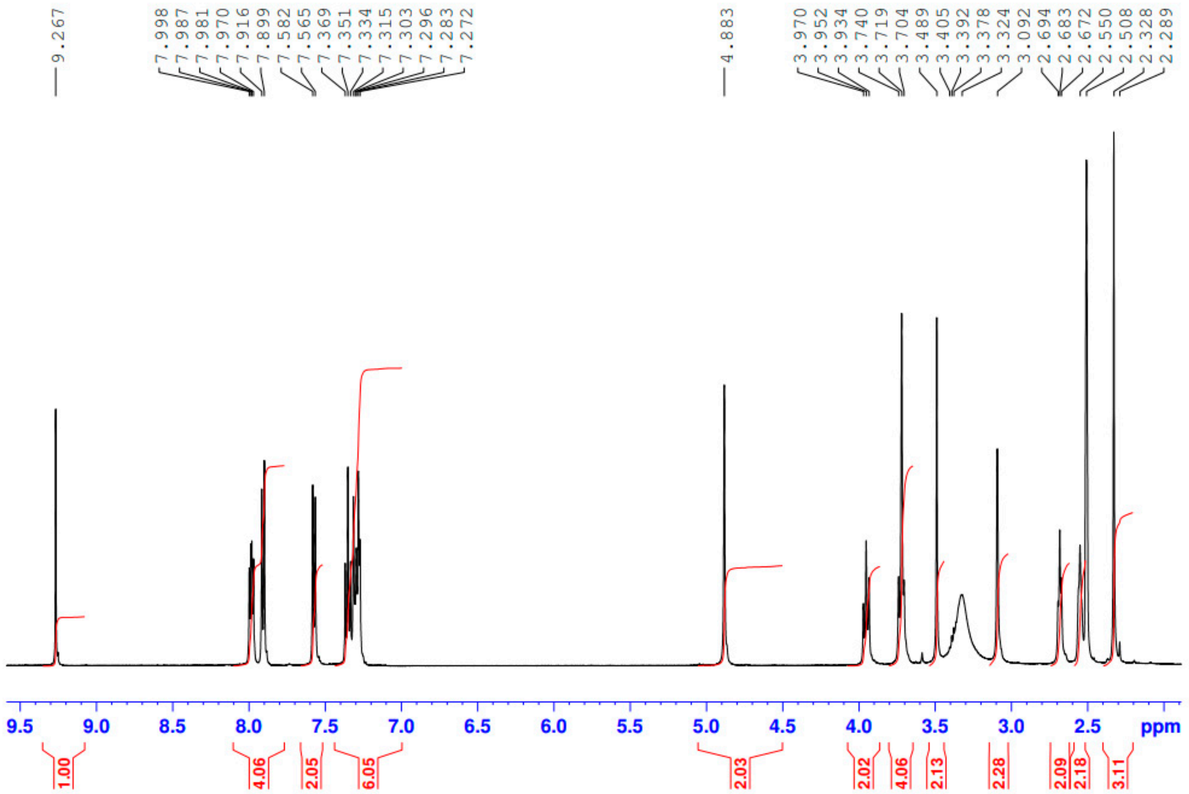

^{13}C-NMR of **43b**

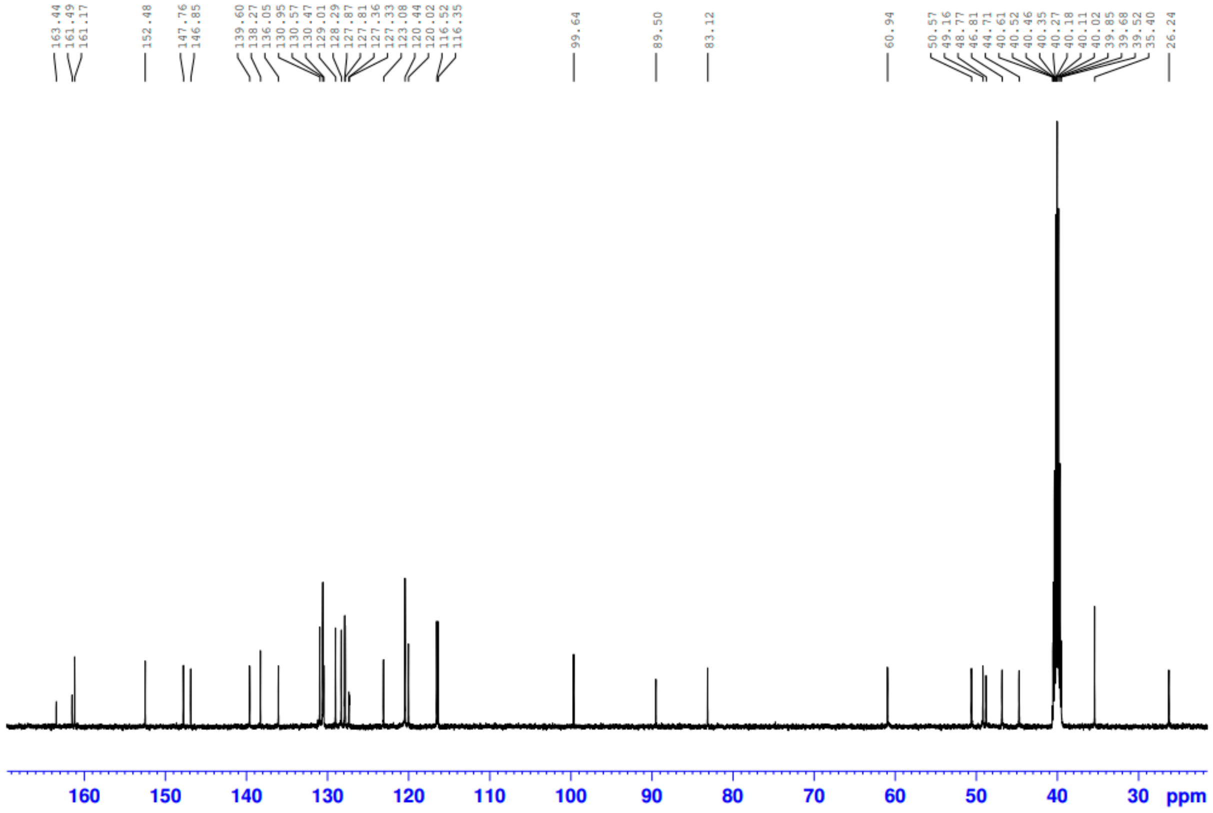

^{1}H-NMR of **43c**

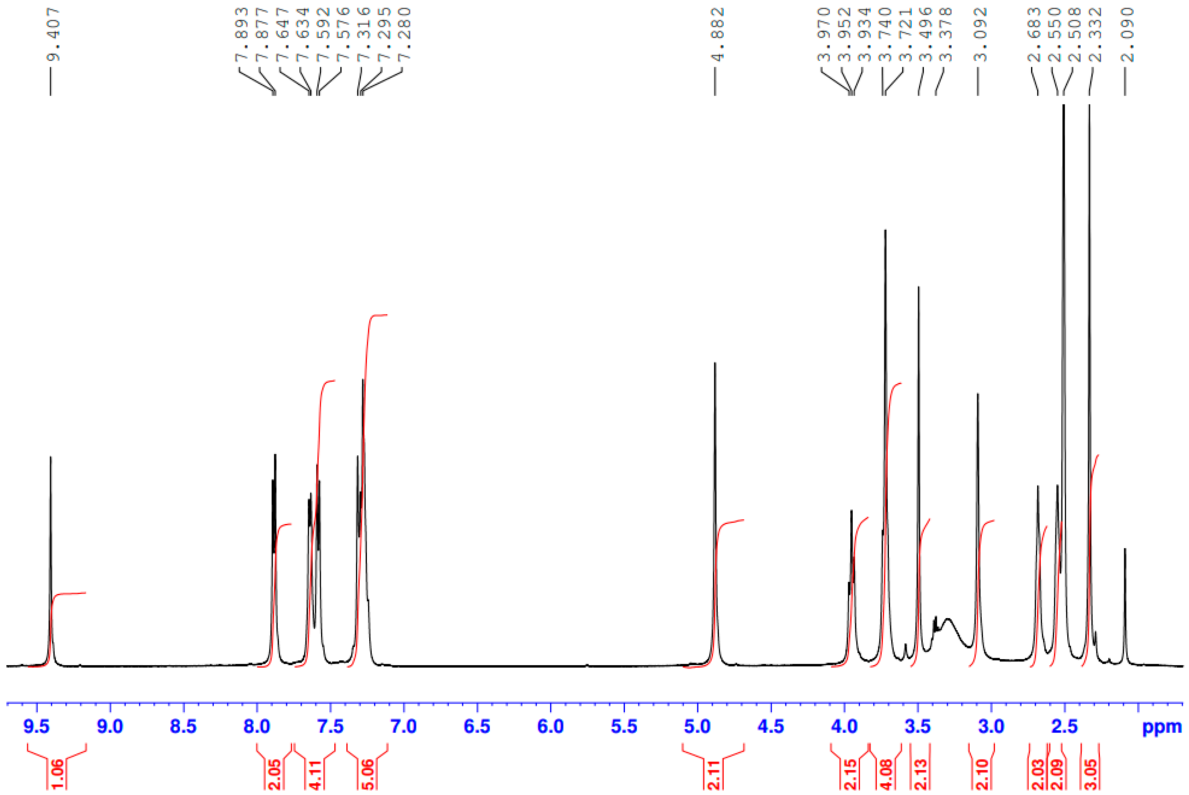

^{13}C-NMR of **43c**

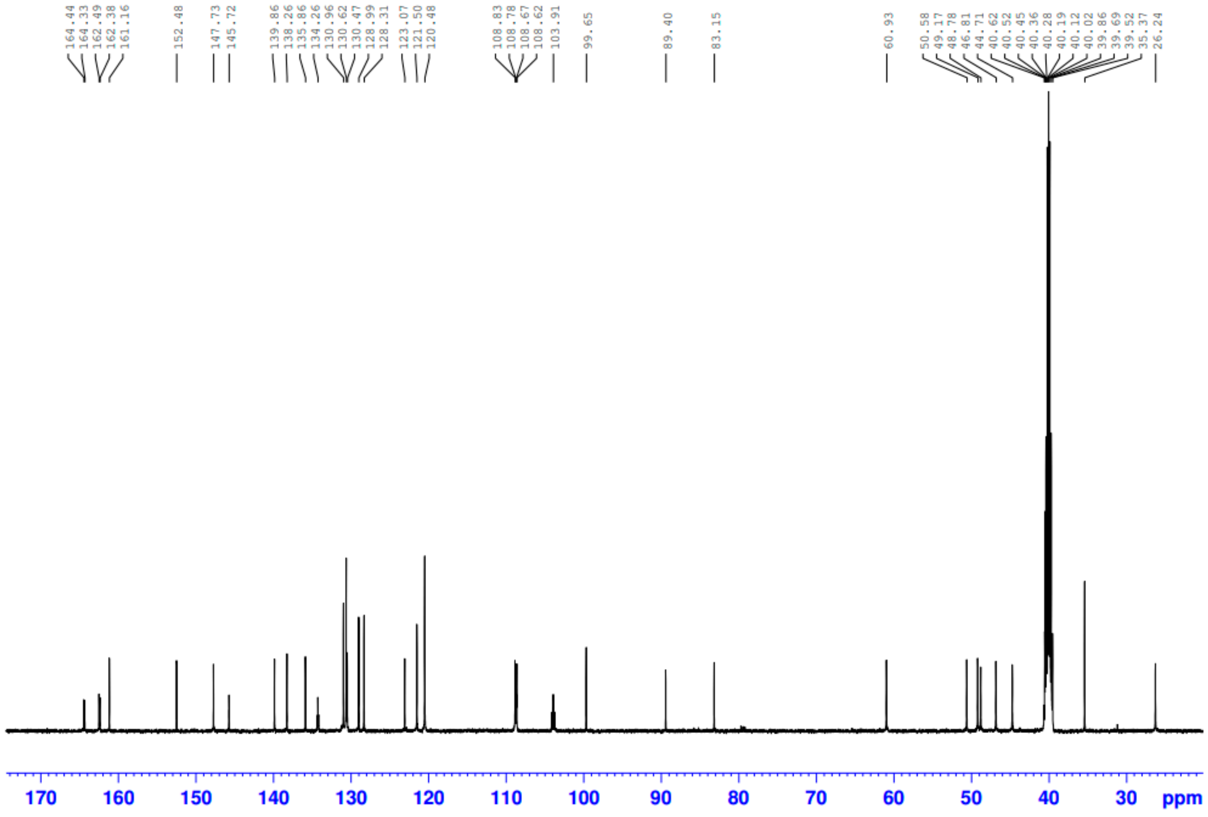

S3. Copy of HRMS spectra of the targeted compounds

HRMS of **1a**

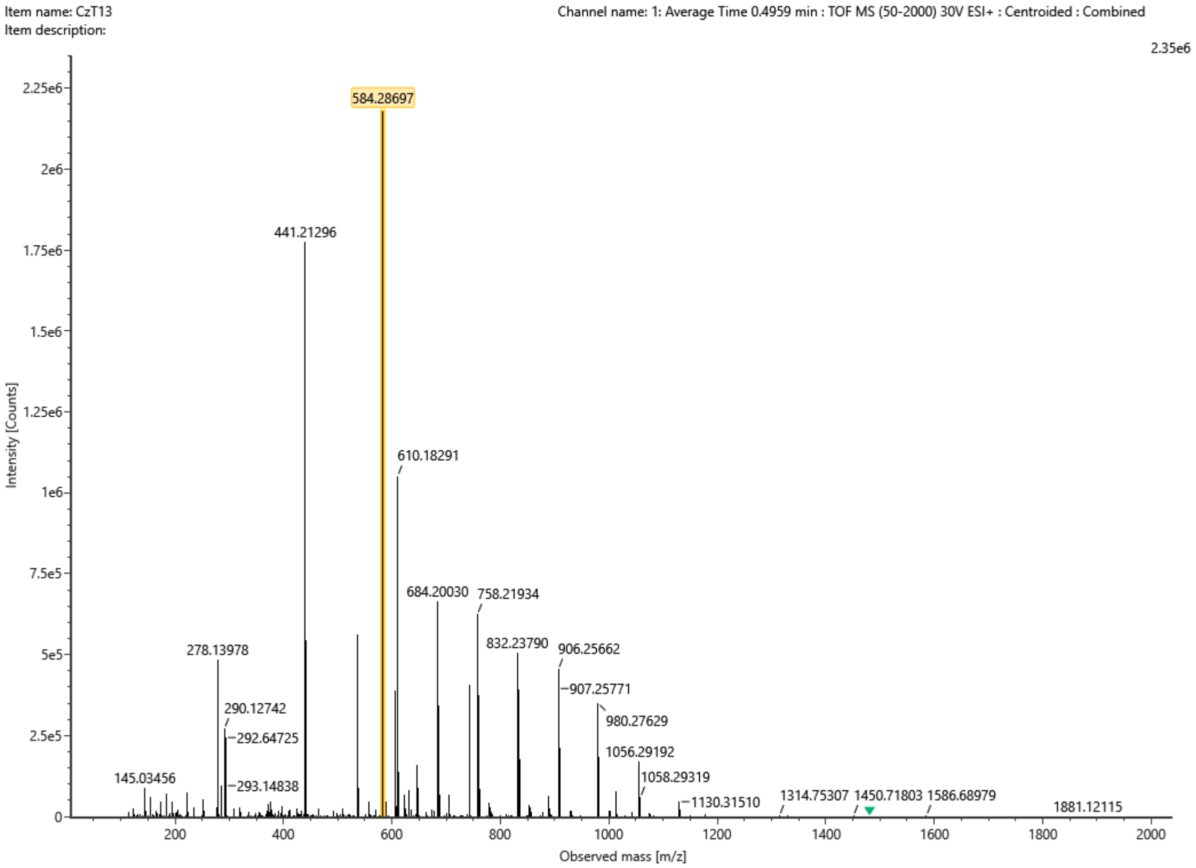

HRMS of **1b**

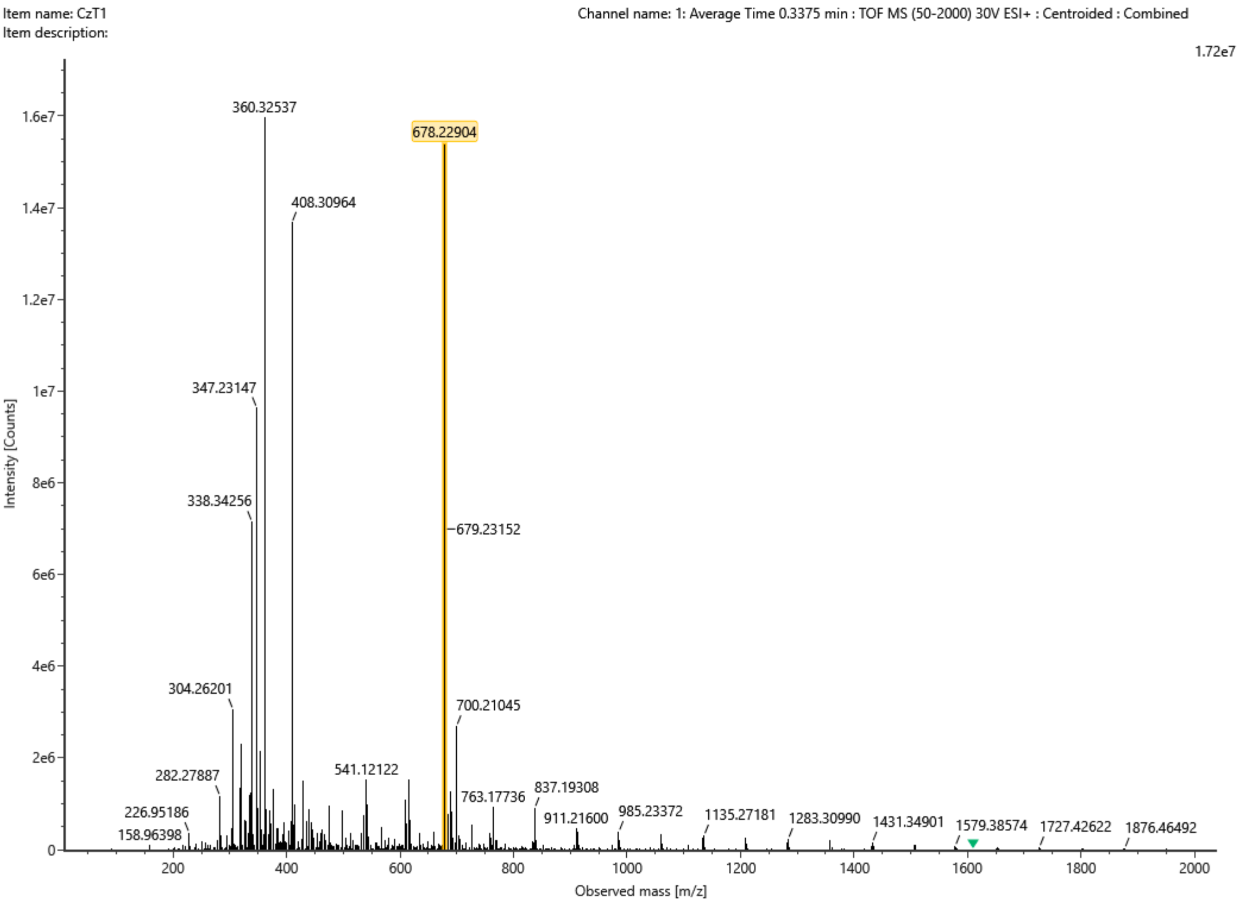

Item name: CzT1
Item description:
Channel name: 1: Average Time 0.3375 min : TOF MS (50-2000) 30V ESI+ : Centroided : Combined
1.72e7
Intensity [Counts]
Observed mass [m/z]
360.32537
678.22904
408.30964
347.23147
338.34256
679.23152
304.26201
700.21045
282.27887
541.12122
763.17736
837.19308
226.95186
158.96398
911.21600
985.23372
1135.27181
1283.30990
1431.34901
1579.38574
1727.42622
1876.46492

HRMS of **1c**

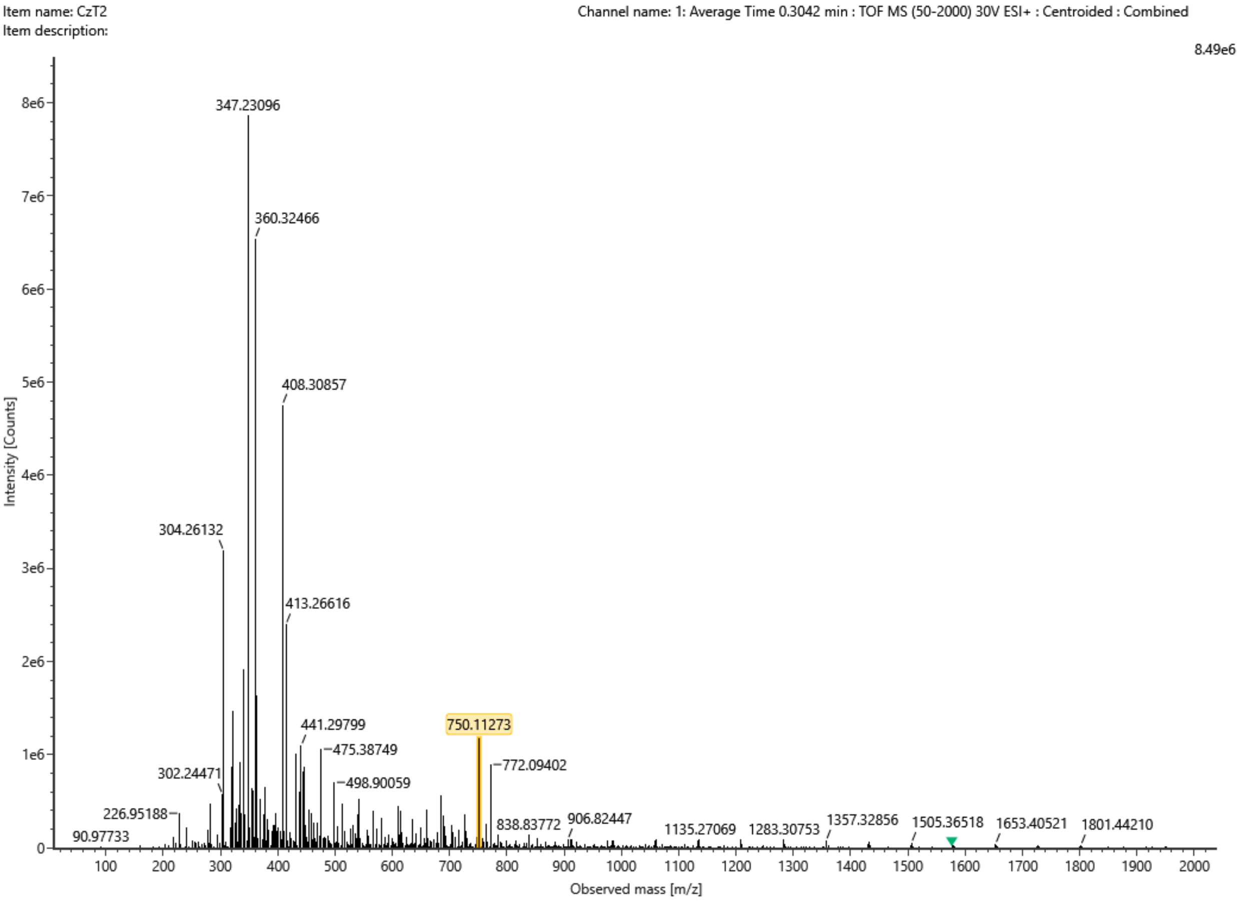

Item name: CzT2
Item description:
Channel name: 1: Average Time 0.3042 min : TOF MS (50-2000) 30V ESI+ : Centroided : Combined
8.49e6
Intensity [Counts]
Observed mass [m/z]
347.23096
360.32466
408.30857
304.26132
413.26616
441.29799
475.38749
750.11273
772.09402
302.24471
498.90059
226.95188
90.97733
838.83772
906.82447
1135.27069
1283.30753
1357.32856
1505.36518
1653.40521
1801.44210

HRMS of **1c**

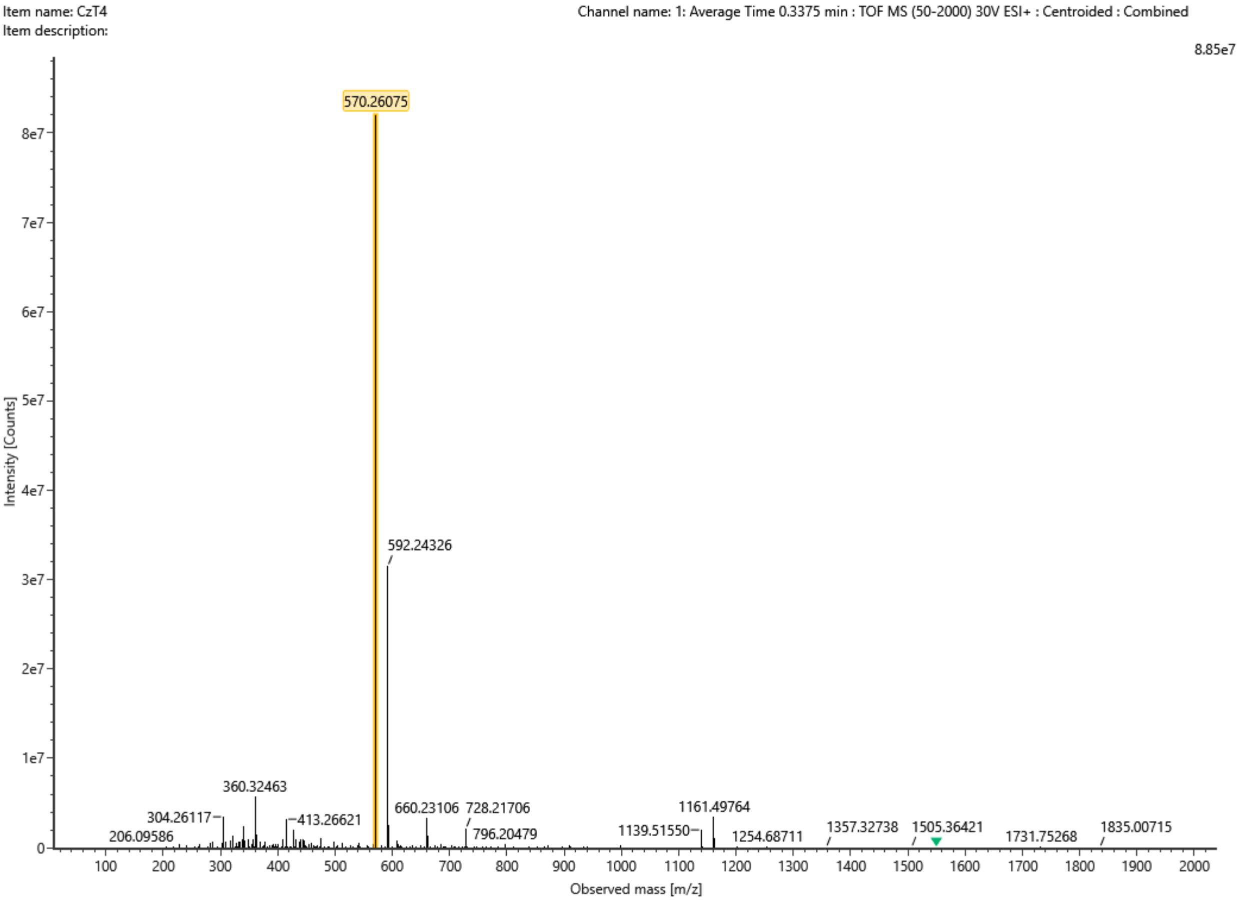

Item name: CzT4
Item description:
Channel name: 1: Average Time 0.3375 min : TOF MS (50-2000) 30V ESI+ : Centroided : Combined
8.85e7
570.26075
592.24326
360.32463
304.26117
413.26621
206.09586
660.23106
728.21706
796.20479
1139.51550
1161.49764
1254.68711
1357.32738
1505.36421
1731.75268
1835.00715
Intensity [Counts]
Observed mass [m/z]

HRMS of **1d**

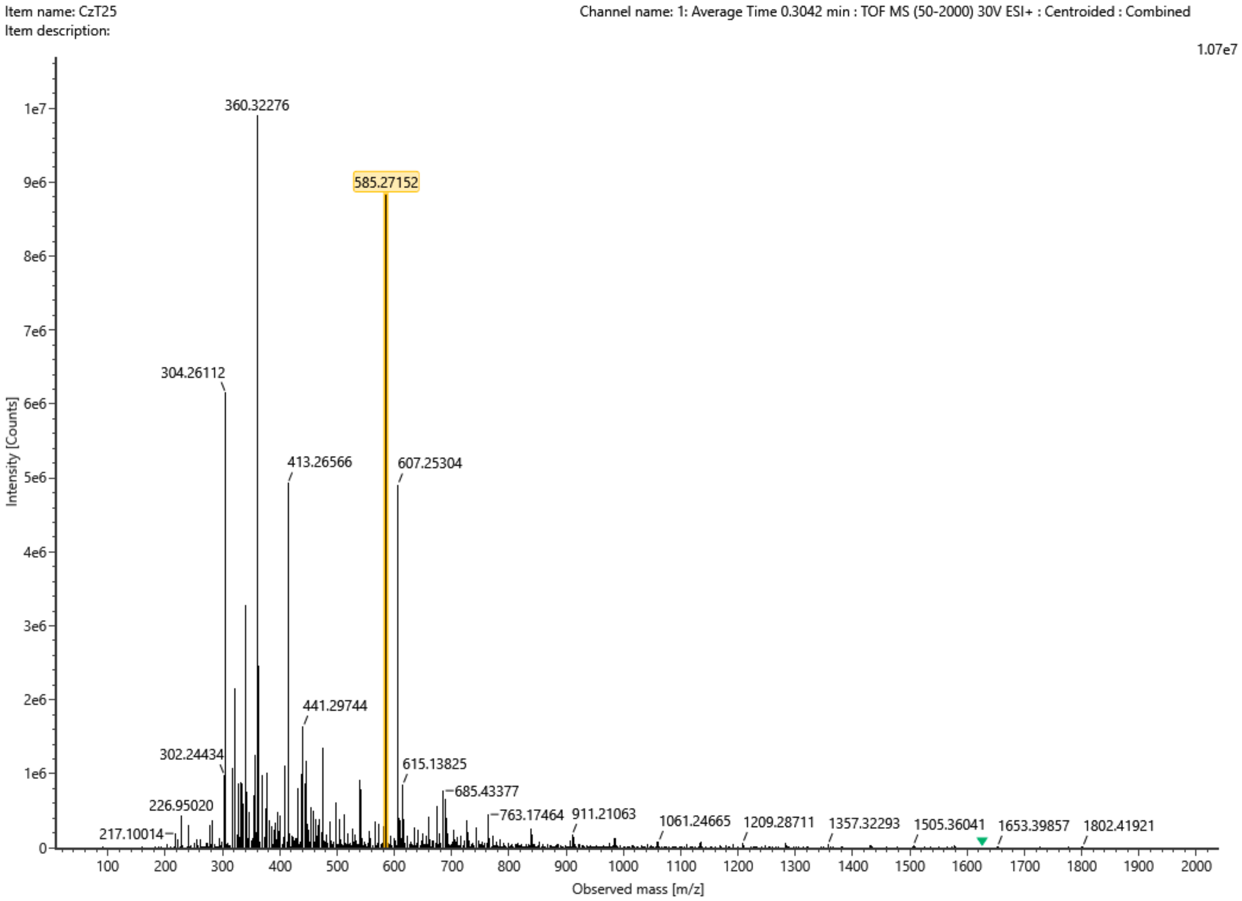

Item name: CzT25
Item description:
Channel name: 1: Average Time 0.3042 min : TOF MS (50-2000) 30V ESI+ : Centroided : Combined
1.07e7
360.32276
585.27152
304.26112
413.26566
607.25304
441.29744
302.24434
615.13825
685.43377
226.95020
763.17464
911.21063
217.10014
1061.24665
1209.28711
1357.32293
1505.36041
1653.39857
1802.41921
Intensity [Counts]
Observed mass [m/z]

HRMS of **1e**

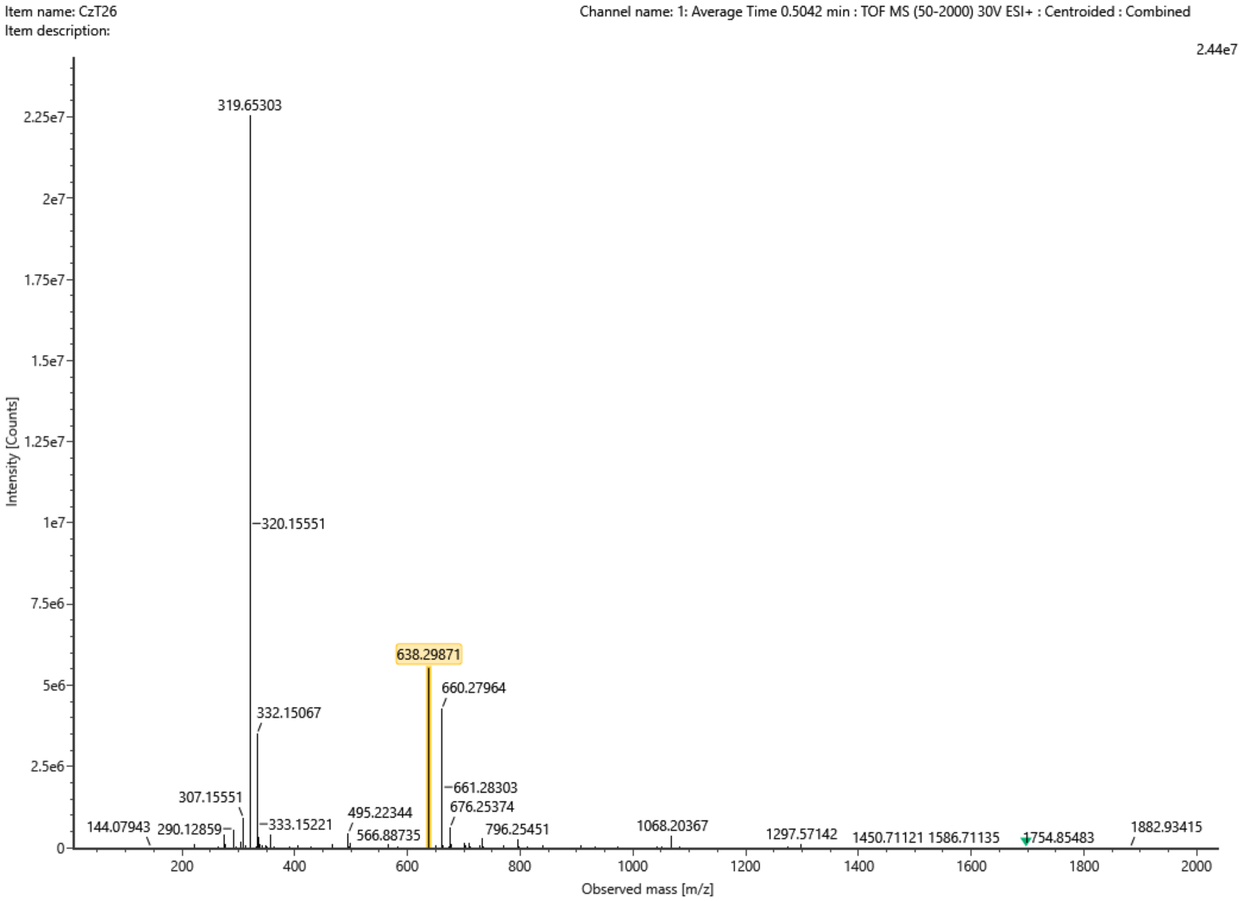

Item name: CzT26
Item description:
Channel name: 1: Average Time 0.5042 min : TOF MS (50-2000) 30V ESI+ : Centroided : Combined
2.44e7
319.65303
320.15551
638.29871
660.27964
332.15067
661.28303
676.25374
307.15551
495.22344
144.07943
290.12859
333.15221
566.88735
796.25451
1068.20367
1297.57142
1450.71121
1586.71135
1754.85483
1882.93415
Intensity [Counts]
Observed mass [m/z]

HRMS of **2a**

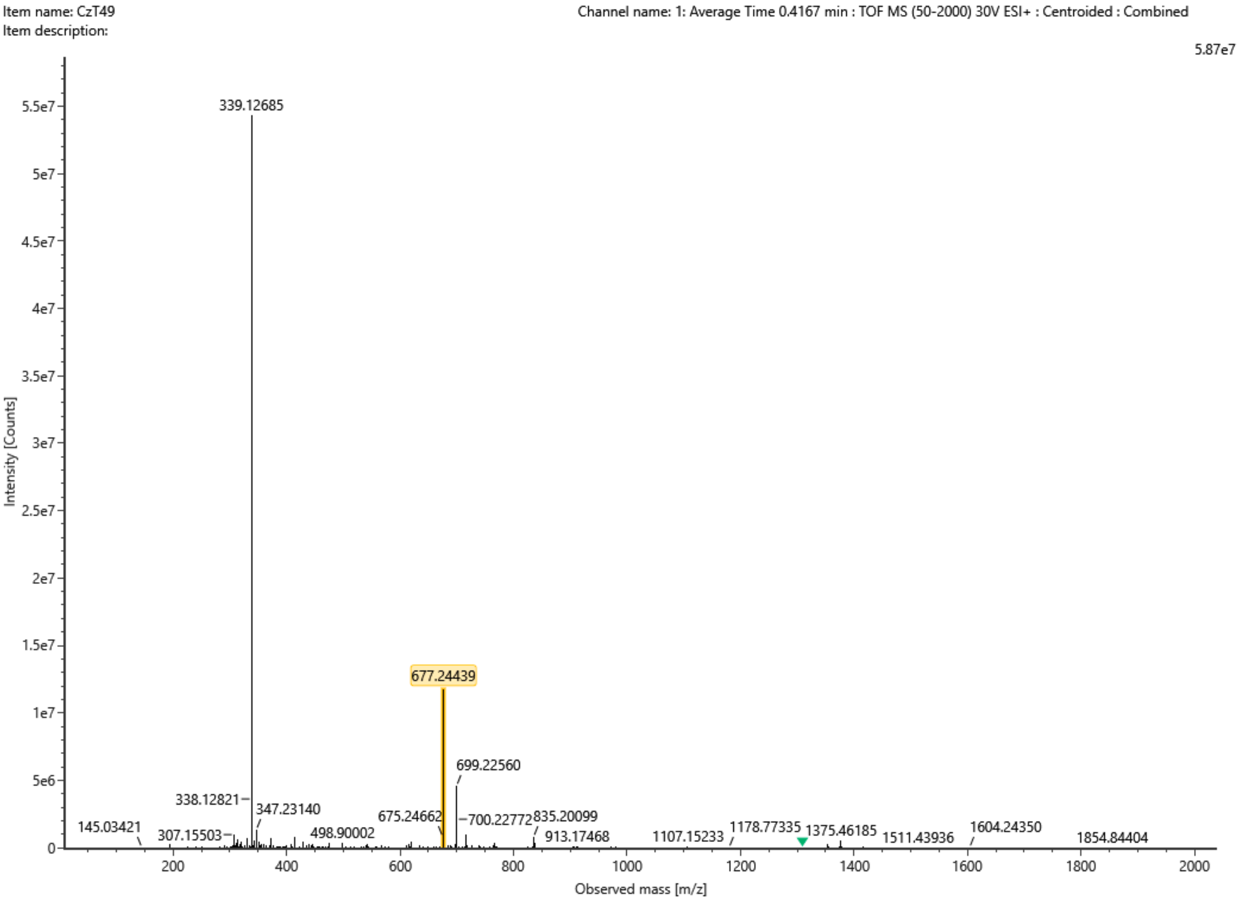

Item name: CzT49
Item description:
Channel name: 1: Average Time 0.4167 min : TOF MS (50-2000) 30V ESI+ : Centroided : Combined
5.87e7
339.12685
677.24439
699.22560
338.12821
347.23140
145.03421
307.15503
498.90002
675.24662
700.22772
835.20099
913.17468
1107.15233
1178.77335
1375.46185
1511.43936
1604.24350
1854.84404
Intensity [Counts]
Observed mass [m/z]

HRMS of **2b**

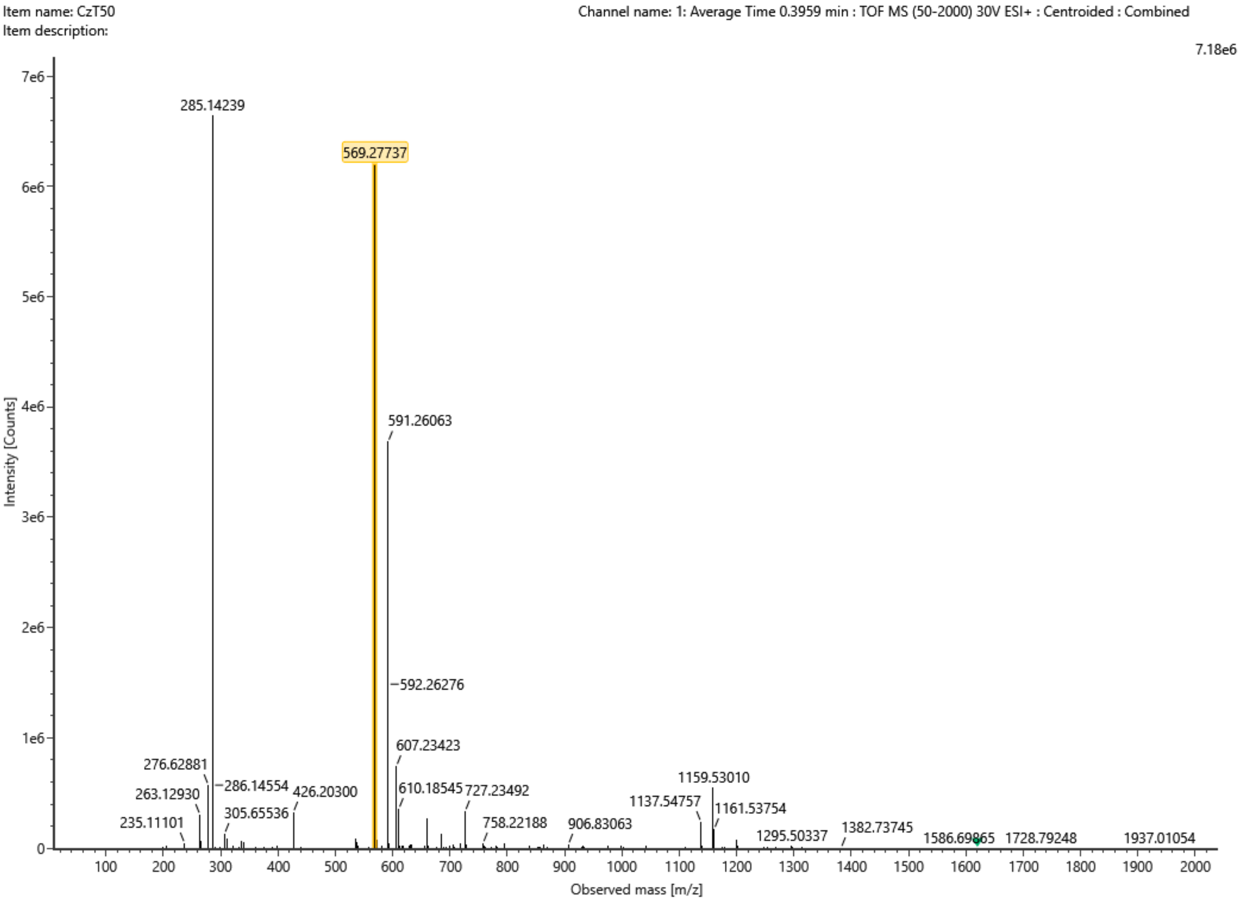

Item name: CzT50
Item description:
Channel name: 1: Average Time 0.3959 min : TOF MS (50-2000) 30V ESI+ : Centroided : Combined
7.18e6
285.14239
569.27737
591.26063
592.26276
607.23423
276.62881
286.14554
263.12930
426.20300
610.18545
727.23492
235.11101
305.65536
758.22188
906.83063
1159.53010
1137.54757
1161.53754
1295.50337
1382.73745
1586.69865
1728.79248
1937.01054
Intensity [Counts]
Observed mass [m/z]

HRMS of **2c**

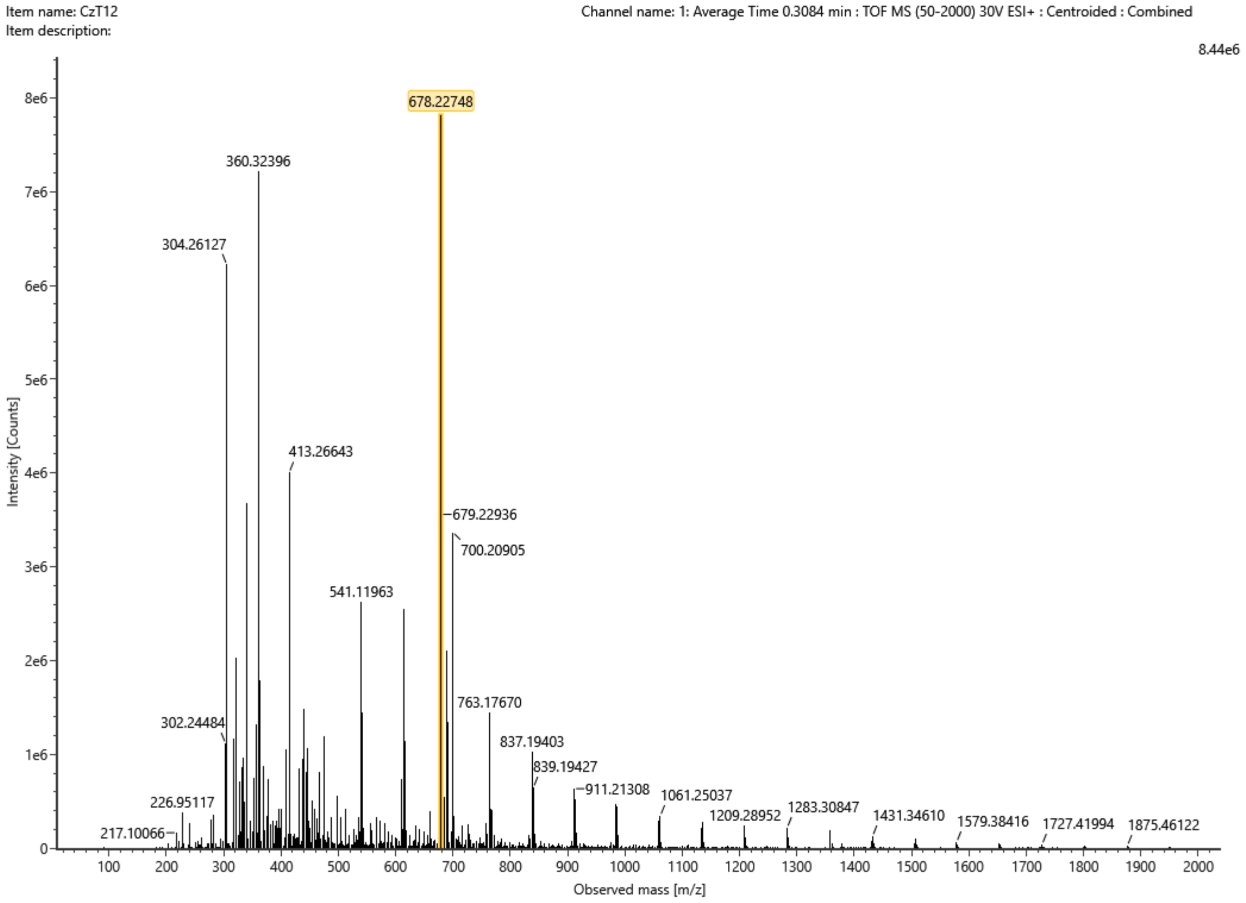

Item name: CzT12
Item description:
Channel name: 1: Average Time 0.3084 min : TOF MS (50-2000) 30V ESI+ : Centroided : Combined
8.44e6
678.22748
360.32396
304.26127
413.26643
679.22936
700.20905
541.11963
763.17670
302.24484
837.19403
839.19427
911.21308
1061.25037
226.95117
1209.28952
1283.30847
1431.34610
1579.38416
1727.41994
1875.46122
217.10066
Intensity [Counts]
Observed mass [m/z]

HRMS of **2d**

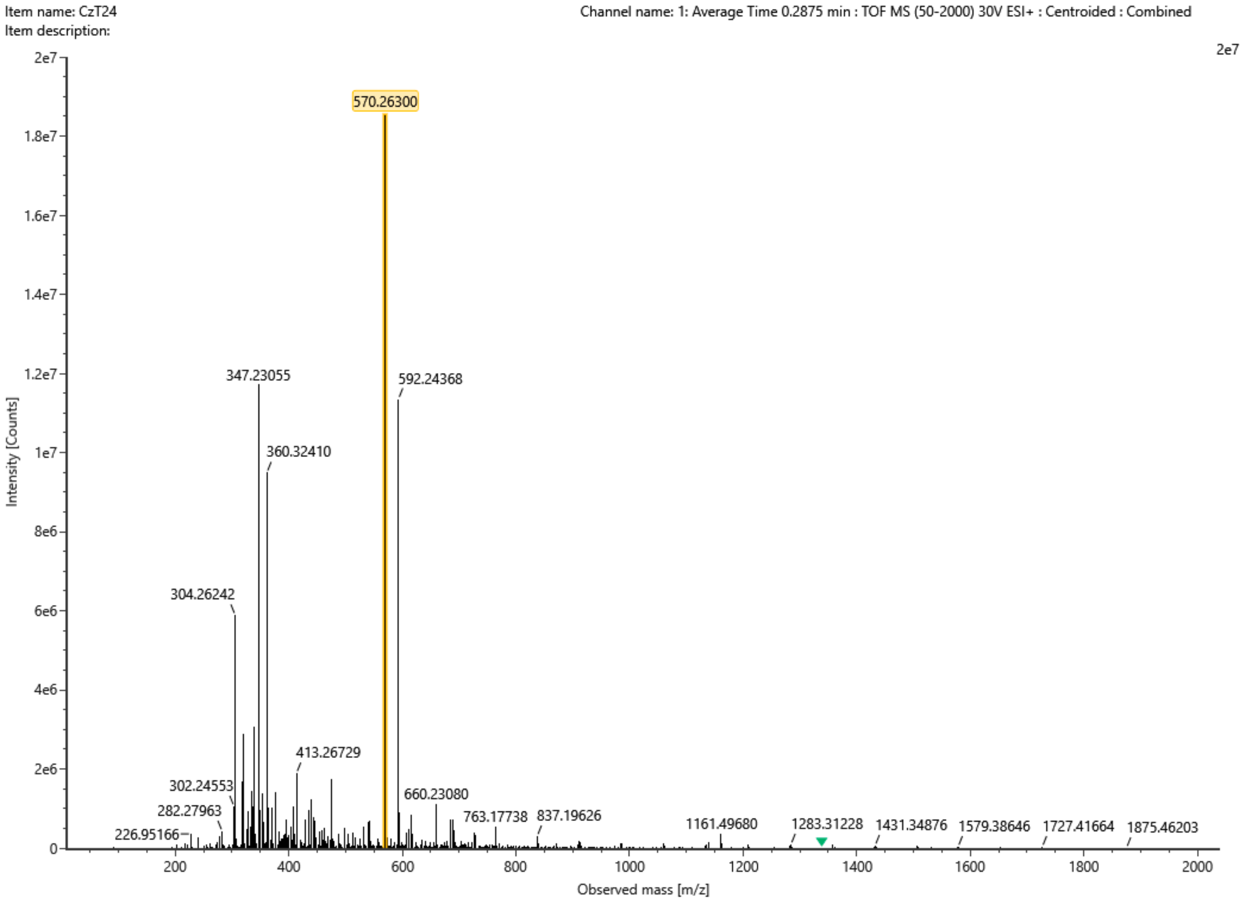

Item name: CzT24
Item description:
Channel name: 1: Average Time 0.2875 min : TOF MS (50-2000) 30V ESI+ : Centroided : Combined
2e7
570.26300
347.23055
592.24368
360.32410
304.26242
413.26729
302.24553
660.23080
282.27963
763.17738
837.19626
226.95166
1161.49680
1283.31228
1431.34876
1579.38646
1727.41664
1875.46203
Intensity [Counts]
Observed mass [m/z]

HRMS of **3a**

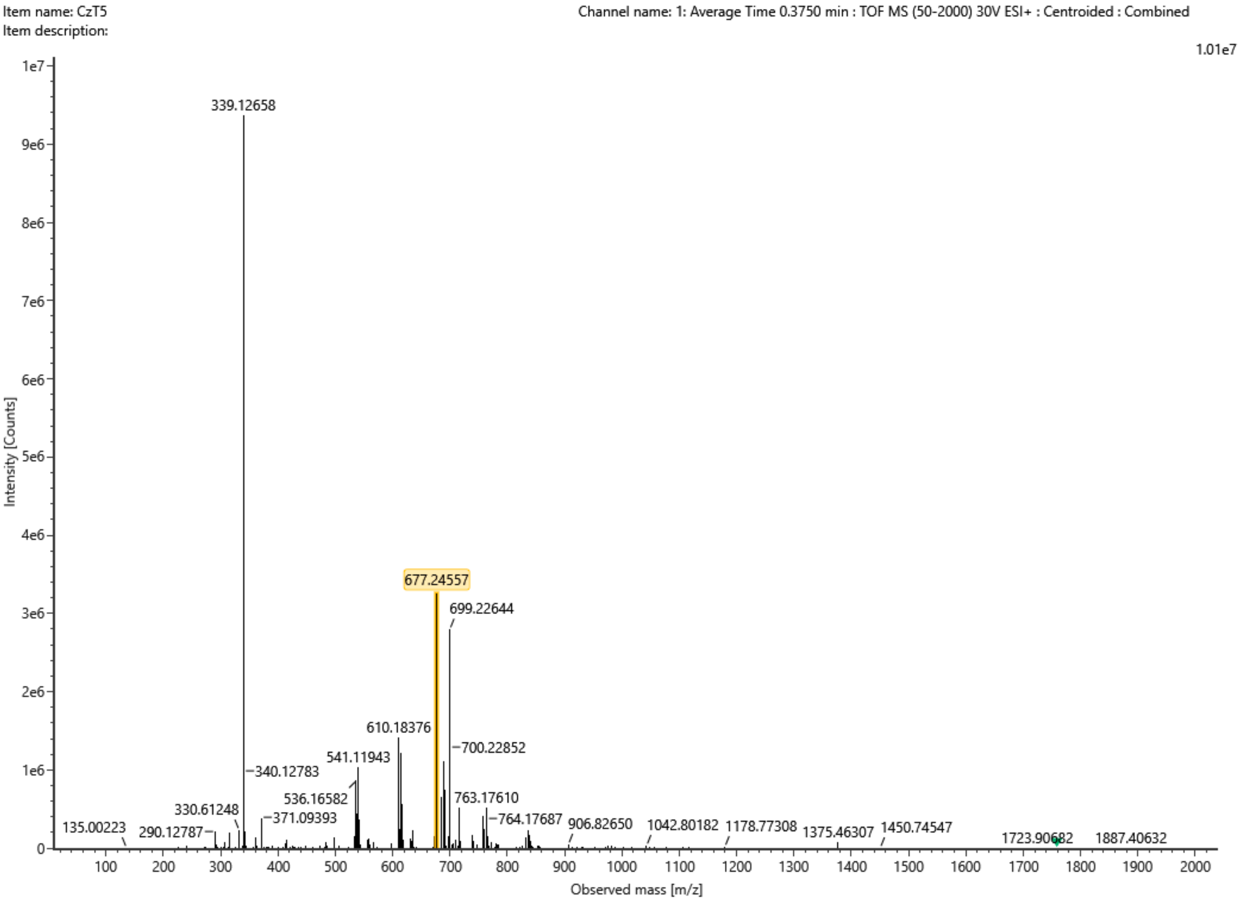

Item name: CzT5
Item description:
Channel name: 1: Average Time 0.3750 min : TOF MS (50-2000) 30V ESI+ : Centroided : Combined
1.01e7
339.12658
677.24557
699.22644
610.18376
700.22852
541.11943
340.12783
536.16582
763.17610
330.61248
371.09393
764.17687
135.00223
290.12787
906.82650
1042.80182
1178.77308
1375.46307
1450.74547
1723.90682
1887.40632
Intensity [Counts]
Observed mass [m/z]

HRMS of **3b**

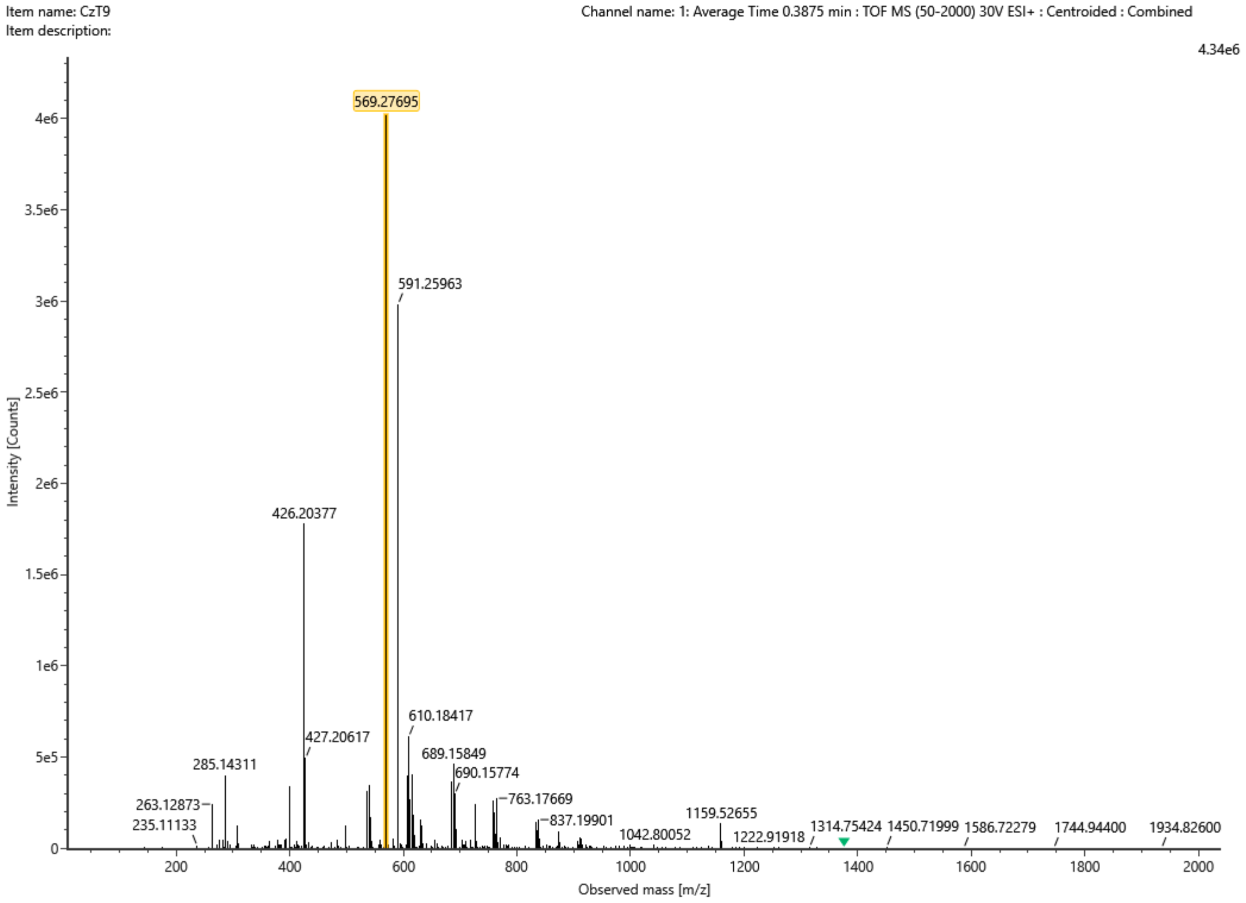

Item name: CzT9
Item description:
Channel name: 1: Average Time 0.3875 min : TOF MS (50-2000) 30V ESI+ : Centroided : Combined
4.34e6
569.27695
591.25963
426.20377
610.18417
427.20617
689.15849
285.14311
690.15774
763.17669
263.12873
837.19901
235.11133
1159.52655
1042.80052
1222.91918
1314.75424
1450.71999
1586.72279
1744.94400
1934.82600
Intensity [Counts]
Observed mass [m/z]

HRMS of **3c**

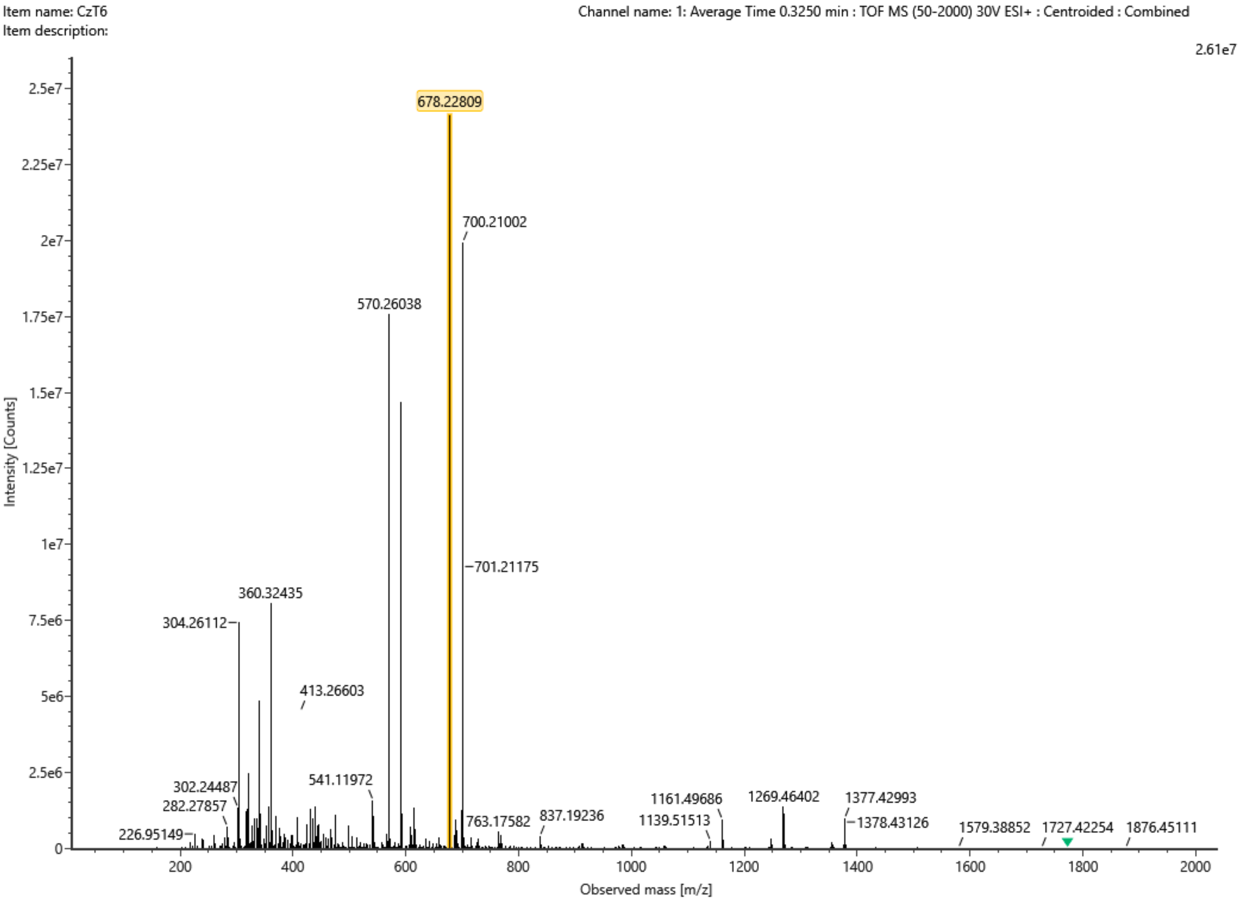

Item name: CzT6
Item description:
Channel name: 1: Average Time 0.3250 min : TOF MS (50-2000) 30V ESI+ : Centroided : Combined
2.61e7
678.22809
700.21002
570.26038
701.21175
360.32435
304.26112
413.26603
541.11972
302.24487
282.27857
226.95149
763.17582
837.19236
1161.49686
1139.51513
1269.46402
1377.42993
1378.43126
1579.38852
1727.42254
1876.45111
Intensity [Counts]
Observed mass [m/z]

HRMS of **3d**

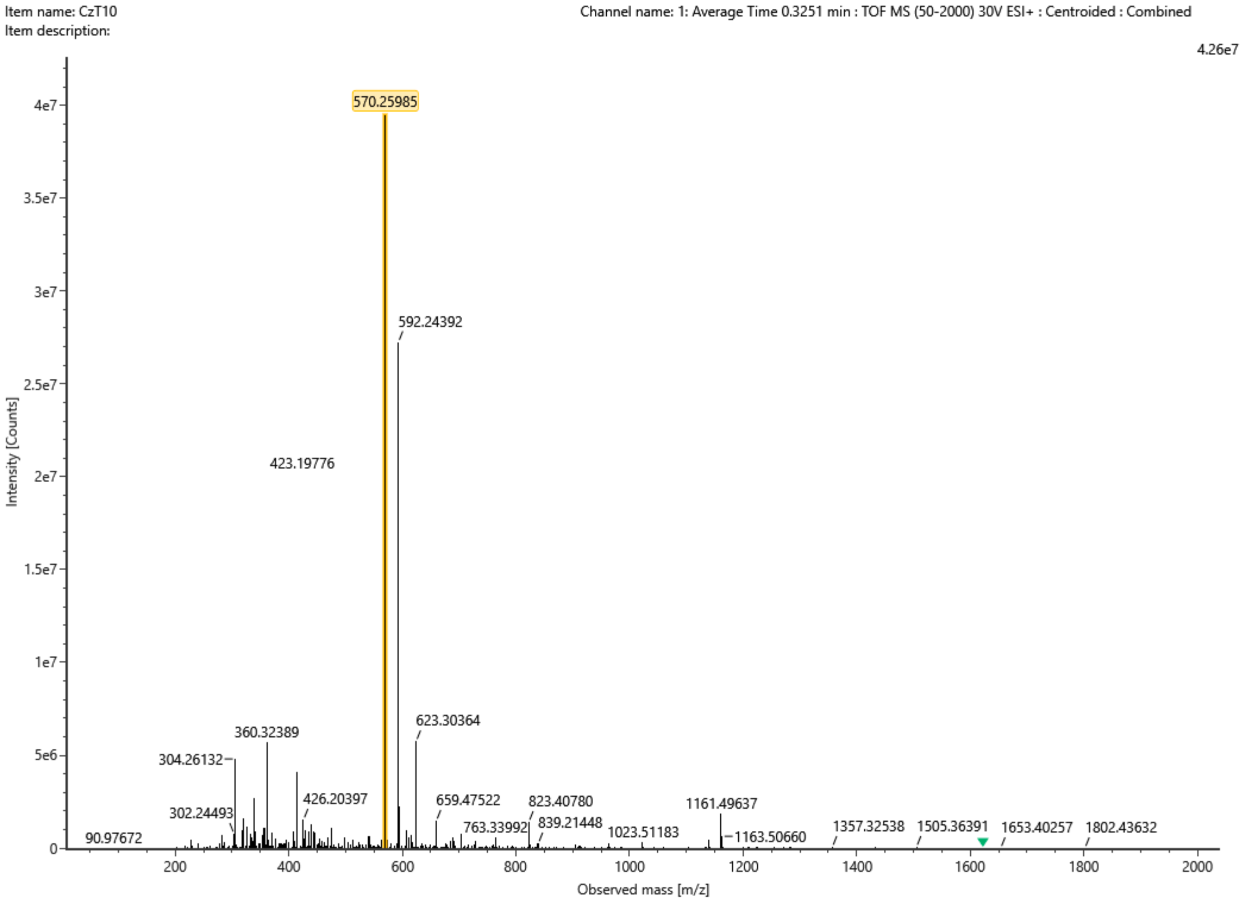

Item name: CzT10
Item description:
Channel name: 1: Average Time 0.3251 min : TOF MS (50-2000) 30V ESI+ : Centroided : Combined
4.26e7
570.25985
592.24392
423.19776
623.30364
360.32389
304.26132
426.20397
302.24493
659.47522
823.40780
1161.49637
763.33992
839.21448
1023.51183
1163.50660
1357.32538
1505.36391
1653.40257
1802.43632
90.97672
Intensity [Counts]
Observed mass [m/z]

HRMS of **4a**

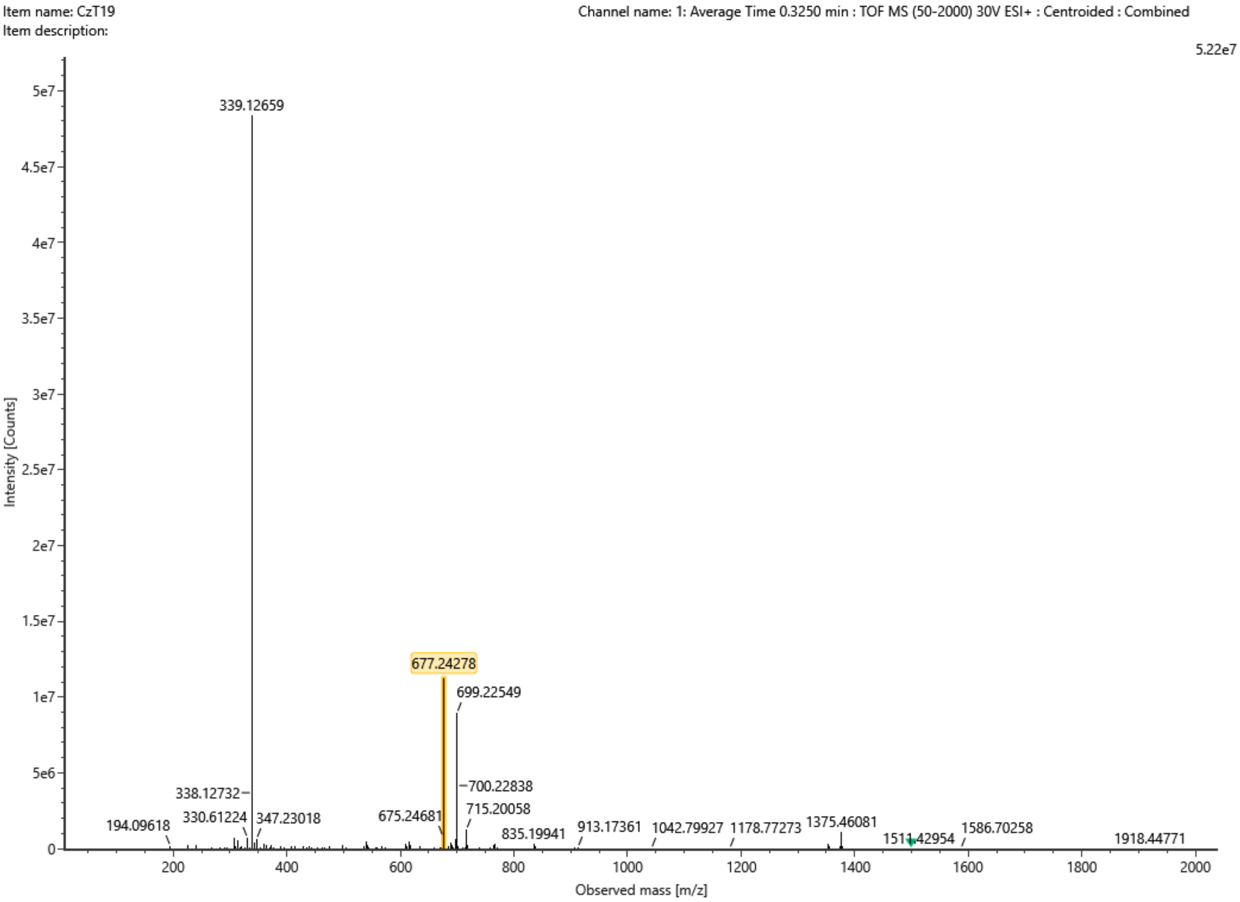

Item name: CzT19
Item description:
Channel name: 1: Average Time 0.3250 min : TOF MS (50-2000) 30V ESI+ : Centroided : Combined
5.22e7
339.12659
677.24278
699.22549
700.22838
715.20058
338.12732
330.61224
347.23018
194.09618
675.24681
835.19941
913.17361
1042.79927
1178.77273
1375.46081
1511.42954
1586.70258
1918.44771
Intensity [Counts]
Observed mass [m/z]

HRMS of **4b**

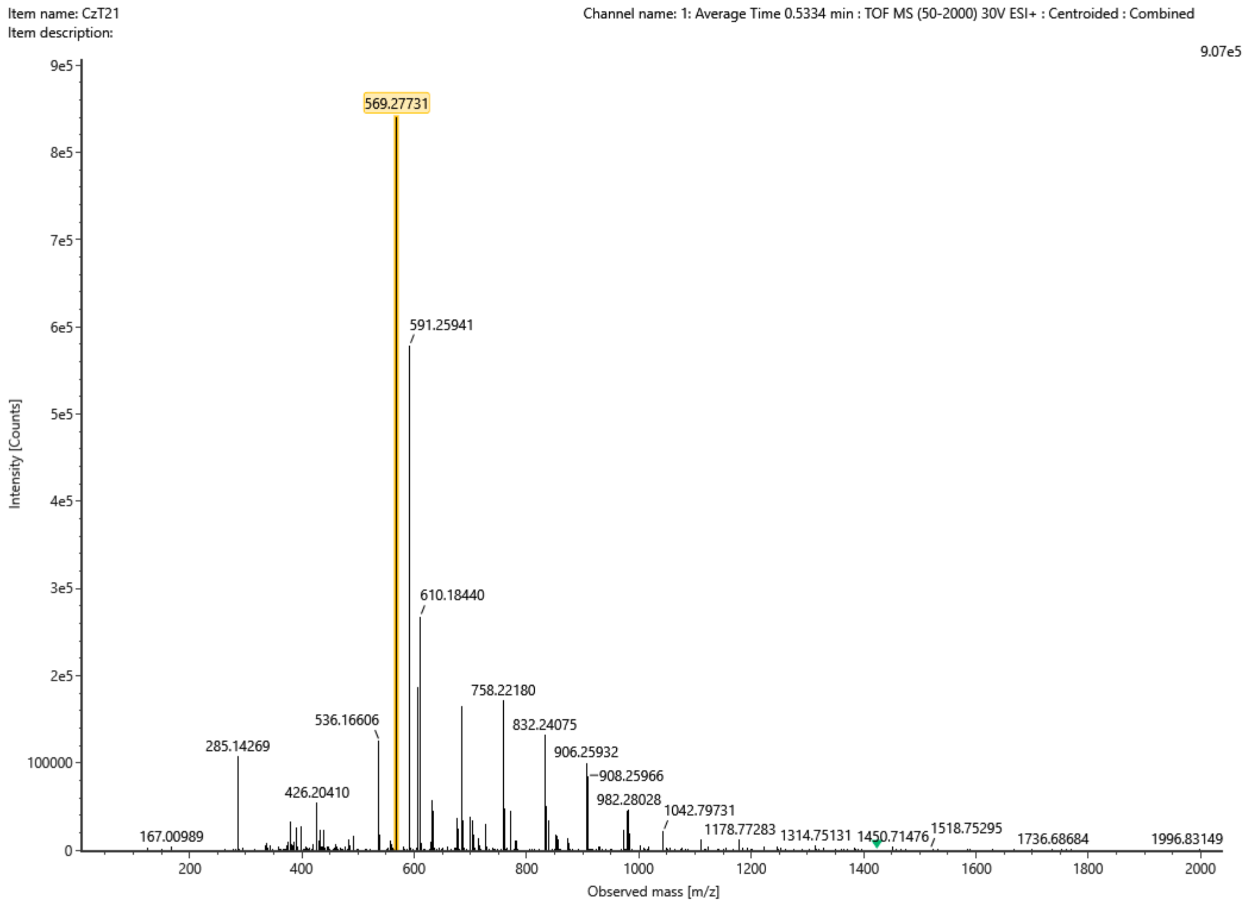

Item name: CzT21
Item description:
Channel name: 1: Average Time 0.5334 min : TOF MS (50-2000) 30V ESI+ : Centroided : Combined
9.07e5
569.27731
591.25941
610.18440
758.22180
832.24075
536.16606
285.14269
906.25932
908.25966
426.20410
982.28028
1042.79731
167.00989
1178.77283
1314.75131
1450.71476
1518.75295
1736.68684
1996.83149
Intensity [Counts]
Observed mass [m/z]

HRMS of **4d**

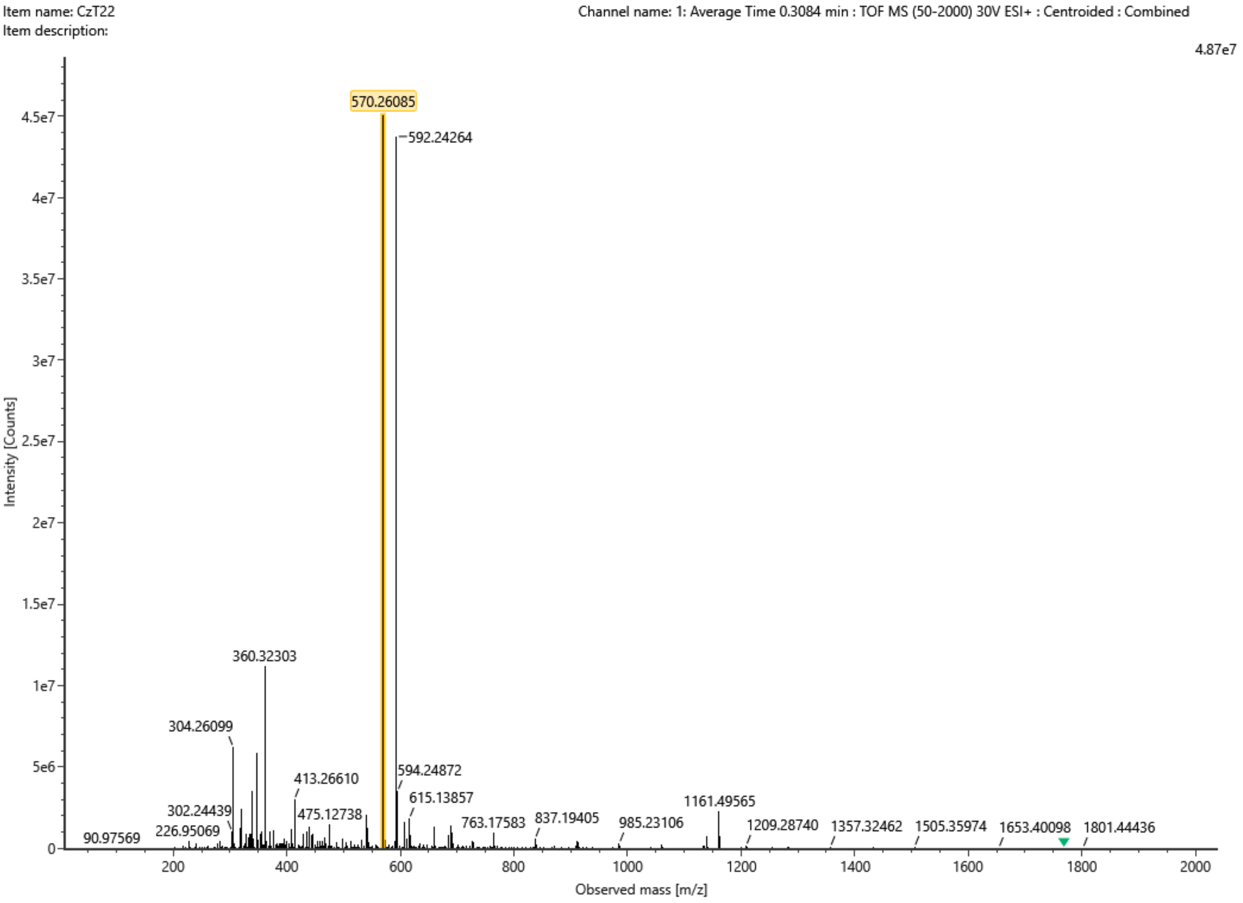

Item name: CzT22
Item description:
Channel name: 1: Average Time 0.3084 min : TOF MS (50-2000) 30V ESI+ : Centroided : Combined
4.87e7
570.26085
592.24264
360.32303
304.26099
413.26610
594.24872
615.13857
302.24439
226.95069
90.97569
475.12738
763.17583
837.19405
985.23106
1161.49565
1209.28740
1357.32462
1505.35974
1653.40098
1801.44436
Intensity [Counts]
Observed mass [m/z]

HRMS of **23a**

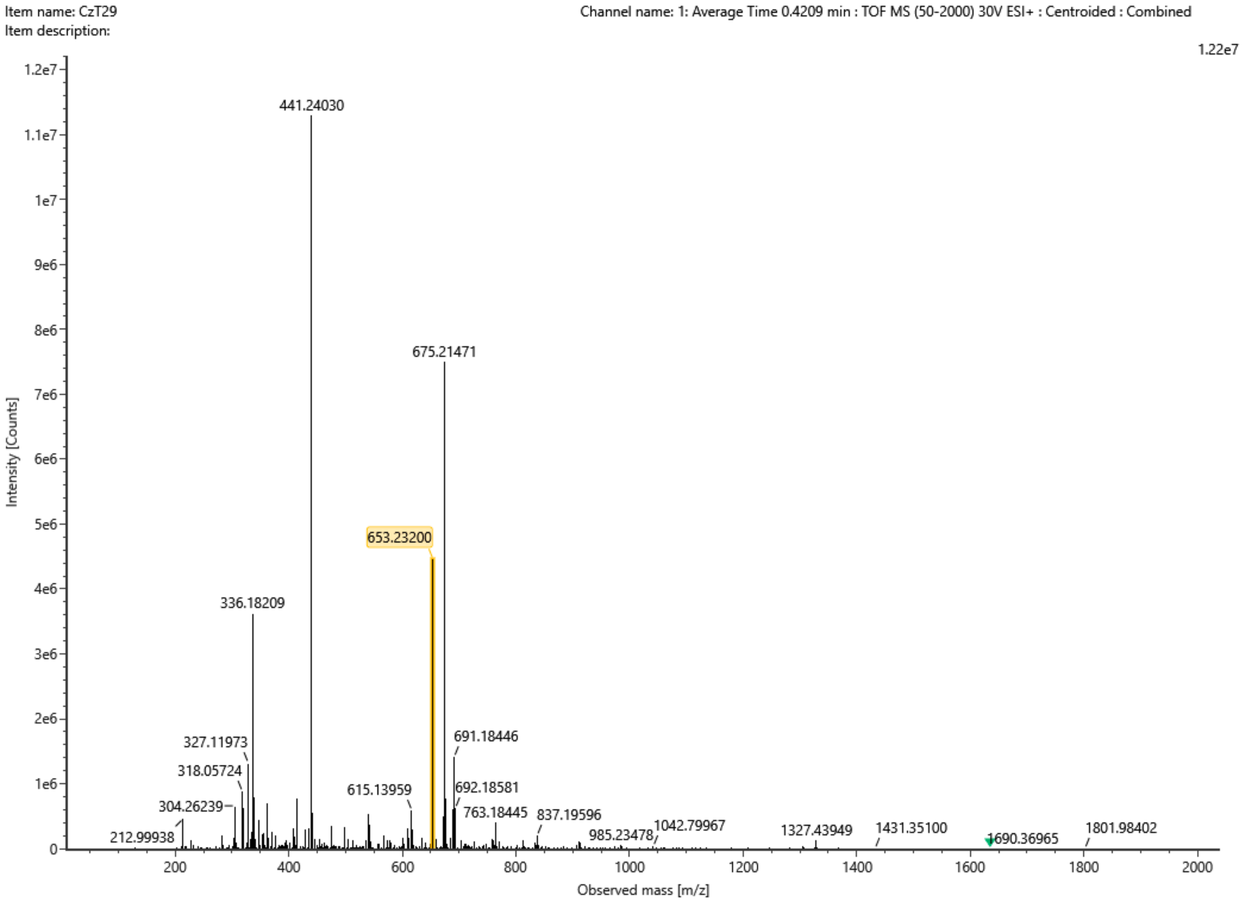

Item name: CzT29
Item description:
Channel name: 1: Average Time 0.4209 min : TOF MS (50-2000) 30V ESI+ : Centroided : Combined
1.22e7
441.24030
675.21471
653.23200
336.18209
691.18446
327.11973
318.05724
692.18581
615.13959
304.26239
763.18445
837.19596
212.99938
985.23478
1042.79967
1327.43949
1431.35100
1690.36965
1801.98402
Intensity [Counts]
Observed mass [m/z]

HRMS of **23b**

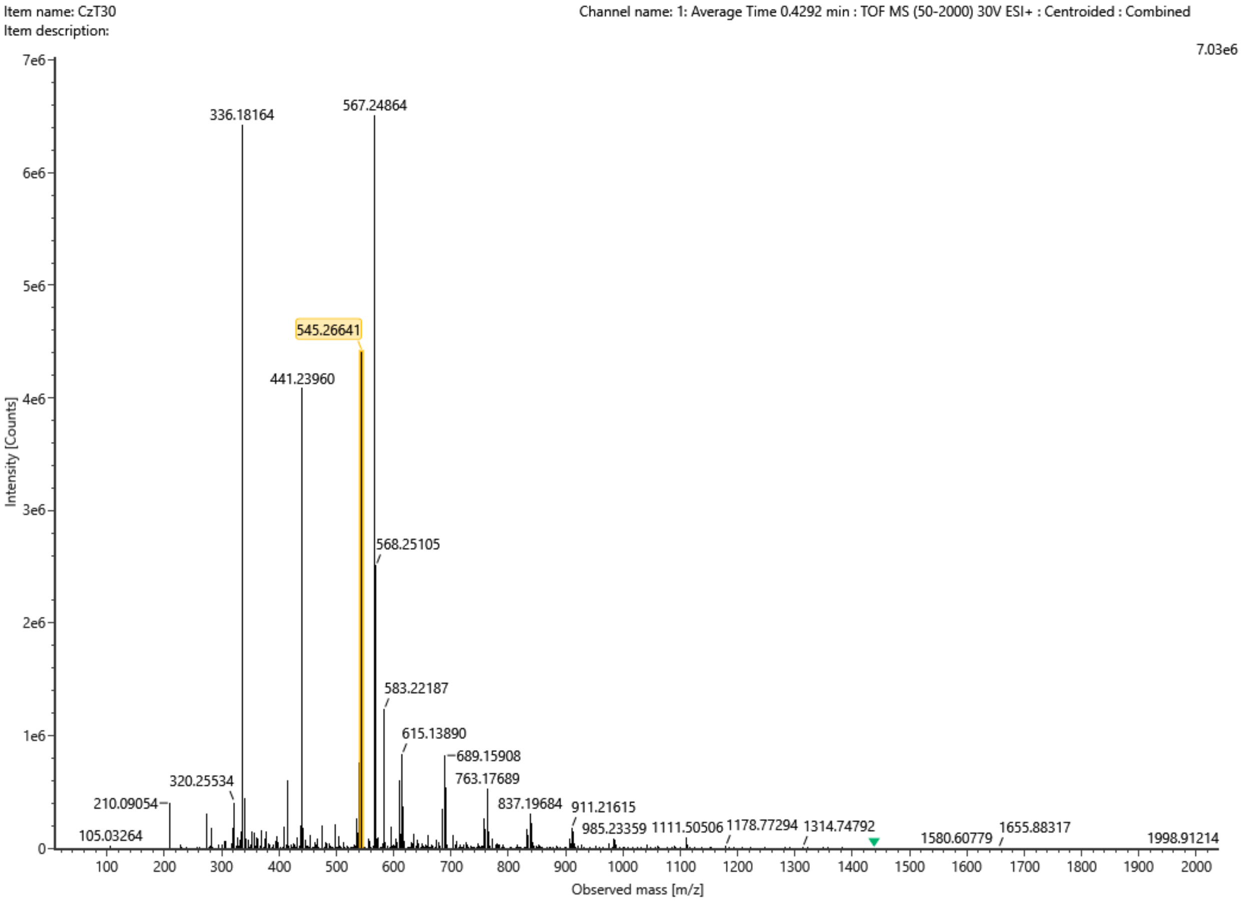

Item name: CzT30
Item description:
Channel name: 1: Average Time 0.4292 min : TOF MS (50-2000) 30V ESI+ : Centroided : Combined
7.03e6
336.18164
567.24864
545.26641
441.23960
568.25105
583.22187
615.13890
689.15908
763.17689
837.19684
911.21615
985.23359
1111.50506
1178.77294
1314.74792
1580.60779
1655.88317
1998.91214
320.25534
210.09054
105.03264
Intensity [Counts]
Observed mass [m/z]

HRMS of **33b**

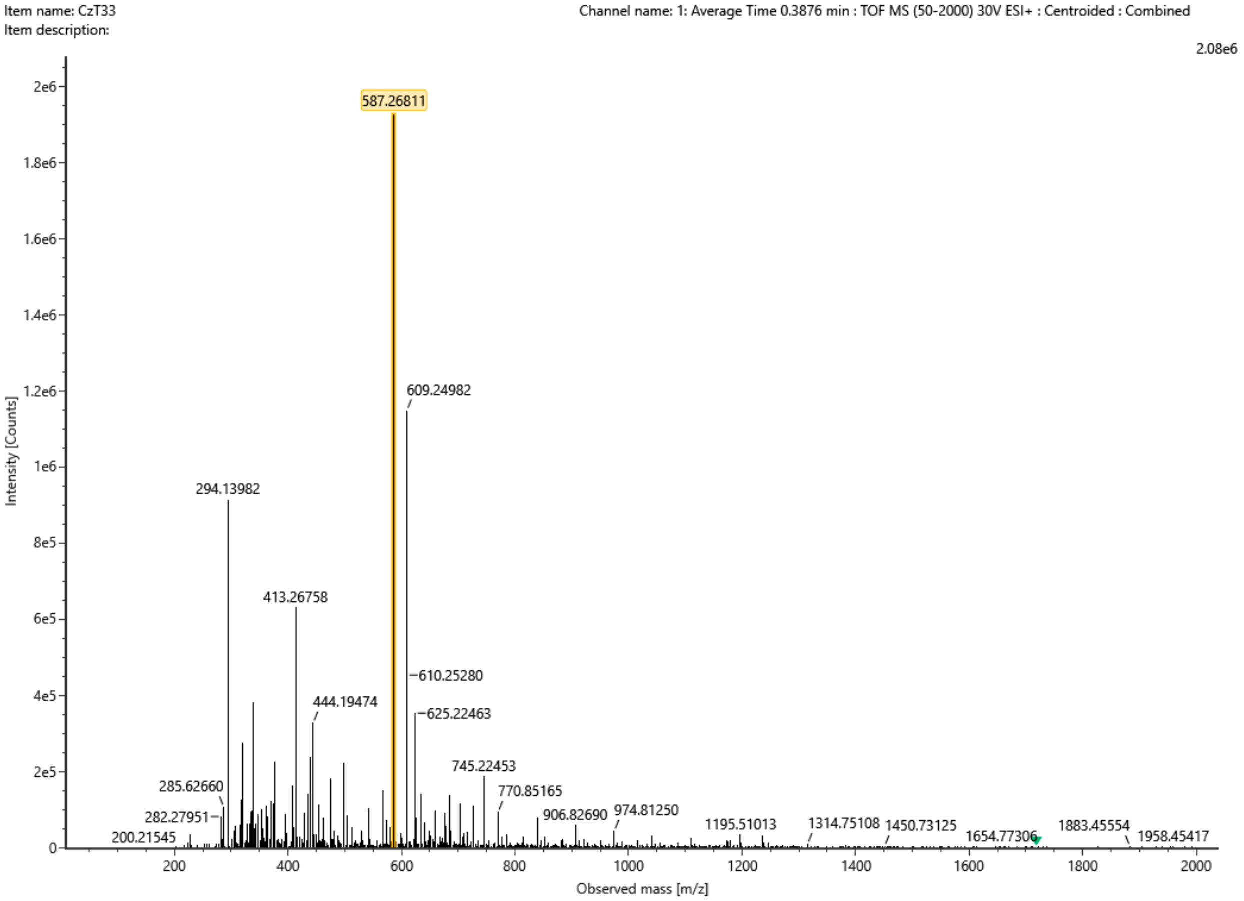
Item name: CzT33
Item description:
Channel name: 1: Average Time 0.3876 min : TOF MS (50-2000) 30V ESI+ : Centroided : Combined
2.08e6
587.26811
609.24982
294.13982
413.26758
610.25280
444.19474
625.22463
745.22453
285.62660
770.85165
282.27951
906.82690
974.81250
1195.51013
1314.75108
1450.73125
200.21545
1654.77306
1883.45554
1958.45417
Intensity [Counts]
Observed mass [m/z]

HRMS of **36a**

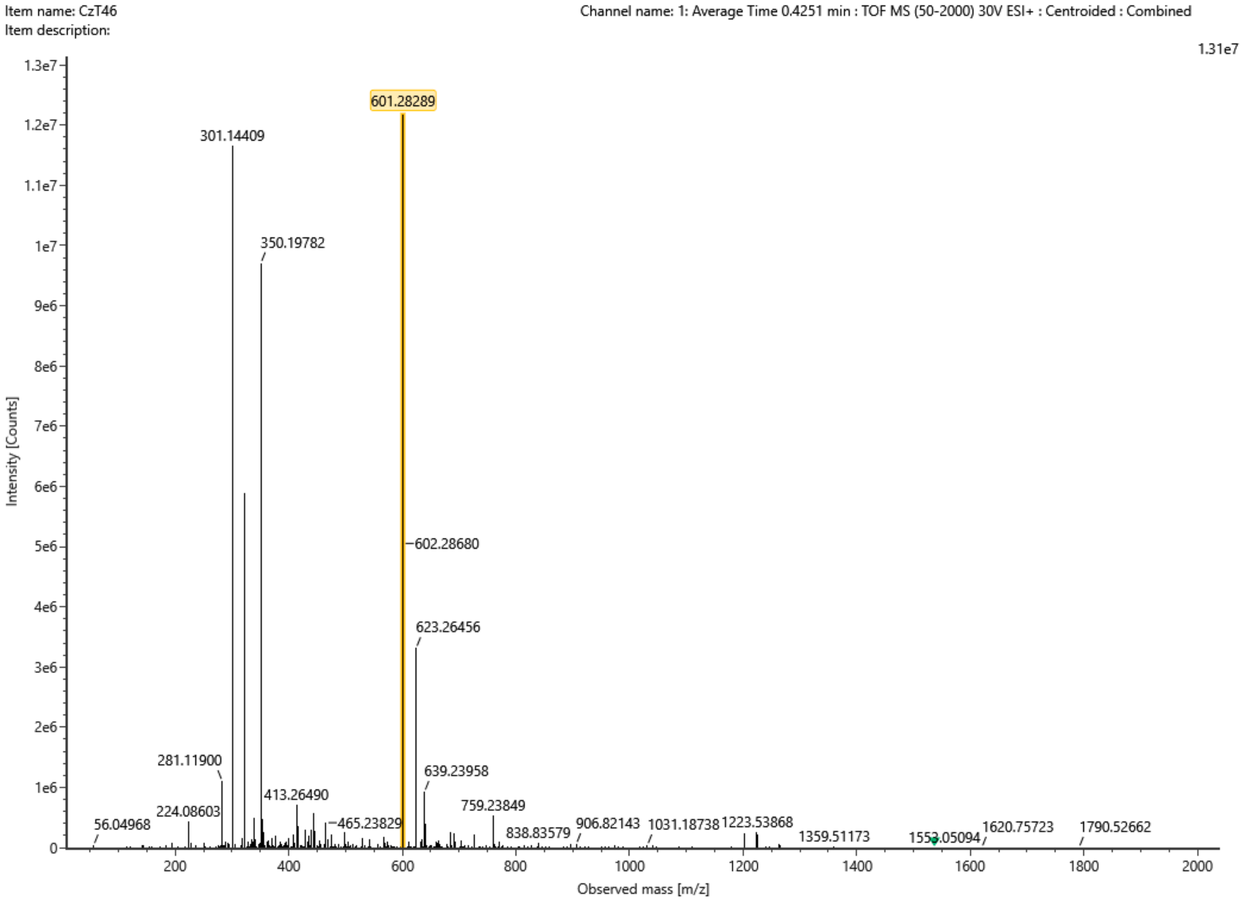

Item name: CzT46
Item description:
Channel name: 1: Average Time 0.4251 min : TOF MS (50-2000) 30V ESI+ : Centroided : Combined
1.31e7
601.28289
301.14409
350.19782
602.28680
623.26456
281.11900
639.23958
413.26490
224.08603
759.23849
56.04968
465.23829
838.83579
906.82143
1031.18738
1223.53868
1359.51173
1620.75723
1790.52662
Intensity [Counts]
Observed mass [m/z]

HRMS of **36b**

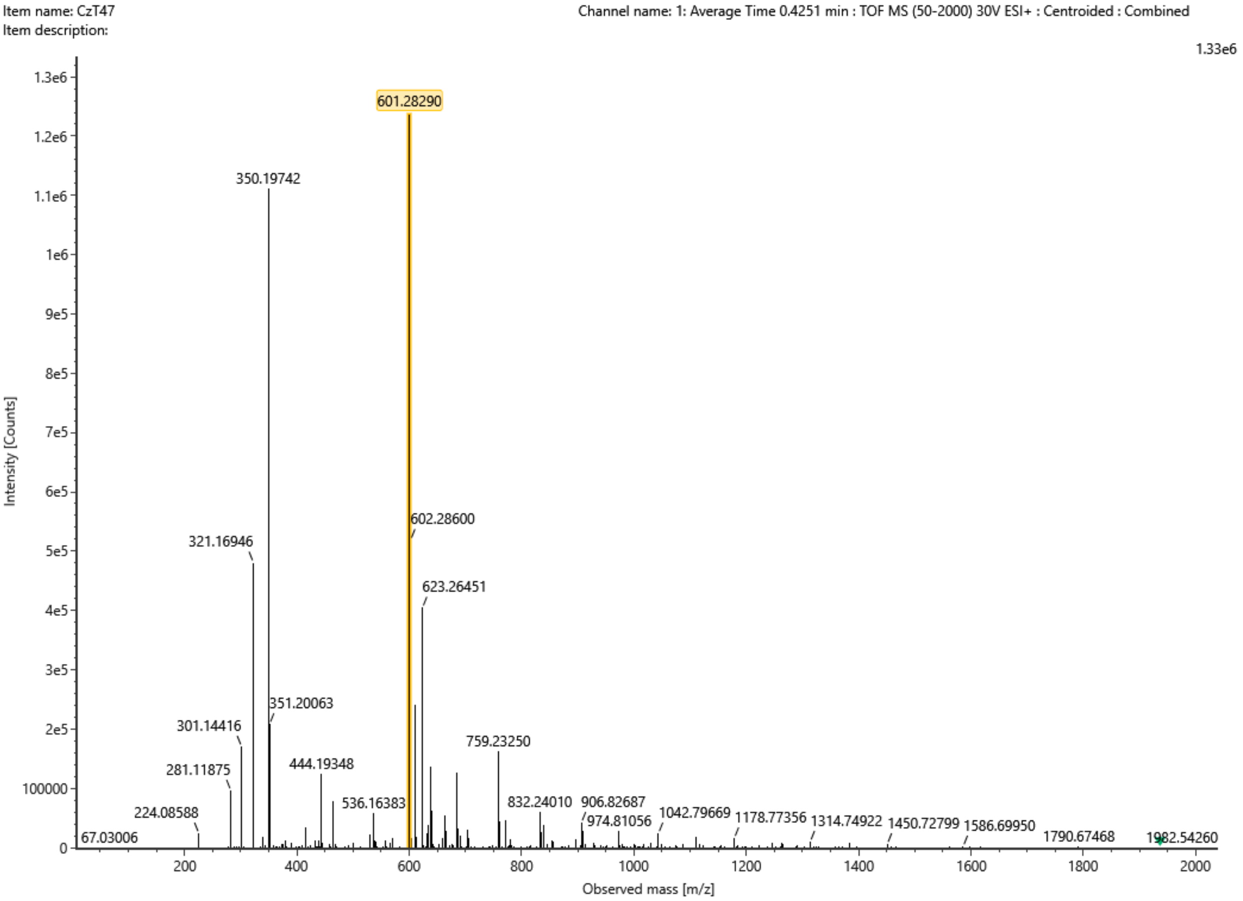
Item name: CzT47
Item description:
Channel name: 1: Average Time 0.4251 min : TOF MS (50-2000) 30V ESI+ : Centroided : Combined
1.33e6
601.28290
350.19742
602.28600
321.16946
623.26451
351.20063
301.14416
759.23250
444.19348
281.11875
536.16383
832.24010
906.82687
974.81056
1042.79669
1178.77356
1314.74922
1450.72799
1586.69950
1790.67468
1982.54260
224.08588
67.03006
Intensity [Counts]
Observed mass [m/z]

HRMS of **36c**

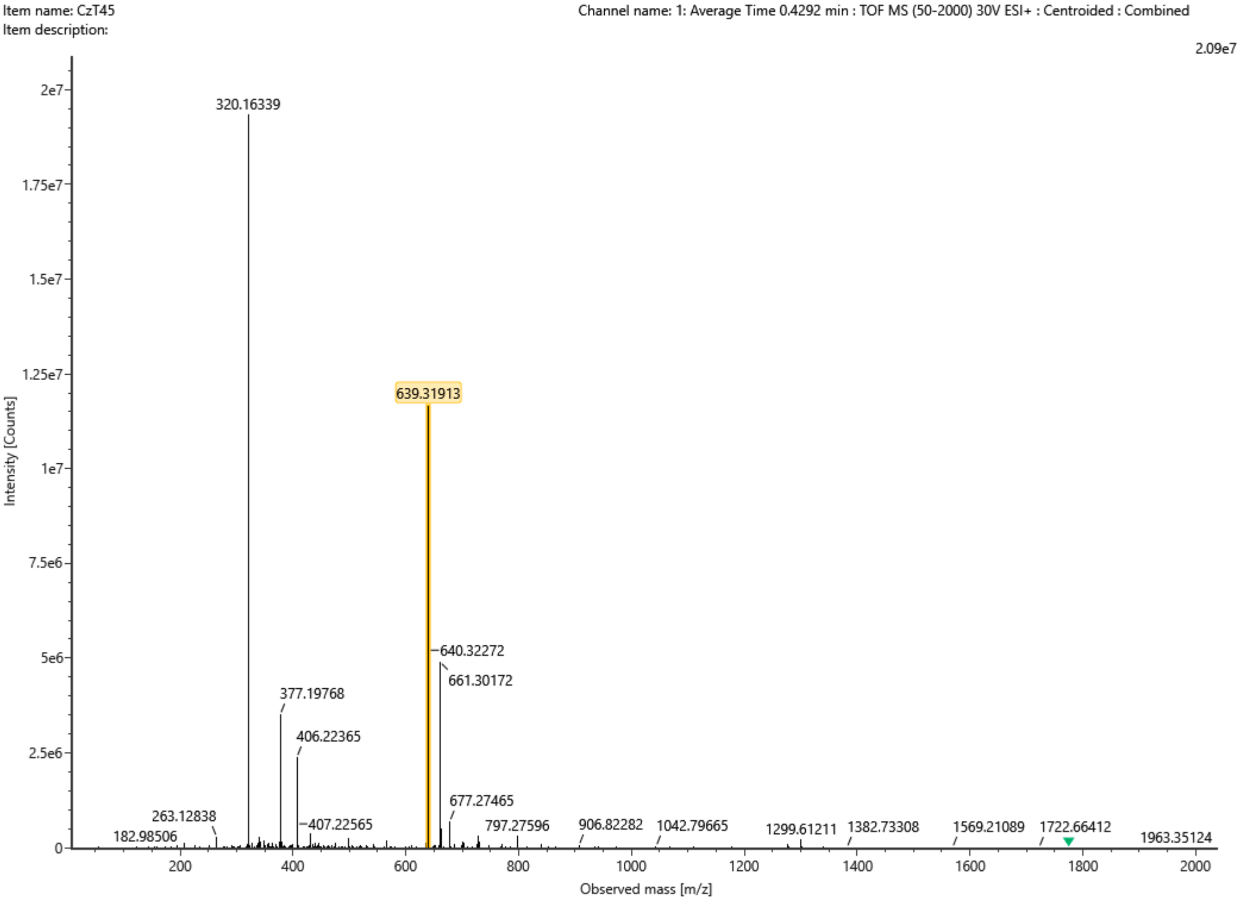

Item name: CzT45
Item description:
Channel name: 1: Average Time 0.4292 min : TOF MS (50-2000) 30V ESI+ : Centroided : Combined
2.09e7
320.16339
639.31913
640.32272
661.30172
377.19768
406.22365
407.22565
677.27465
263.12838
182.98506
797.27596
906.82282
1042.79665
1299.61211
1382.73308
1569.21089
1722.66412
1963.35124
Intensity [Counts]
Observed mass [m/z]

HRMS of **37a**

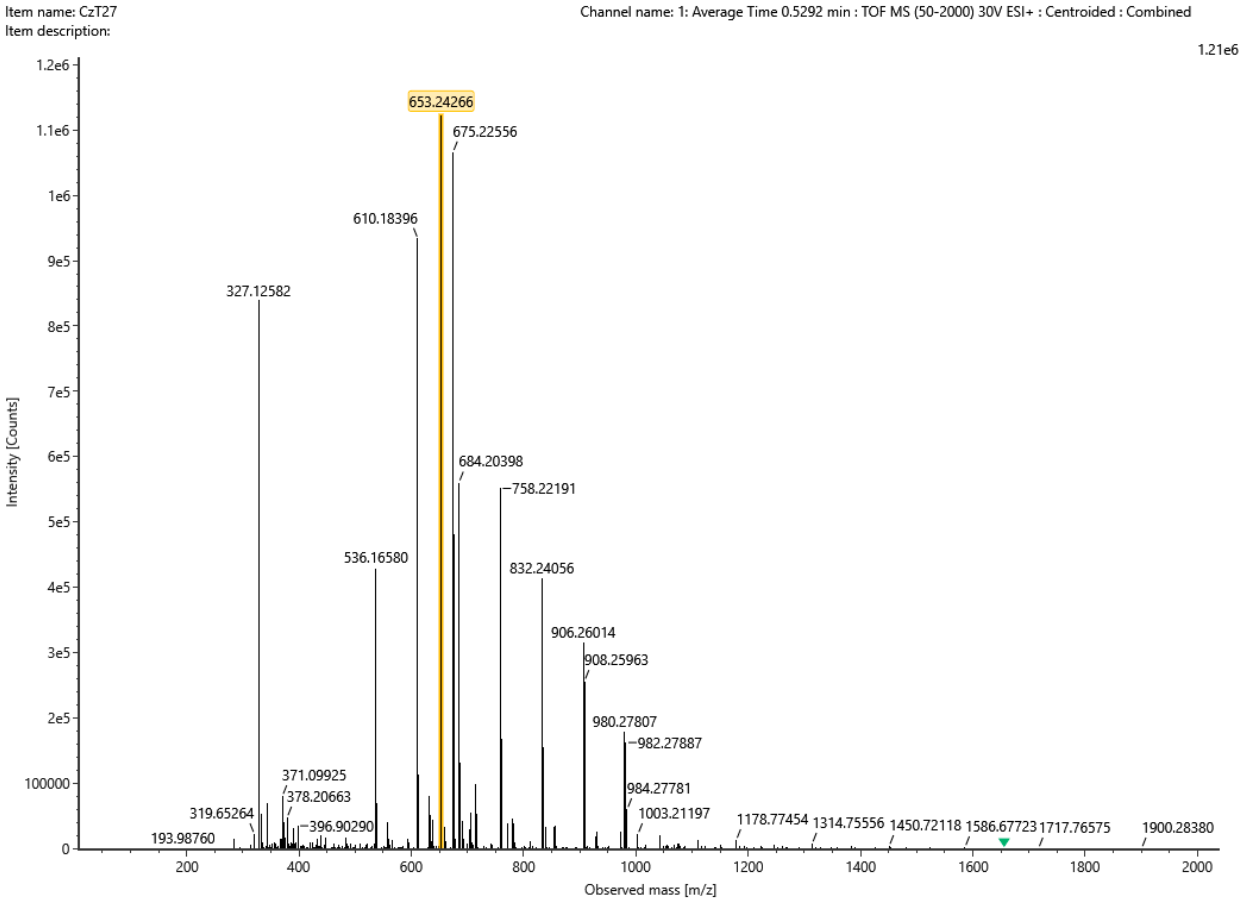

Item name: CzT27
Item description:
Channel name: 1: Average Time 0.5292 min : TOF MS (50-2000) 30V ESI+ : Centroided : Combined
1.21e6
Intensity [Counts]
Observed mass [m/z]
653.24266
675.22556
610.18396
327.12582
684.20398
758.22191
536.16580
832.24056
906.26014
908.25963
980.27807
982.27887
371.09925
378.20663
984.27781
319.65264
396.90290
1003.21197
193.98760
1178.77454
1314.75556
1450.72118
1586.67723
1717.76575
1900.28380

HRMS of **38a**

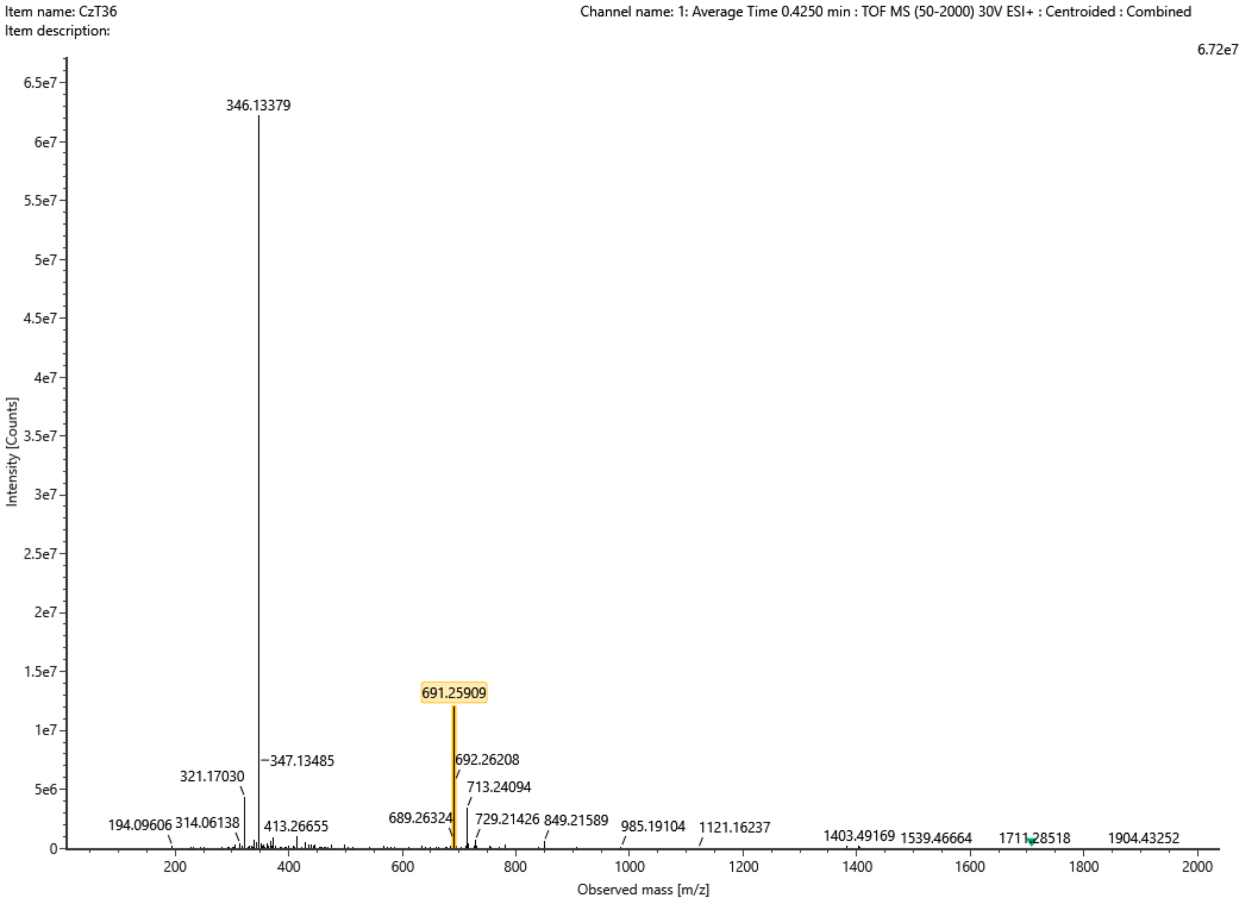
Item name: CzT36
Item description:
Channel name: 1: Average Time 0.4250 min : TOF MS (50-2000) 30V ESI+ : Centroided : Combined
6.72e7
346.13379
691.25909
347.13485
692.26208
321.17030
713.24094
194.09606
314.06138
413.26655
689.26324
729.21426
849.21589
985.19104
1121.16237
1403.49169
1539.46664
1711.28518
1904.43252
Intensity [Counts]
Observed mass [m/z]

HRMS of **38b**

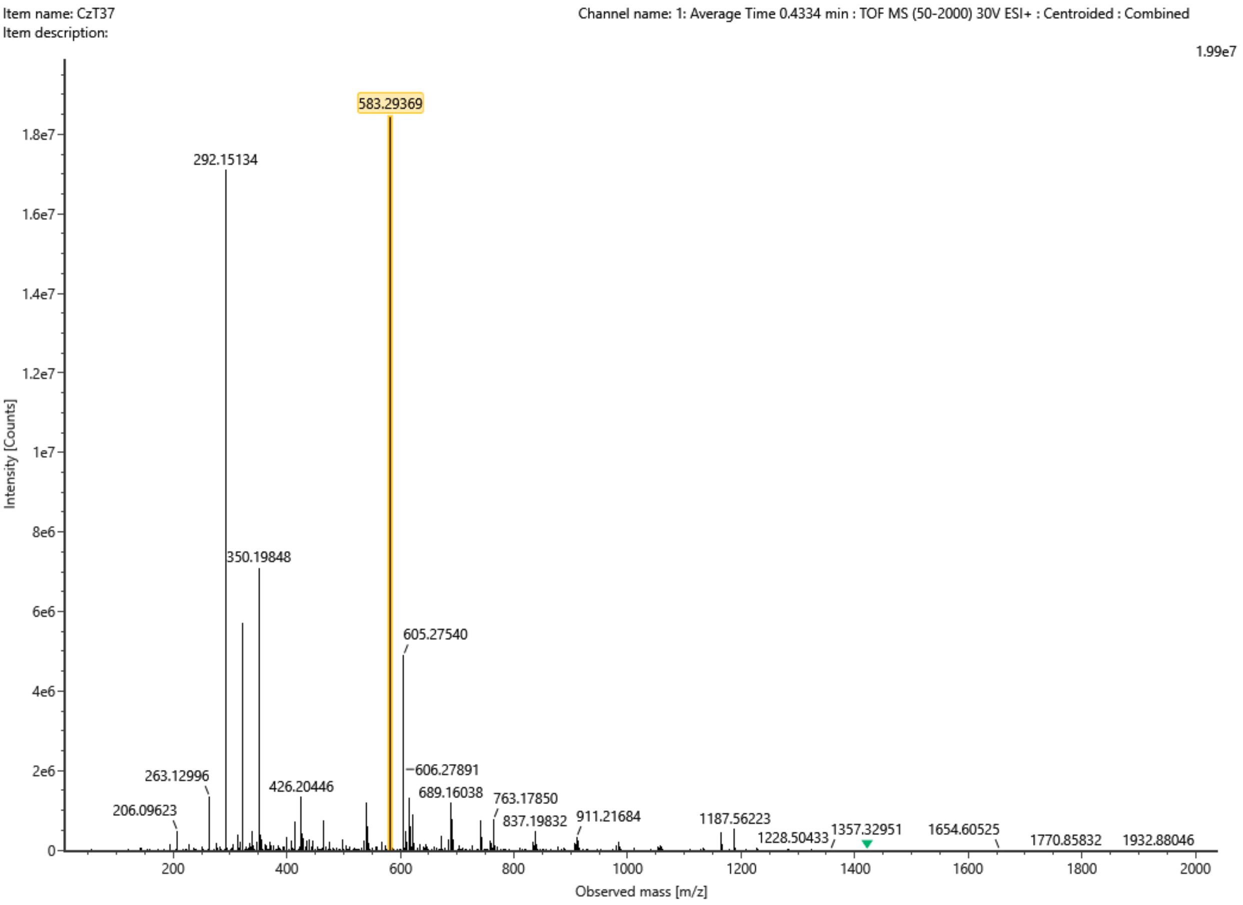
Item name: CzT37
Item description:
Channel name: 1: Average Time 0.4334 min : TOF MS (50-2000) 30V ESI+ : Centroided : Combined
1.99e7
583.29369
292.15134
350.19848
605.27540
606.27891
263.12996
426.20446
689.16038
763.17850
206.09623
837.19832
911.21684
1187.56223
1228.50433
1357.32951
1654.60525
1770.85832
1932.88046
1.8e7
1.6e7
1.4e7
1.2e7
1e7
8e6
6e6
4e6
2e6
0
200
400
600
800
1000
1200
1400
1600
1800
2000
Intensity [Counts]
Observed mass [m/z]

HRMS of **39a**

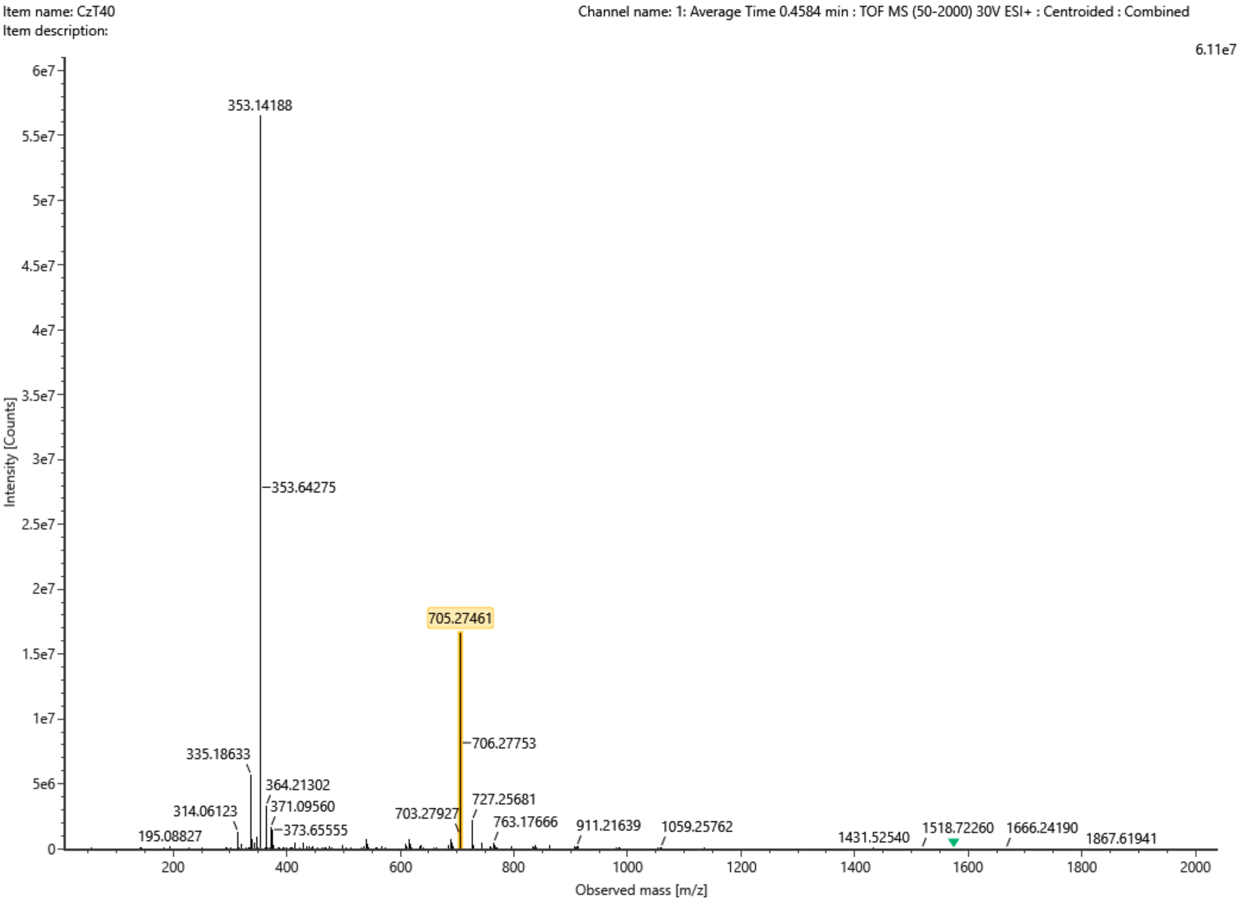

Item name: CzT40
Item description:
Channel name: 1: Average Time 0.4584 min : TOF MS (50-2000) 30V ESI+ : Centroided : Combined
6.11e7
353.14188
353.64275
705.27461
706.27753
335.18633
364.21302
314.06123
371.09560
373.65555
195.08827
703.27927
727.25681
763.17666
911.21639
1059.25762
1431.52540
1518.72260
1666.24190
1867.61941
Intensity [Counts]
Observed mass [m/z]

HRMS of **39b**

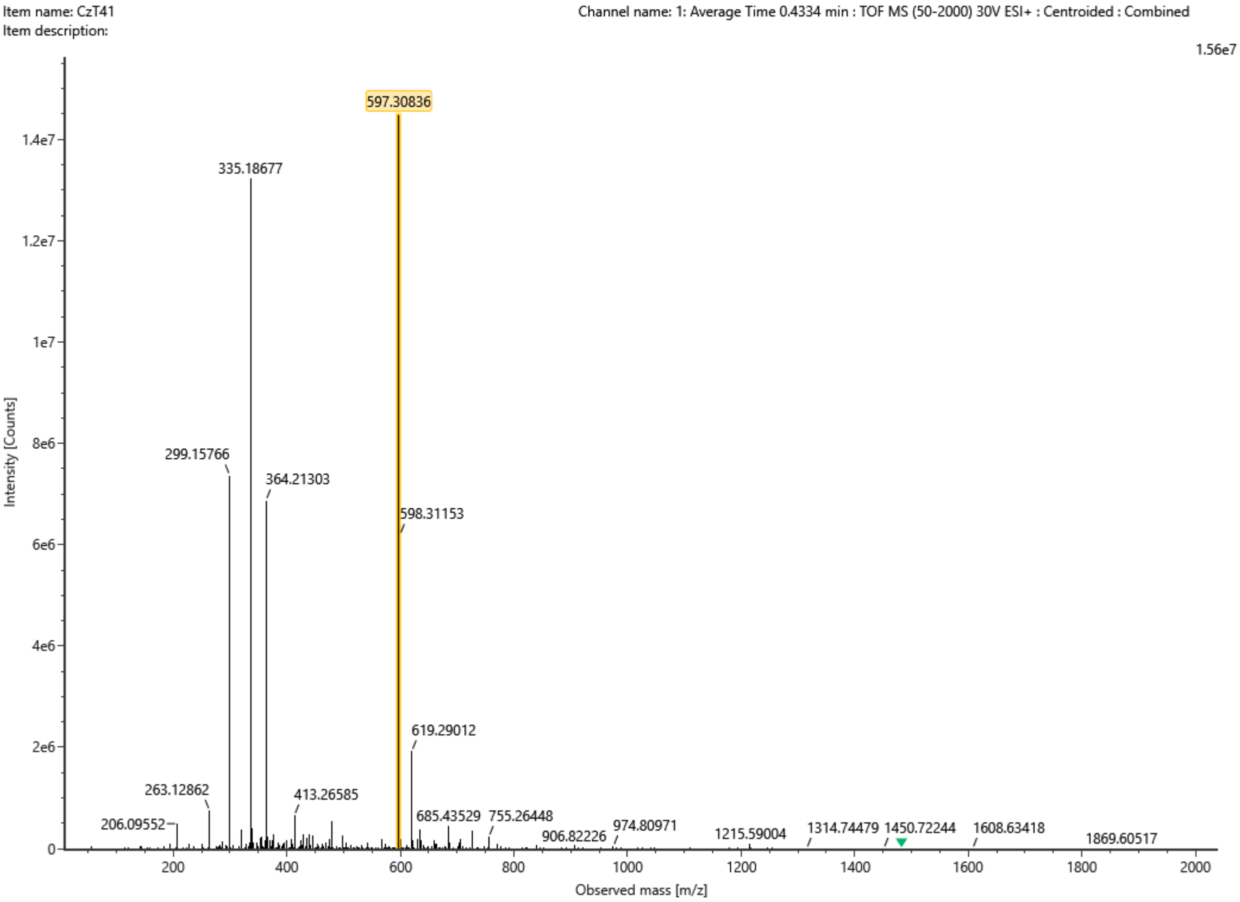

Item name: CzT41
Item description:
Channel name: 1: Average Time 0.4334 min : TOF MS (50-2000) 30V ESI+ : Centroided : Combined
1.56e7
597.30836
335.18677
299.15766
364.21303
598.31153
619.29012
263.12862
413.26585
206.09552
685.43529
755.26448
906.82226
974.80971
1215.59004
1314.74479
1450.72244
1608.63418
1869.60517
Intensity [Counts]
Observed mass [m/z]

HRMS of **40a**

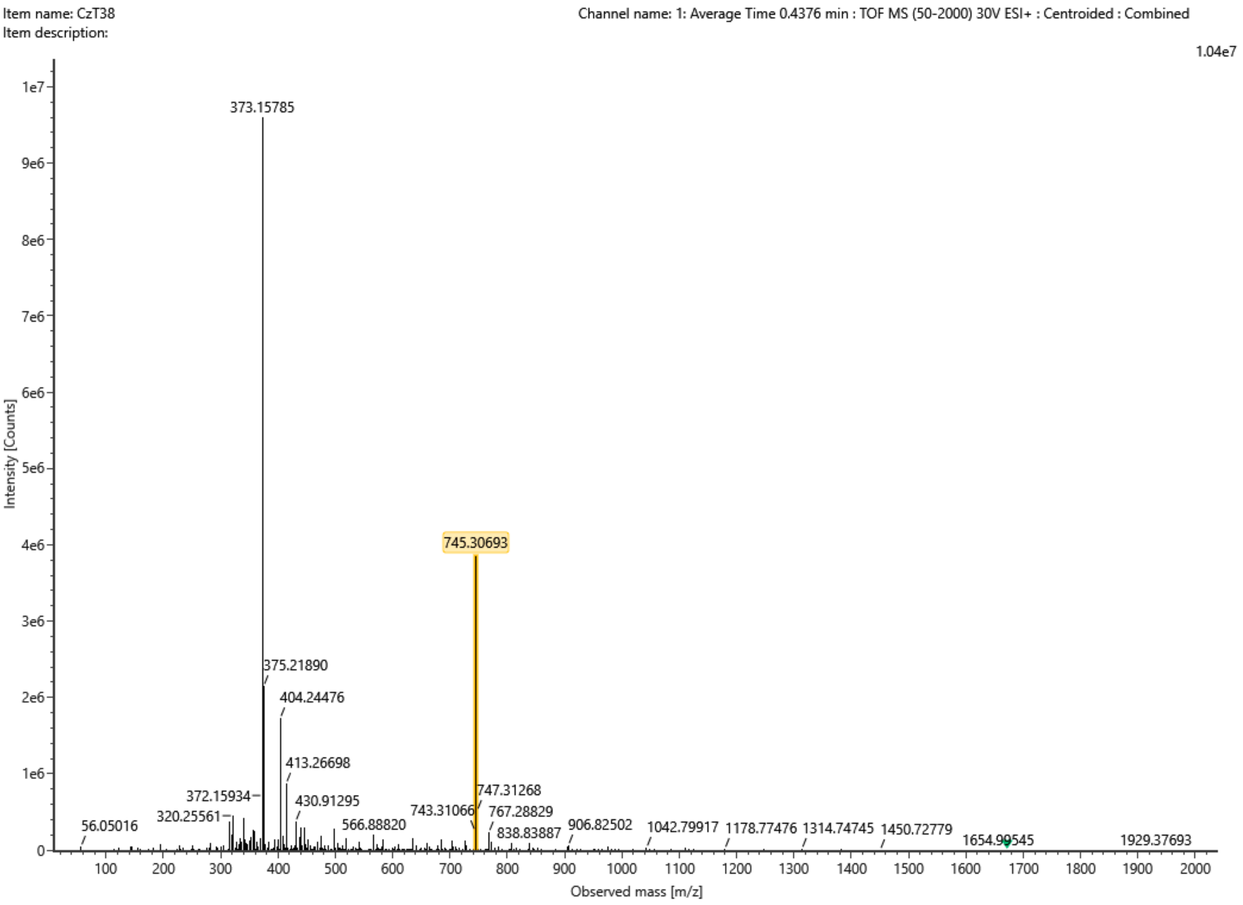

Item name: CzT38
Item description:
Channel name: 1: Average Time 0.4376 min : TOF MS (50-2000) 30V ESI+ : Centroided : Combined
1.04e7
373.15785
745.30693
375.21890
404.24476
413.26698
372.15934
430.91295
320.25561
747.31268
743.31066
767.28829
56.05016
566.88820
838.83887
906.82502
1042.79917
1178.77476
1314.74745
1450.72779
1654.99545
1929.37693
Intensity [Counts]
Observed mass [m/z]

HRMS of **40b**

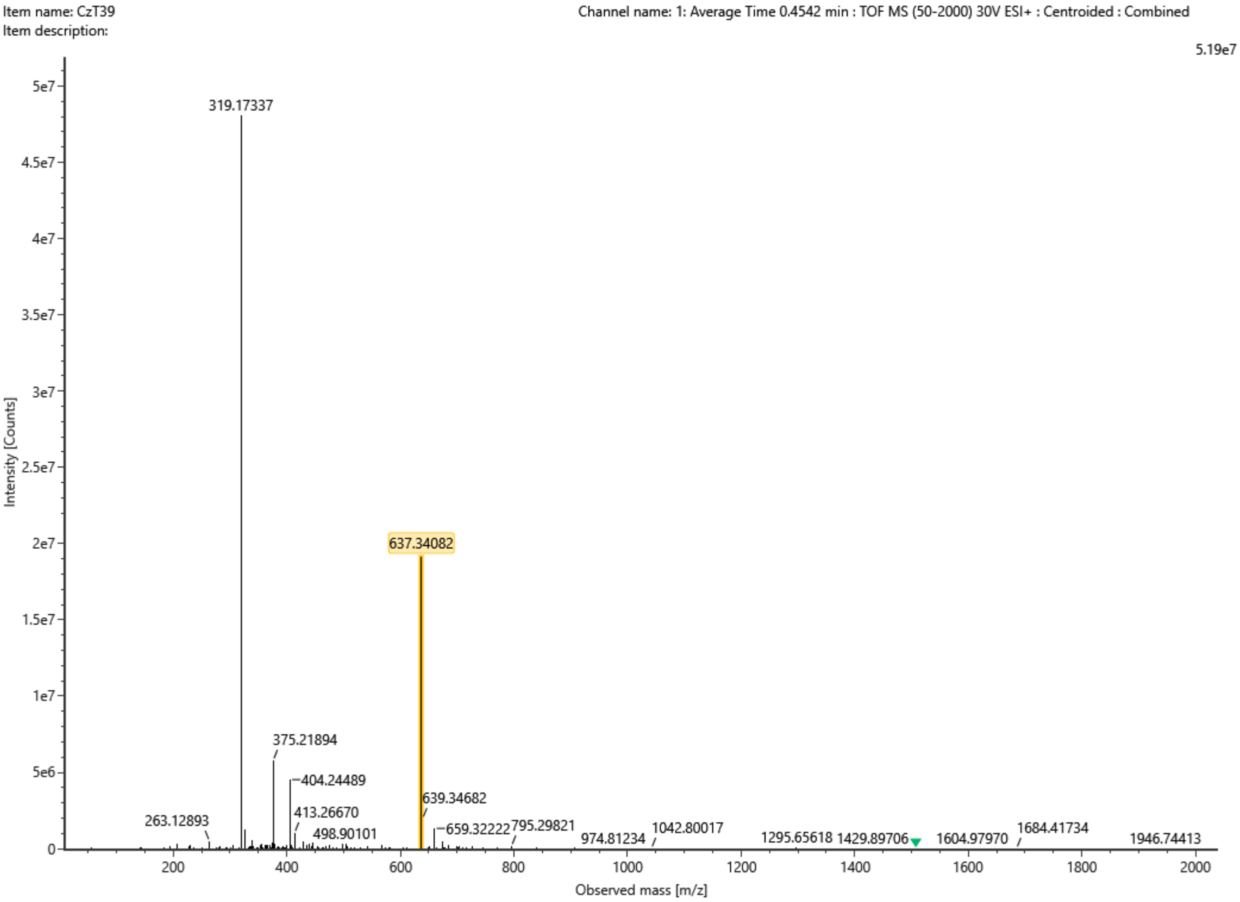

Item name: CzT39
Item description:
Channel name: 1: Average Time 0.4542 min : TOF MS (50-2000) 30V ESI+ : Centroided : Combined
5.19e7
319.17337
637.34082
375.21894
404.24489
413.26670
263.12893
498.90101
639.34682
659.32222
795.29821
974.81234
1042.80017
1295.65618
1429.89706
1604.97970
1684.41734
1946.74413
Intensity [Counts]
Observed mass [m/z]

HRMS of **41a**

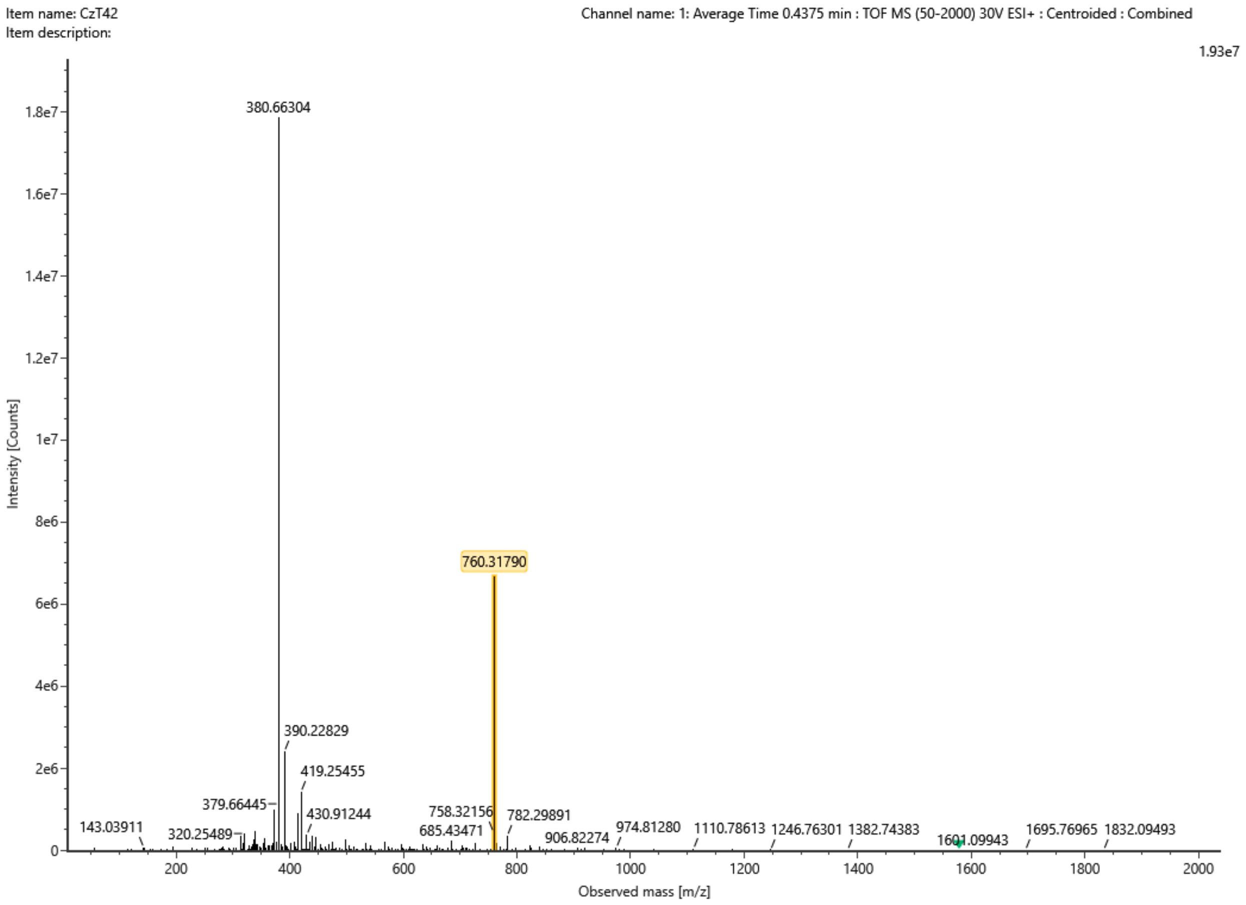
Item name: CzT42
Item description:
Channel name: 1: Average Time 0.4375 min : TOF MS (50-2000) 30V ESI+ : Centroided : Combined
1.93e7
380.66304
760.31790
390.22829
419.25455
379.66445
430.91244
758.32156
782.29891
685.43471
143.03911
320.25489
906.82274
974.81280
1110.78613
1246.76301
1382.74383
1601.09943
1695.76965
1832.09493
Intensity [Counts]
Observed mass [m/z]

HRMS of **41b**

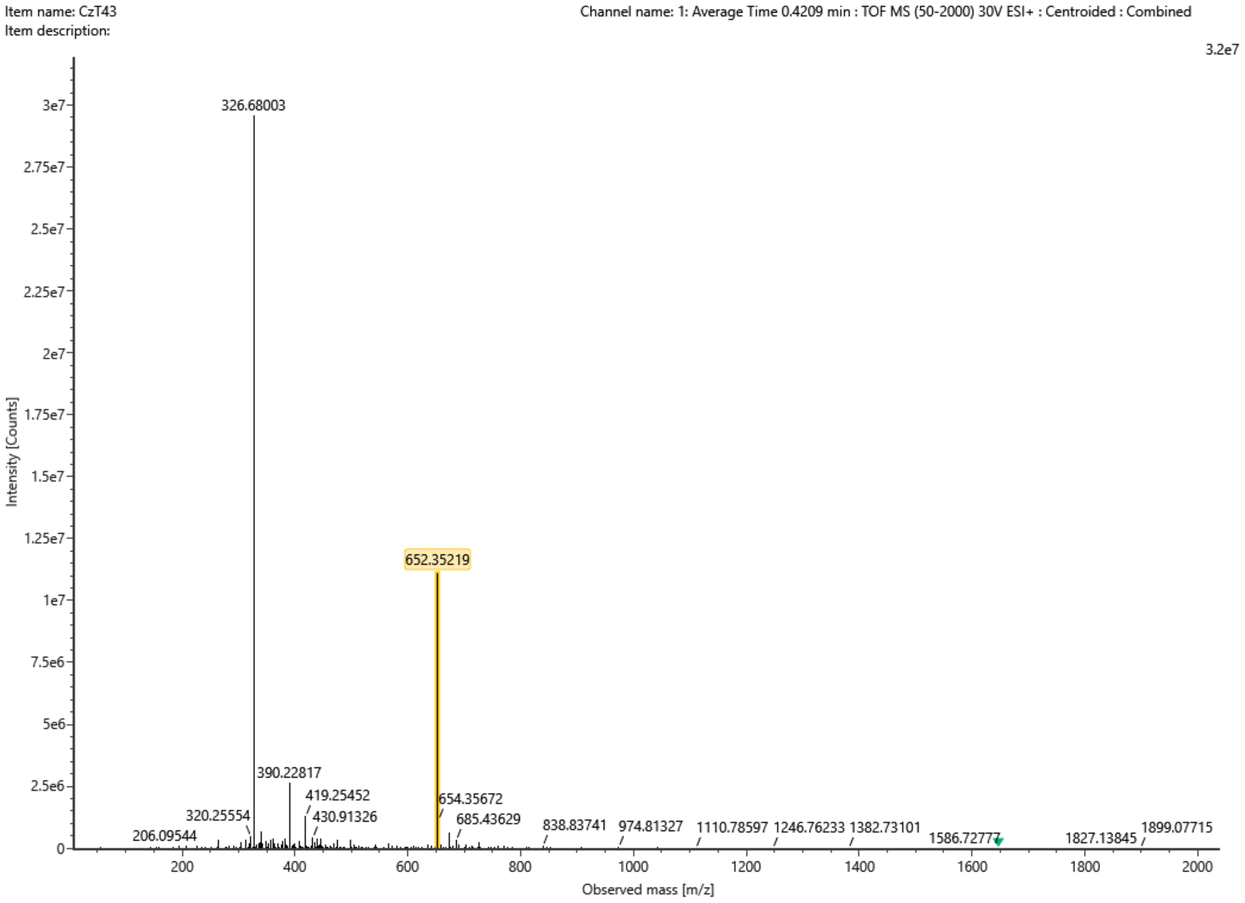

Item name: CzT43
Item description:
Channel name: 1: Average Time 0.4209 min : TOF MS (50-2000) 30V ESI+ : Centroided : Combined
3.2e7
326.68003
652.35219
390.22817
419.25452
430.91326
320.25554
206.09544
654.35672
685.43629
838.83741
974.81327
1110.78597
1246.76233
1382.73101
1586.72777
1827.13845
1899.07715
Intensity [Counts]
Observed mass [m/z]

HRMS of **42a**

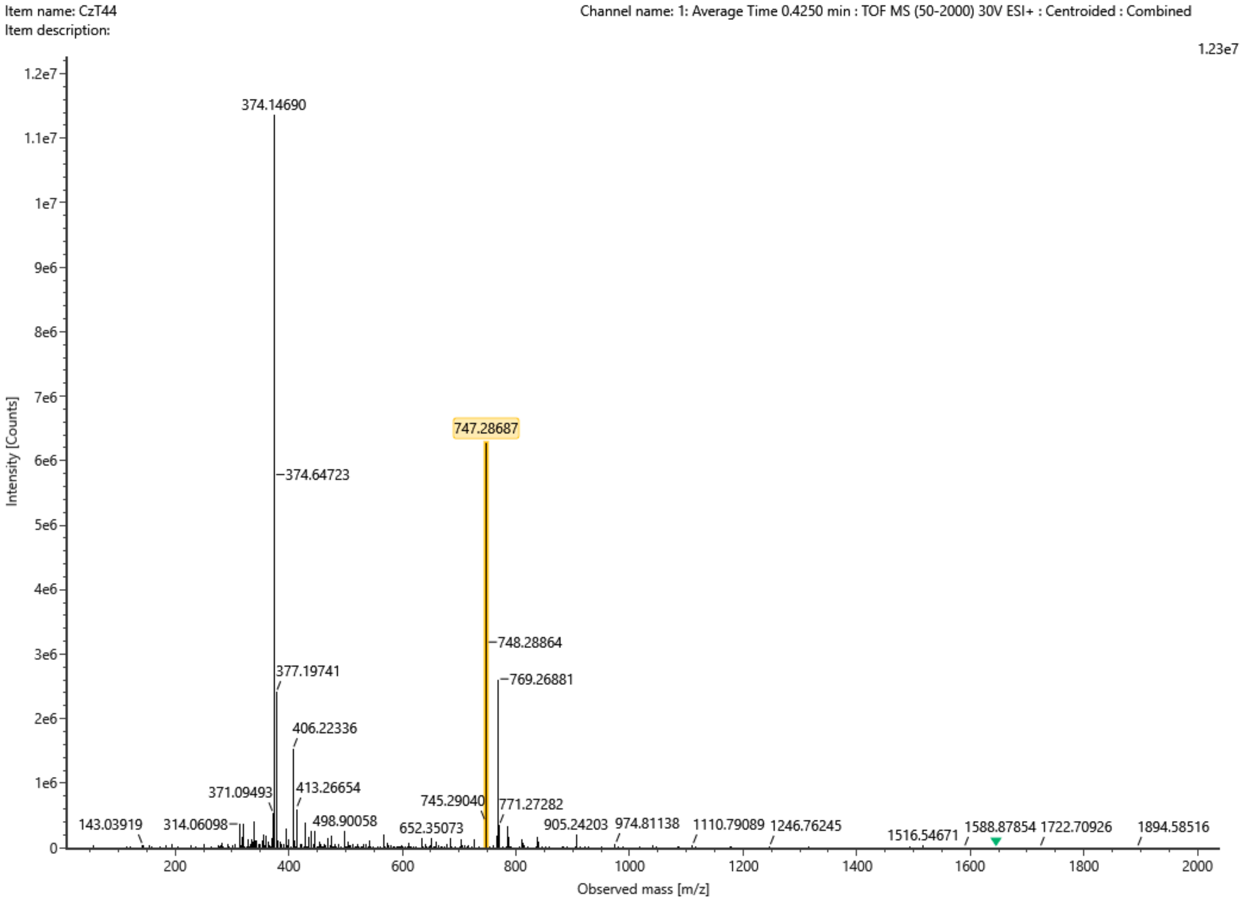

Item name: CzT44
Item description:
Channel name: 1: Average Time 0.4250 min : TOF MS (50-2000) 30V ESI+ : Centroided : Combined
1.23e7
374.14690
374.64723
747.28687
748.28864
769.26881
377.19741
406.22336
413.26654
371.09493
745.29040
771.27282
143.03919
314.06098
498.90058
652.35073
905.24203
974.81138
1110.79089
1246.76245
1516.54671
1588.87854
1722.70926
1894.58516
Intensity [Counts]
Observed mass [m/z]

HRMS of **42b**

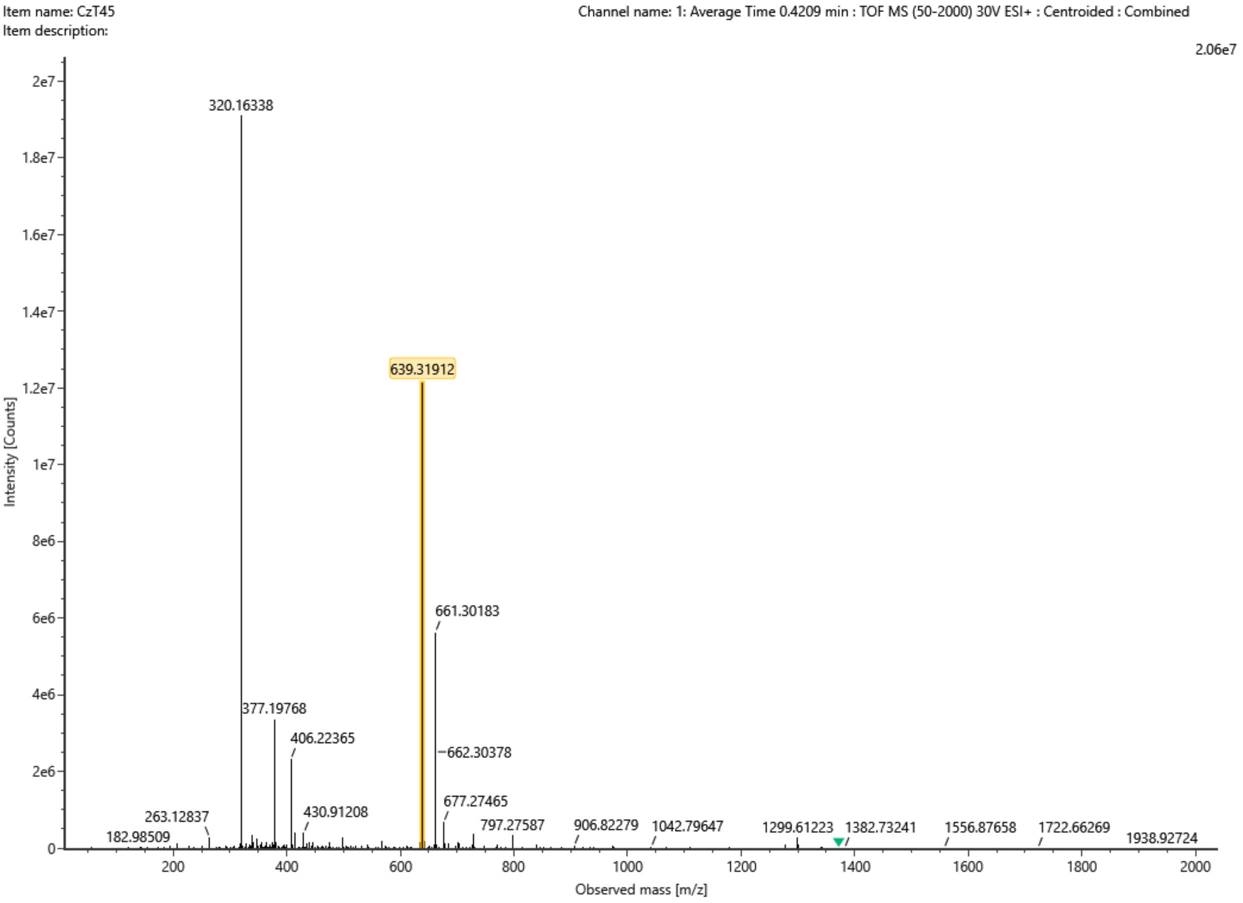

Item name: CzT45
Item description:
Channel name: 1: Average Time 0.4209 min : TOF MS (50-2000) 30V ESI+ : Centroided : Combined
2.06e7
Intensity [Counts]
320.16338
639.31912
661.30183
377.19768
406.22365
662.30378
677.27465
430.91208
263.12837
182.98509
797.27587
906.82279
1042.79647
1299.61223
1382.73241
1556.87658
1722.66269
1938.92724
Observed mass [m/z]

HRMS of **43a**

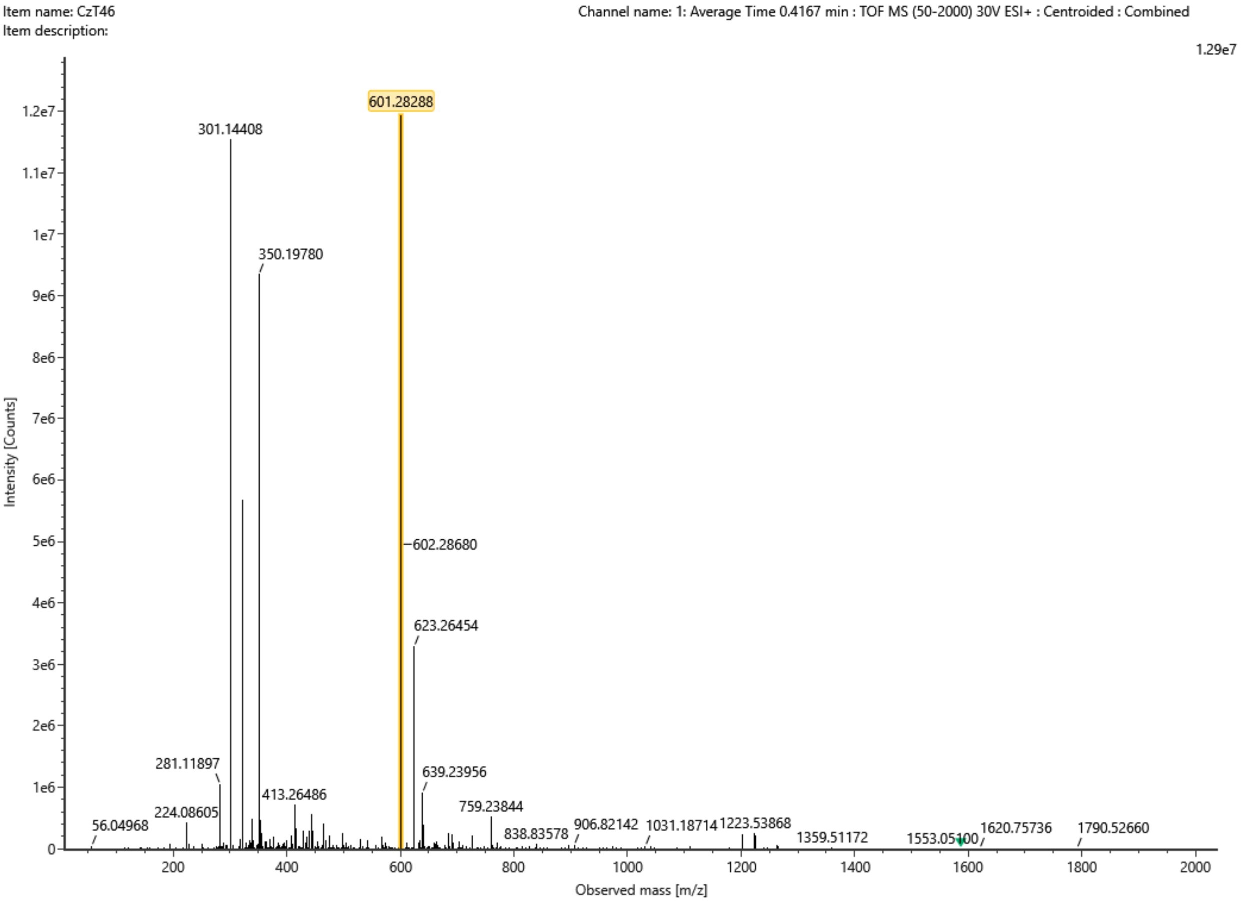

Item name: CzT46
Item description:
Channel name: 1: Average Time 0.4167 min : TOF MS (50-2000) 30V ESI+ : Centroided : Combined
1.29e7
601.28288
301.14408
350.19780
602.28680
623.26454
281.11897
639.23956
413.26486
224.08605
759.23844
56.04968
906.82142
1031.18714
1223.53868
838.83578
1359.51172
1553.05100
1620.75736
1790.52660
Intensity [Counts]
Observed mass [m/z]

HRMS of **43b**

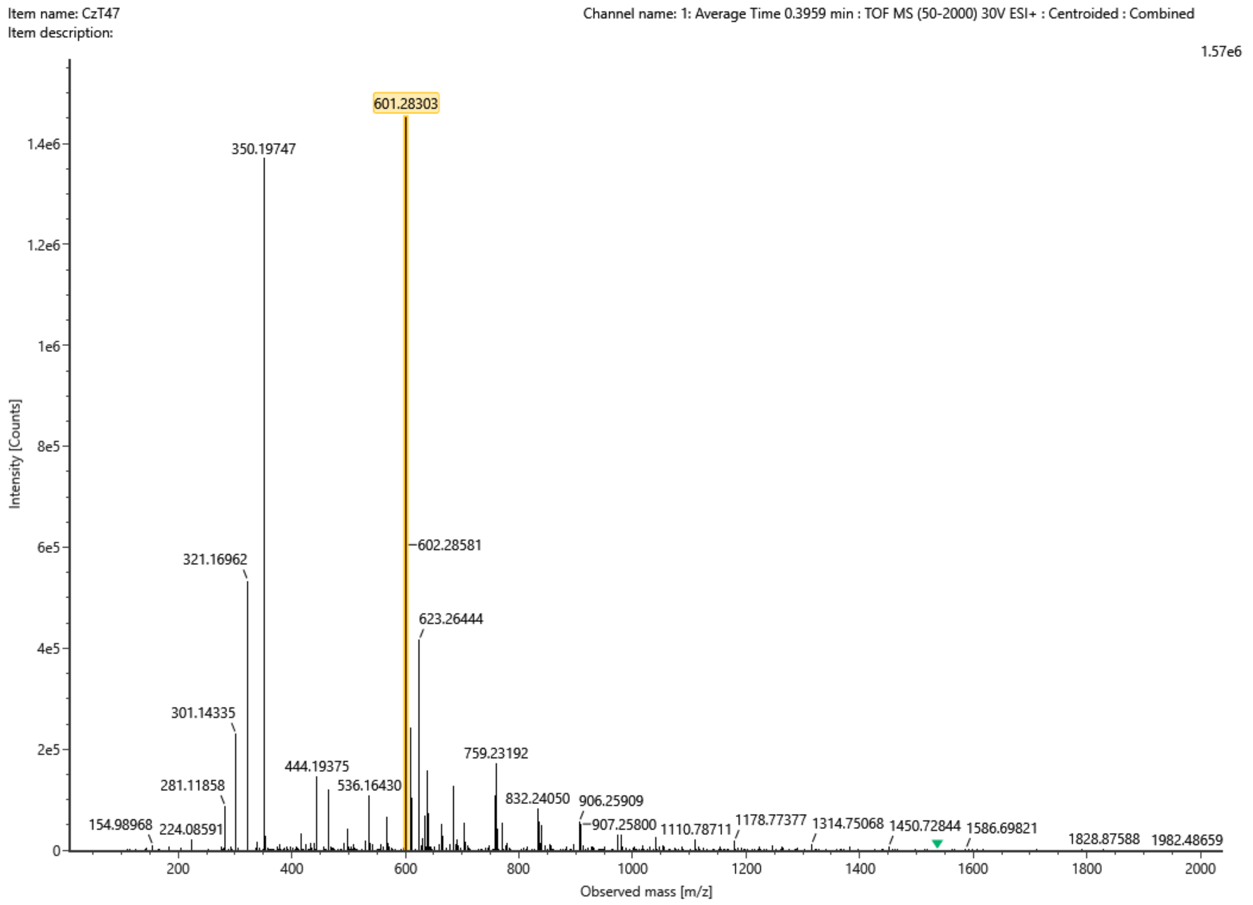

Item name: CzT47
Item description:
Channel name: 1: Average Time 0.3959 min : TOF MS (50-2000) 30V ESI+ : Centroided : Combined
1.57e6
601.28303
350.19747
602.28581
623.26444
321.16962
301.14335
281.11858
444.19375
536.16430
759.23192
832.24050
906.25909
907.25800
1110.78711
1178.77377
1314.75068
1450.72844
1586.69821
1828.87588
1982.48659
154.98968
224.08591
Intensity [Counts]
Observed mass [m/z]

HRMS of **43c**

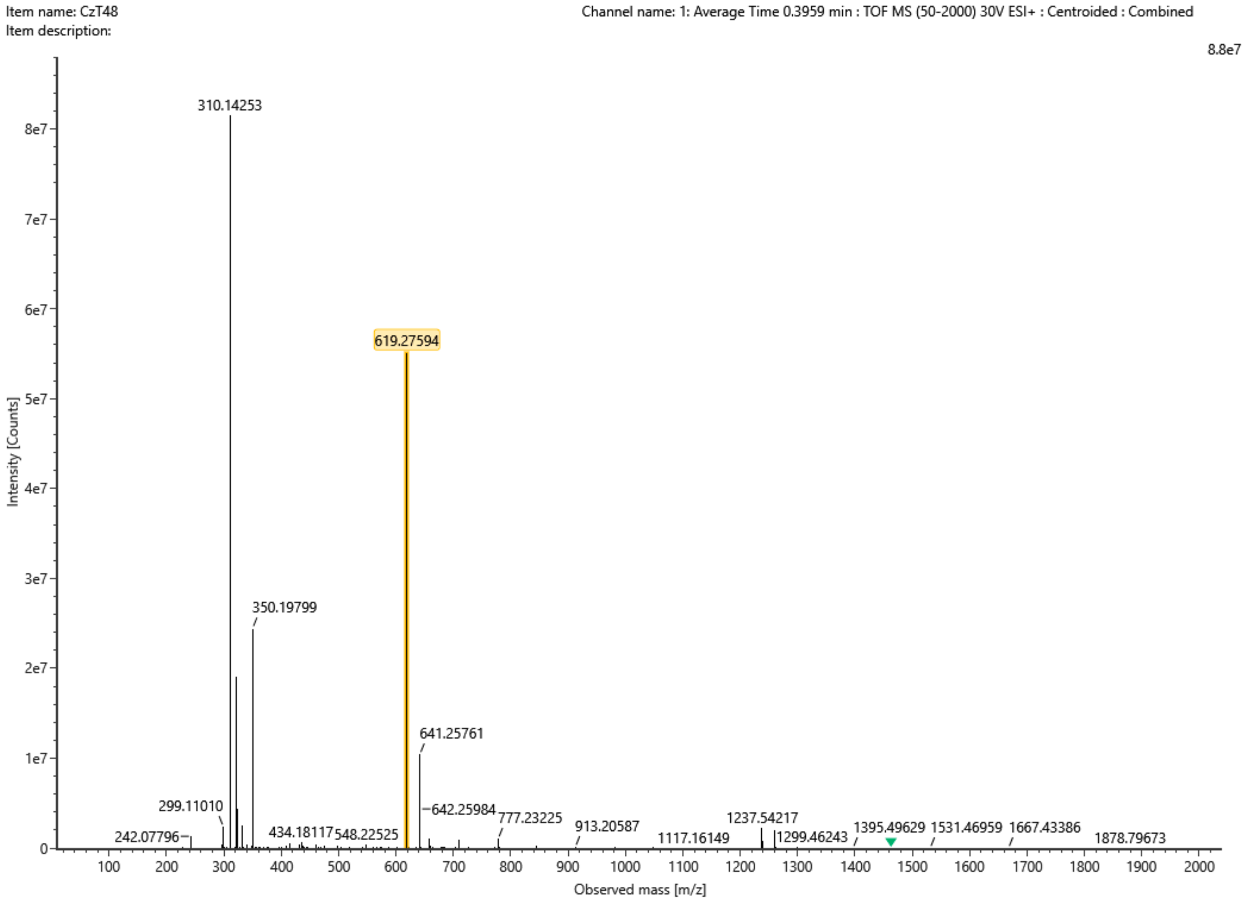

Item name: CzT48
Item description:
Channel name: 1: Average Time 0.3959 min : TOF MS (50-2000) 30V ESI+ : Centroided : Combined
8.8e7
310.14253
619.27594
350.19799
641.25761
299.11010
242.07796
434.18117
548.22525
642.25984
777.23225
913.20587
1117.16149
1237.54217
1299.46243
1395.49629
1531.46959
1667.43386
1878.79673
Intensity [Counts]
Observed mass [m/z]

S.4. HPLC chromatograms of selected compounds

ONC201

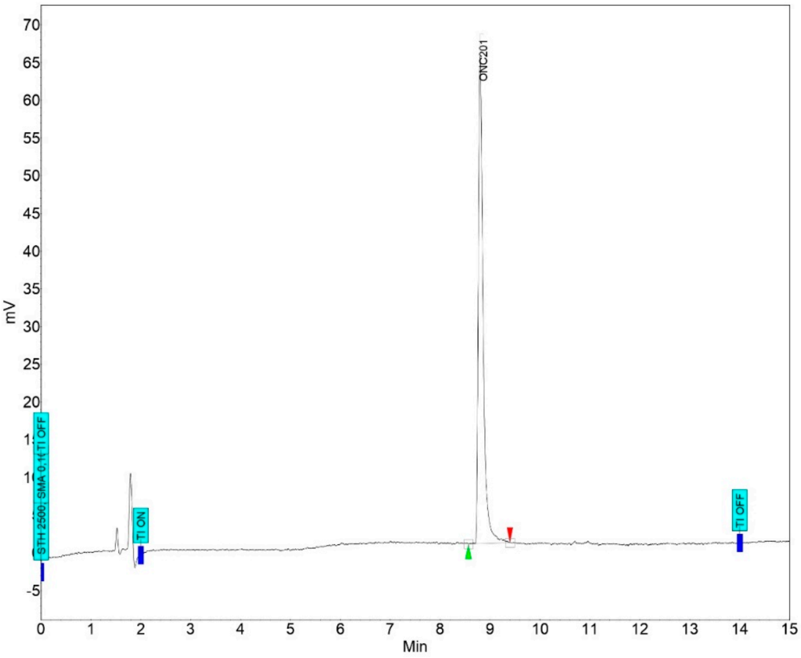

Index	Name	Time [Min]	Quantity [uM]	Height [mV]	Area [mV.Min]	Area % [%]	Quantity [uM]
1	ONC201	8,802	0,00	64,6	7,244	100,000	0,00
Total			0,00	64,6	7,244	100,000	0,00

38a

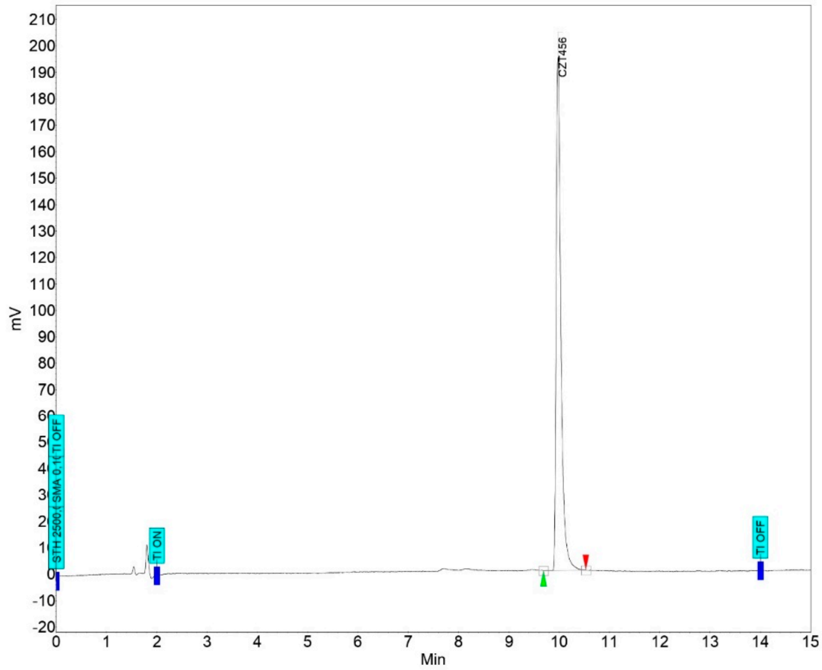

Index	Name	Time [Min]	Quantity [uM]	Height [mV]	Area [mV.Min]	Area % [%]	Quantity [uM]
1	CZT456	9,977	0,00	194,2	21,202	100,000	0,00
Total			0,00	194,2	21,202	100,000	0,00

38b

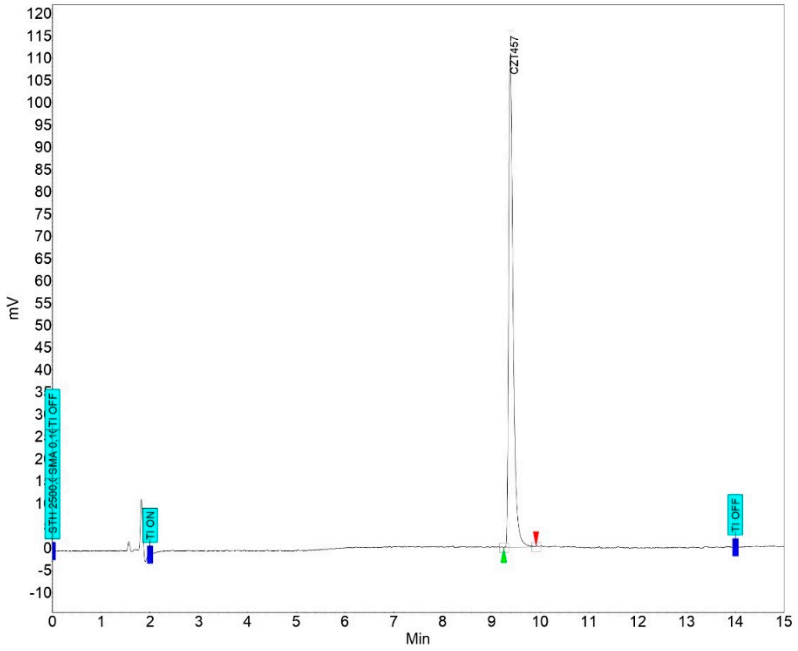

Index	Name	Time [Min]	Quantity [uM]	Height [mV]	Area [mV.Min]	Area % [%]	Quantity [uM]
1	CZT457	9,392	0,00	110,5	11,724	100,000	0,00
Total			0,00	110,5	11,724	100,000	0,00

S5. CellTiter-Glo Cell Viability Assay Data

PANC-1 Cell viability (% of controll)										
***c* [μM]**	**26**	**17.3**	**11.6**	**7.7**	**5.1**	**3.4**	**2.3**	**1.5**	**1**	**0.7**
	33	37	43	48	54	60	84	101	101	90
**ONC201**	32	34	37	46	49	64	72	85	94	92
	29	35	38	43	51	57	78	86	102	97
	0	2	54	109	119	112	111	115	115	106
**I/A**	0	0	32	93	99	108	109	110	109	102
	0	1	39	79	97	109	107	105	116	107
	0	0	1	90	116	113	113	111	107	102
**I/B**	0	0	1	72	112	108	112	111	111	106
	0	0	2	72	109	108	108	108	106	104
	0	0	0	0	0	2	115	108	110	112
**38a**	0	0	0	0	0	2	97	116	107	107
	0	0	0	0	0	4	105	119	114	109
	0	0	0	0	0	1	98	116	108	110
**38b**	0	0	0	0	0	0	81	109	110	103
	0	0	0	0	0	0	79	107	109	103
	0	0	0	0	0	0	107	112	115	114
**43a**	0	0	0	0	0	0	93	102	110	108
	0	0	0	0	0	0	84	102	112	111
	0	0	0	0	0	7	103	110	104	104
**43b**	0	0	0	0	0	13	106	104	104	103
	0	0	0	0	0	10	102	99	107	101
	0	0	0	0	0	8	82	103	105	104
**43c**	0	0	0	0	0	6	89	100	104	110
	0	0	0	0	0	5	80	103	99	102

Fadu Cell viability (% of controll)										
***c*[μM]**	**26**	**17.3**	**11.6**	**7.7**	**5.1**	**3.4**	**2.3**	**1.5**	**1**	**0.7**
	31	33	35	38	39	40	55	66	85	94
**ONC201**	22	23	24	28	26	25	31	59	86	99
	24	24	27	27	26	26	30	51	77	93
	0	5	47	99	105	104	109	107	110	111
**I/A**	0	0	19	86	106	109	109	108	112	115
	0	1	28	82	102	110	104	109	109	108
	0	0	46	93	104	105	110	109	112	106
**I/B**	0	0	22	100	107	112	112	108	116	110
	0	0	30	97	107	109	109	114	109	109
	0	0	0	0	0	43	96	102	110	109
**38a**	0	0	0	0	0	13	91	103	106	115
	0	0	0	0	0	13	87	100	105	106
	0	0	0	0	10	70	104	104	113	103
**38b**	0	0	0	0	1	37	99	105	109	108
	0	0	0	0	2	29	98	106	105	109
	0	0	0	0	5	71	95	103	112	108
**43a**	0	0	0	0	0	29	80	104	108	109
	0	0	0	0	0	38	88	103	109	108
	0	0	0	0	1	58	91	105	102	108
**43b**	0	0	0	0	0	46	96	102	106	108
	0	0	0	0	0	27	91	102	101	104
	0	0	0	0	0	79	89	90	97	94
**43c**	0	0	0	0	0	59	90	97	102	99

	0	0	0	0	0	42	79	97	93	100

Primer fibroblast Cell viability (% of controll)										
***c* [µM]**	**26**	**17.3**	**11.6**	**7.7**	**5.1**	**3.4**	**2.3**	**1.5**	**1**	**0.7**
**ONC201**	65	67	68	69	82	109	92	98	99	99
	85	92	92	97	99	107	108	104	104	96
	87	87	88	94	96	111	102	110	103	95
**I/A**	0	42	84	109	98	104	104	104	99	96
	0	8	92	115	107	104	102	102	100	98
	0	24	93	118	109	107	102	105	103	99
**I/B**	0	0	33	104	98	98	98	98	100	93
	0	0	42	92	104	101	100	105	99	98
	0	0	21	107	110	109	107	108	104	97
**38a**	0	0	0	0	9	100	101	100	100	97
	0	0	0	0	9	108	105	105	111	105
	0	0	0	0	6	108	107	113	113	109
**38b**	0	0	0	0	8	101	103	106	101	103
	0	0	0	0	6	112	116	106	112	107
	0	0	0	0	5	110	107	118	117	109
**43a**	0	0	0	0	0	29	104	99	106	99
	0	0	0	0	0	35	111	114	109	109
	0	0	0	0	0	34	101	125	108	107
**43b**	0	0	0	0	0	50	99	97	103	96
	0	0	0	0	0	89	105	107	102	105
	0	0	0	0	0	54	102	105	113	104
**43c**	0	0	0	0	0	80	103	99	92	91
	0	0	0	0	0	95	97	95	104	98
	0	0	0	0	0	99	98	118	106	102

HEK293T WT Cell viability (% of controll)										
***c* [µM]**	**26**	**16.25**	**10.2**	**6.4**	**4.0**	**2.5**	**1.6**	**1.0**	**0.6**	**0.4**
**ONC201**	15	15	19	18	19	21	28	30	60	93
	15	18	18	19	19	19	25	28	70	105
	16	18	20	22	22	22	29	32	65	96
	18	19	20	20	20	23	28	31	68	95
**38a**	0	0	0	0	0	11	65	82	106	106
	0	0	0	0	0	6	60	90	109	101
	0	0	0	0	0	5	52	94	81	99
	0	0	0	0	0	5	52	81	106	100
**38b**	0	0	0	0	0	6	64	109	107	121
	0	0	0	0	0	15	66	110	110	116
	0	0	0	0	0	12	67	107	120	117
	0	0	0	0	0	12	83	102	116	121

HEK293T CLPP-/- Cell viability (% of controll)										
**c [µM]**	**26**	**16.25**	**10.2**	**6.4**	**4.0**	**2.5**	**1.6**	**1.0**	**0.6**	**0.4**
**ONC201**	101	108	110	109	109	107	109	104	107	106
	109	112	112	109	112	113	118	116	113	109
	100	111	110	103	108	108	115	110	103	109
	97	120	124	125	113	118	123	117	106	110
**CZT456**	0	0	0	0	1	41	92	105	111	109

	0	0	0	0	1	48	93	106	110	108
	0	0	0	0	8	59	96	107	111	110
	0	0	0	0	9	55	103	105	109	114
**CZT457**	0	0	0	0	0	13	103	115	112	113
	0	0	0	0	1	25	100	118	117	116
	0	0	0	0	0	33	107	112	112	114
	0	0	0	0	0	32	101	111	110	119

Goodness of fit: R^2 Values of Nonlinear Regression for IC_{50} Determination Across Different Cell Lines and Compounds

R^2	**ONC201**	**I/A**	**I/B**	**38a**	**38b**	**43a**	**43b**	**43c**
PANC-1	0.9659	0.9782	0.994	0.9956	0.9956	0.9945	0.9986	0.9982
Fadu	0.9384	0.988	0.9922	0.9891	0.9852	0.9818	0.9911	0.9821
Primary fibroblast	0.5474	0.9693	0.9865	0.9956	0.9950	0.9928	0.9842	0.9895
HEK293T WT	0.9839			0.9864	0.9949			
HEK293T CLPP-/-	0.003094			0.9956	0.9966			

For the values marked in red, only curve fitting was performed without deriving IC_{50} values, which are indicated as "n.d." (not determined) in Table 3.

S.6. Comparison of IC_{50} Values for Novel Imipridone Hybrids Across Cell Lines Using One-Way ANOVA Analysis Relative to ONC201.

	**PANC-1 IC_{50} values [μM]**							
	**ONC201**	**I/A**	**I/B**	**38a**	**38b**	**43a**	**43b**	**43c**
n1	3.222	11.494	8.429	2.786	2,581	2,492	2,876	2,632
n2	3.141	10.273	7.987	2.620	2,391	2,489	3,260	2,672
n3	2.776	10.112	8.151	2.716	2,379	2,467	3,048	2,593
Mean	3.05	10.63	8.19	2.71	2,45	2,48	3,06	2,63
SEM	0.14	0.44	0.13	0.05	0,07	0,01	0,11	0,02
p value		<0.0001	<0.0001	0.6159	0,1308	0,1643	>0,9999	0,4243

	**Fadu IC_{50} values [μM]**							
	**ONC201**	**I/A**	**I/B**	**38a**	**38b**	**43a**	**43b**	**43c**
n1	1.347	11.169	11.032	3.200	3,720	3,703	3,480	3,715
n2	1.408	9.272	9.908	2.748	3,137	2,805	3,276	3,553
n3	1.257	9.606	10.191	2.751	3,033	3,039	2,990	3,180
Mean	1.34	10.02	10.38	2.90	3,30	3,18	3,25	3,48
SEM	0.04	0.58	0.34	0.15	0,21	0,27	0,14	0,16
p value		<0.0001	<0.0001	0.0072	0,001	0,0017	0,0012	0,0004

	**Primary fibroblast IC_{50} values [μM]**							
	**ONC201**	**I/A**	**I/B**	**38a**	**38b**	**43a**	**43b**	**43c**
n1	*5.082*	16.776	11.397	4.795	4,346	3,318	3,402	3,570
n2	*9.938*	13.885	11.082	4.801	4,759	3,321	3,571	3,757
n3	*6.481*	14.931	11.132	4.281	4,241	3,054	3,406	3,786
Mean	7.17	15.20	11.20	4.63	4,45	3,23	3,46	3,70
SEM	1.44	0.85	0.10	0.17	0,16	0,09	0,06	0,07
p value		<0.0001	0.0012	0.0434	0,0288	0,0016	0,0027	0,0049

	**HEK293T WT IC_{50} values [μM]**		
	**ONC201**	**38a**	**38b**
n1	0.394	1.605	1.610
n2	0.626	1.499	1.663
n3	0.558	1.652	1.628
n4	0.643	1.624	1.795
Mean	0.56	1.60	1.67
SEM	0.06	0.03	0.04
p value		<0.0001	<0.0001

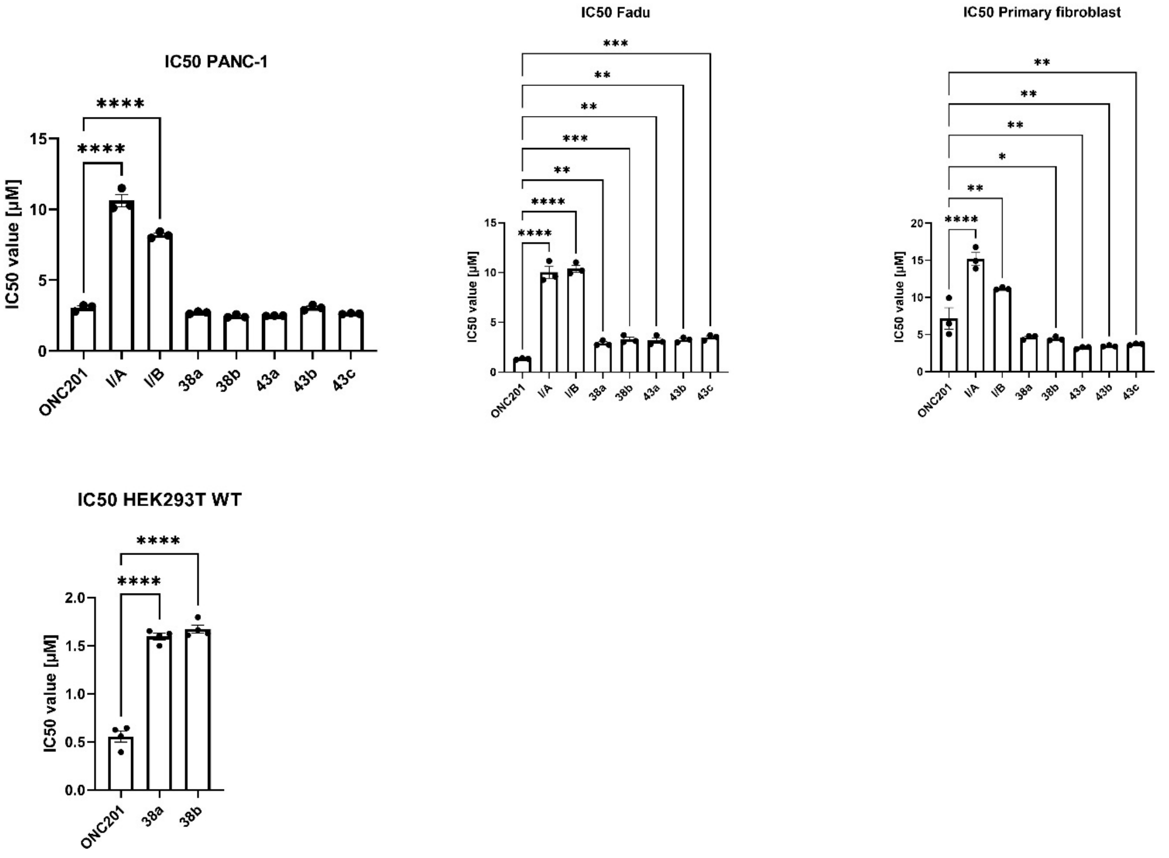

This figure presents the IC_{50} values of **ONC201** and novel imipridone hybrids in four cell lines (PANC-1, Fadu, Primary fibroblasts, and HEK293T WT), calculated using nonlinear regression (four-parameter logistic model) with GraphPad Prism. For each replicate measurement, an IC_{50} value was independently determined. These values were then used to calculate mean IC_{50} values, SEM, and perform one-way ANOVA for statistical comparison.

Differences from Table 3: The IC_{50} values presented here differ from those in Table 3, as this analysis relies on independently calculated IC_{50} values for each replicate measurement. This method provides a more granular understanding of variability across replicates and allows for robust statistical comparisons.

Statistical Approach: One-way ANOVA was performed to compare the IC_{50} values of novel hybrids to **ONC201** for each cell line separately. ANOVA assumptions, including normality (assessed via QQ plots) and homogeneity of variances (tested with the Brown-Forsythe test), were verified. The assumption of independence was met, as the comparisons are based on three independent experimental replicates for each compound and cell line. Significance levels are indicated by stars above the bars: $p < 0.05$, $p < 0.01$, $p < 0.00$, $p < 0.0001$.

PANC-1 and Fadu Cell Lines: The novel hybrids (**38a**, **38b**, **43a**, **43b**, and **43c**) exhibited superior efficacy compared to **ONC201**, as indicated by their lower IC_{50} values and their ability to completely eliminate cancer cells. **I/A** and **I/B**, in contrast, showed significantly higher IC_{50} values, reflecting reduced potency. While **ONC201** has lower absolute IC_{50} values for Fadu cells (1.34 µM), the hybrids' complete eradication capability highlights their enhanced therapeutic potential.

Primary Fibroblasts: The IC_{50} value for **ONC201** could not be directly determined due to the fibroblast viability remaining above 50% in the tested concentration range. This reflects a lower cytotoxicity of **ONC201** under these conditions (as seen in Figure 5). GraphPad software mathematically estimated an IC_{50} value based on the inflection point of the curve, assuming symmetry. While this does not represent true toxicity, it allowed for statistical comparisons with the novel hybrids, which exhibited significantly higher fibroblast cytotoxicity.

HEK293T WT Cells: The IC_{50} values for hybrids **38a** and **38b** were significantly higher than ONC201, suggesting reduced off-target effects and enhanced safety profiles in non-cancerous cells.

The novel imipridone hybrids demonstrate not only comparable or lower IC_{50} values for cancer cell lines but, more importantly, exhibit the ability to completely eliminate tumor cells, a feature not observed with **ONC201** under the same conditions. This eradication capability highlights the superior therapeutic potential of these compounds.
